# Saturn Anomalous Myriametric Radiation, a New Type of Saturn Radio Emission Revealed by Cassini

**DOI:** 10.1029/2022GL099237

**Published:** 2022-08-19

**Authors:** S. Y. Wu, S. Y. Ye, G. Fischer, U. Taubenschuss, C. M. Jackman, E. O'Dwyer, W. S. Kurth, S. Yao, Z. H. Yao, J. D. Menietti, Y. Xu, M. Y. Long, B. Cecconi

**Affiliations:** ^1^ Department of Earth and Space Sciences Southern University of Science and Technology Shenzhen People's Republic of China; ^2^ LESIA Observatoire de Paris Université PSL CNRS Sorbonne Université Université de Paris Meudon France; ^3^ Space Research Institute Austrian Academy of Sciences Graz Austria; ^4^ Department of Space Physics Institute of Atmospheric Physics of the Czech Academy of Sciences Prague Czechia; ^5^ School of Cosmic Physics DIAS Dunsink Observatory Dublin Institute for Advanced Studies Dublin Ireland; ^6^ Department of Physics and Astronomy University of Iowa Iowa City IA USA; ^7^ School of Geophysics and Information Technology China University of Geosciences (Beijing) Beijing People's Republic of China; ^8^ Key Laboratory of Earth and Planetary Physics Institute of Geology and Geophysics Chinese Academy of Sciences Beijing People's Republic of China; ^9^ Department of Space Physics School of Electronic Information Wuhan University Wuhan People's Republic of China

## Abstract

A new radio component namely Saturn Anomalous Myriametric Radiation (SAM) is reported. A total of 193 SAM events have been identified by using all the Cassini Saturn orbital data. SAM emissions are L‐O mode radio emission and occasionally accompanied by a first harmonic in R‐X mode. SAM's intensities decrease with increasing distance from Saturn, suggesting a source near Saturn. SAM has a typical central frequency near 13 kHz, a bandwidth greater than 8 kHz and usually drifts in frequency over time. SAM's duration can extend to near 11 hr and even longer. These features distinguish SAM from the regular narrowband emissions observed in the nearby frequency range, hence the name anomalous. The high occurrence rate of SAM after low frequency extensions of Saturn Kilometric Radiation and the SAM cases observed during compressions of Saturn's magnetosphere suggest a special connection to solar wind dynamics and magnetospheric conditions at Saturn.

## Key Points

- A new radio component namely the Saturn Anomalous Myriametric Radiation (SAM) is reported
- The characteristics of SAM are given including its spatial distribution, typical frequency, bandwidth and time duration
- SAM shows a possible connection to the compression of the magnetosphere

## 1 Introduction

Saturnian radio emissions, such as the Saturn Kilometric Radiation (SKR) (Kaiser et al., 1980; Lamy et al., 2008), 5 kHz Narrowband emissions (5 kHz NB or narrowband Saturn Myriametric radiation (n‐SMR) (Gurnett et al., 1981; Louarn et al., 2007; Ye et al., 2010), and 20 kHz Narrowband emissions (Lamy et al., 2008; Ye et al., 2011), have been studied for several decades. Discovered by the Voyager 1 planetary radio experiment (Kaiser et al., 1980), SKR is right hand circularly/elliptically polarized with respect to the source magnetic field with a frequency range of 3 kHz to 1.2 MHz (Fischer et al., 2009; Lamy et al., 2008). SKR is generated through the cyclotron maser instability in the auroral region (Lamy et al., 2008, 2018; Wu & Lee, 1979; Zarka, 1998). NB emissions were discovered by Voyager 1 in the 1980s as well (Gurnett et al., 1981; Scarf & Gurnett, 1977). NB emissions are observed in stable frequency ranges near 5 and 20 kHz, with a much narrower bandwidth in contrast to the broad‐banded SKR (Ye et al., 2011). They are thought to be generated through mode conversion processes (Gurnett et al., 1981; Ye et al., 2009, 2010). The L‐O mode NB emissions (for both 5 and 20 kHz) can be converted from Z‐mode NB emissions that have been shown to be generated by non‐thermal plasma distributions characterized by a loss cone and temperature anisotropy (Menietti et al., 2010, 2011, 2016, 2018).

Radio emissions are closely connected to magnetospheric activities in the Saturnian magnetosphere (Louarn et al., 2007; Mitchell et al., 2015; Wing et al., 2020). The power of SKR is strongly correlated with solar wind characteristics (Desch, 1982; Desch & Rucker, 1983; Jackman et al., 2010; Taubenschuss et al., 2006). A typical phenomenon, namely the low frequency extension (LFE) of SKR, which is an expansion of the entire kilometric spectrum and in particular of the main band from high to low frequencies, is shown to be a good proxy for reconnection events and compression‐induced hot plasma injections in Saturn's magnetosphere (Bunce et al., 2005; Jackman & Arridge, 2011; Jackman et al., 2009, 2010; Reed et al., 2018). The LFE of SKR can be explained by the variation of the SKR source region, which would extend to a higher altitude along the magnetic field lines as a direct consequence of the precipitation of energetic particles along field lines into the auroral zone (Bunce et al., 2005; Jackman et al., 2009), and this feature is also commonly seen at Earth (Morioka et al., 2007, 2014). The SKR enhancement can be related to so‐called type‐1 plasma injection events (Mitchell et al., 2015), which are related to current sheet collapse and magnetotail reconnection at radial distances beyond ∼12 Rs, (Rs: Saturn radius = 60,268 km). Recently, the 5 kHz NB emissions have been found to be connected to type‐2 injection events (Wing et al., 2020), which are related to the interchange instability in the inner magnetosphere at a smaller radial distance (Hill, 1976).

We identify for the first time a new radio component which is usually observed near 10 kHz. Emissions at 10 kHz were observed in previous works (Menietti et al., 2009; Wing et al., 2020), but were simply referred to as NB emissions observed near 10 kHz. We name this type of emission Saturn Anomalous Myriametric radiation (SAM) based on their differences with the 5 and 20 kHz NB emissions (that can sometimes extent up or down to 10 kHz), such as the average wavelength, duration of the SAM signals and the morphology in the electric field spectrogram. Examples of the newly defined SAM emissions are shown in Section 2 and their characteristics are shown in Section 3. The main characteristics of SAM are a larger frequency drift, a larger bandwidth, and a longer duration compared to ordinary NB emissions around 10 kHz. This justifies their identification, isolation and separate study in this work. We also discuss these events in their broader magnetospheric context, exploring their link to SKR bursts and LFEs (Section 4).

## 2 Examples of the SAM Emission

**Figure 1:**
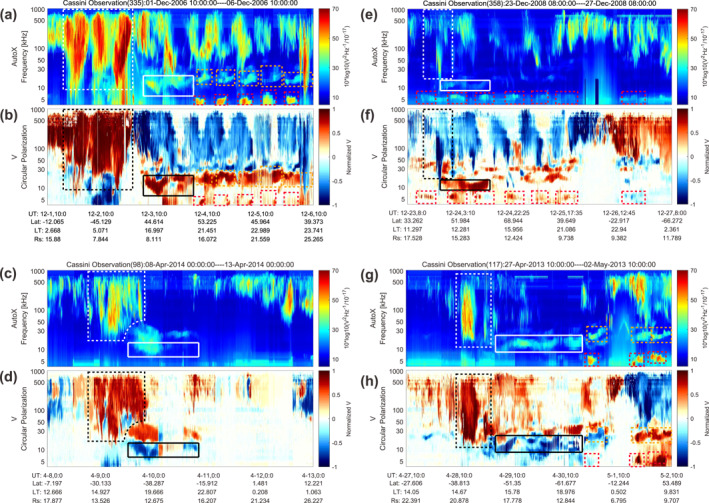
Four examples of the Saturn Anomalous Myriametric Radiation (SAM) signals. Panels (a–h) show four SAM cases with Panels (a, c, e, and g) showing the electric field spectrograms (measured by the Cassini Radio and Plasma Wave Science High Frequency Receiver (HFR)) and panels (b, d, f, and h) giving the circular polarization degree (obtained from the inversion method of Cecconi and Zarka (2005)). The SAM emissions are marked by the white and black solid boxes in the intensity and polarization panels (only the fundamental emissions are boxed), separately. The white and black dashed boxes indicate the Saturn Kilometric Radiation low frequency extension preceding the SAM. The red and orange dashed boxes indicate the 5 and 20 kHz NB, respectively. Cassini ephemeris data given at the bottom of each case are in the Saturn planetocentric coordinates.

Four examples of SAM are shown in Figure 1. The electric field spectrograms (in panels (a), (c), (e), and (g)) are from the Cassini Radio and Plasma Wave Science (RPWS) instrument and the values plotted in the figure are the auto‐correlations of the dipole X‐antenna signals (Gurnett et al., 2004). The circular polarization data shown in Figures 1b, 1d, 1f, and 1h are the Stokes V parameter (defined in the plane perpendicular to the wave normal, Kraus, 1966; Lamy et al., 2011; Fischer et al., 2009), obtained using the algorithm of Cecconi and Zarka (2005) by presetting the source at the center of Saturn. The R‐X mode emissions in the polarization data in Figure 1b show a circular polarization degree close to −1 (blue) in the northern hemisphere and 1 (red) in the southern hemisphere due to the variation of the angle between the wave vector and the background magnetic field (Lamy et al., 2011; Ye et al., 2009). The L‐O mode has opposite senses of polarization with respect to the wave vector in the northern (*V* = +1) and southern (*V* = −1) hemispheres.

**Figure 2:**
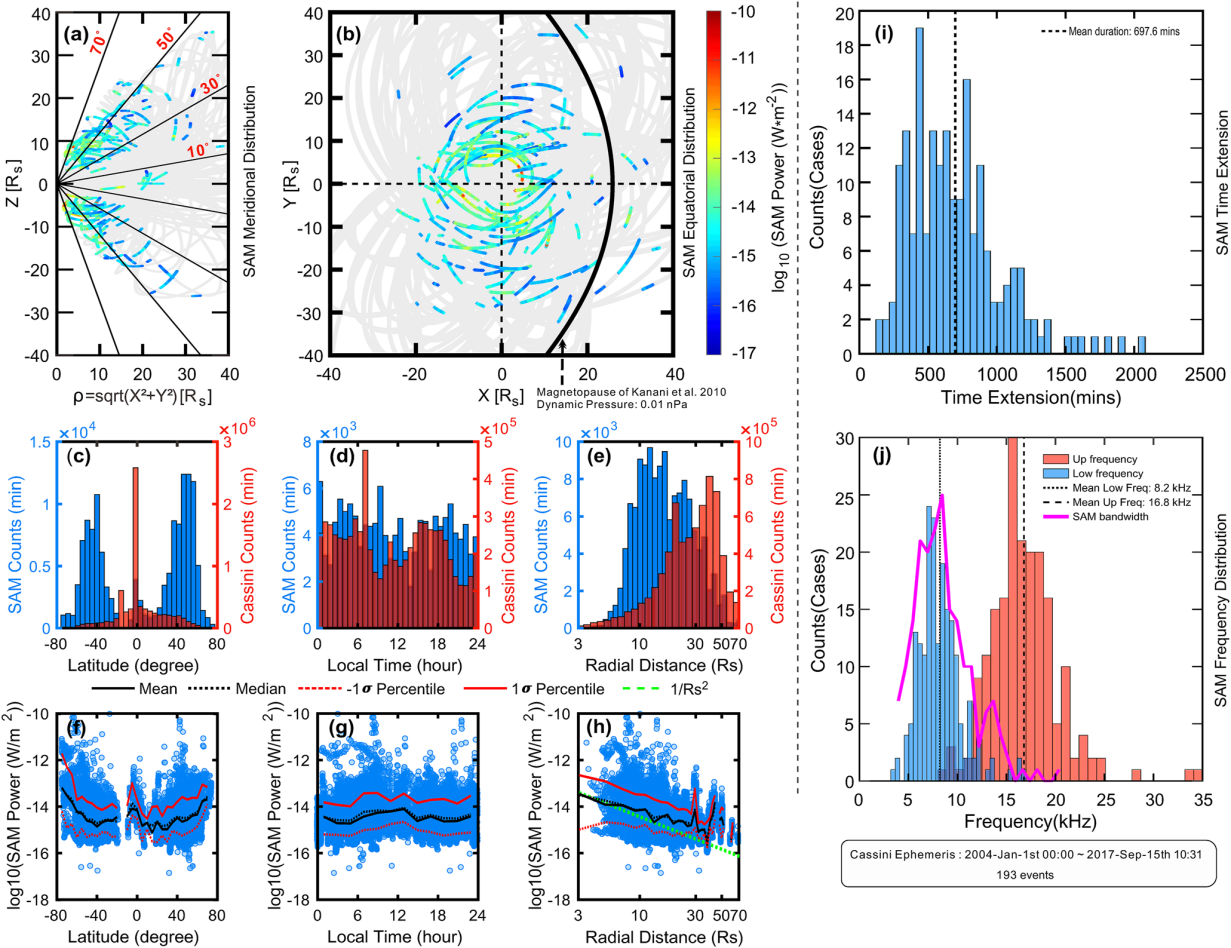
Characteristics of Saturn Anomalous Myriametric Radiation (SAM). Panels (a and b) give the spatial distribution of SAM in the meridional and equatorial plane with the power flux of SAM coded using the color bar on the right. The trajectory of Cassini during its Saturn orbital tour when SAM is not observed is marked in gray. The blue bins in Panels (c–e) show the histogram of SAM distribution in latitude, local time and radial distance. The overplotted red bins are the respective Cassini dwell times. Panels (f–h) give the power flux distribution of SAM in latitudes, local time and radial distance. The black and red lines (both solid and dotted) in panels (f–h) show the calculated power values (black: mean and median, red: percentile of ±1 standard deviation) to indicate the variation trends of the data. The green line in Panel (h) marks a 1/*r* ^2^ decreasing trend of the SAM power with the radial distance. Panels (i and j) give the time duration and frequency distribution of the SAM emissions. The pink line in panel (j) gives the bandwidth of SAM emissions.

The morphologies of the SAM (white solid boxes) in the spectrogram are different from the 5 kHz NB (red dashed boxes), the 20 kHz NB (orange dashed boxes) and the SKR (white dashed boxes in the spectrogram and black dashed boxes in the polarization plot). The SAM signals show variable frequency drifts (both downward and upward) with the lowest frequency extending down to 5 kHz (white box in panel (d)) and the highest frequency extending up to 20 kHz or even higher (panel (a)). The frequencies of SAM center around 13 kHz (detailed in Figure 2j). Compared to the 5 and 20 kHz NB, which usually show a stable frequency within a narrow band (Gurnett et al., 1981; Wang et al., 2010), SAM emissions sometimes cover broader frequency ranges (in panels (a) and (g) and also Figure 2j, often with more significant drifts (see details in Section 3). The frequency drifts in Figures 1a and 1c are roughly within the range 5 kHz/day to 12 kHz/day (see details in Section 3). In general, the duration of SAM is longer than typical 5 and 20 kHz NB emissions as shown in panels (a) and (g).

SAM occasionally appear with a weaker first harmonic as can be recognized more easily in the circular polarization data in panels (d) and (h). The circular polarization degree shown in panels (d) and (h) suggests that the fundamental SAM is L‐O mode and the harmonic is R‐X mode. As indicated by the white dashed boxes in Panels (a), (c), (e) and (g), SAM usually appears after an LFE of SKR. Therefore, we will investigate a possible connection between the SKR LFE and the SAM in Section 4.

## 3 Method to Identify SAM and the Characteristics of SAM

Based on the Cassini RPWS instrument data (Gurnett et al., 2004), we manually identified 193 SAM cases by using the data from 2004 DOY (day‐of‐year) 001 to 2017 DOY 258. First, the start time, end time, lower and upper frequency limits of the fundamental emission of each event are marked in the electric field spectrogram by hand as indicated by the white solid boxes in Figures 1a, 1c, 1e, and 1g. The accurate information of each SAM event is then obtained by setting a threshold on the circular polarization to |*V*| > 0.2 within each white box. The power flux of each SAM event is then calculated by using the spectral density in the corresponding frequency range, the detailed calculation procedure is the same as given in Zarka et al. (2004) and Ye et al. (2010). The final list of all identified events is given in the Supporting Information S1.

The criteria which enabled us to distinguish the SAM from the neighboring SKR and from NB emissions in the overlapping frequency range of 3–35 kHz are as follows:

1. The SAM emissions' frequency varies between 3 and 35 kHz (typically 3–8 kHz for 5 kHz NB and 12–30 kHz for 20 kHz NB. Wu et al., 2021), and the center frequency of the fundamental emissions is near 10–15 kHz (typically around 5 and 20 kHz for the NB emissions Ye et al., 2009).
2. The SAM emissions' frequency shows larger frequency drifts (roughly several kilohertz/day, a simple eye‐estimation of the frequency drifts on the cases in Figures 1a and 1c are approximately from 5 to 12 kHz/day) than the NB (typically upward frequency drift with 200 ∼ 400 Hz/day for 5 and 20 kHz NB. Wang et al., 2010), and the upward drift and downward drift can be observed both in a single event.
3. The bandwidth of the fundamental SAM emissions is broader than the NB (2 ∼ 3 kHz for 5 kHz NB and within 10 for 20 kHz NB. Wang et al., 2010).

We note here that each single SAM case in the list should meet at least the first two requirements above to be identified (for some cases the emissions are not that broadbanded but do satisfy the first two criteria). Finally, 193 cases of the SAM were identified, and the SAM events are very rare when compared to more than 2,000 cases for 5 kHz NB and more than 1,000 cases of the 20 kHz NB during the 13 years of data (Wu et al., 2021). Because of the rarity of this emission and the similarity between SAM and NB emissions, we note here that identifying the SAM in the spectrogram could be pretty subjective in some cases. However, the criteria mentioned above and the different spectral morphology of SAM and NB as displayed in Figure 1 warrant correct identification in the vast majority of the cases. The rarity of SAM observations in the complete Cassini RPWS data set may be due to the following reasons:

1. The SAM intensities are often weak. The power spectral density of a considerable part of the SAM cases is close to 10^−15^ ∼ 10^−16^ V^2^ Hz^−1^, which is close to the background noise level (Gurnett et al., 2004). Many cases in our event list were identified by looking at the polarization data since the signal in the power spectrogram is too weak.
2. SAMs are preferably observed at higher latitudes as shown in Figures 2a and 2c. No SAM emission was observed in 2015 due to the low inclination of Cassini's orbit during that year. Only 8 SAM cases are observed in the equatorial plane as shown in panel (a), which could be due to the reflection at the magnetosheath when the electron density in the magnetosheath is high enough, similar to the 5 kHz NB (Wu et al., 2022). This suggests that SAM emissions rarely undergo reflections at the magnetosheath as their frequency is mostly above the plasma frequency in the sheath (Wu et al., 2022).
3. These SAM emissions are sporadic events that may be attributed to some special magnetospheric conditions which are not fully understood at this time (as discussed in detailed in Section 4).

We note here that all the characteristics described below are for the fundamental emissions of SAM. The distributions of the 193 SAM events in the meridional and equatorial planes are given in Figures 2a and 2b. The SAM emissions are mainly observed at high latitudes with latitudes above 20°, similar to the 20 kHz NB (Wang et al., 2010; Wu et al., 2021).

The detailed occurrence and power flux distributions are given in panels (c)–(e) and (f)–(h) of Figure 2. The blue and red bins in panels (c)–(e) are the SAM counts and all Cassini orbital positions, respectively. The majority of the SAM emissions is observed between 20° and 70° southern and northern latitudes. Local time dependence is not observed. Most SAMs are observed inside the magnetopause as marked by the black solid line in Panel (b) (Kanani et al., 2010).

After integration over the SAM spectral frequency range for each of the 193 events, a total of 134,608 power flux values is obtained. These are plotted as blue circles with respect to latitude, local time and radial distance in Figures 2f–2h. The power of SAM decreases from high latitudes to low latitudes as indicated by the overplotted profiles in panel (f) (black solid and dotted for mean and median, respectively, red for percentile of 1 standard deviation, these profiles are derived from the logarithm scale (=mean of exponents)). The integration carried out within a box is sometimes polluted by a low frequency extension of SKR, especially near the equatorial plane, where it is difficult to separate SAM from SKR according to polarization (SKR emitted from the opposite hemisphere has the same sign of Stokes‐V as the SAM emissions). Therefore, the small peak of the calculated average intensity near 0° in latitude in panel (f) may be an overestimate. No significant variation with local time is visible in panel (g). The power of SAM ranging from 10^−16^ W/m^2^ to 10^−10^ W/m^2^  shows a 1/*r* ^2^ dependence on the radial distance (indicated by the green dashed line and also the calculated mean/median lines in panel (h)), which is consistent with a point source close to Saturn as the signal gradually weakens as it propagates away from Saturn. We also checked the longitudinal distribution of the SAM emissions, which shows quite uniform distribution (not shown). SAM events usually last for several hours, that is, they cover a broad longitude range due to Saturn's rapid rotation. It is thus not surprising that SAM visibility smears over all longitudes almost uniformly.

The distribution of time durations of SAM in Panel (i) peaks around 500 min with a mean duration of 697.6 min. Given Saturn's rotation period of roughly 10.6 hr (636 min), the average SAM duration is just a bit longer than one Saturn rotation, and in some cases, SAM can last for 2–3 Saturn rotations (e.g., case in Figure 1c and Figure S159 in the Supporting Information S1). The frequency distribution shown in Figure 2j suggests that SAMs' frequency ranges from 3 to 30 kHz and centers near 13 kHz. The pink line in Panel (j) gives the approximate bandwidths of the SAM emissions. Most of the SAM emissions have bandwidths larger than 5 kHz.

## 4 Relations Between SAM, LFE, Reconnection, and the Compression of the Magnetosphere

When identifying SAM events from the data, we notice that the SAM emissions are often observed after an SKR LFE (defined in the next paragraph). The SKR LFE is deemed as a good proxy for strong magnetospheric dynamics, which can include tail reconnection (e.g., Bunce et al., 2005; Jackman et al., 2009) that may be induced by solar wind compression (Reed et al., 2018).

To further explore this feature, we adopted an interactive polygon labeling method (Louis et al., 2022) to label the SKR LFE. The specific criteria of labeling SKR LFE are similar to the criteria of Reed et al. (2018) and are listed below:

1. The LFE begins when the main SKR band extends below 100 kHz and ends when it has returned to above 100 kHz.
2. The SKR has a minimum frequency of 40 kHz or less for it to be an LFE.
3. The SKR LFE also is a continuous extension, that is, there should be no frequency gap.

We identified 381 LFEs during the time interval between 24 hr before the SAM start time and the SAM end time. Finally, by confining the time to within 10.6 hr (one Saturn rotation) around the SAM start time (|SAM start time ± LFE start or end time|<10.6 hr), 136 of 193 SAM events (ratio: 136/193∼ = 0.7) are labeled as an event that is accompanied by LFE. Indeed, a total of 128 SAM events are observed to be preceded by the LFE emissions among these 136 cases. We perform a significance Z‐test on the proportion of the SAM events accompanied by LFE (136/193) to test the relation between SAM and LFE. The SAM events are divided into two categories: SAMs that occurred with LFE, and SAMs that occurred without LFE. We could simply adopt a null hypothesis that the two categories are random processes with occurrence of the two groups *q*1 = *q*2 = 50%. The *Z* value could be calculated through (Fleiss, 2003; Li & Yao, 2020): $Z=\left(p_{1}-p_{2}\right)-\left(q_{1}-q_{2}\right)/\sqrt{\frac{q_{1}\left(1-q_{1}\right)}{n_{1}}+\frac{q_{2}\left(1-q_{2}\right)}{n_{2}}}$ (where the *p*_1_ = 0.704 and *p*_2_ = 0.295 are the proportions of category 1 and category 2. *q*_1_ and *q*_2_ are the probabilities that are assumed to be equal (*q*_1_ = *q*_2_ = 50%). *n*_1_ = 136 and *n*_2_ = 57 are the sample numbers of the two categories). The calculated *Z* value is 5.188, exceeding the critical *Z* test value (1.645) at a 95% confidence level, which implies a rather high proportion of SAM events associated with an LFE.

**Figure 3:**
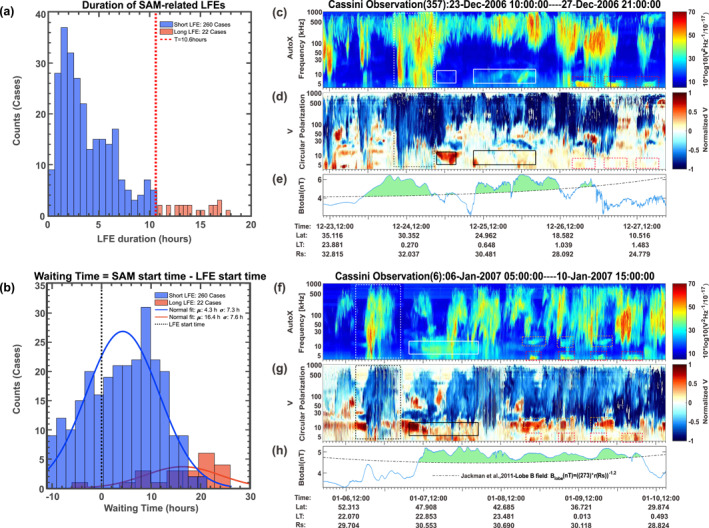
Saturn Anomalous Myriametric Radiation‐low frequency extension (SAM‐LFE) relation. Panel (a), the time duration of the SAM‐related LFEs, separated to long/short LFEs using a threshold 10.6 hr. Panel (b), the waiting time histogram between the start time of SAM emissions and the start time of SAM‐related LFEs. Panels (c and d) and (f and g) are in the same format as Figure 1. Panels (e and h) are the total magnetic field measured by the Cassini magnetometer instrument (Dougherty et al., 2004). The black dashed line marks the fitted averaged lobe field derived by Jackman et al. (2011 ). The green shaded area marks the region with magnetic field surpassing the averaged lobe field.

The statistical study of LFEs (Reed et al., 2018) based on data from 2006 suggests that short LFEs are a good proxy for tail reconnection and longer LFEs can be associated with the solar wind dynamics. We check the durations of the SAM‐related LFEs as shown in Figure 3a. Note here for one SAM event, there could be several LFEs within the time range: SAM *start* *time* ±10.6 hr. These events are all plotted in Figure 3a and 3b. The 10.6 hr (i.e., 1 Saturn rotation) threshold is used to separate the short and long LFEs, which is reasonable as suggested by the former studies (Bunce et al., 2005; Reed et al., 2018 and the references therein). Roughly 92% of LFEs (260/282, as shown in Figure 3a) have a duration shorter than 10.6 hr, suggesting these LFEs are possibly related to the tail reconnection (indicated by blue bins in Figure 3a). We stress, however, that tail reconnection may be driven internally (by the Vasyliunas cycle (Vasyliunas, 1983)) or externally (by the Dungey cycle (Dungey, 1961)). Seeing more short LFEs does not necessarily favor internal drivers over external ones (e.g., solar wind compression). Moreover, assessment of the duration of the LFEs is also influenced by the spacecraft location, because the observer local time relative to the beaming from the hollow cone of emission has a significant impact on the received radio power. It is possible that “short” LFEs are merely truncated due to the spacecraft moving into/out of the primary radio viewing region before the entire episode can be sampled. Thus it is likely that the duration of the shorter LFEs could be taken as a slight underestimate in some cases. There are 22 SAM events that are accompanied by these 22 long LFEs and 114 SAM events are accompanied by the rest 262 short LFEs. Indeed, the fact that 282 LFEs are observed near the 136 SAM events suggests that the SAM emissions are generated under active magnetospheric conditions.

The time delays between the labeled 136 SAM events and the labeled SAM‐related LFEs (in total 282 events) are shown in Figure 3b. The approximate time delay is derived by subtracting the start time of SAM emission from the start time of SKR LFEs. The mean delay time between short LFE and SAM is about 4.3 hr (with median value 4.89 hr) as marked by the blue line in Figure 3b. The waiting time between the long LFEs and the SAM emission is approximately 16.4 hr (estimated from 22 cases, as marked by the red line in Figure 3b). For these 22 long LFEs, it is their stop‐time which gets closer than 10.6 hr to the SAM start‐time. Therefore, the long LFE start‐time can be much further away from the SAM start‐time, explaining the very long delay times. Furthermore, it is possible that SAM would be there much closer to the start‐time of a long LFE, but it is just not visible in the spectrum because the long LFE is superposing the weaker SAM emission entirely. These results suggest that the occurrence of SAM emissions is associated with the magnetospheric conditions that lead to SKR LFE. Note here that the derived waiting time is only approximate due to the uncertainty of the start time of the LFEs, which strongly relies on the spacecraft viewing geometry.

Even though the majority of the SAM emissions are observed after SKR LFE, according to previous studies (Jackman et al., 2009; Reed et al., 2018), most of the LFEs appeared without being followed by SAM. This can be attributed to several reasons, like tighter visibility constraints for SAM in comparison to SKR (especially with latitude and intensity), or additional requirements for the excitation of SAM on top of those for SKR LFEs. We show possible evidence of the connection between SAM and the solar wind compressions in Figures 3c, 3e, 3f, and 3h. The magnetic field data in Panels (e) and (h) are from the Cassini‐MAG instrument (Dougherty et al., 2004). Cassini was in the northern tail lobe region during the two time intervals. Clear magnetotail compressions are observed (indicated by the green shaded region in Panels (e) and (h)) with an increase in the total magnetic field strength above what is typically observed in the lobe at that radial distance (Jackman & Arridge, 2011). The compression lasts for almost 3 days for the second case in Panel (h), until 2007 DOY 010 10:00 UT when the lobe magnetic field gradually decreased. The SAM emission in Panel (f) is almost synchronized with the start of the compression, which could imply that the excitation of the SAM emission is due to the compression of the magnetosphere.

Here we highlight the close relationship between SAM emissions and SKR LFEs that the LFE is always seen when there is a SAM. The examples in Figure 3 suggest that the SAM emission could be triggered by magnetospheric compression in favor of tail reconnection, which would also implied that the LFE seen simultaneously were also triggered by the magnetospheric compression.

## 5 Discussion and Summary

As reported in earlier studies (Bradley et al., 2020; Bunce et al., 2005; Cowley et al., 2005; Guo et al., 2018; Jackman et al., 2009; Mitchell et al., 2015; Reed et al., 2018; Thomsen et al., 2019), compressions of the Saturnian magnetosphere caused by interplanetary corotating interaction regions or Coronal Mass Ejections can trigger a series of magnetospheric responses including motion of the magnetospheric boundaries, dayside and nightside magnetic reconnection, magnetotail current sheet collapse, plasmoid release, hot plasma injection and the intensification of SKR. In the absence of an upstream probe at Saturn, studies have used magnetospheric boundary locations or propagated solar wind models (e.g., Tao et al., 2005; Zieger and Hansen., 2008) to infer the state of the upstream medium. There is also significant reason to use the radio emissions as a remote proxy for upstream conditions if the radio emissions can be cataloged and understood appropriately.

The SKR signals are usually used as an important tool to indicate large‐scale disturbances such as auroral intensification, tail reconnection, and intensified field‐aligned current (Bradley et al., 2020; Palmaerts et al., 2018). The SAM emissions could also be used as a remote indicator of the compression or the magnetic reconnections once the detailed relations between these phenomena are clarified in further studies. At present, the generation mechanism of SAM is uncertain due to the lack of observations. The SAM emissions show very similar features to the 20 kHz NB with respect to frequency range, polarization characteristics and the frequent occurrence of harmonic emissions. One may suspect that the SAM emissions are just a kind of anomalous 20 kHz NB but generated under special magnetospheric conditions. However, in some cases, both the SAM emissions and the 20 kHz NB can exist simultaneously, as shown in some cases in the Supporting Information S1 (e.g., Figures S24, S28, S37, S38, and S43 in Supporting Information S1). More detailed studies, for example, a direction‐finding case study may answer this.

In this study, we proposed a new radio component called Saturn Anomalous Myriametric radiation and investigated the basic characteristics of SAM and the possible connection between SAM and the other magnetospheric processes. Our main findings are as follows:

1. The SAM emissions show variable frequency drifts and obvious differences in spectral morphology to narrowband emissions, which are usually observed in the adjacent frequency range. Therefore, these emissions are distinguished in this study and named SAM.
2. The SAM emissions are L‐O mode emissions usually occurring at around 13 kHz, and at high latitudes and sometimes accompanied by the R‐X mode first harmonic.
3. The SAM emissions are often observed following an LFE of SKR and are possibly associated with the solar wind compression of the magnetosphere.

## Supporting information

Supporting Information S1

## Data Availability

The Cassini Radio and Plasma Wave Science data used in this work were downloaded from the LESIA/Kronos collection with n2 level data (Cecconi et al., 2017a) and n3d data (Cecconi et al., 2017b, goniopolarimetric data obtained using the method Cecconi & Zarka. 2005). The Cassini MAG data were downloaded from the Planetary Data System at (MAG: https://doi.org/10.17189/1519602). The SAM catalog is also available from the MASER service via a doi: https://doi.org/10.25935/8may-4320.
