# Supplementary material for: Saturn Anomalous Myriametric Radiation, a New Type of Saturn Radio Emission Revealed by Cassini
Source: Geophys Res Lett. 2022 Aug 19;49(16):e2022GL099237. doi: 10.1029/2022GL099237 (PMC9541930; doi:10.1029/2022GL099237)
Supplement: Supplementary file 1 — Supporting Information S1 [file GRL-49-e2022GL099237-s001.docx]

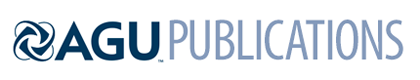


*Geophysical Research Letters*

Supporting Information for

**Saturn Anomalous Myriametric Radiation, A New Type of Saturn Radio Emission Revealed by Cassini**

S.Y. Wu^1,2^, S.Y. Ye^1^, G. Fischer^3^, U. Taubenschuss^4^, C.M. Jackman^5^, E. O’Dwyer^5^, W.S. Kurth^6^, S. Yao^7^, Z.H. Yao^8^, J.D. Menietti^6^, Y. Xu^8^, M.Y. Long^9^, and B. Cecconi^2^

^1^Department of Earth and Space Sciences, Southern University of Science and Technology, Shenzhen, Guangdong, People’s Republic of China

^2^LESIA, Observatoire de Paris, Université PSL, CNRS, Sorbonne Université, Université de Paris, Meudon, Paris, France

^3^Space Research Institute, Austrian Academy of Sciences, Graz, Austria

^4^Department of Space Physics, Institute of Atmospheric Physics of the Czech Academy of Sciences, Prague, Czechia

^5^School of Cosmic Physics, DIAS Dunsink Observatory, Dublin Institute for Advanced Studies, Dublin, Ireland

^6^Department of Physics and Astronomy, University of Iowa, Iowa City, IA, USA

^7^School of Geophysics and Information Technology, China University of Geosciences (Beijing), Beijing, People’s Republic of China

^8^Key Laboratory of Earth and Planetary Physics, Institute of Geology and Geophysics, Chinese Academy of Sciences, Beijing, People’s Republic of China

^9^Department of Space Physics, School of Electronic Information, Wuhan University, Wuhan, People’s Republic of China

**Contents of this file**

Figures S1 - S193 for all SAM cases

Table S1 of 193 SAM cases

**Introduction**

The supplementary materials include all the Figures of SAM emission cases identified that are given below. Each figure includes the wave electric field spectrogram, circular polarization degree, linear polarization degree and total polarization degree data. The horizontal axes provide the location of Cassini by using a boundary crossing list created from the magnetic field data (Jackman et al., 2019), one can find the detail descriptions of the location of Cassini in the bottom panel of Figure 1 in Wu et al., (2022).

Table S1, the event list is given in the end of this file, the columns “low_freq” and ‘up_freq’ give the manually marked lower and upper frequency of the SAM emissions. The column “LFE” mark the SAM emissions that whether it is accompanied by a SKR LFE by using the criteria described in the manuscript.


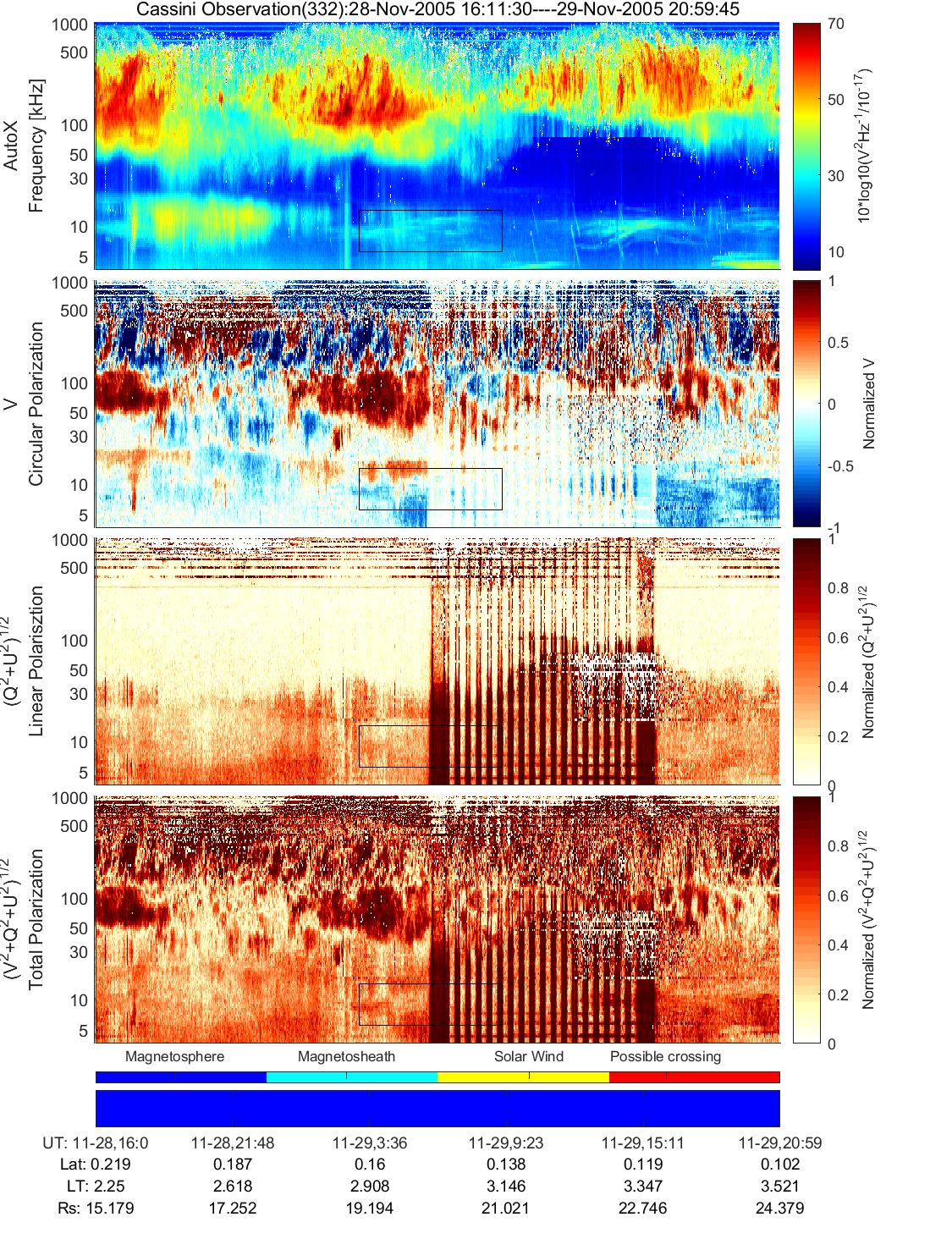


Figure S1, Case 1 of the SAM emission in Table S1. Panels from up to bottom: Wave electric field spectrogram; circular polarization degree; linear polarization degree; total polarization degree; and the location of Cassini: blue means Cassini was in the magnetosphere, cyan, yellow, and red correspond to magnetosheath, solar wind and possible crossing due to the missing of data, detailed in Wu et al., (2022).


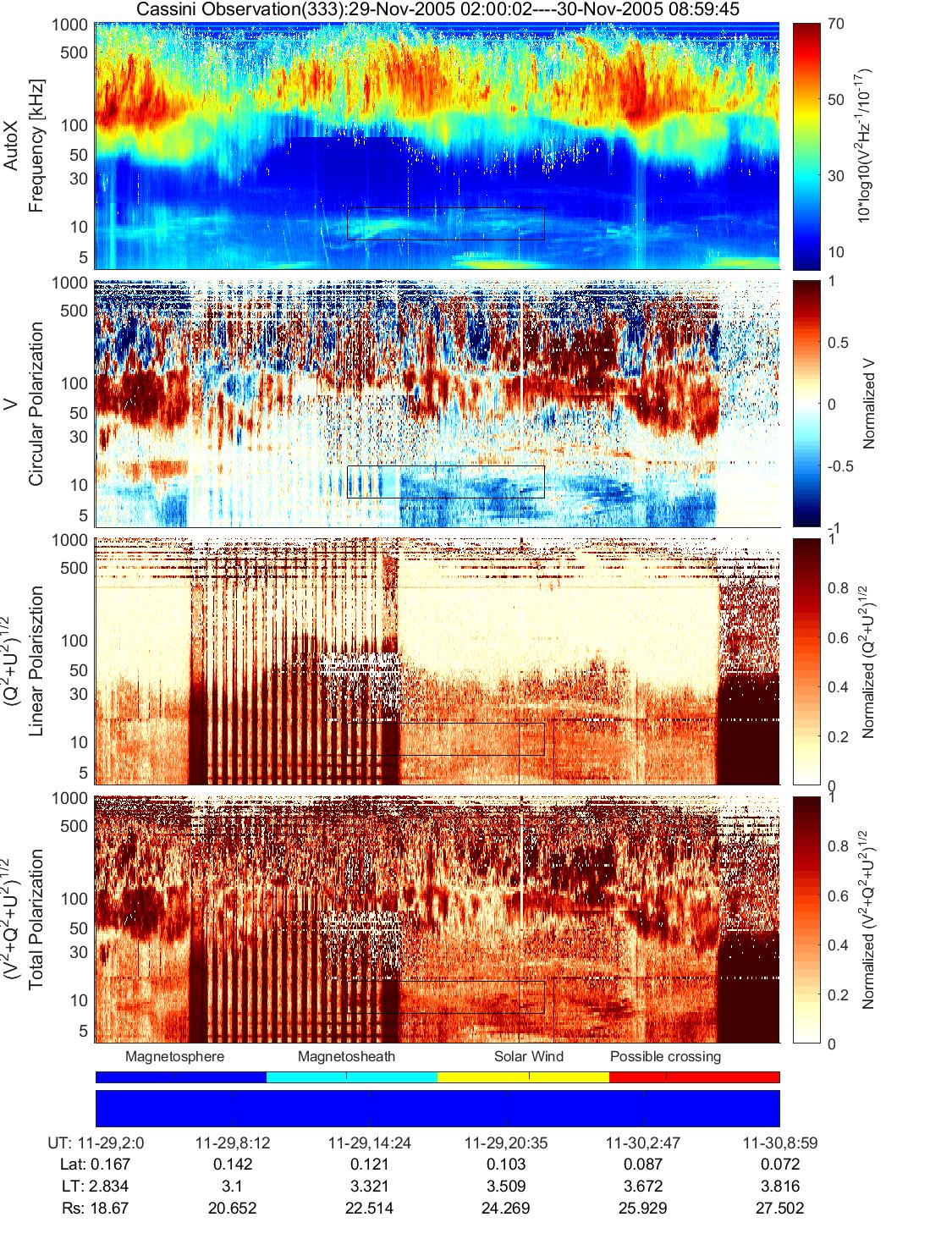


Figure S2, Case 2 of the SAM emission in Table S1. Same format as Figure S1.


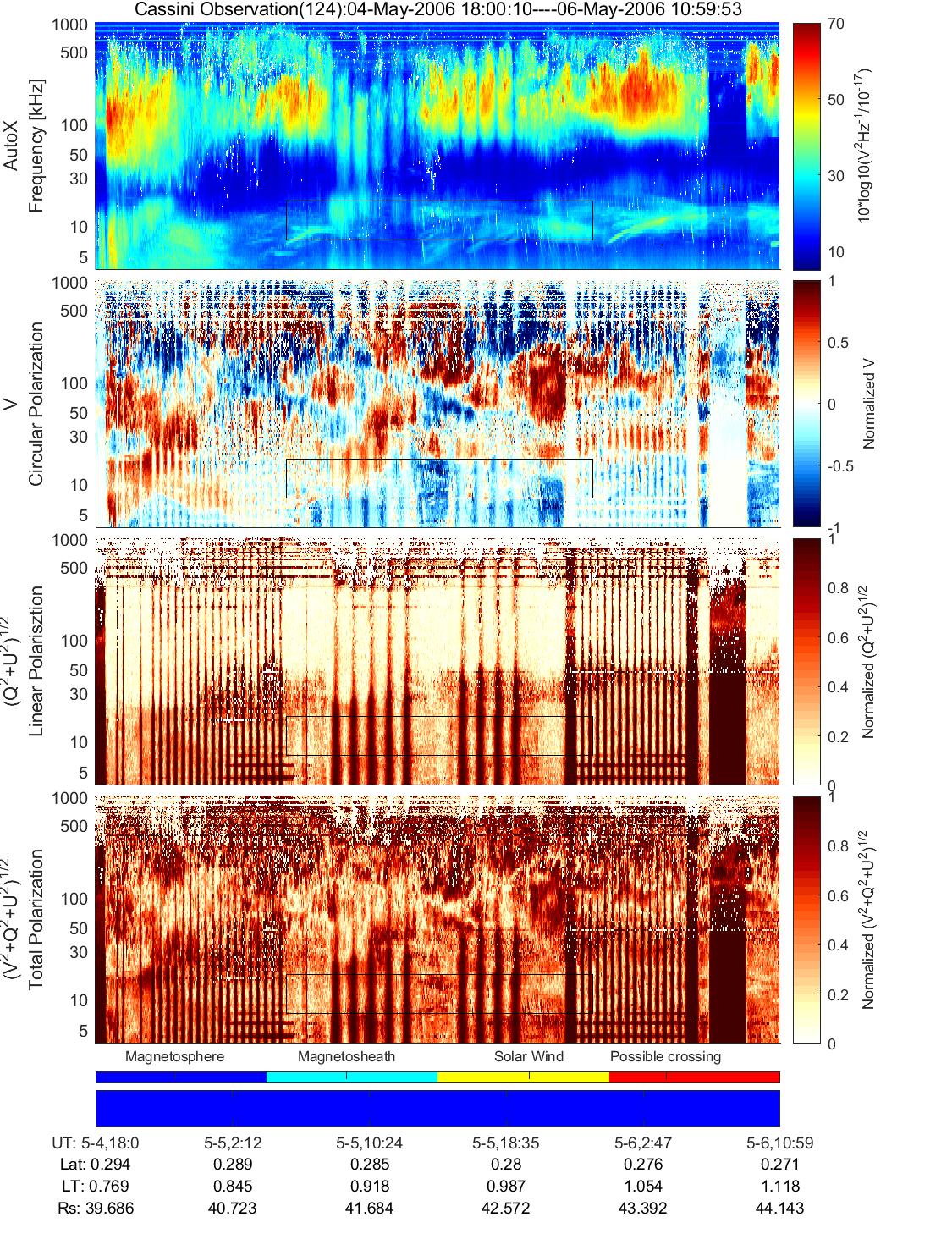


Figure S3, Case 3 of the SAM emission in Table S1. Same format as Figure S1.


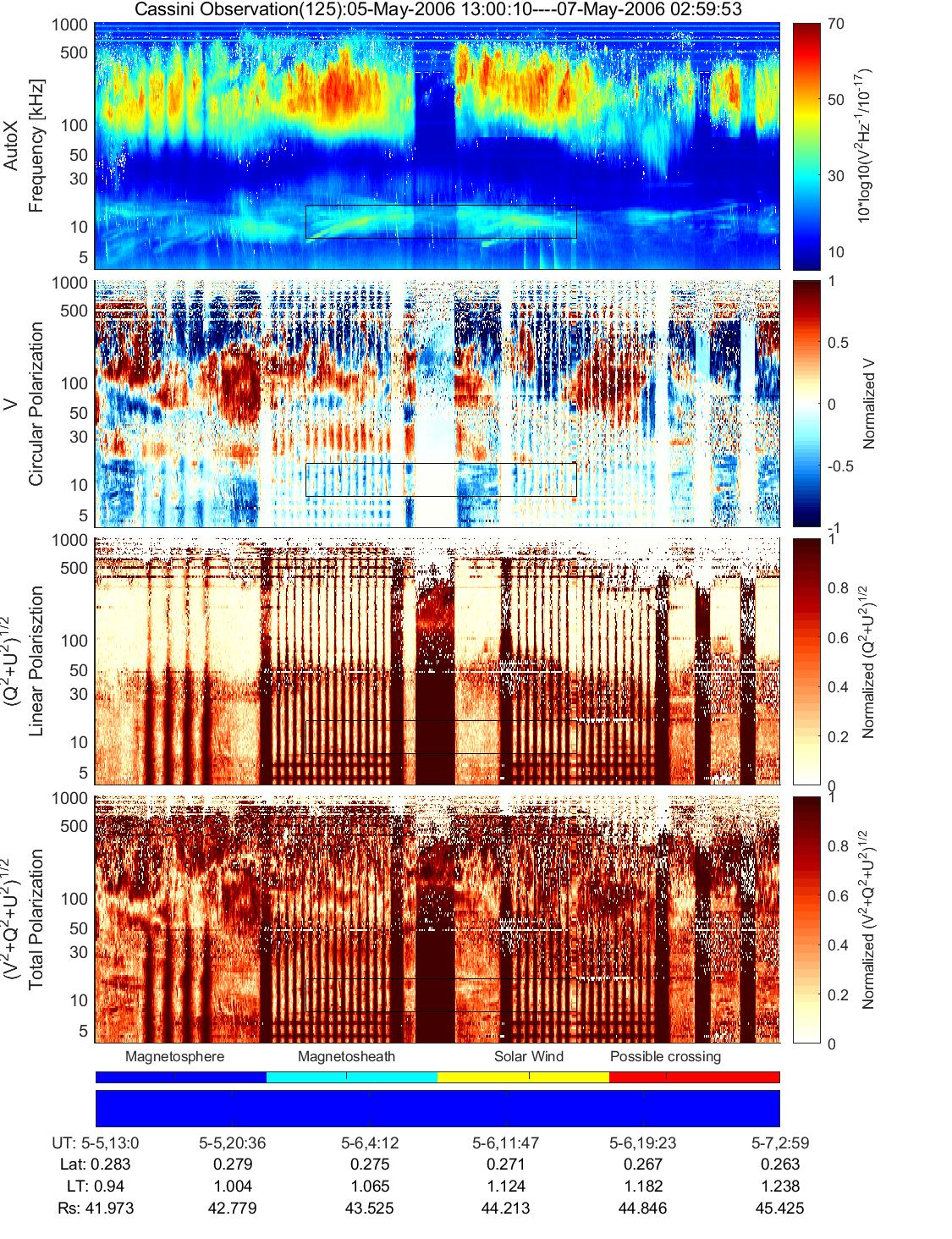


Figure S4, Case 4 of the SAM emission in Table S1. Same format as Figure S1.


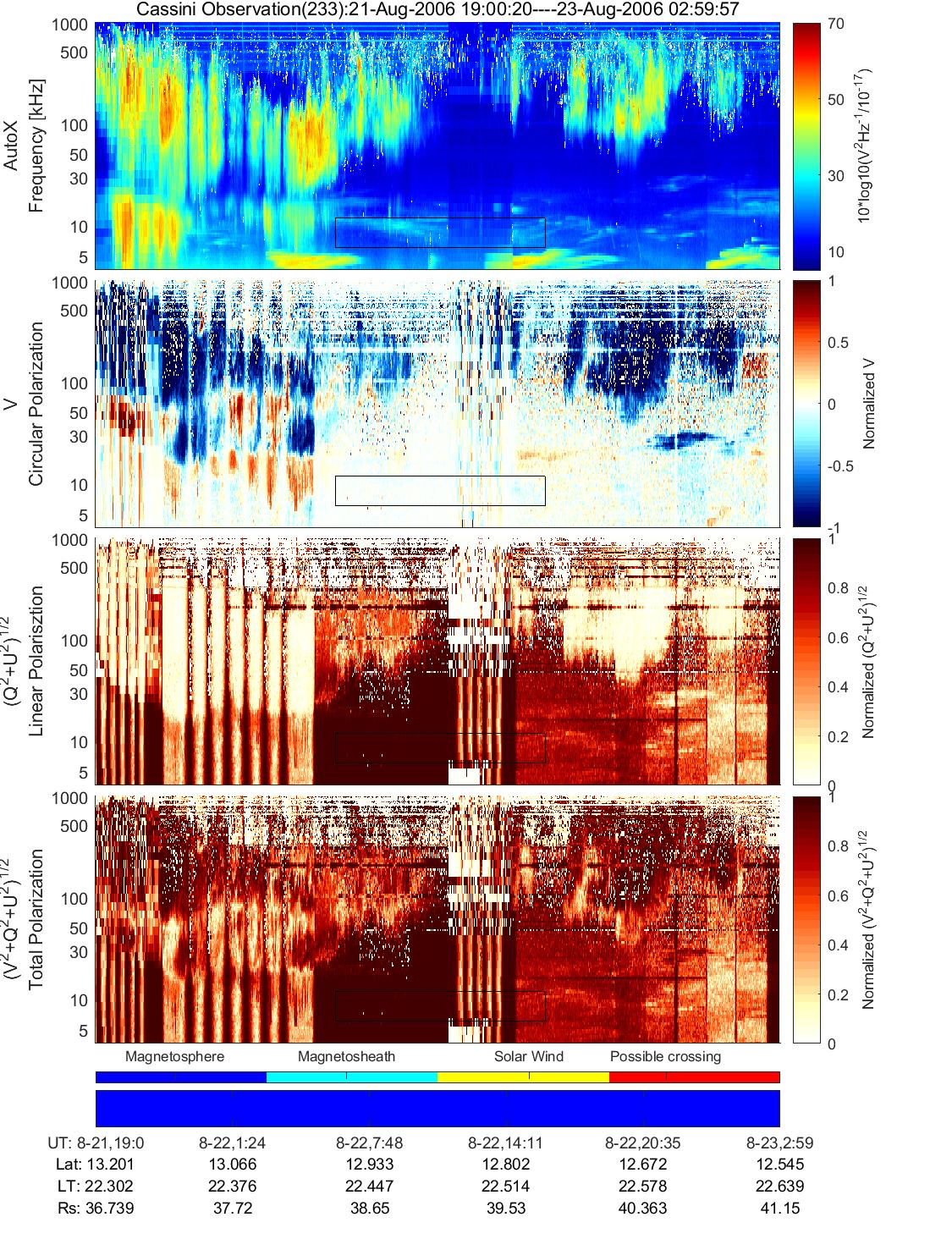


Figure S5, Case 5 of the SAM emission in Table S1. Same format as Figure S1.


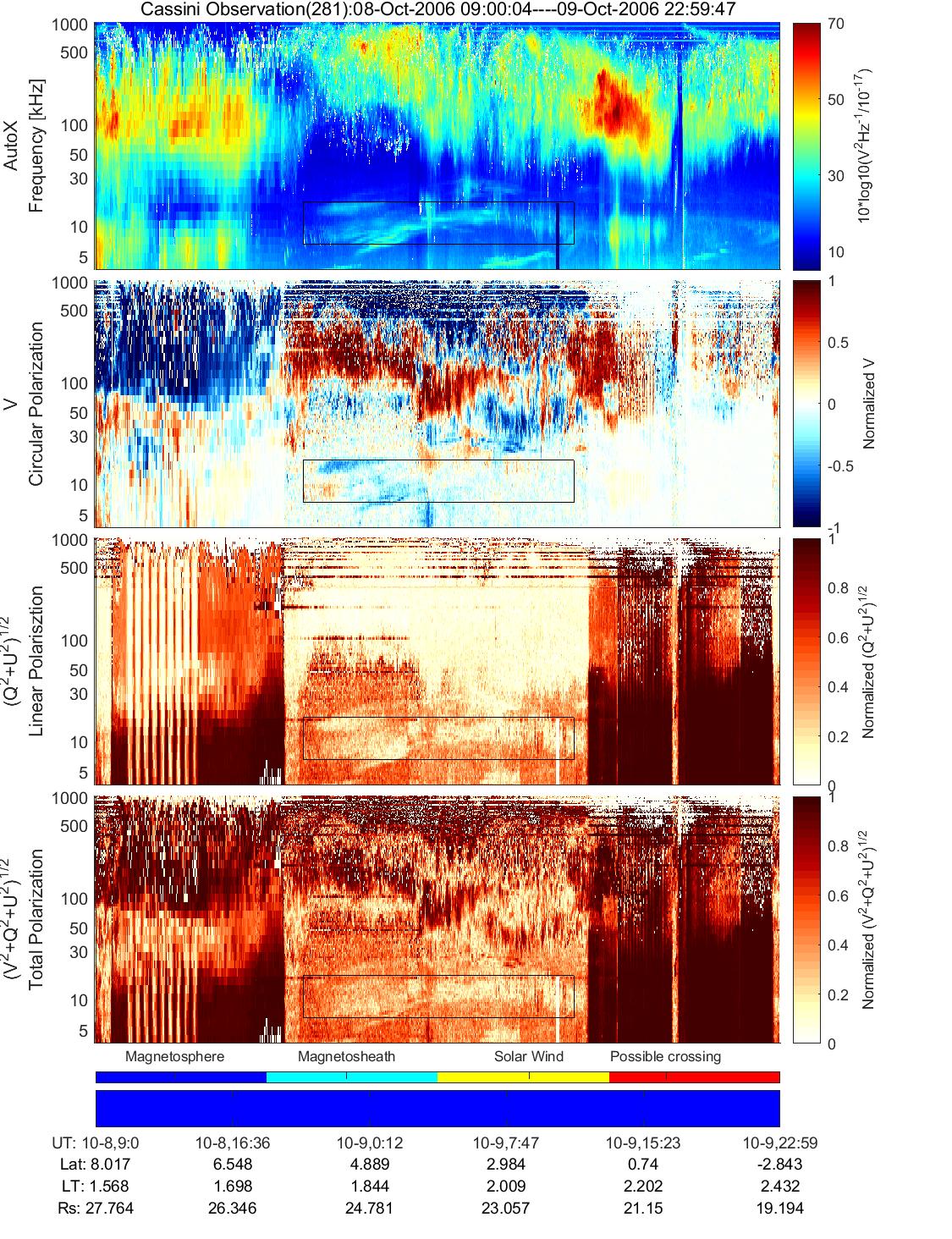


Figure S6, Case 6 of the SAM emission in Table S1. Same format as Figure S1.


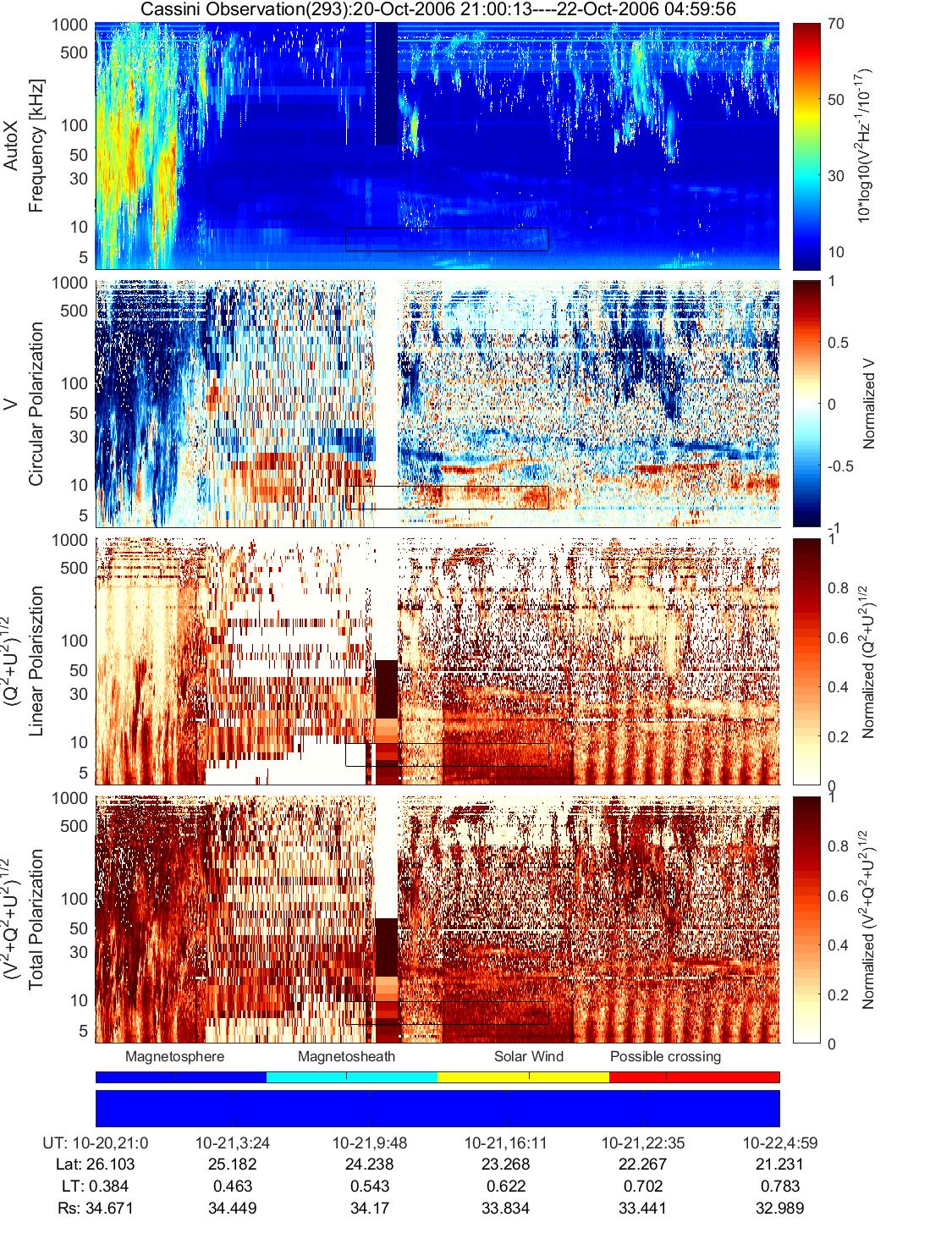


Figure S7, Case 7 of the SAM emission in Table S1. Same format as Figure S1.


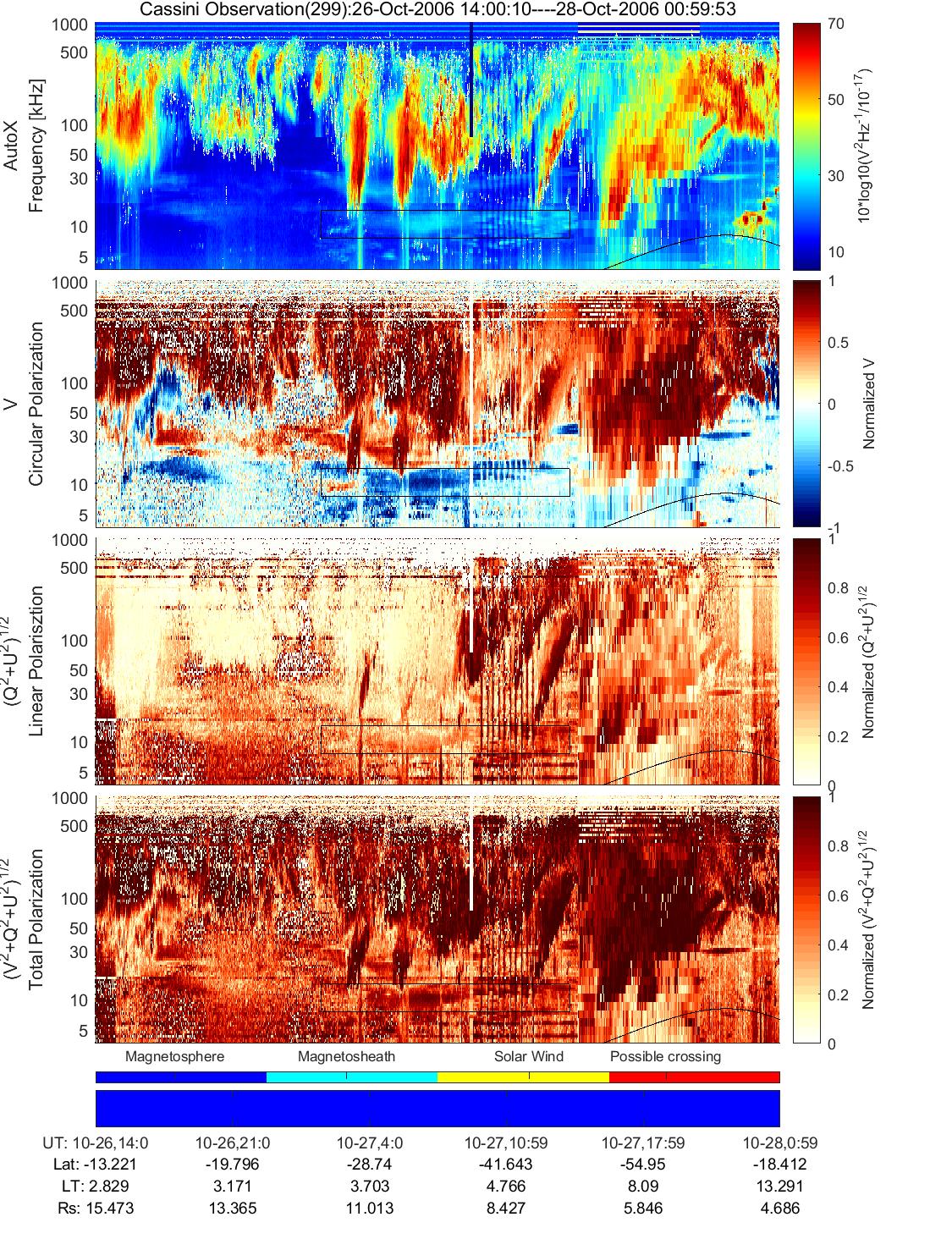


Figure S8, Case 8 of the SAM emission in Table S1. Same format as Figure S1.


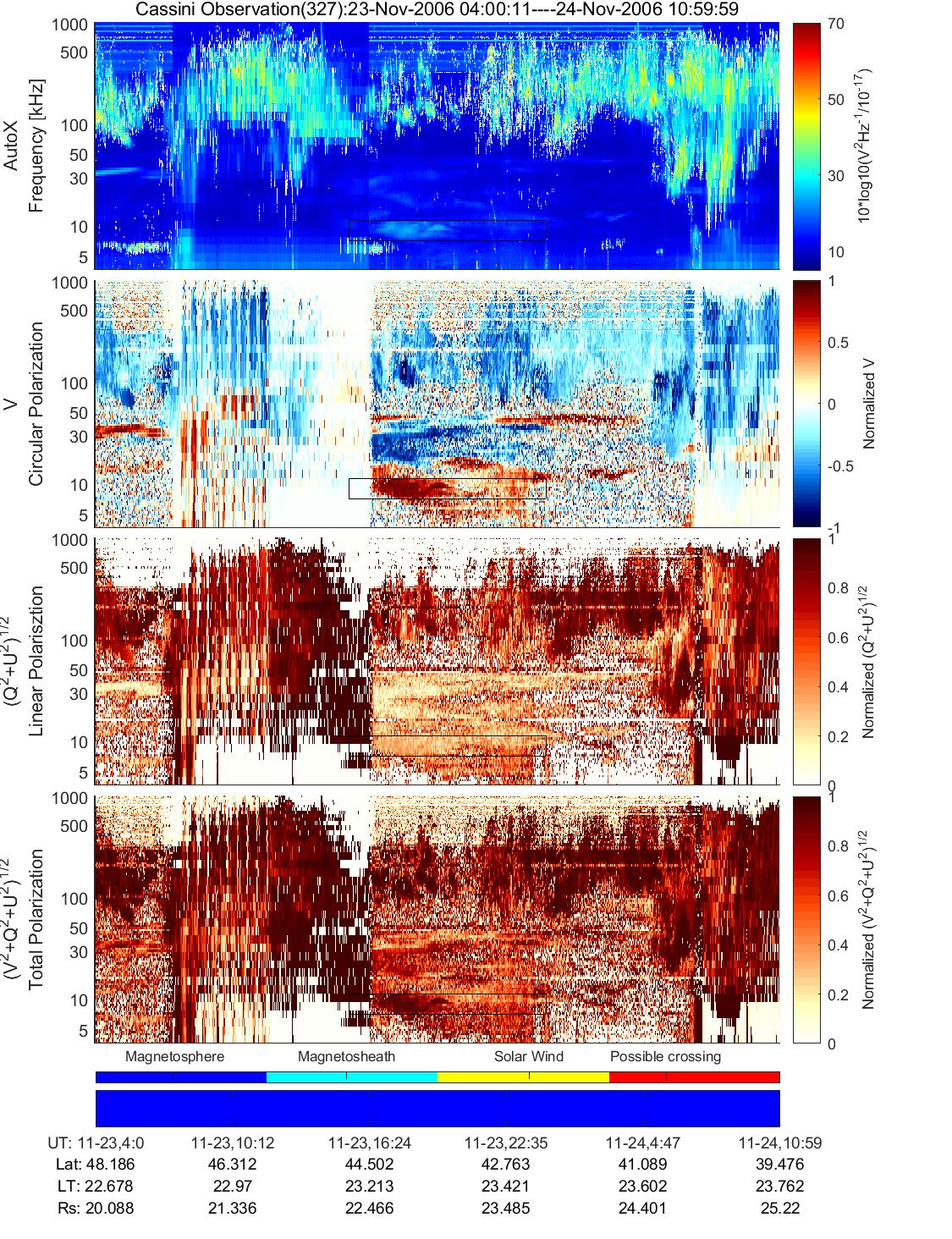


Figure S9, Case 9 of the SAM emission in Table S1. Same format as Figure S1.


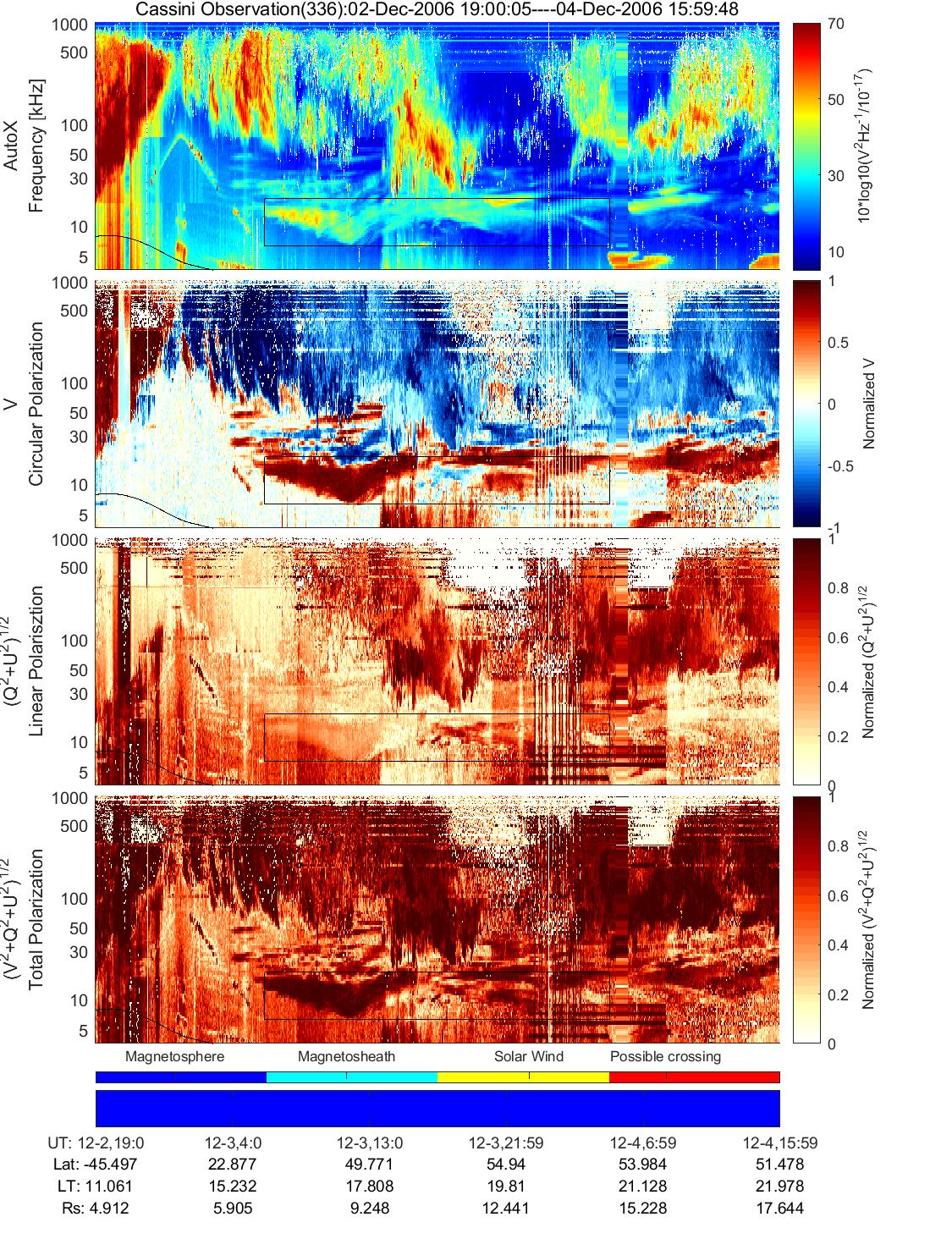


Figure S10, Case 10 of the SAM emission in Table S1. Same format as Figure S1.


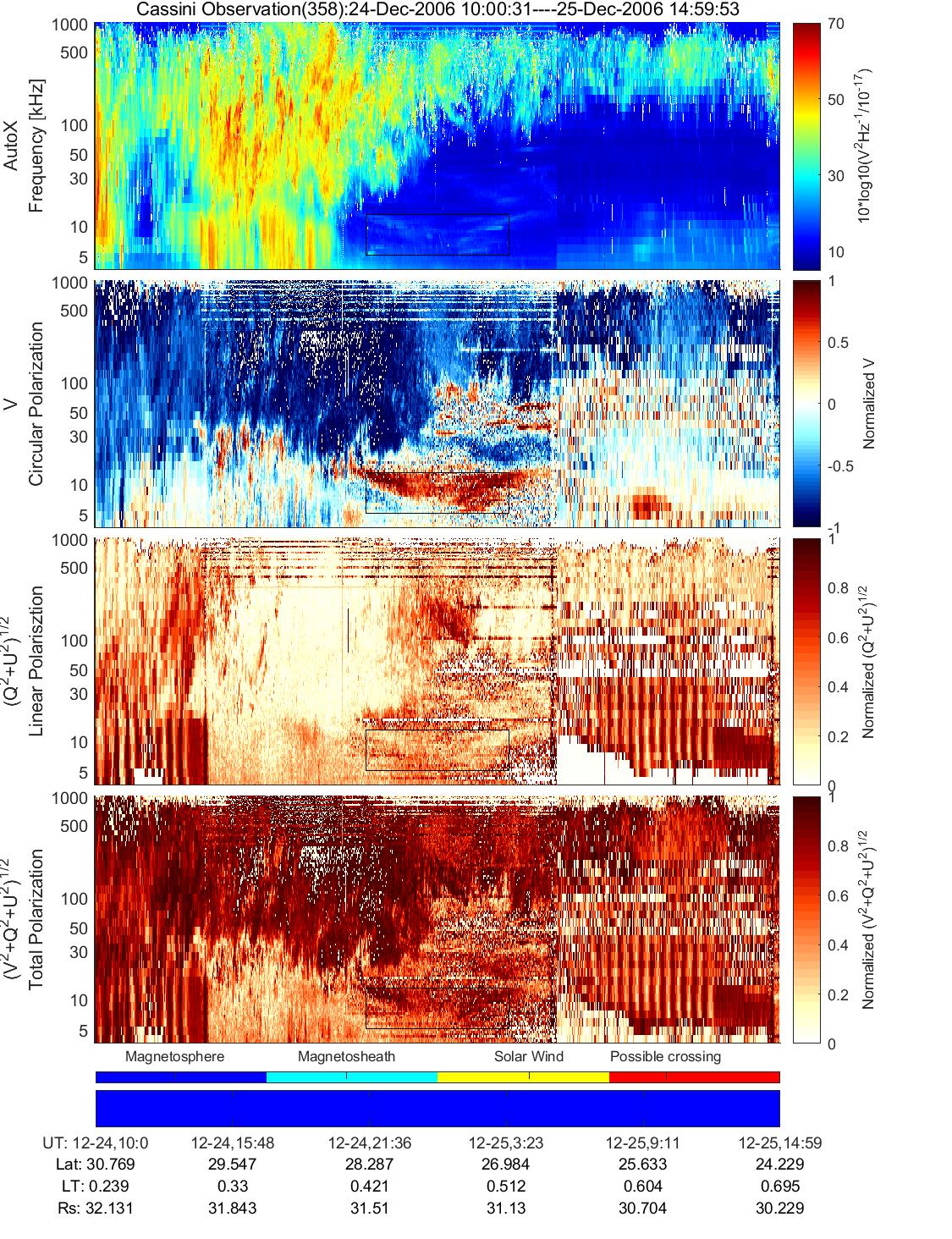


Figure S11, Case 11 of the SAM emission in Table S1. Same format as Figure S1.


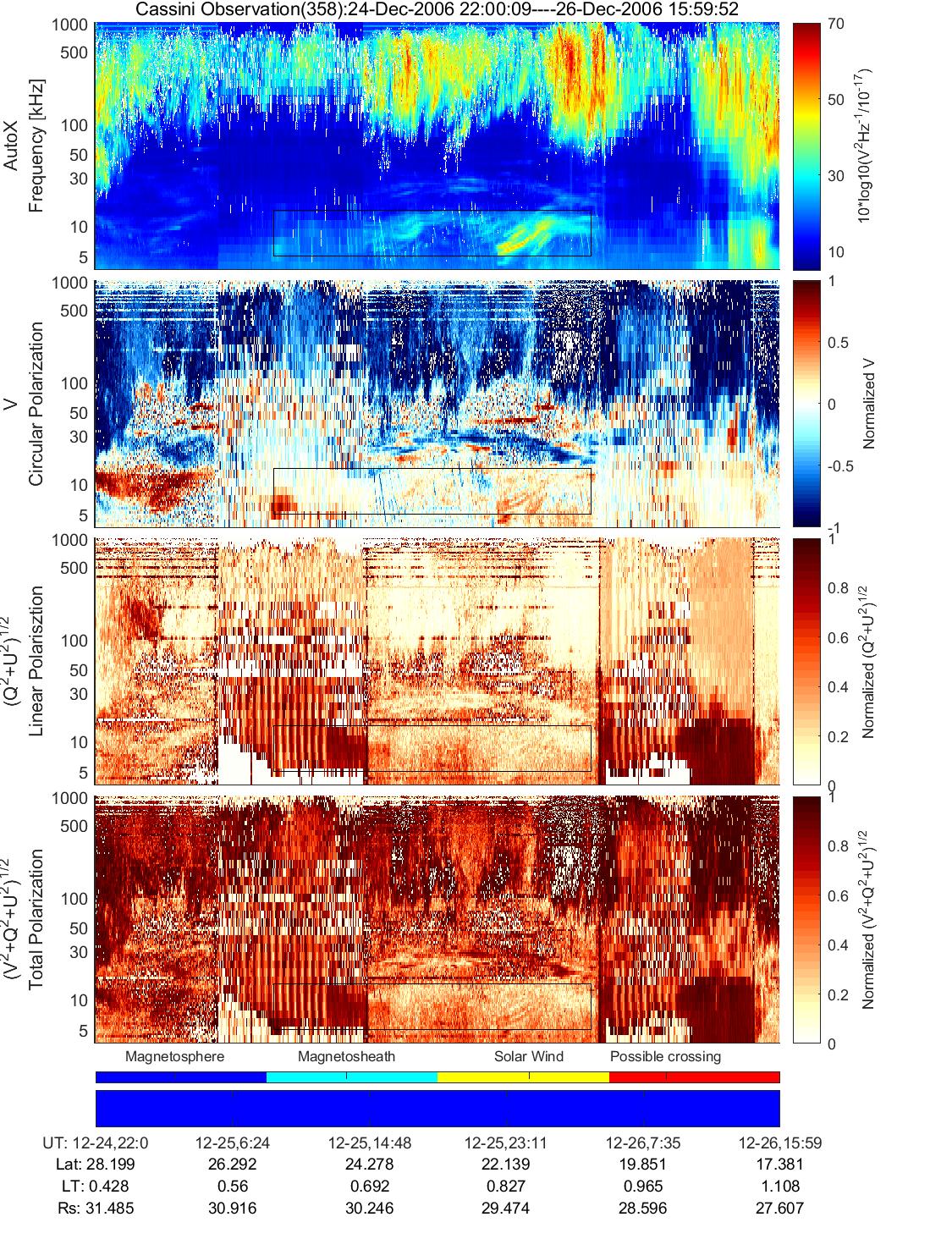


Figure S12, Case 12 of the SAM emission in Table S1. Same format as Figure S1.


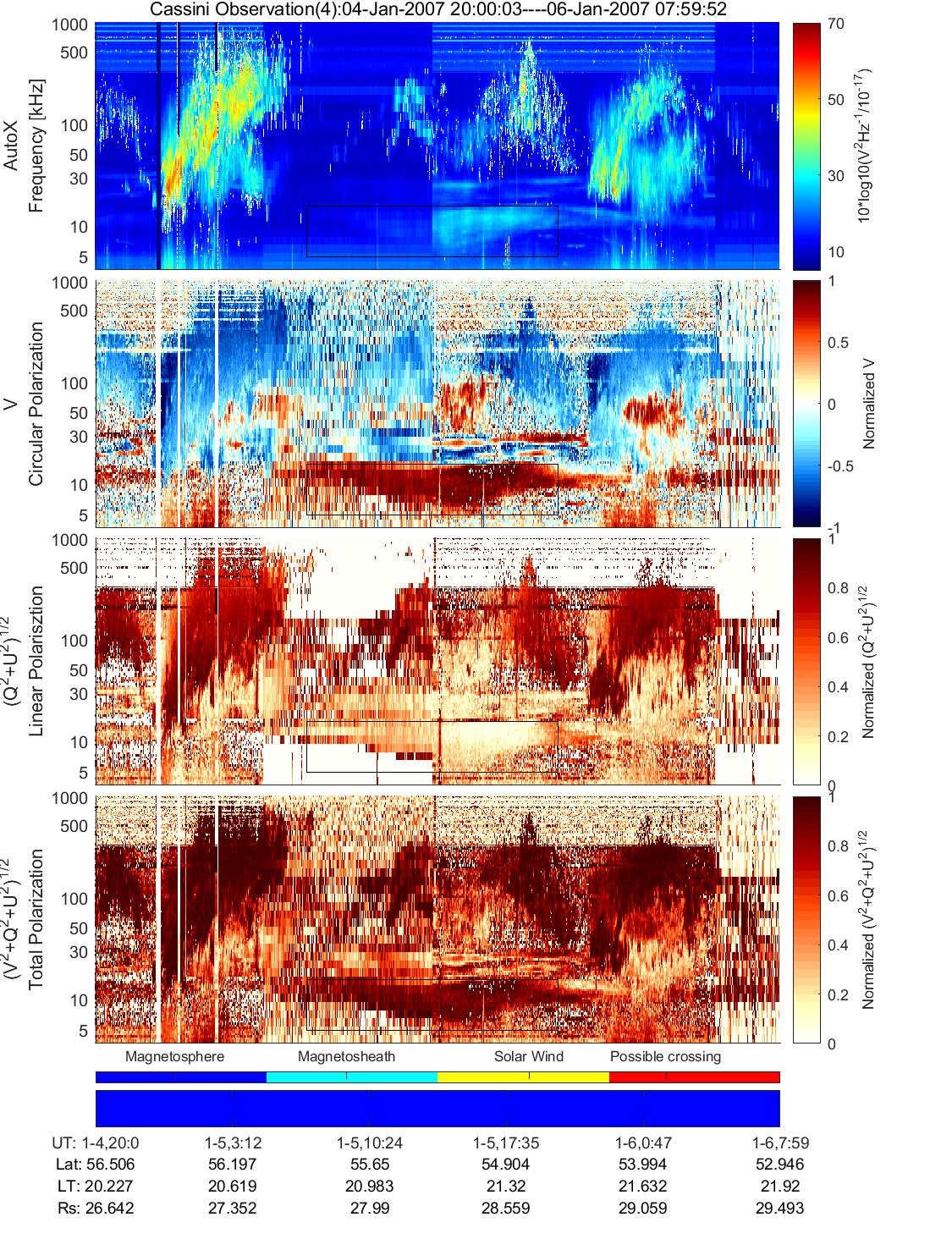


Figure S13, Case 13 of the SAM emission in Table S1. Same format as Figure S1.


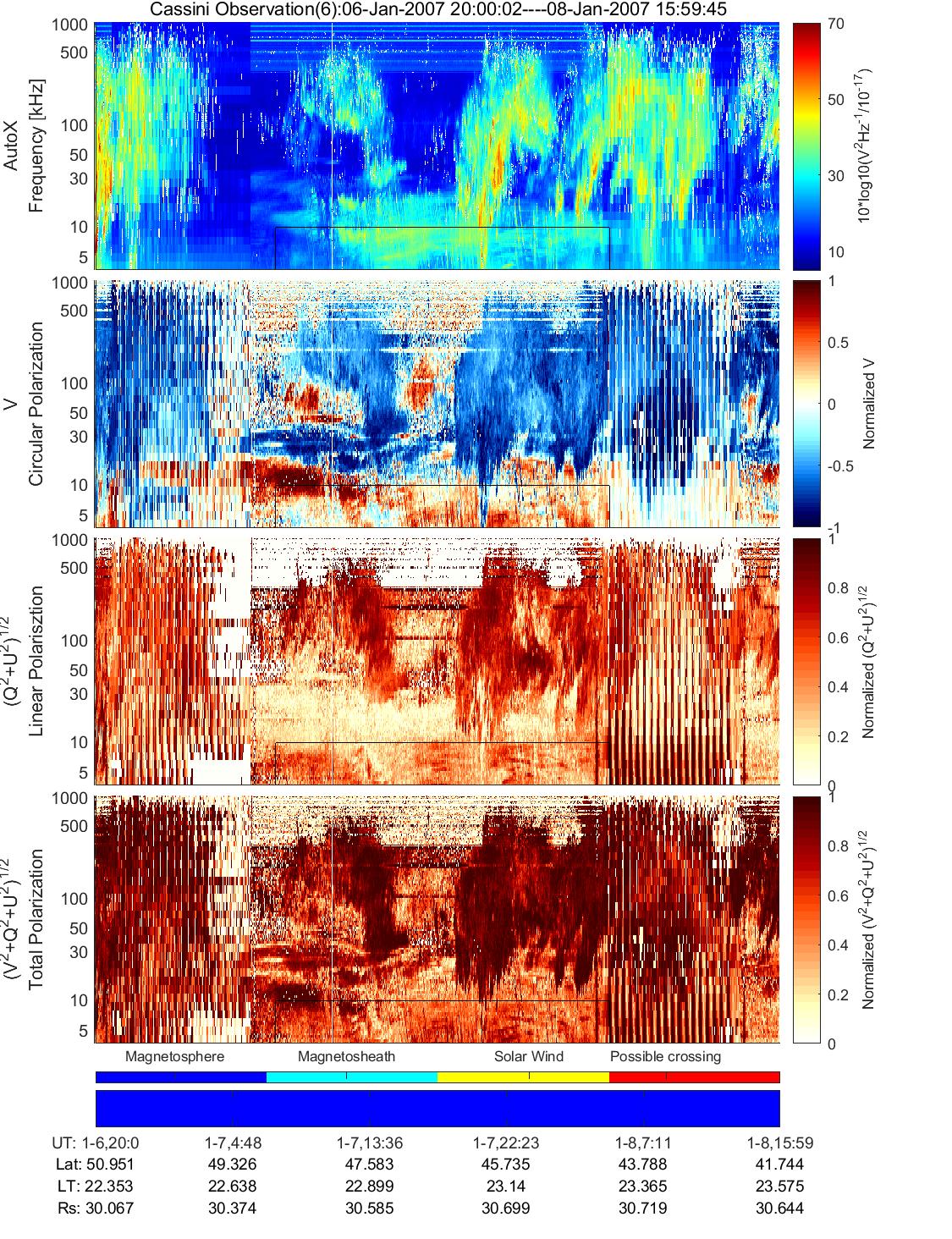


Figure S14, Case 14 of the SAM emission in Table S1. Same format as Figure S1.


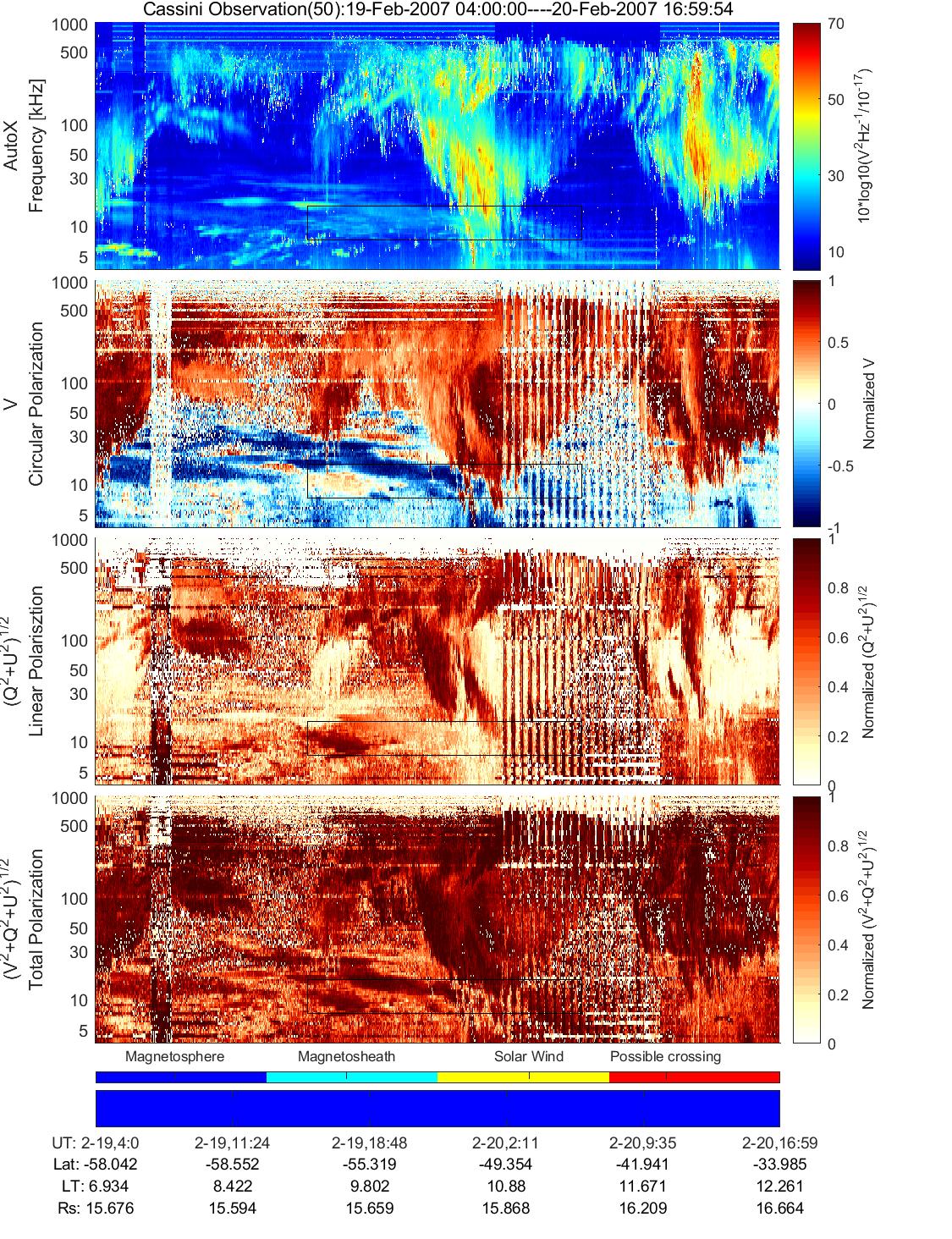


Figure S15, Case 15 of the SAM emission in Table S1. Same format as Figure S1.


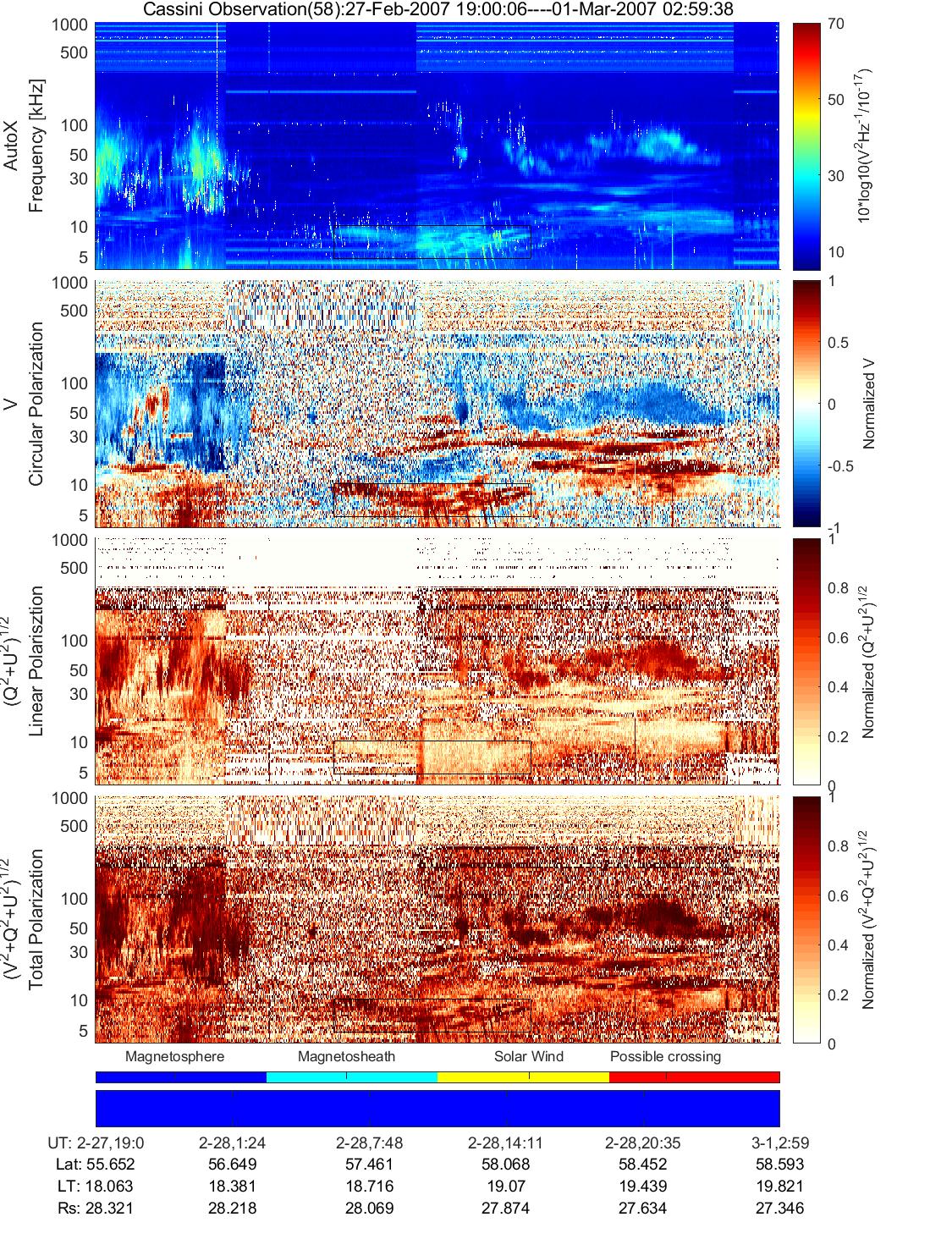


Figure S16, Case 16 of the SAM emission in Table S1. Same format as Figure S1.


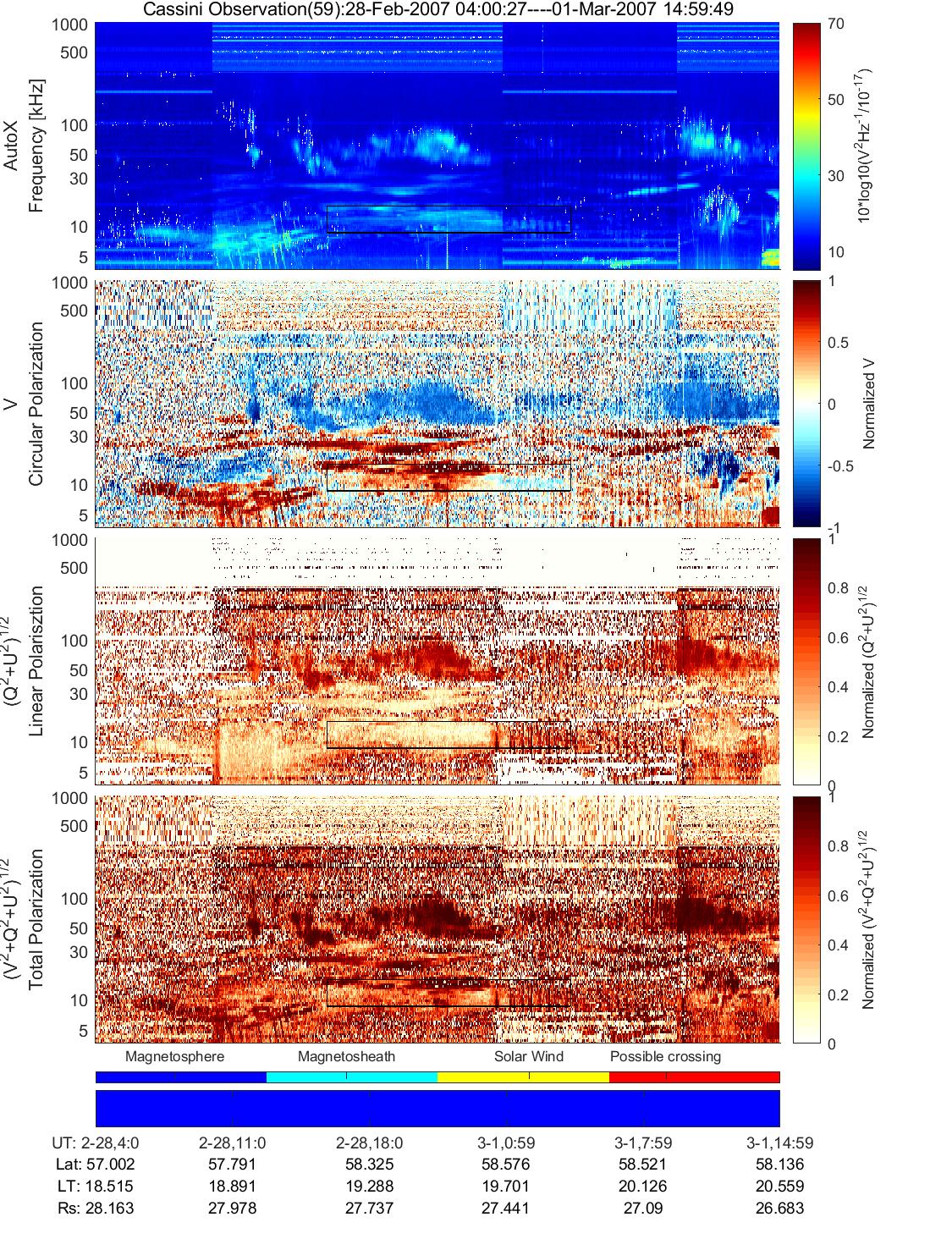


Figure S17, Case 17 of the SAM emission in Table S1. Same format as Figure S1.


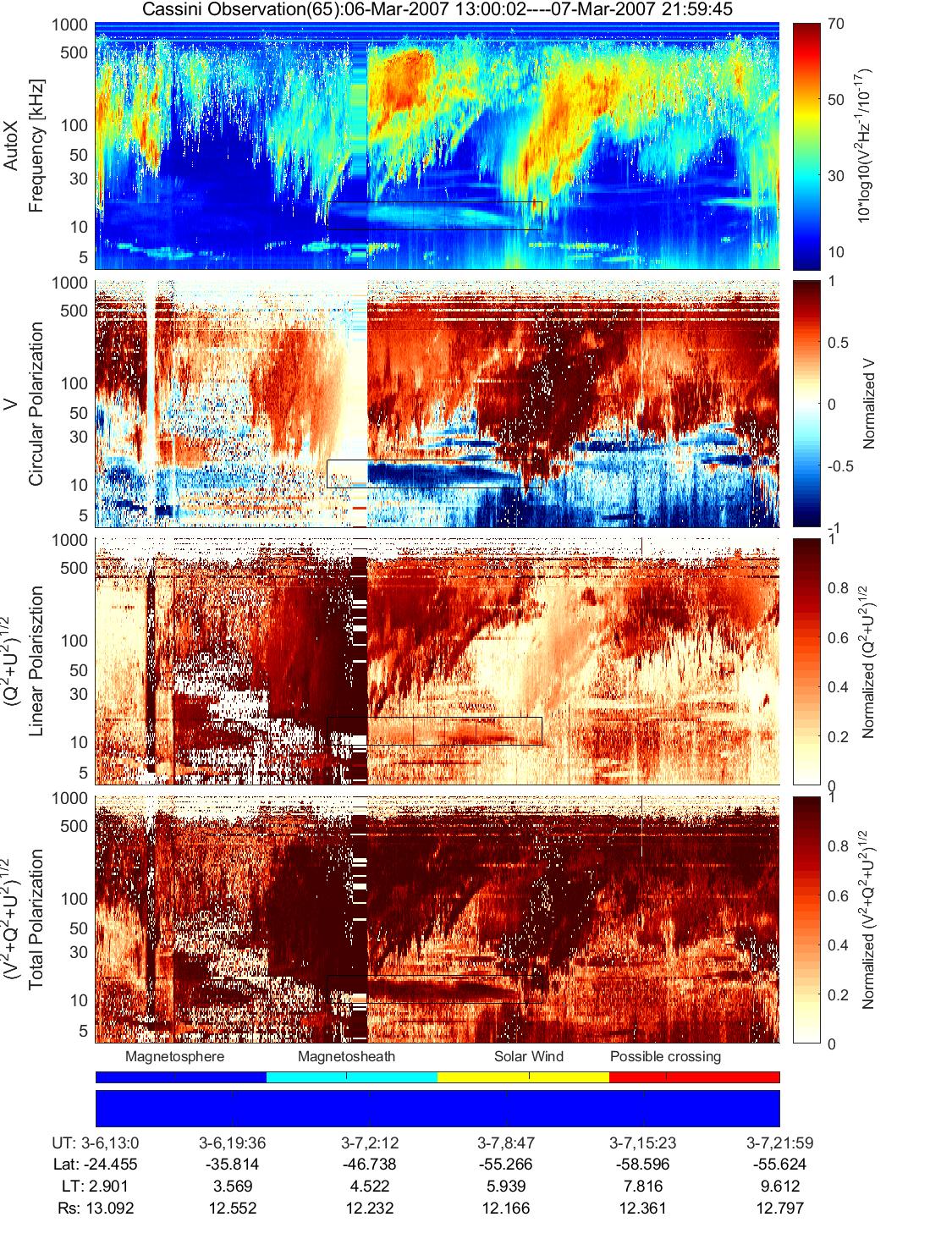


Figure S18, Case 18 of the SAM emission in Table S1. Same format as Figure S1.


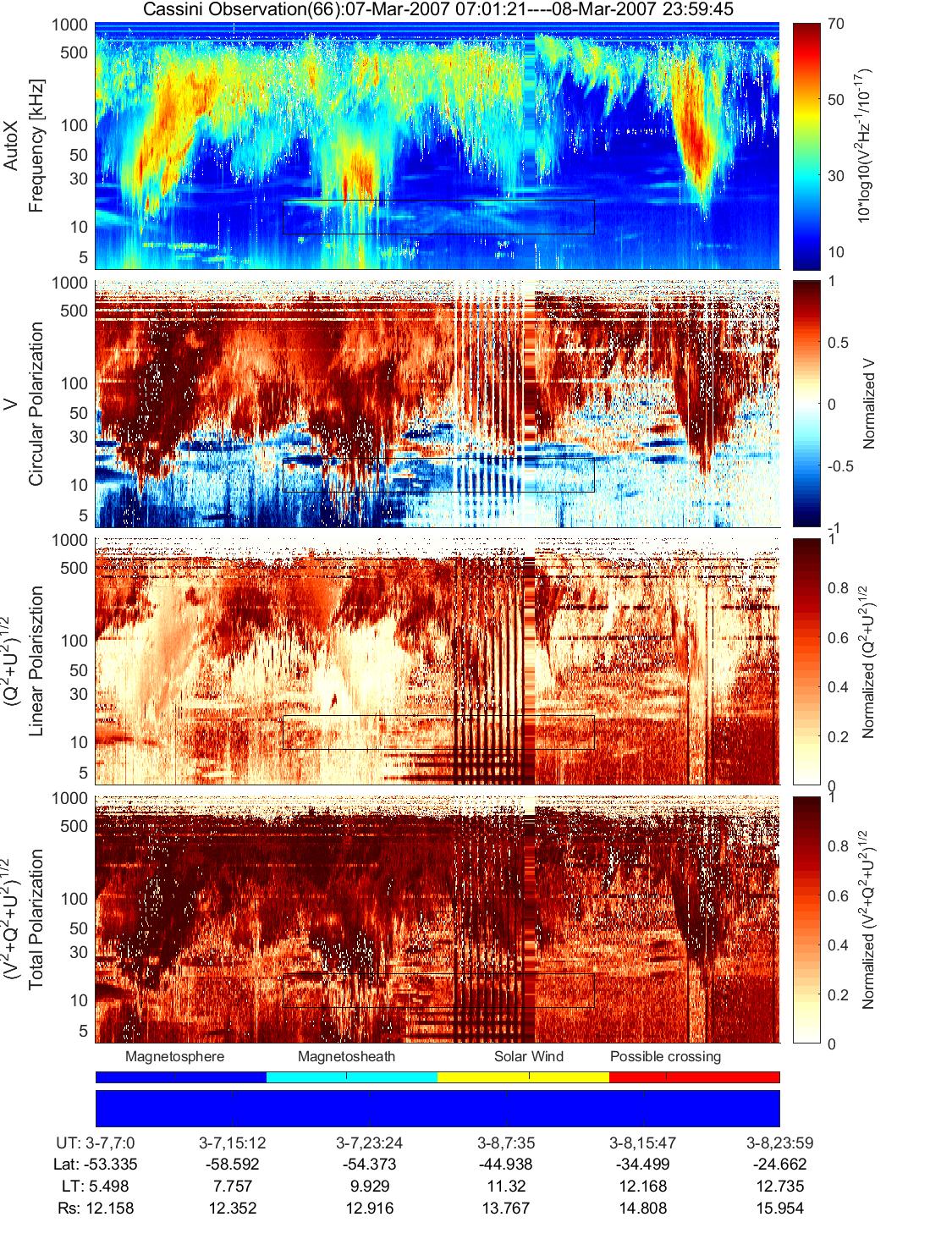


Figure S19, Case 19 of the SAM emission in Table S1. Same format as Figure S1.


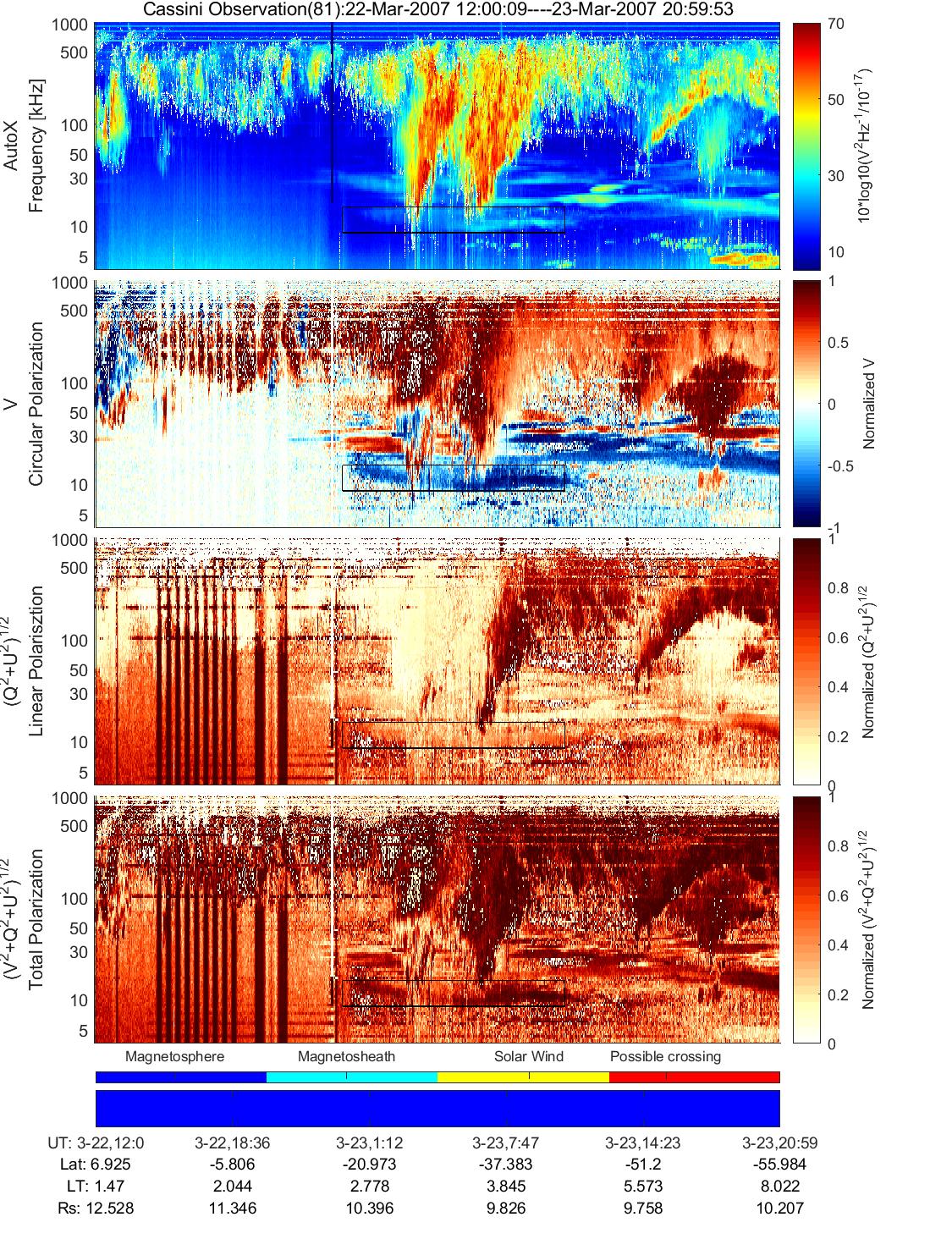


Figure S20, Case 20 of the SAM emission in Table S1. Same format as Figure S1.


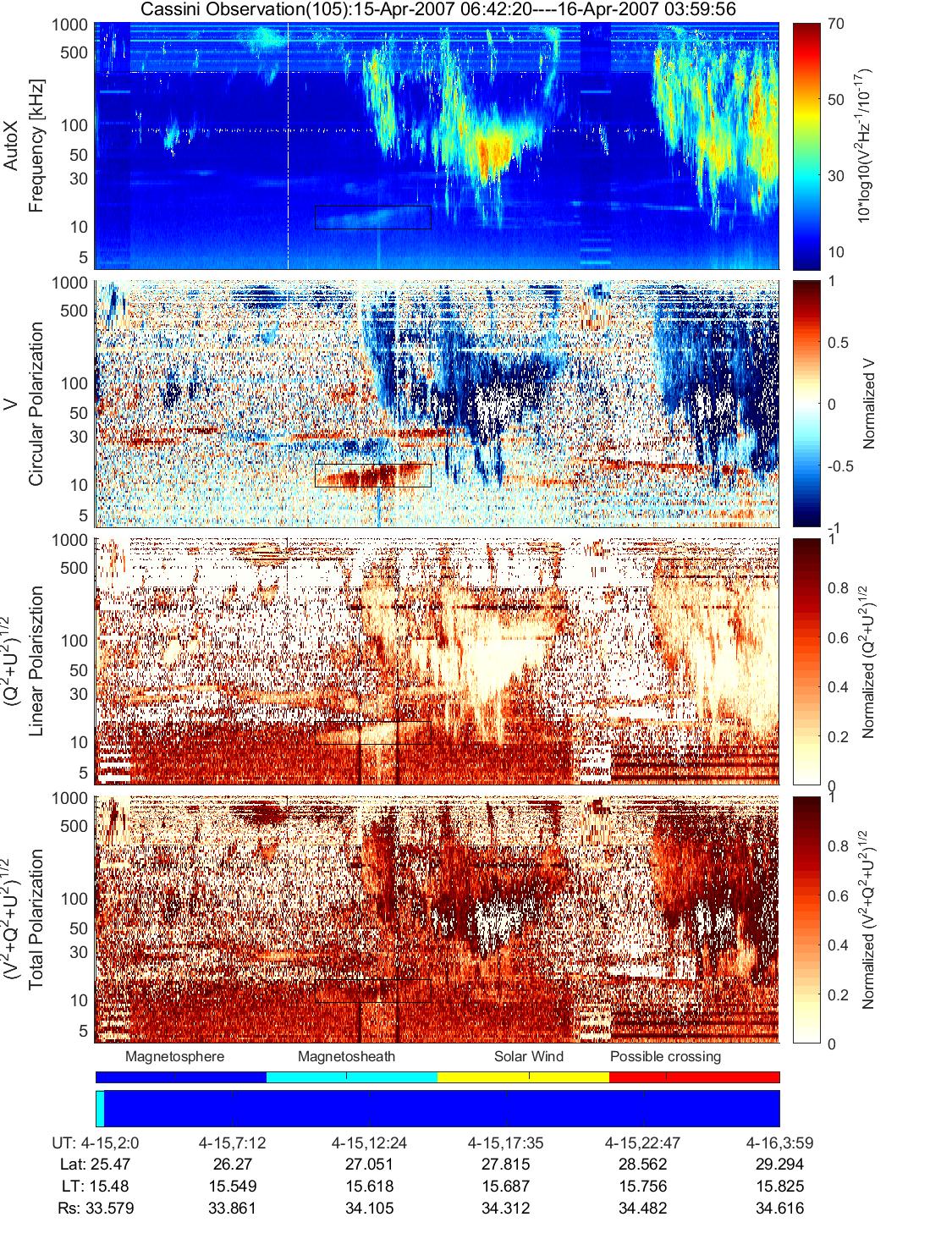


Figure S21, Case 21 of the SAM emission in Table S1. Same format as Figure S1.


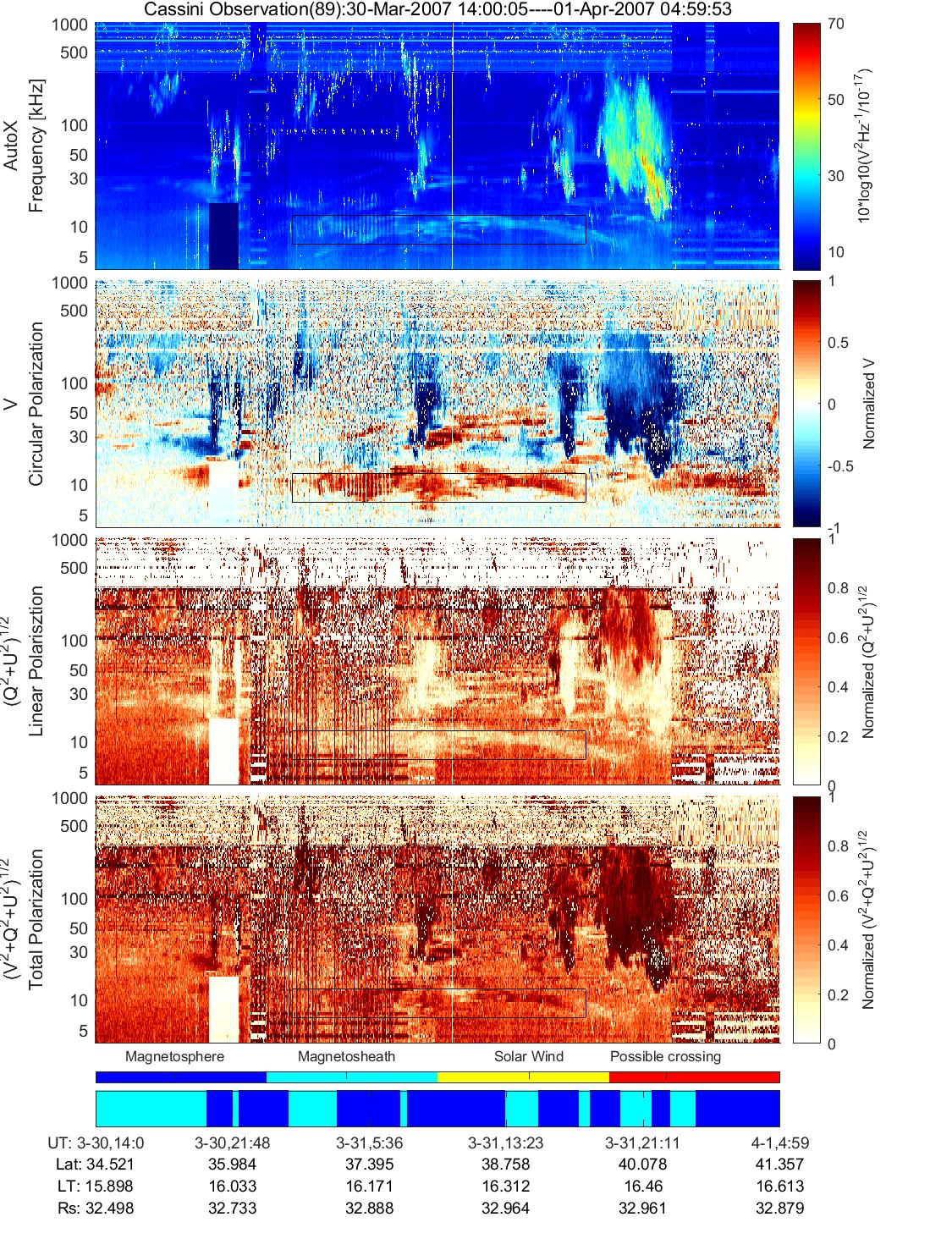


Figure S22, Case 22 of the SAM emission in Table S1. Same format as Figure S1.


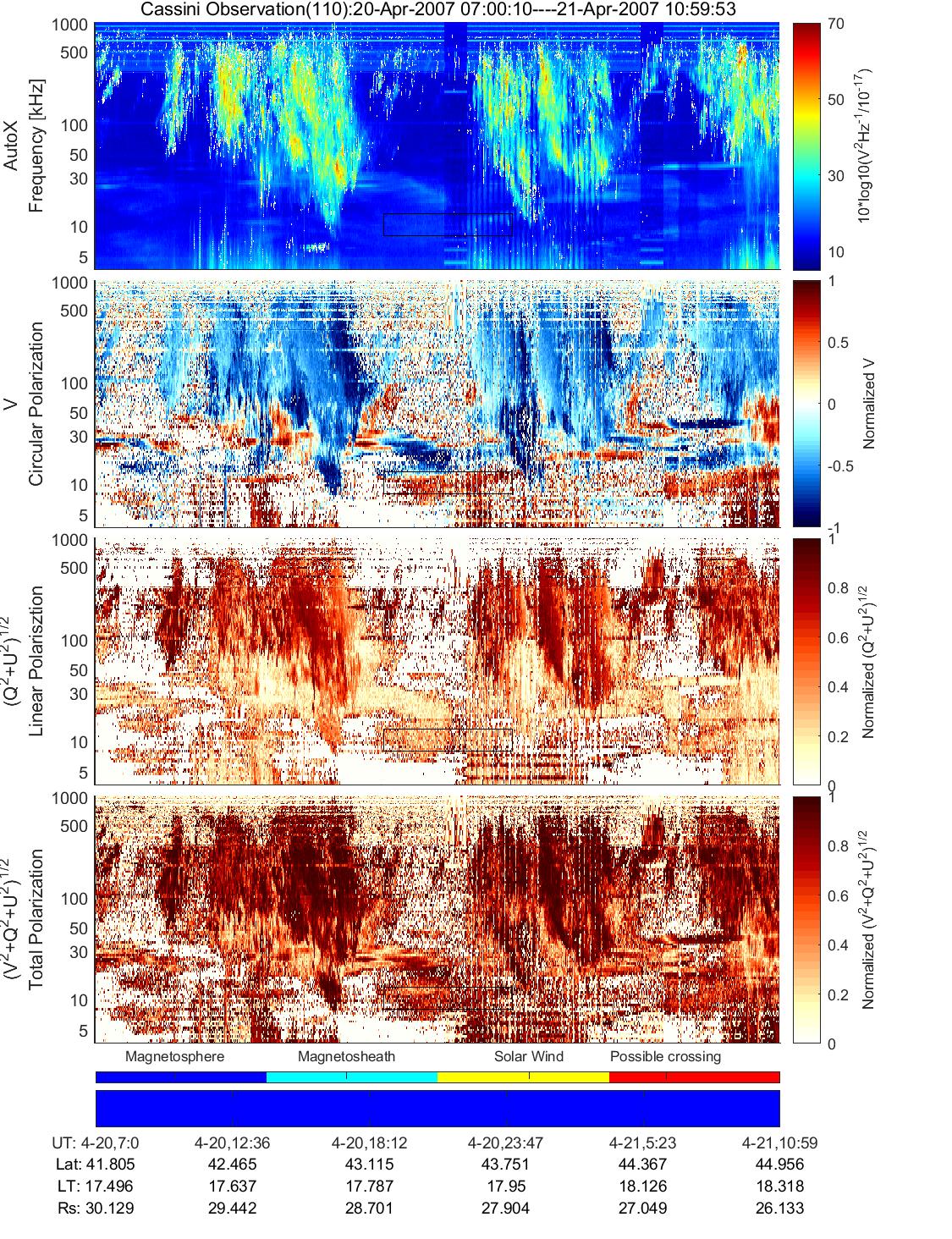


Figure S23, Case 23 of the SAM emission in Table S1. Same format as Figure S1.


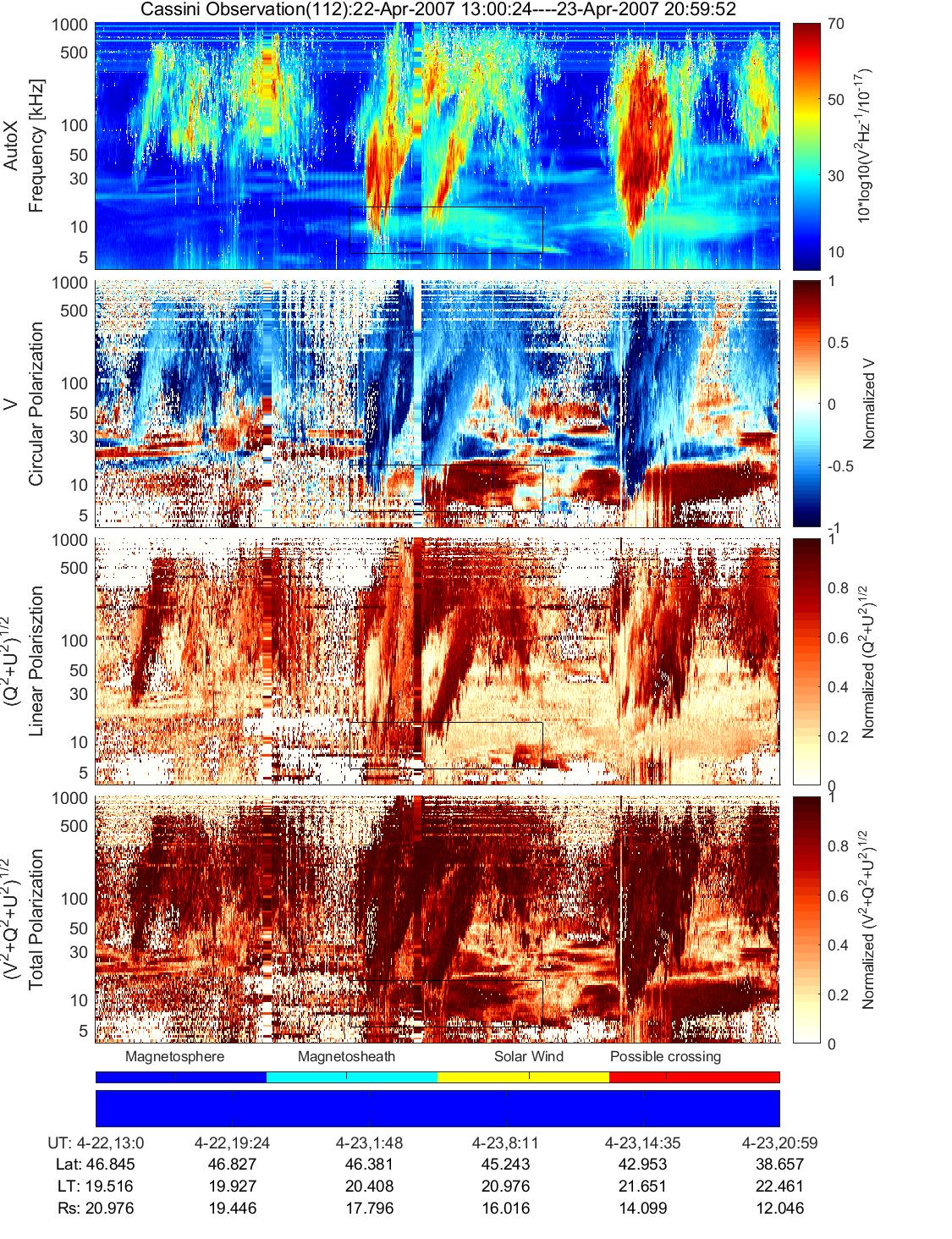


Figure S24, Case 24 of the SAM emission in Table S1. Same format as Figure S1.


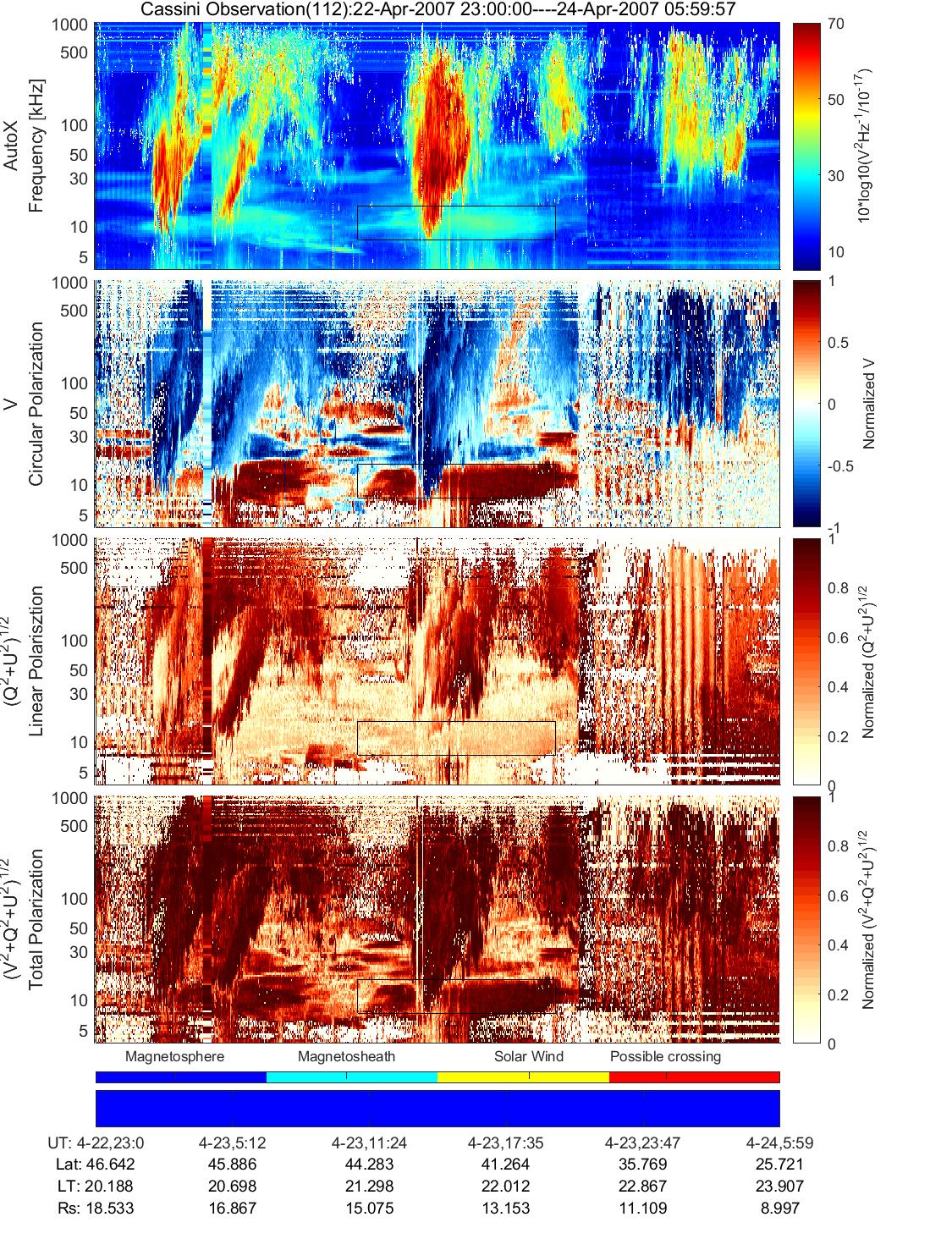


Figure S25, Case 25 of the SAM emission in Table S1. Same format as Figure S1.


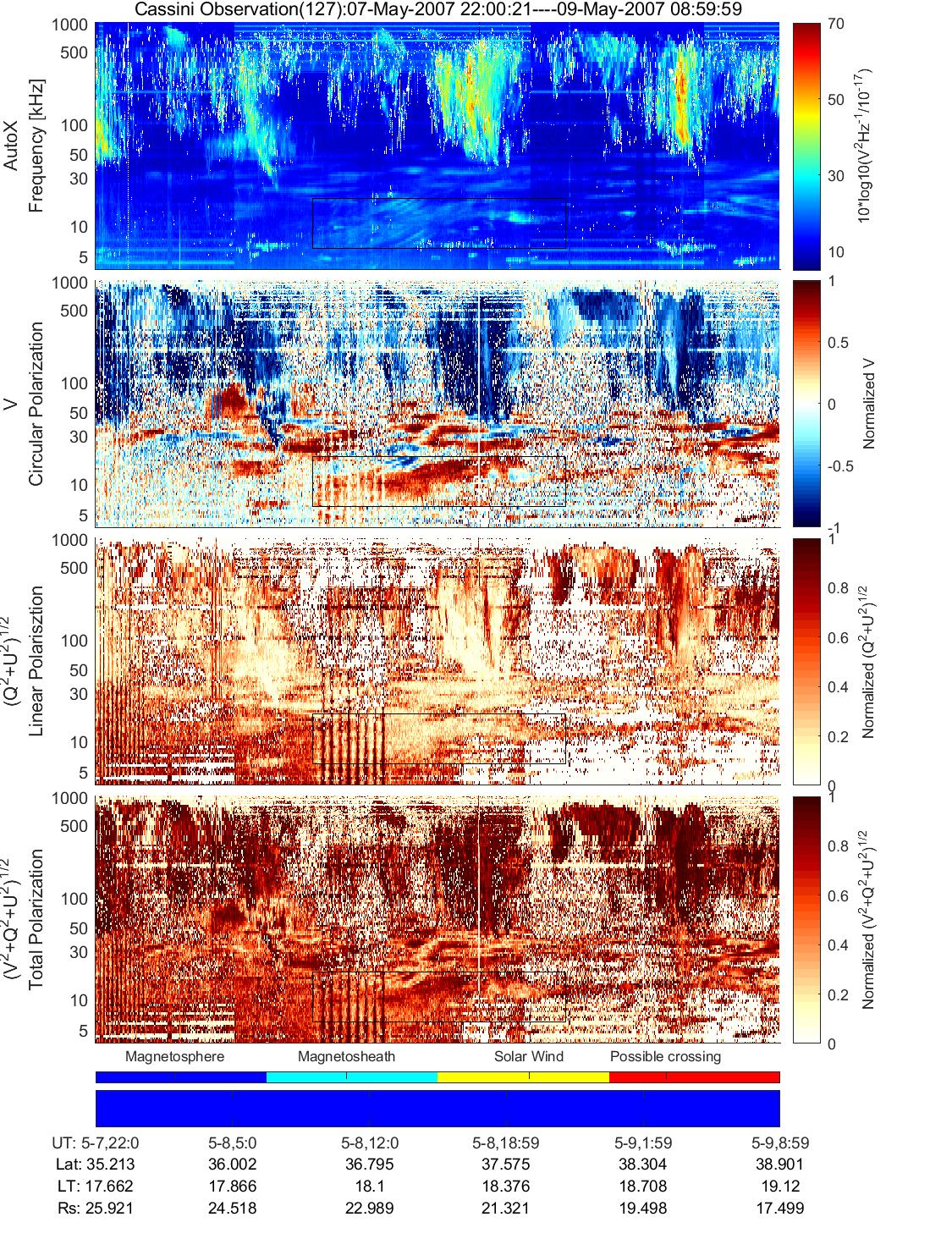


Figure S26, Case 26 of the SAM emission in Table S1. Same format as Figure S1.


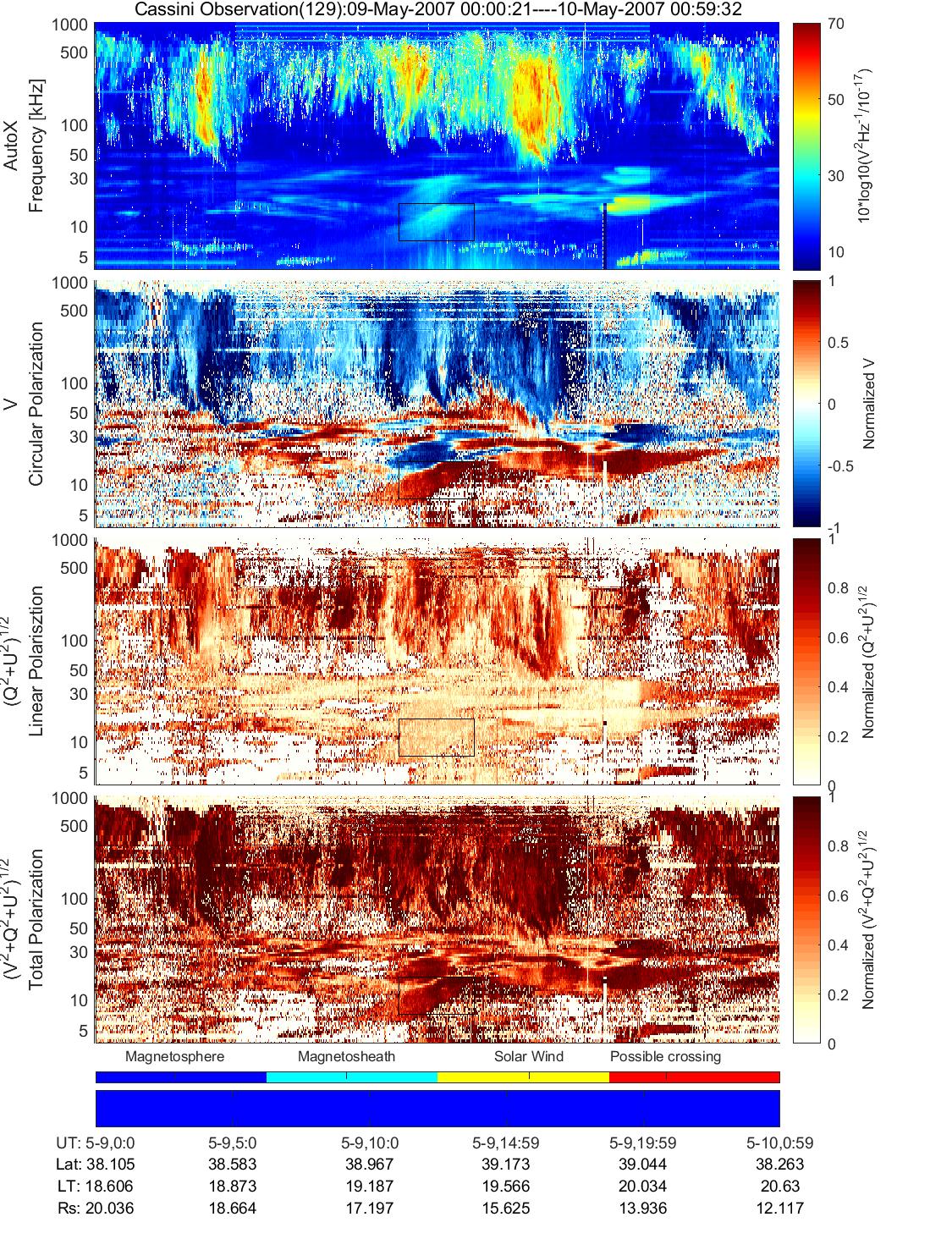


Figure S27, Case 27 of the SAM emission in Table S1. Same format as Figure S1.


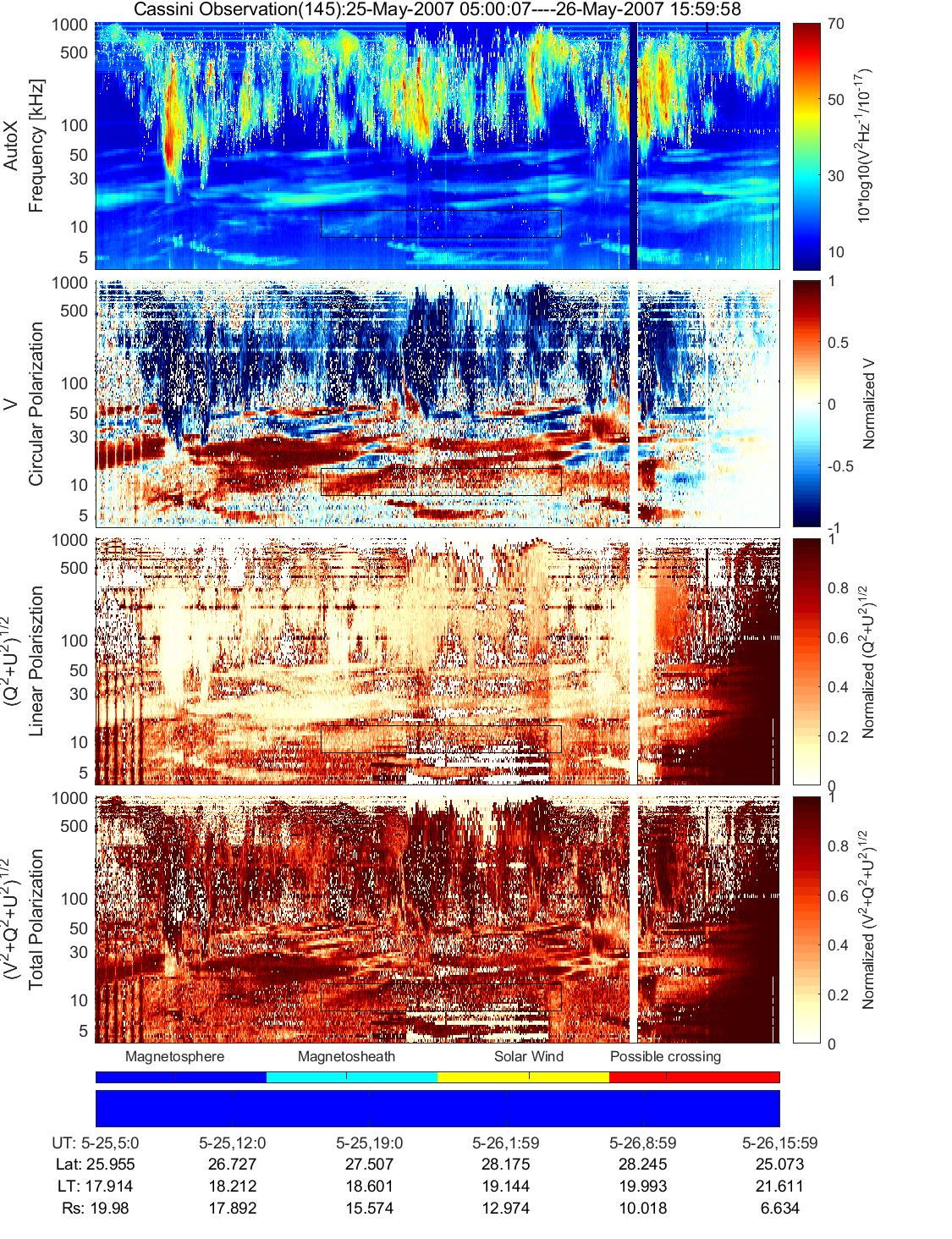


Figure S28, Case 28 of the SAM emission in Table S1. Same format as Figure S1.


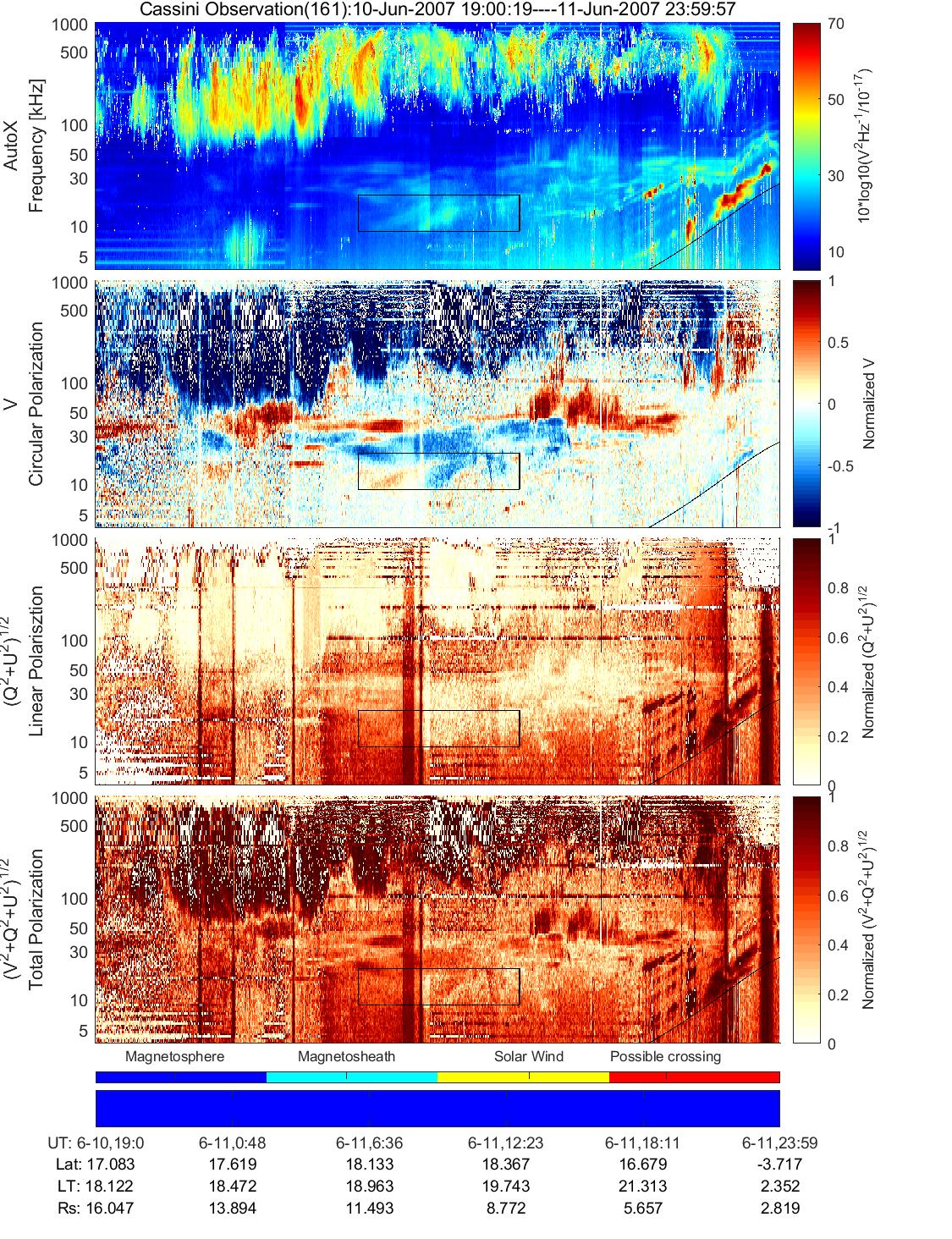
 Figure S29, Case 29 of the SAM emission in Table S1. Same format as Figure S1.


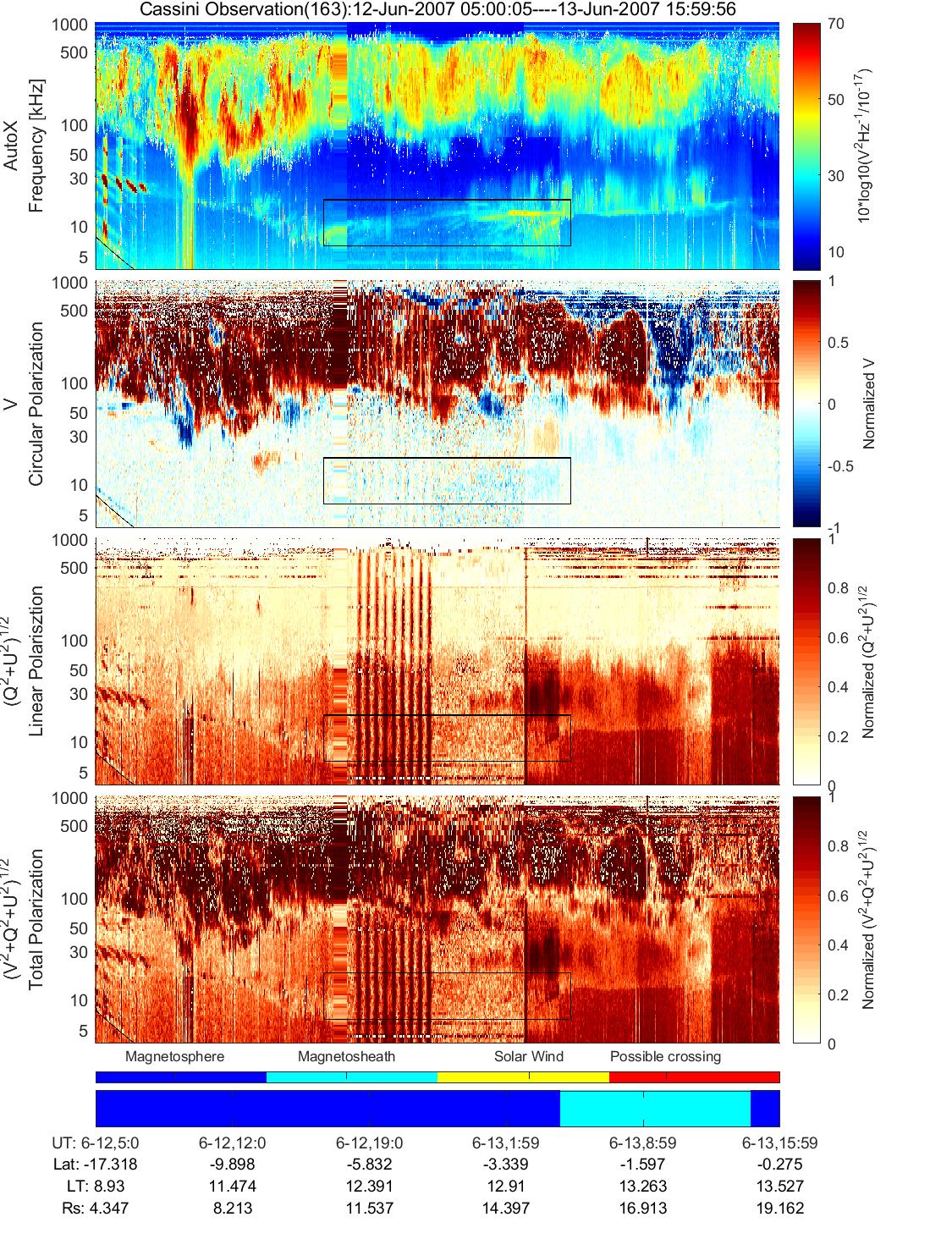


Figure S30, Case 30 of the SAM emission in Table S1. Same format as Figure S1.


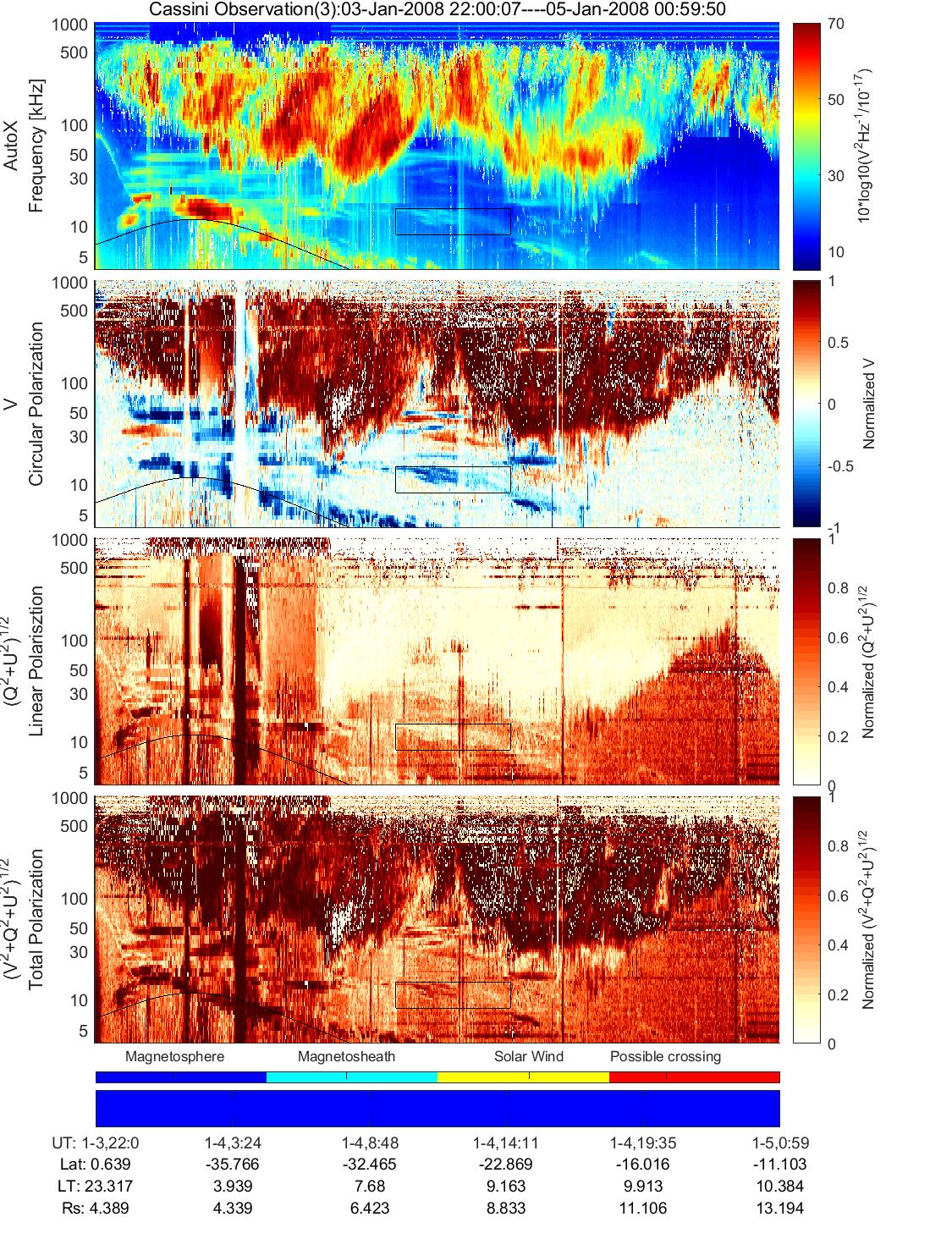


Figure S31, Case 31 of the SAM emission in Table S1. Same format as Figure S1.


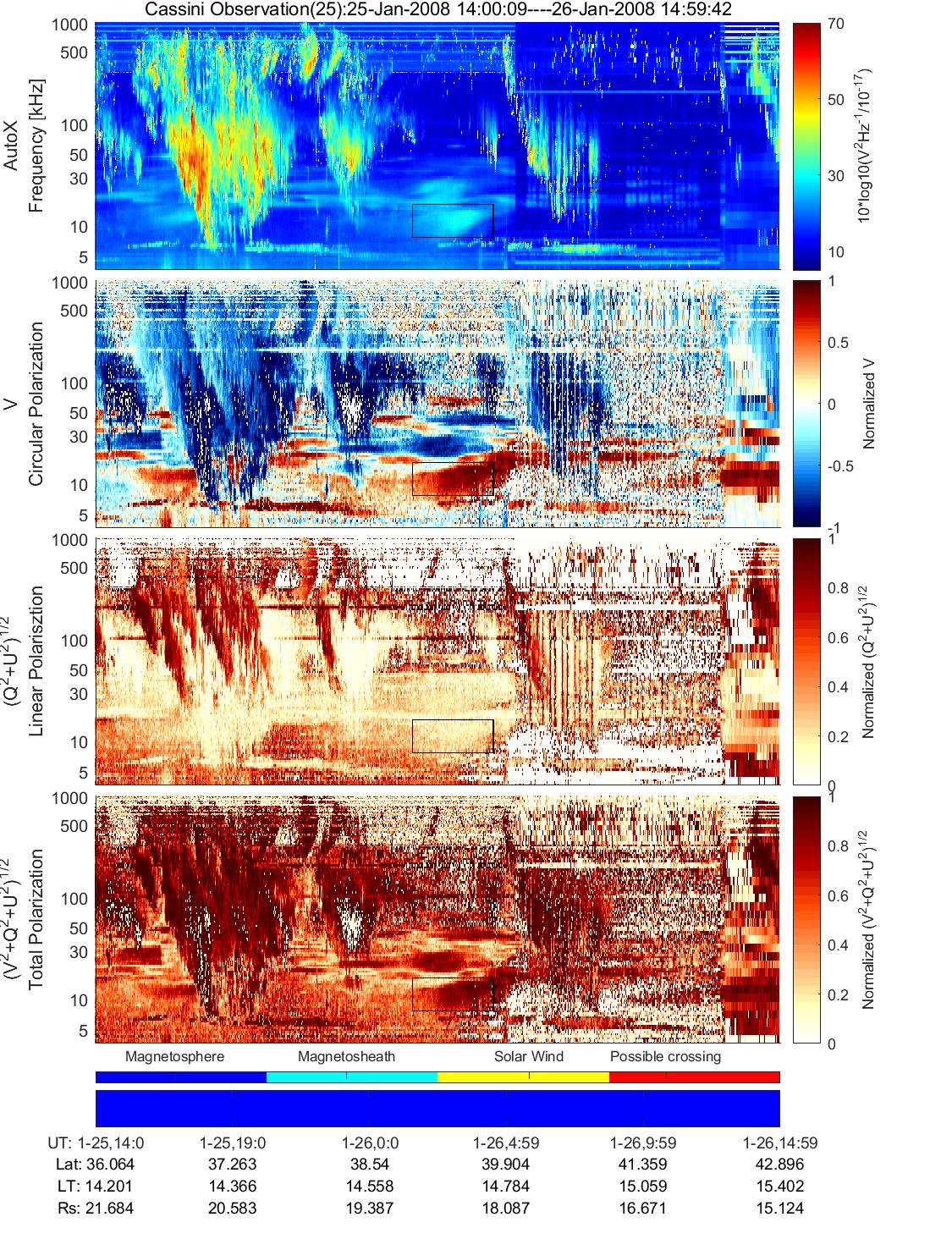


Figure S32, Case 32 of the SAM emission in Table S1. Same format as Figure S1.


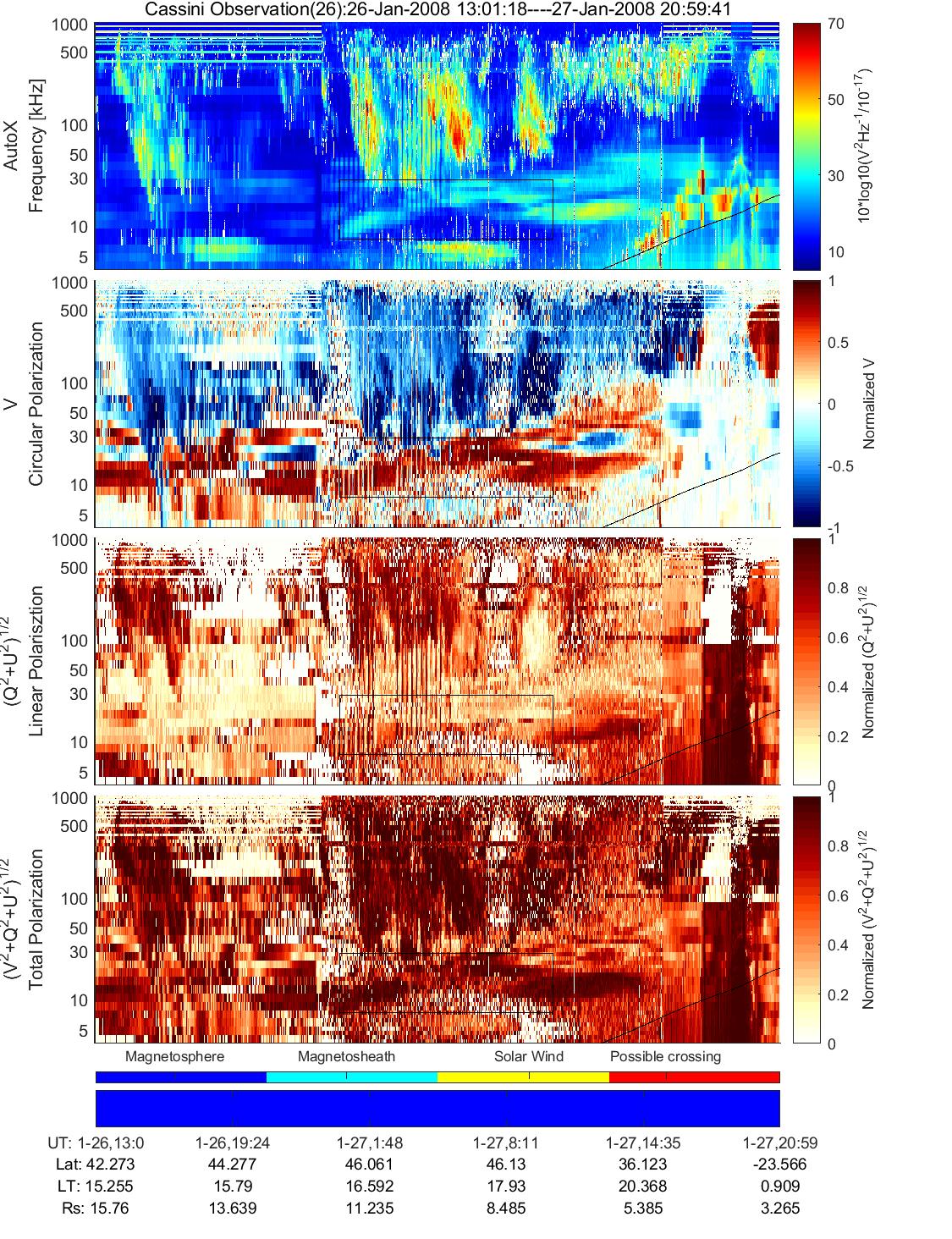


Figure S33, Case 33 of the SAM emission in Table S1. Same format as Figure S1.


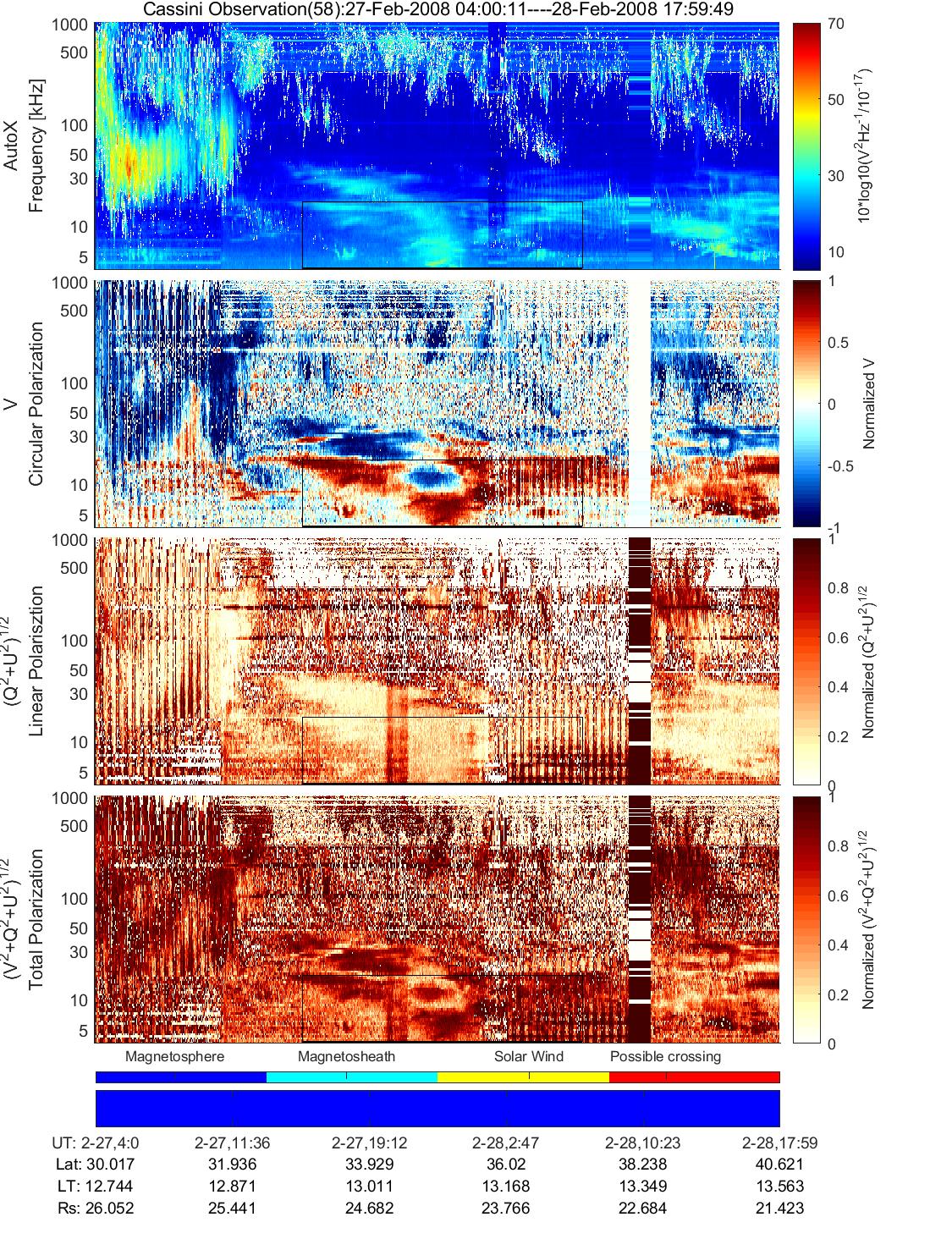


Figure S34, Case 34 of the SAM emission in Table S1. Same format as Figure S1.


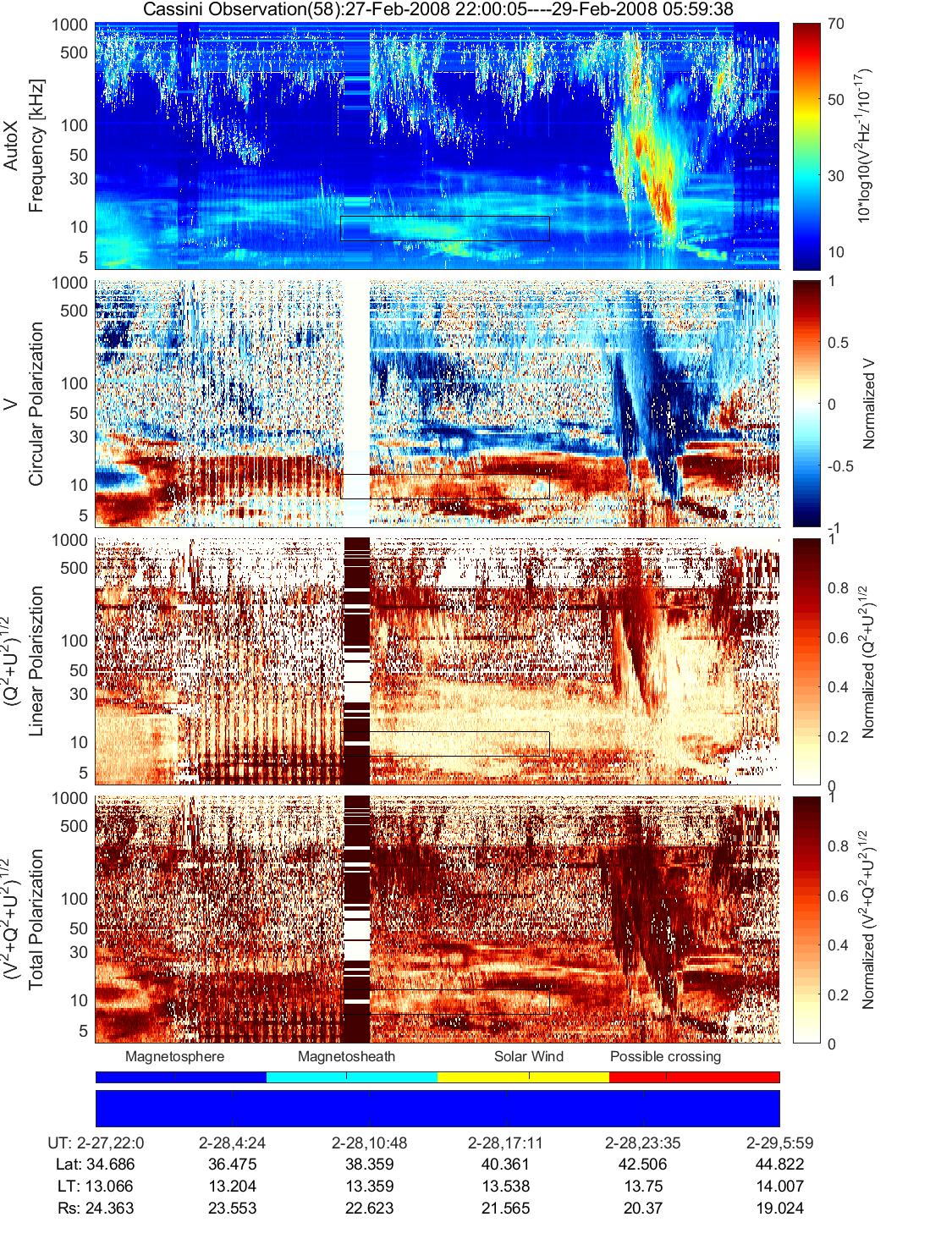


Figure S35, Case 35 of the SAM emission in Table S1. Same format as Figure S1.


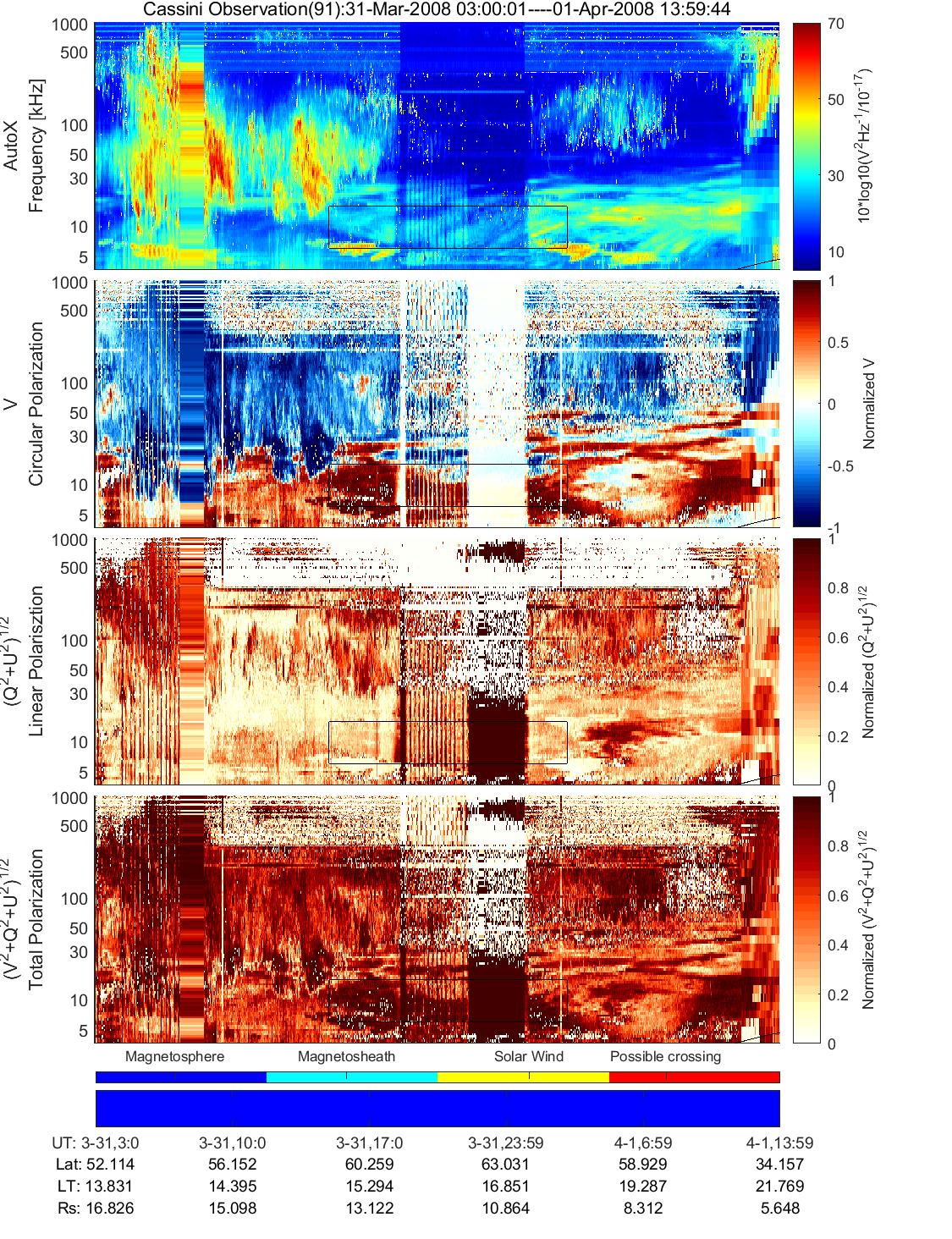


Figure S36, Case 36 of the SAM emission in Table S1. Same format as Figure S1.


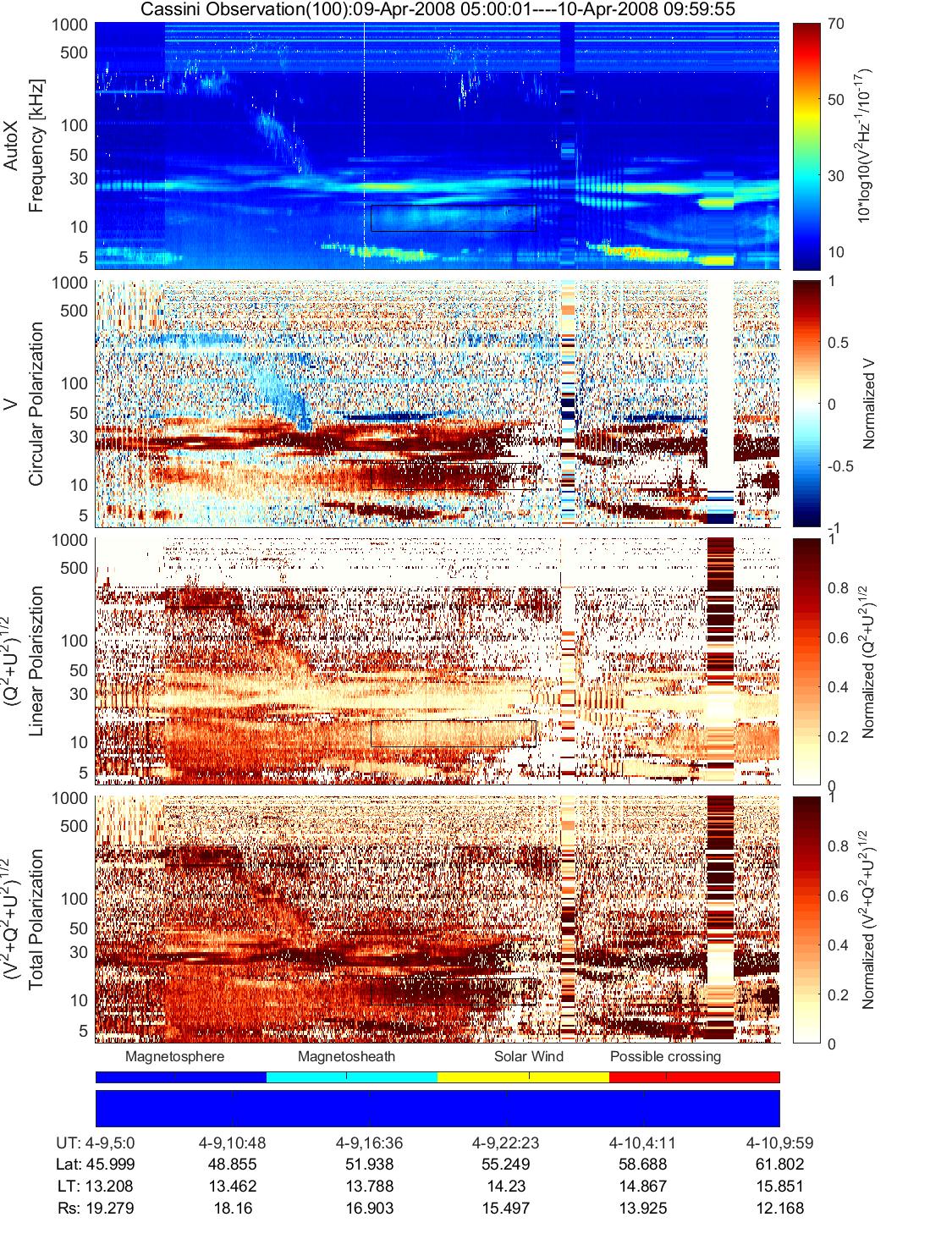


Figure S37, Case 37 of the SAM emission in Table S1. Same format as Figure S1.


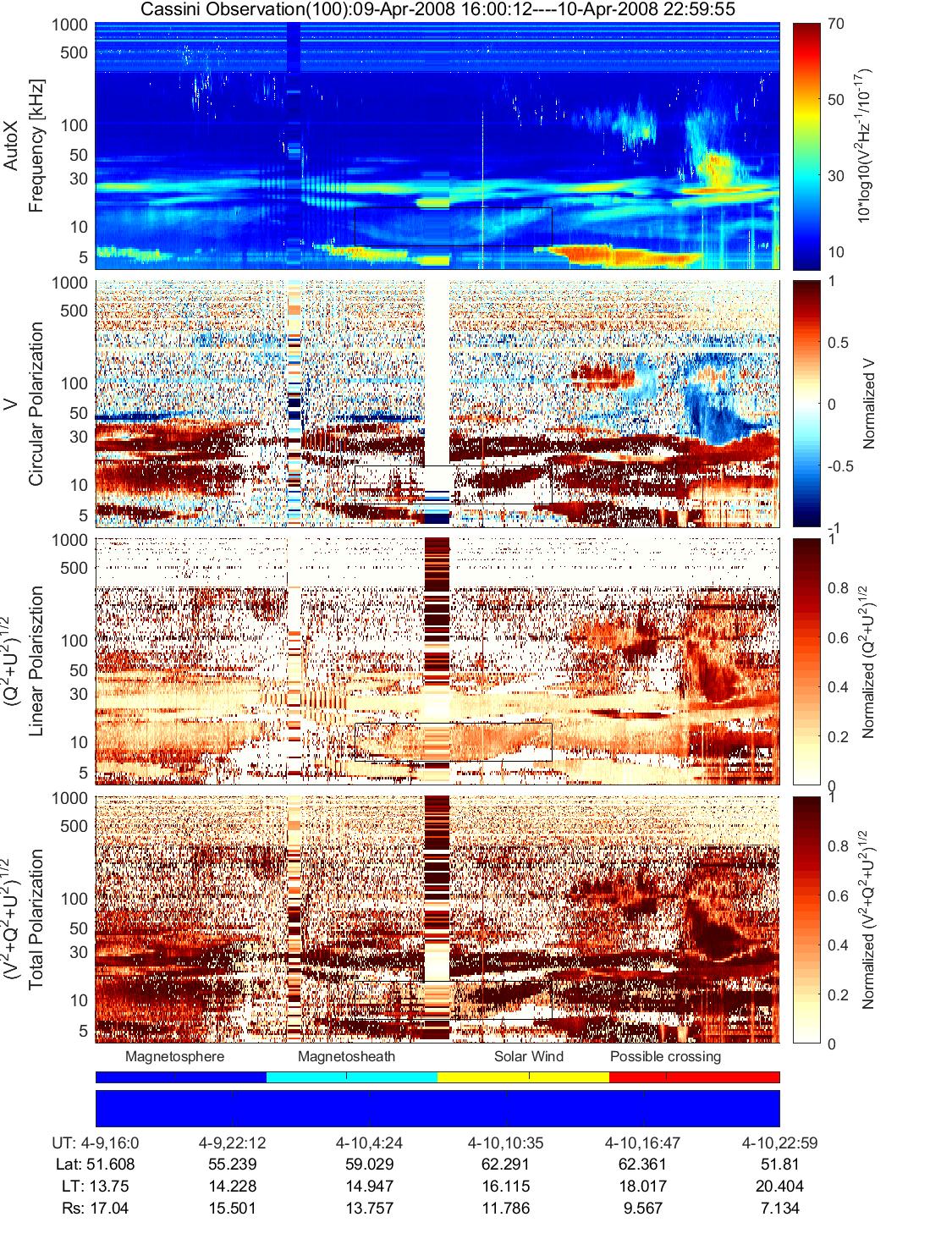


Figure S38, Case 38 of the SAM emission in Table S1. Same format as Figure S1.


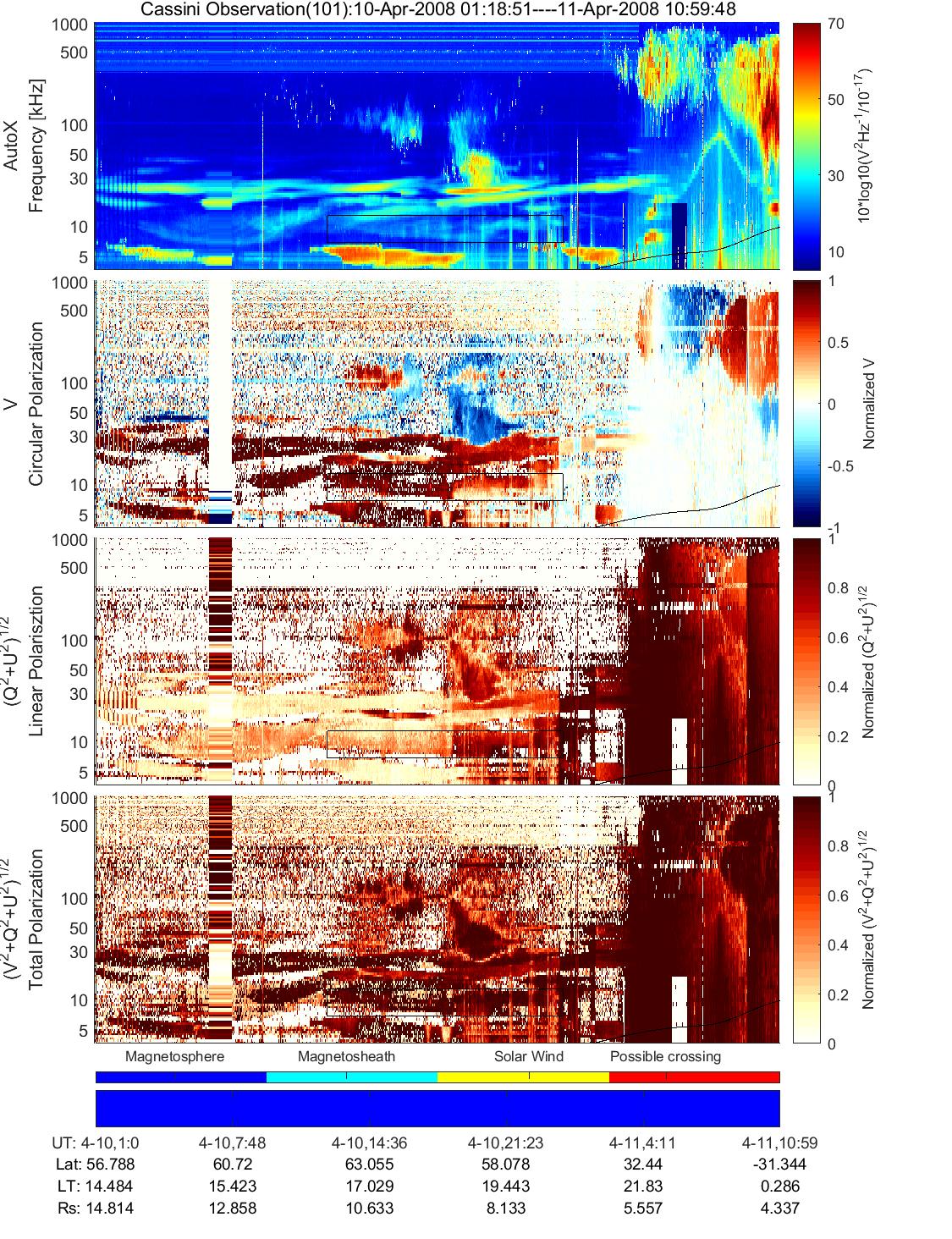


Figure S39, Case 39 of the SAM emission in Table S1. Same format as Figure S1.


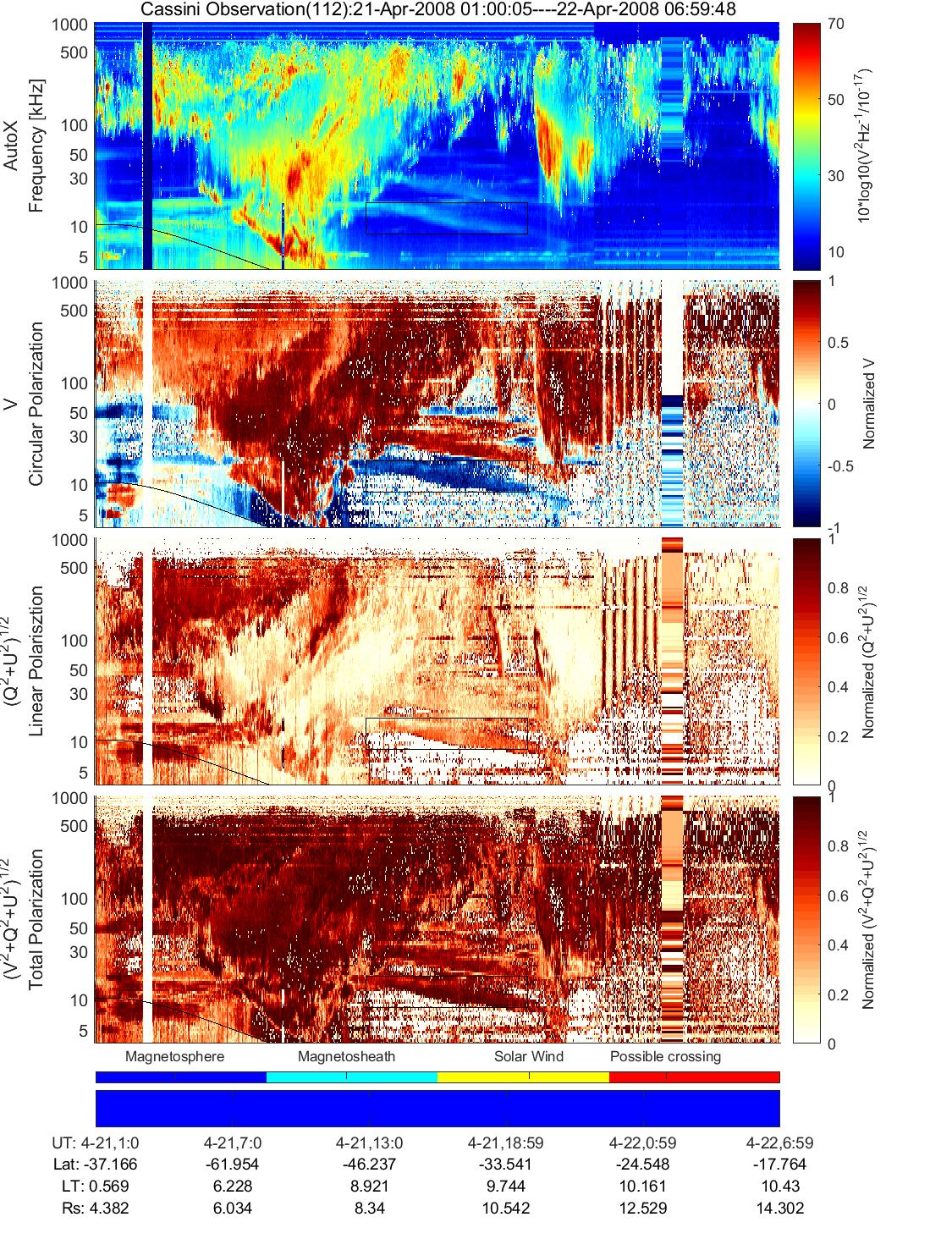


Figure S40, Case 40 of the SAM emission in Table S1. Same format as Figure S1.


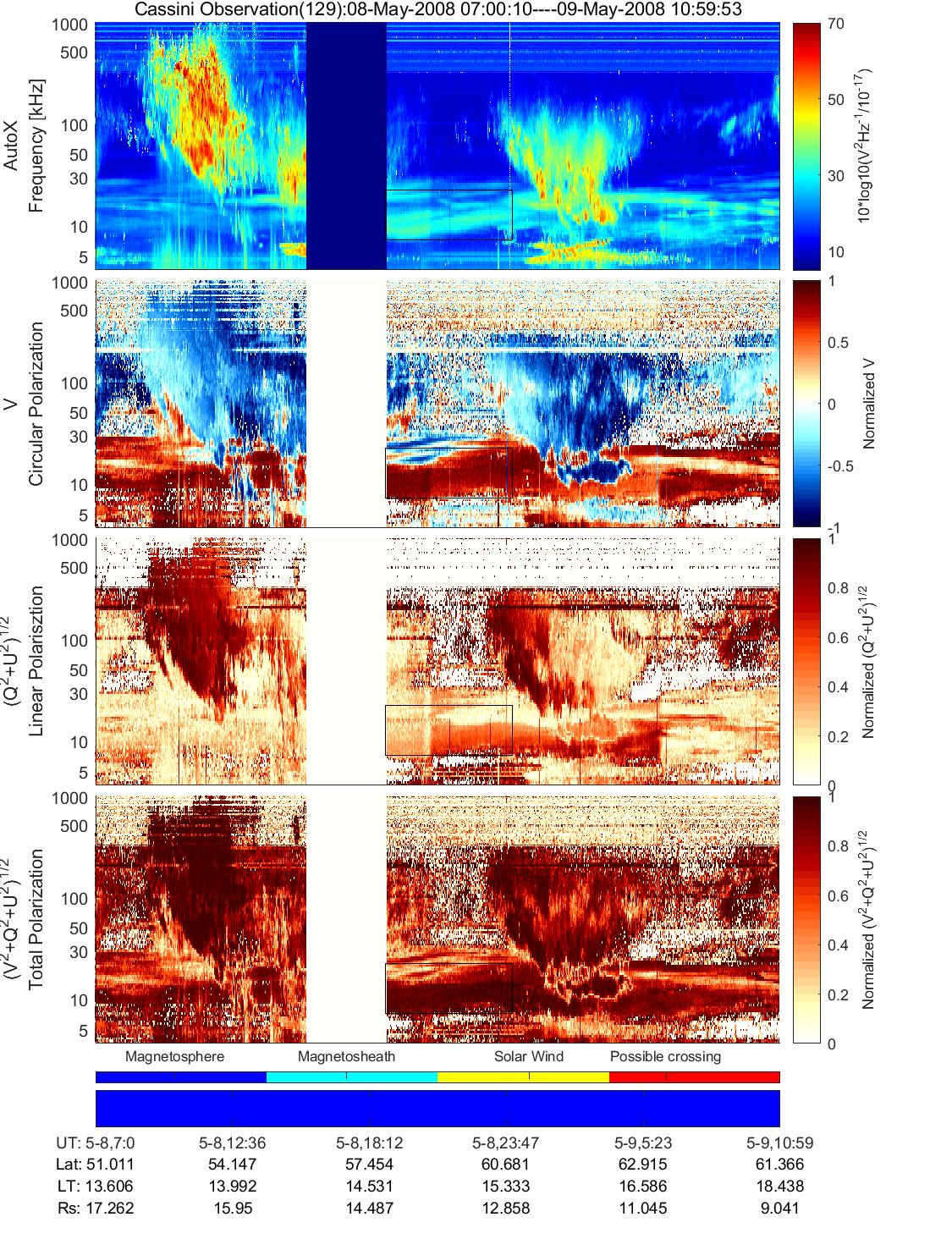


Figure S41, Case 41 of the SAM emission in Table S1. Same format as Figure S1.


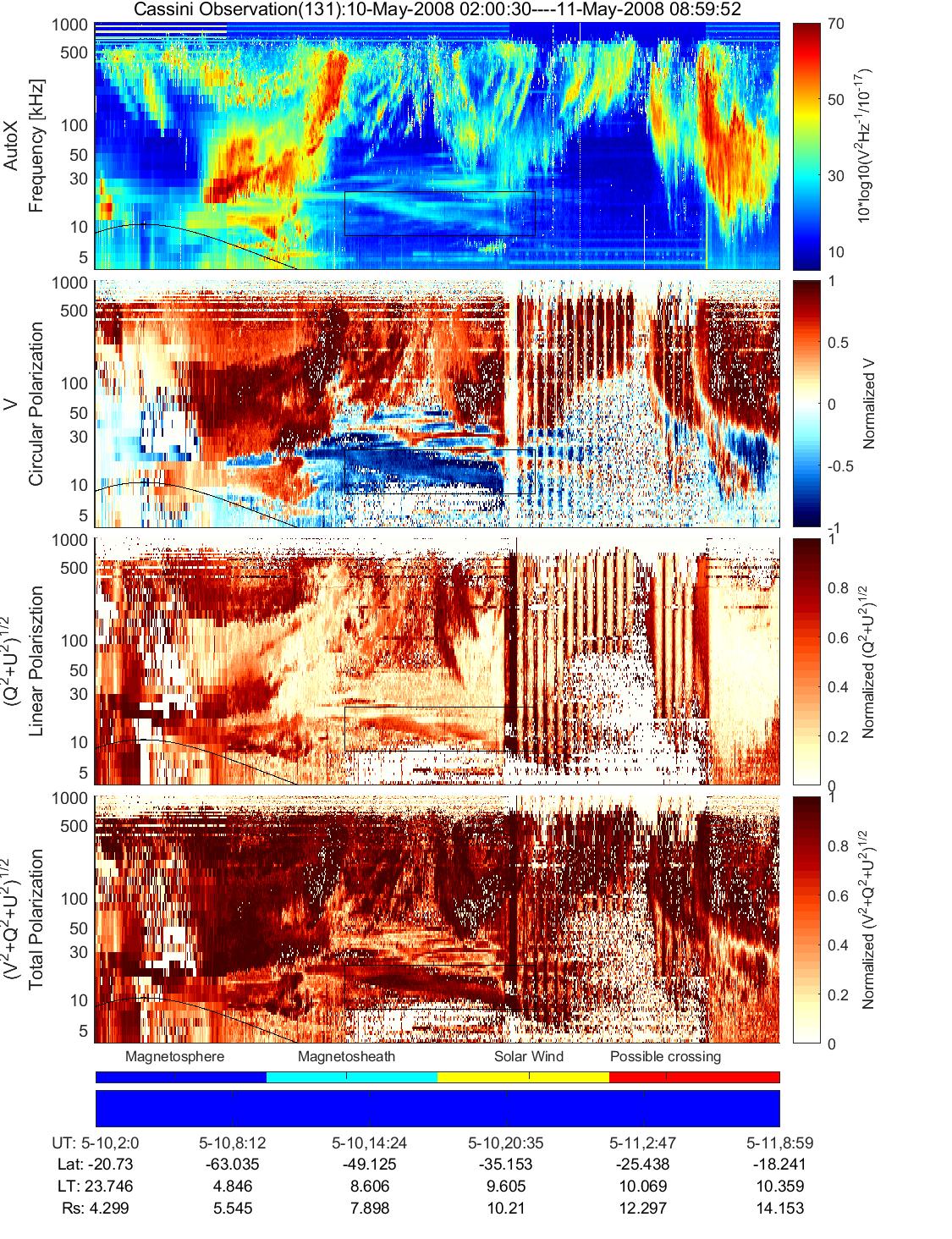


Figure S42, Case 42 of the SAM emission in Table S1. Same format as Figure S1.


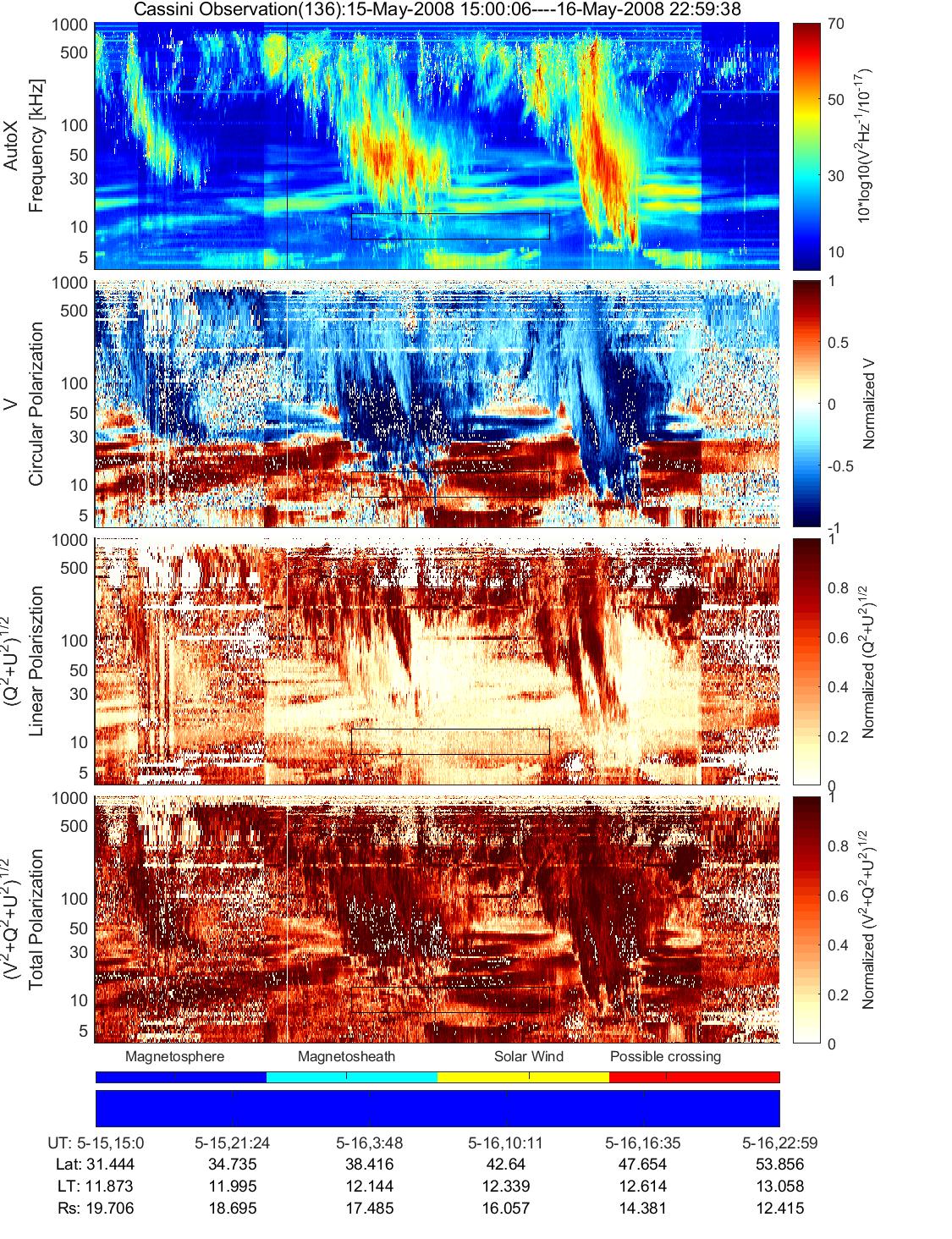


Figure S43, Case 43 of the SAM emission in Table S1. Same format as Figure S1.


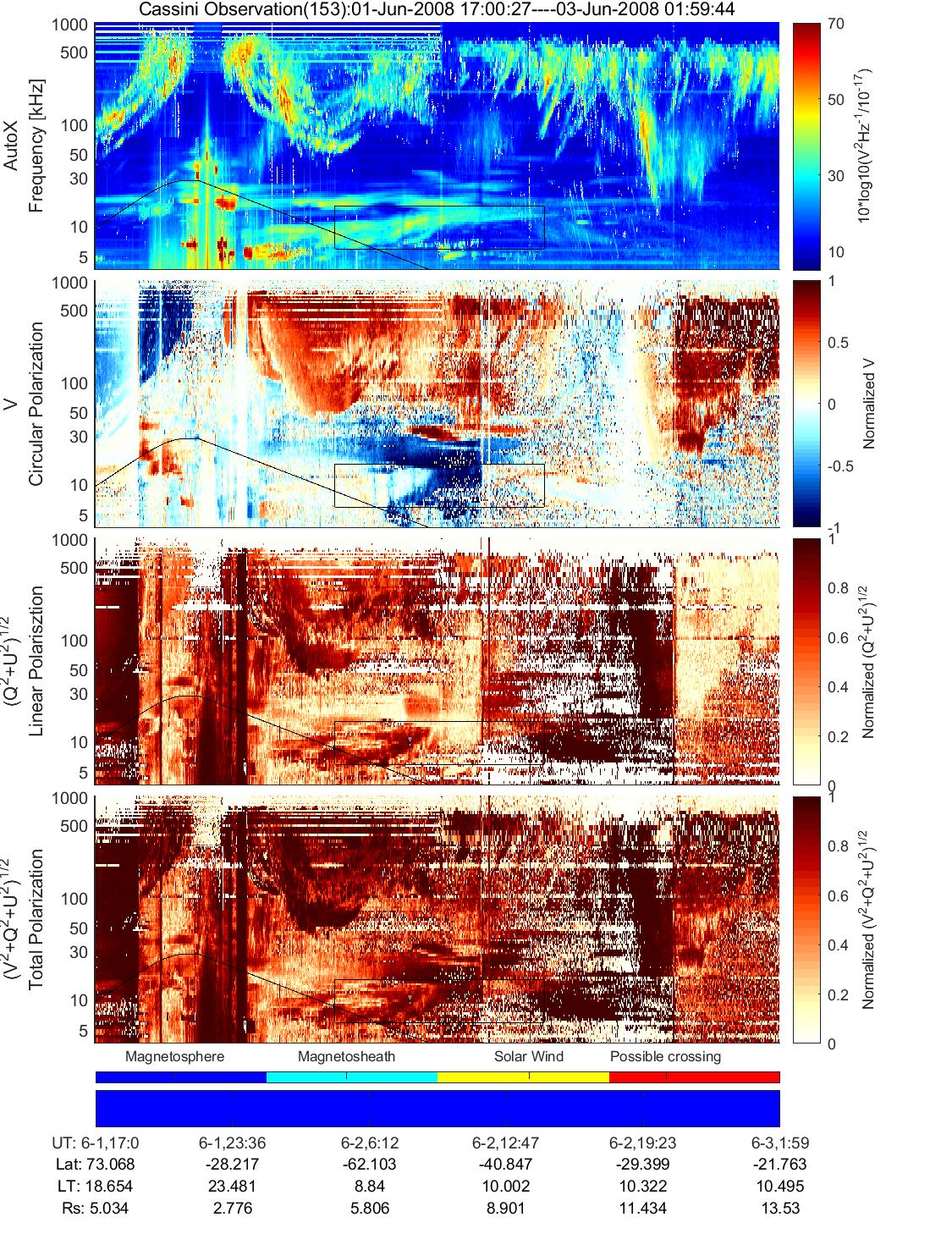


Figure S44, Case 44 of the SAM emission in Table S1. Same format as Figure S1.


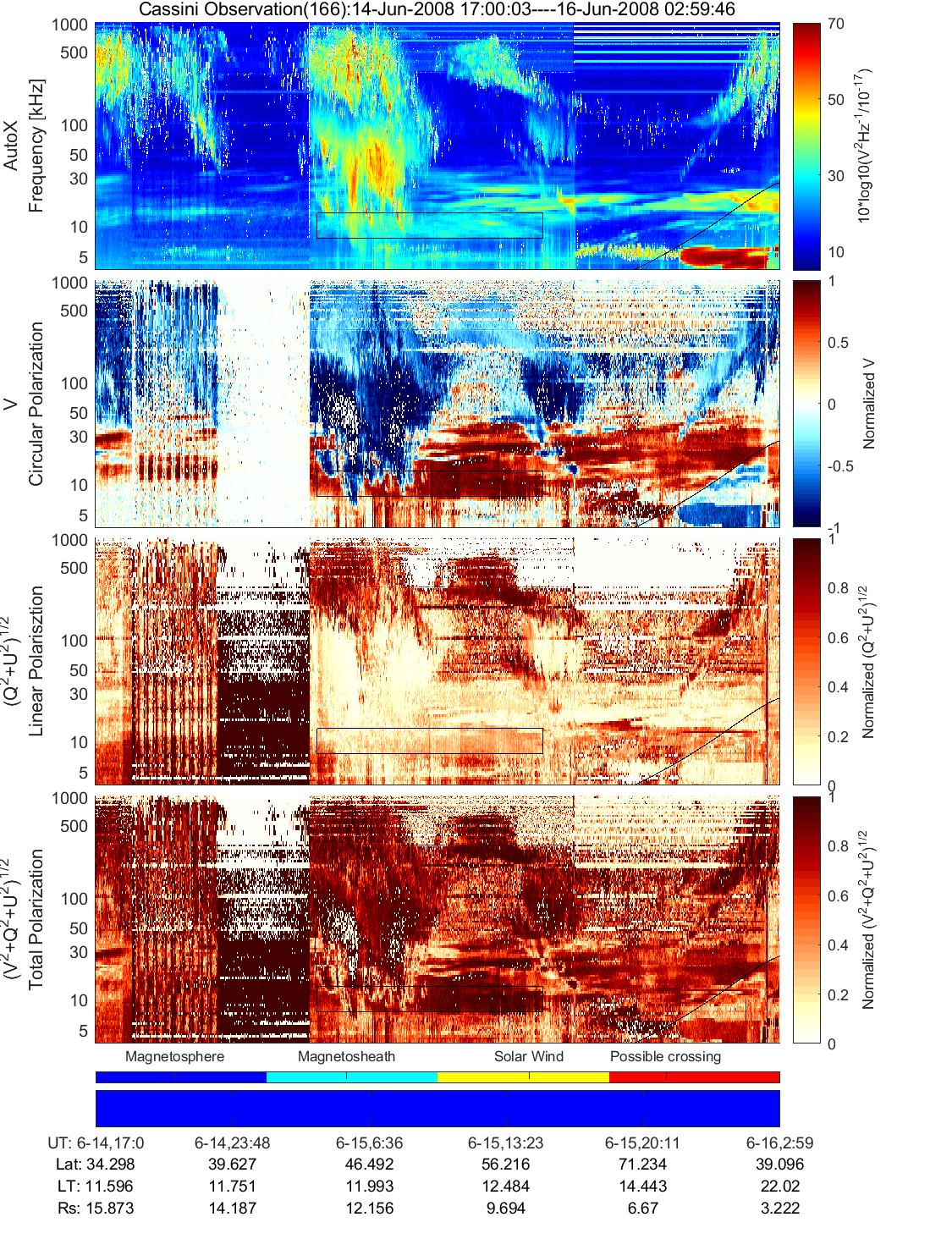


Figure S45, Case 45 of the SAM emission in Table S1. Same format as Figure S1.


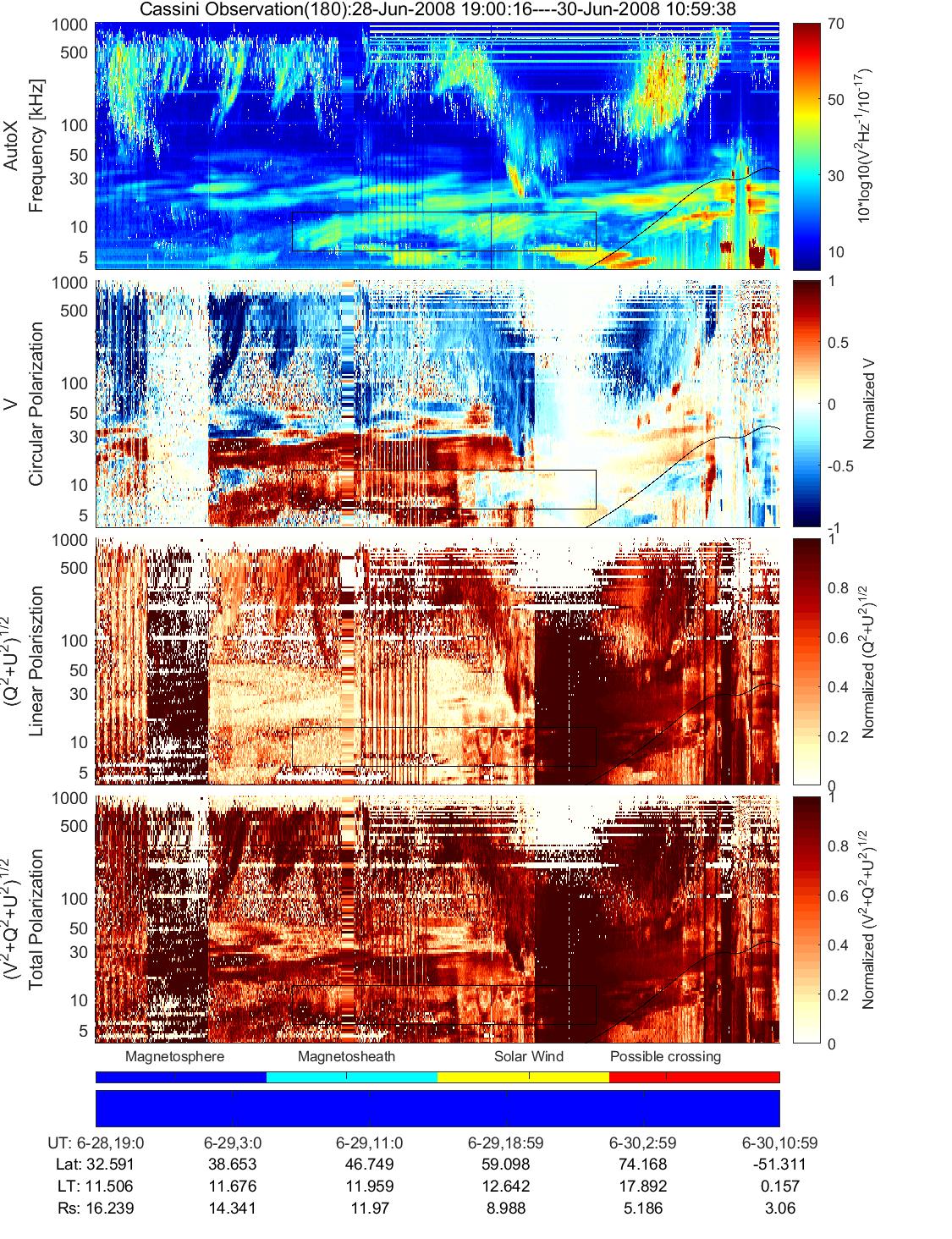


Figure S46, Case 46 of the SAM emission in Table S1. Same format as Figure S1.


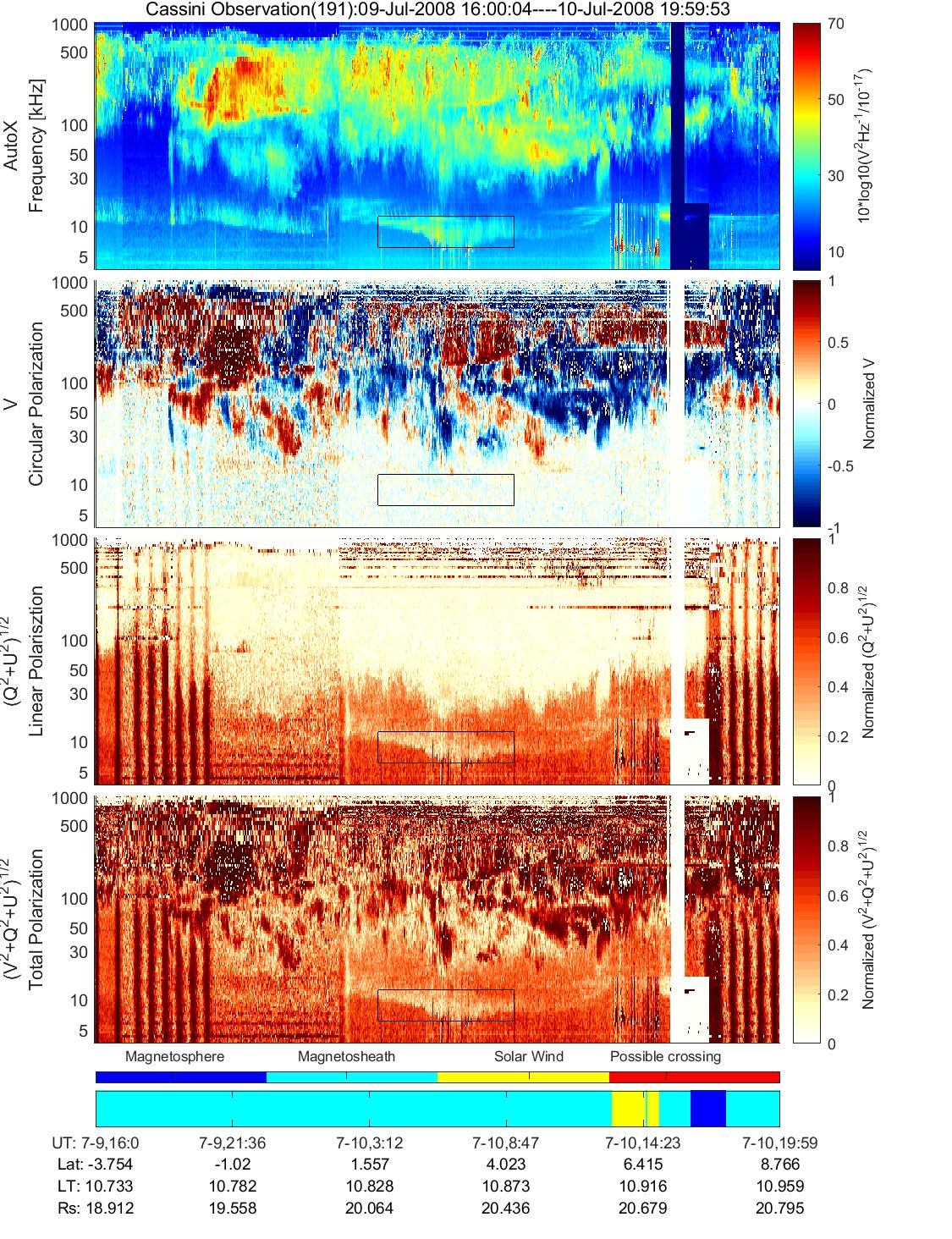


Figure S47, Case 47 of the SAM emission in Table S1. Same format as Figure S1.


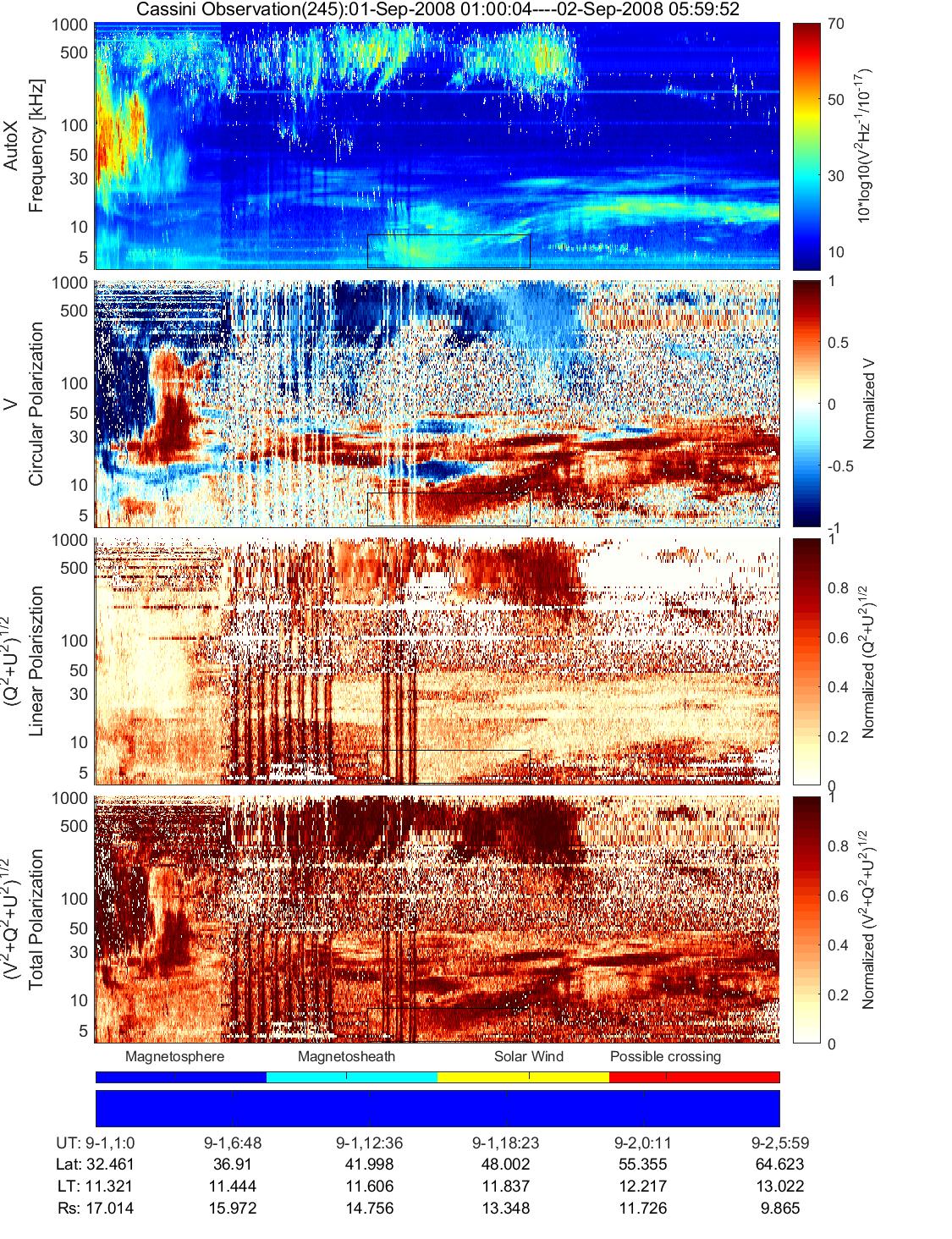


Figure S48, Case 48 of the SAM emission in Table S1. Same format as Figure S1.


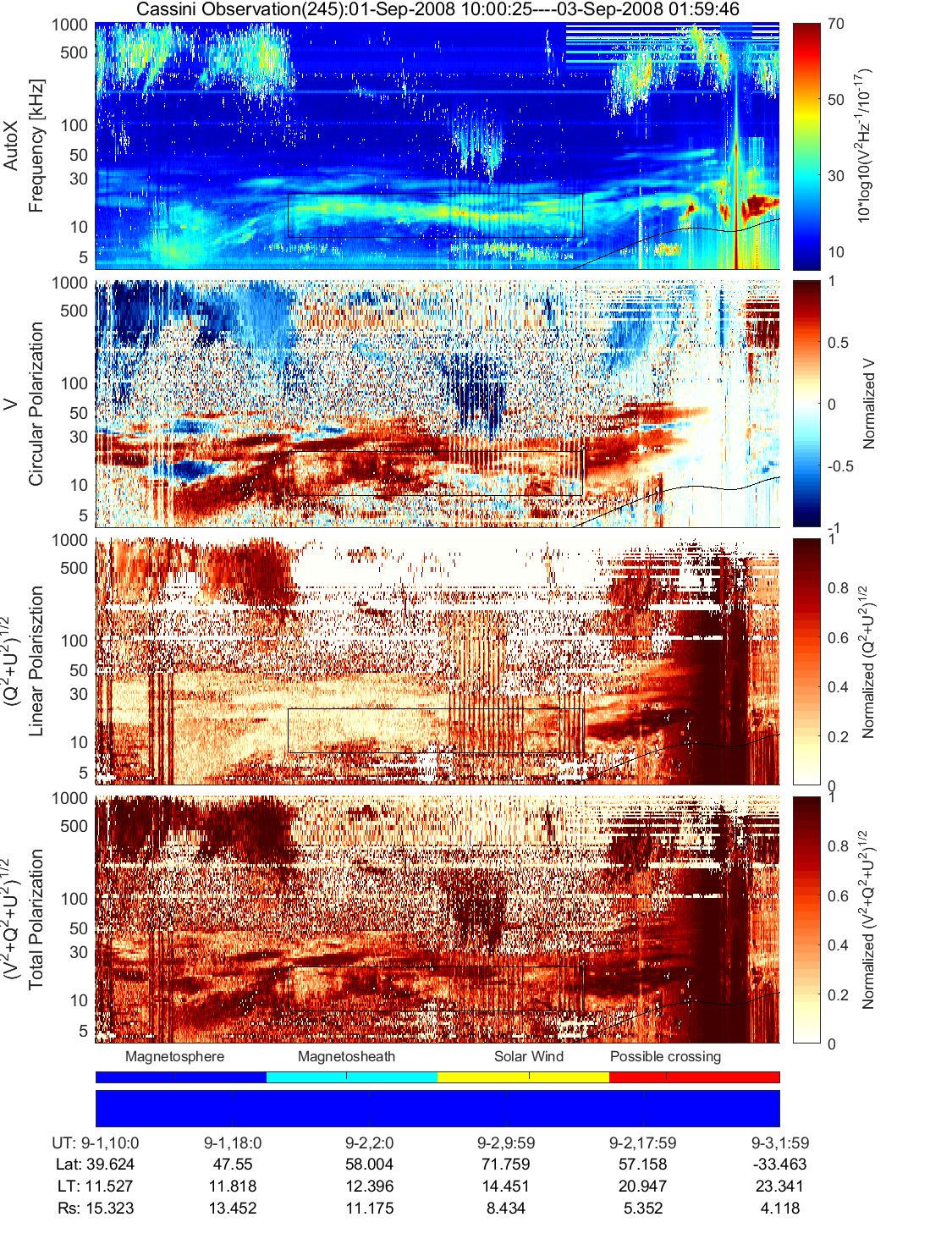


Figure S49, Case 49 of the SAM emission in Table S1. Same format as Figure S1.


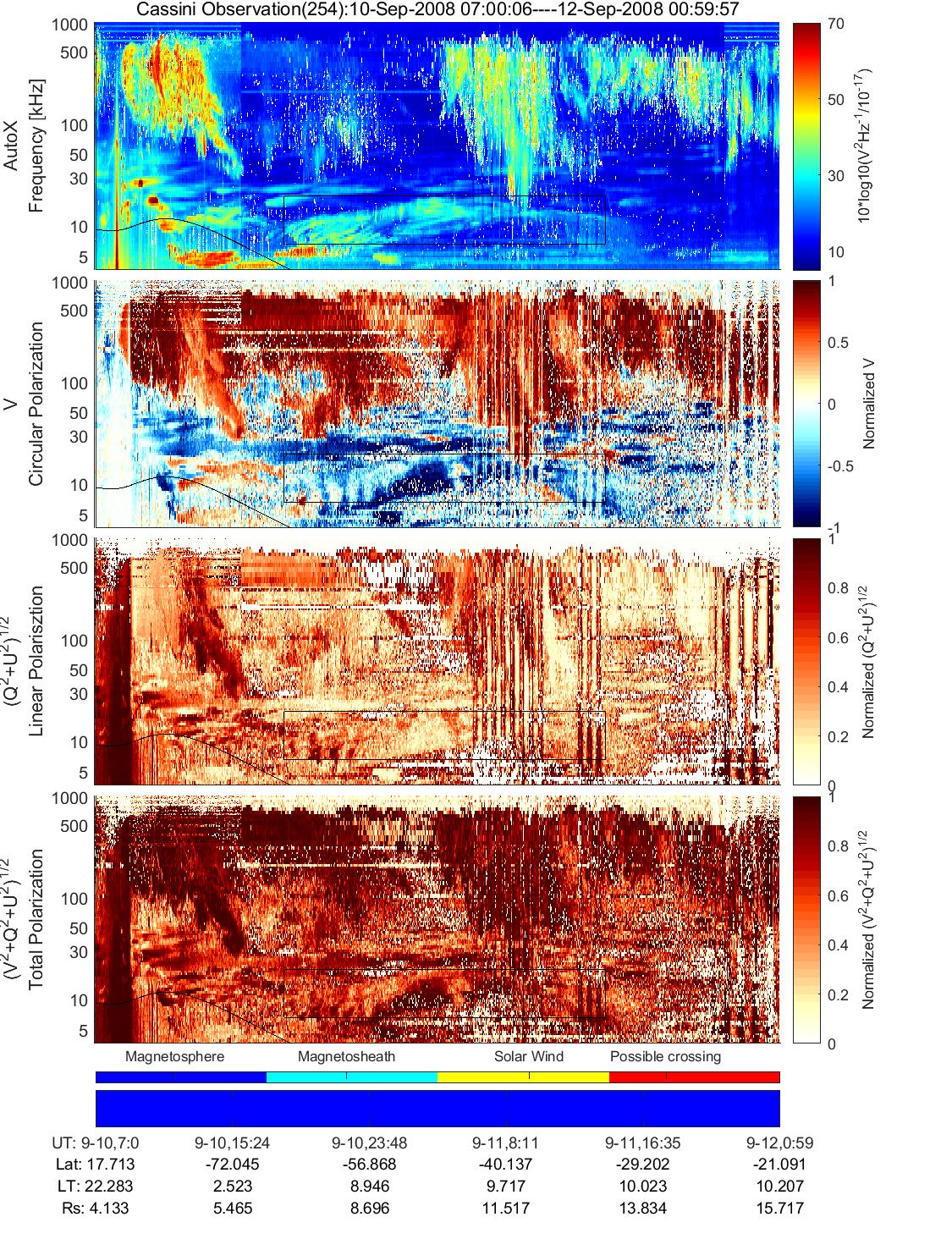
 Figure S50, Case 50 of the SAM emission in Table S1. Same format as Figure S1.


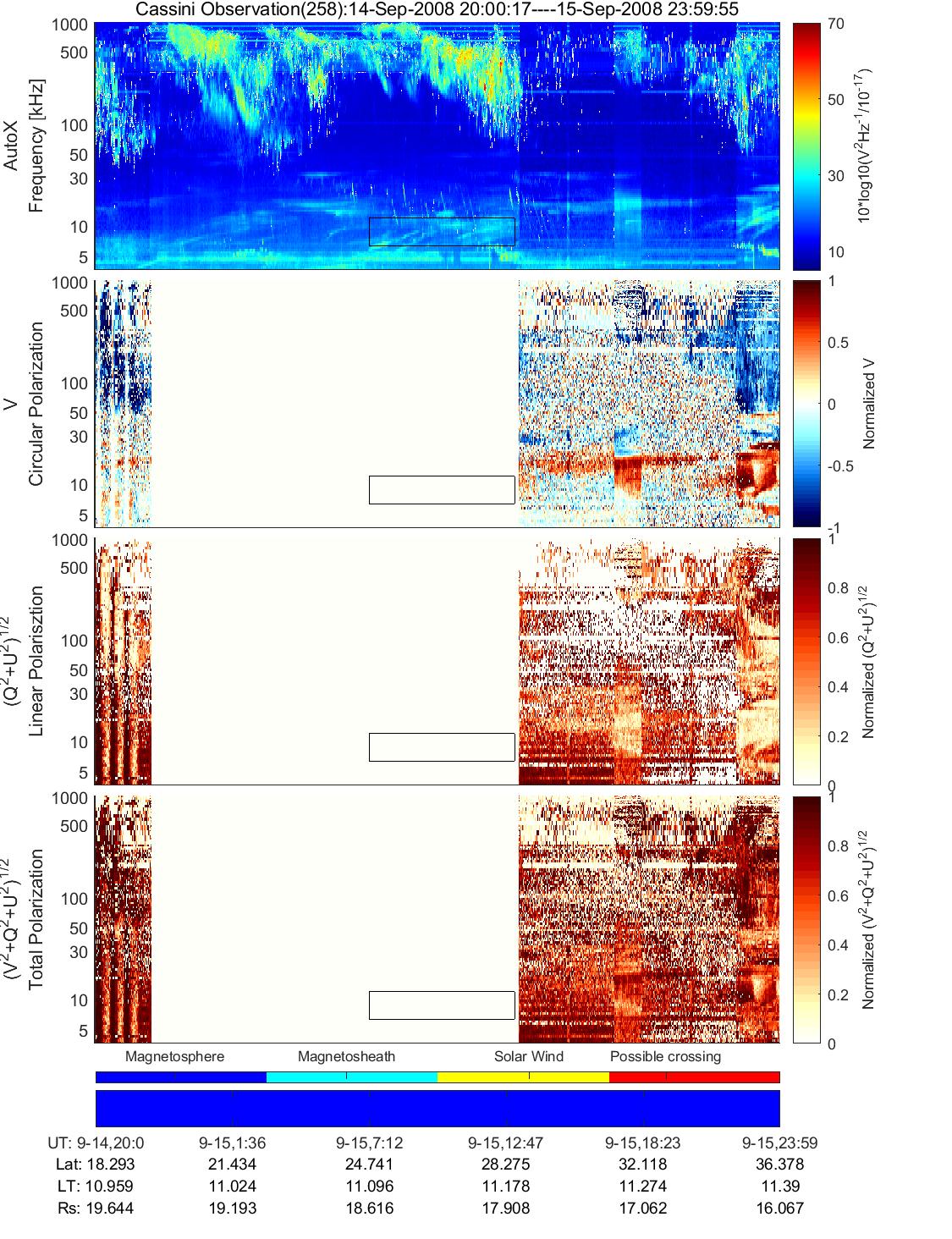
 Figure S51, Case 51 of the SAM emission in Table S1. Same format as Figure S1.


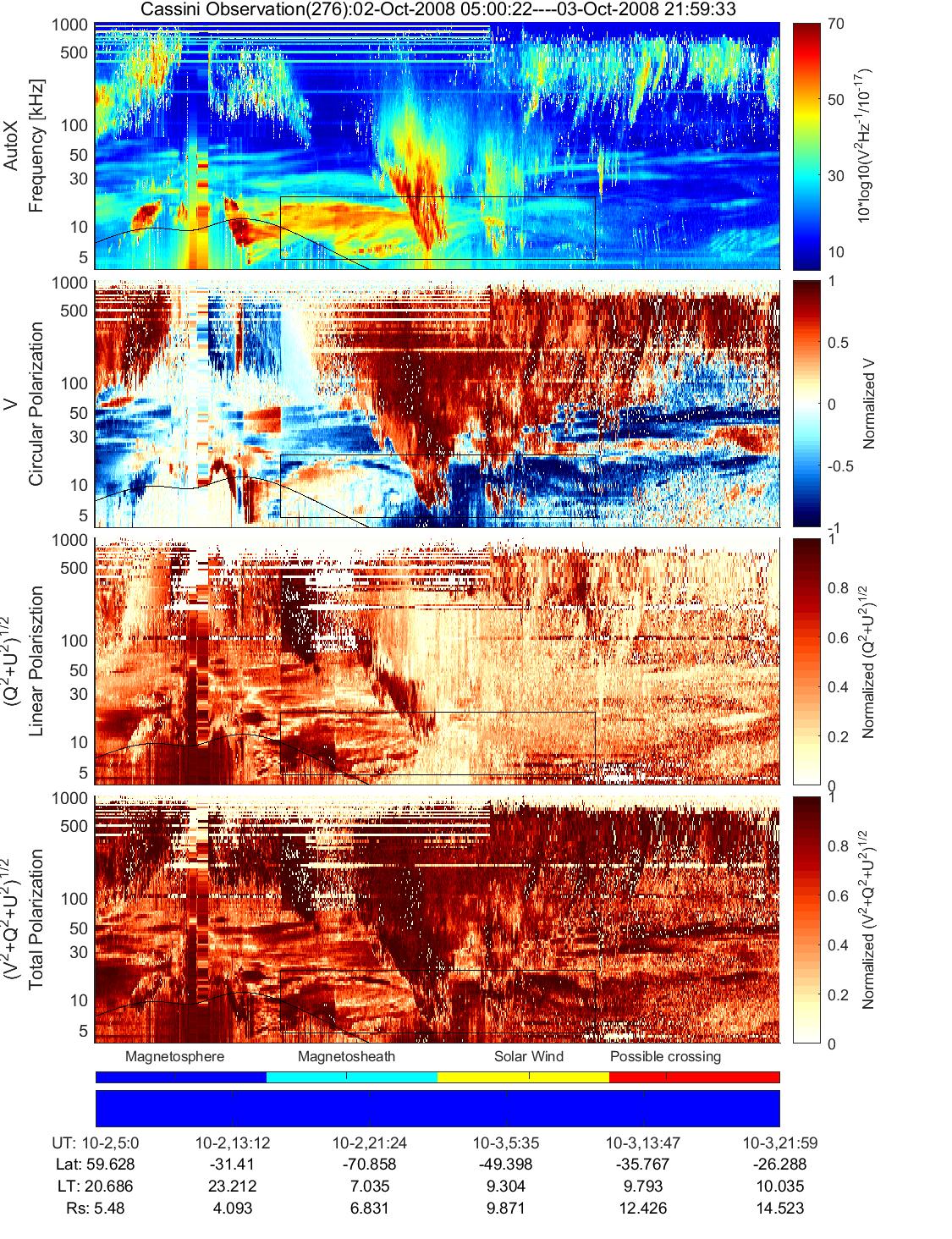
 Figure S52, Case 52 of the SAM emission in Table S1. Same format as Figure S1.


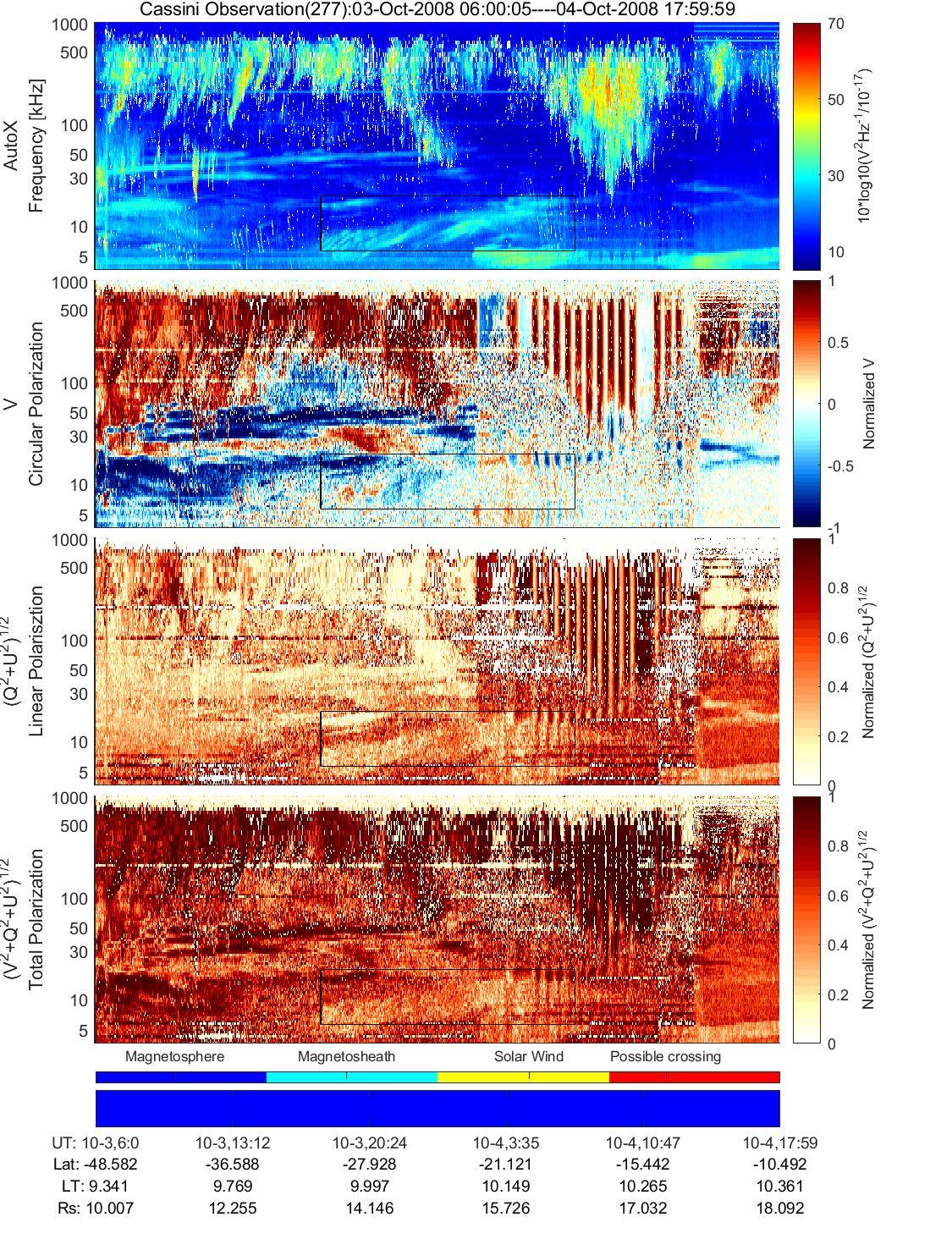
 Figure S53, Case 53 of the SAM emission in Table S1. Same format as Figure S1.


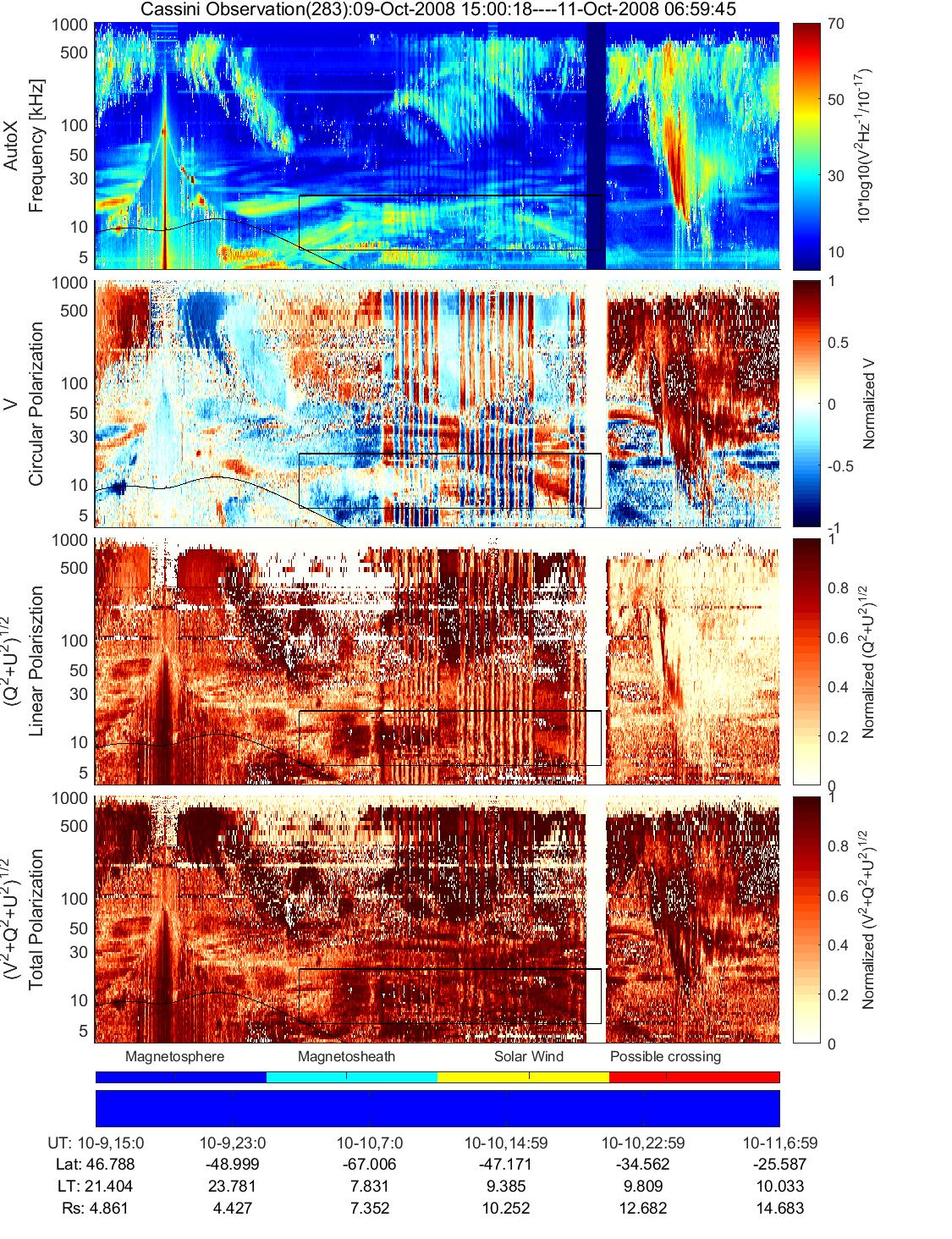
 Figure S54, Case 54 of the SAM emission in Table S1. Same format as Figure S1.


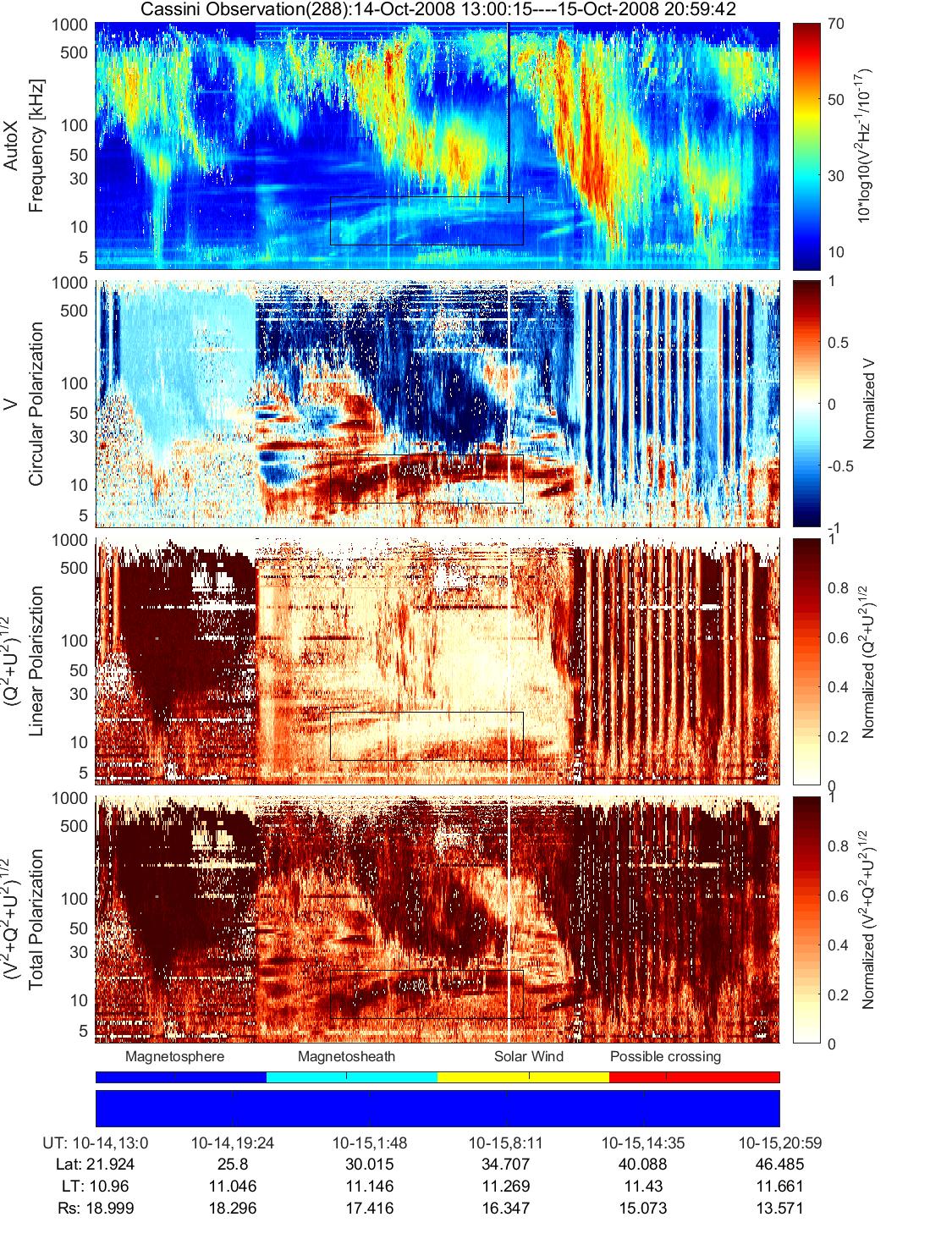
 Figure S55, Case 55 of the SAM emission in Table S1. Same format as Figure S1.


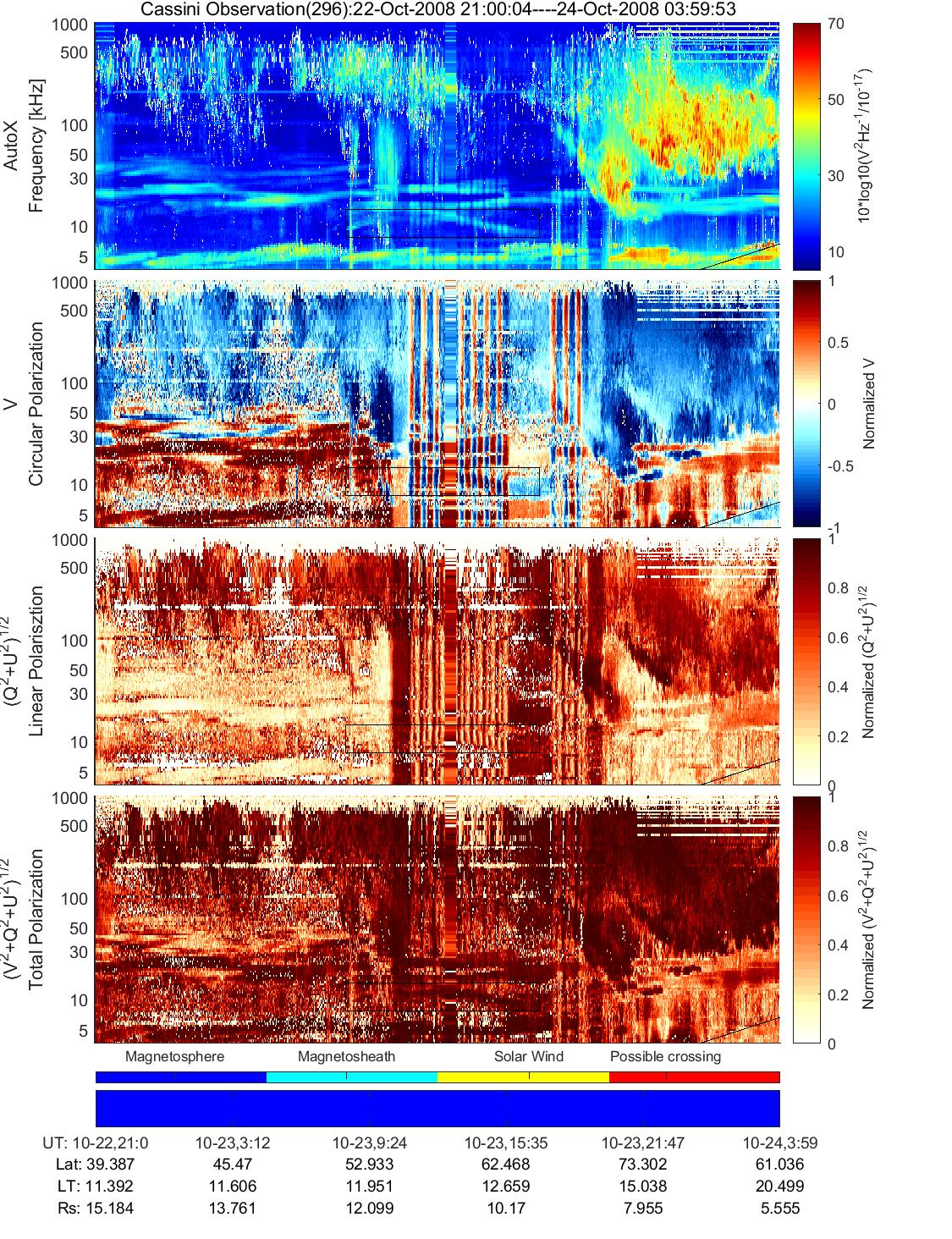
 Figure S56, Case 56 of the SAM emission in Table S1. Same format as Figure S1.


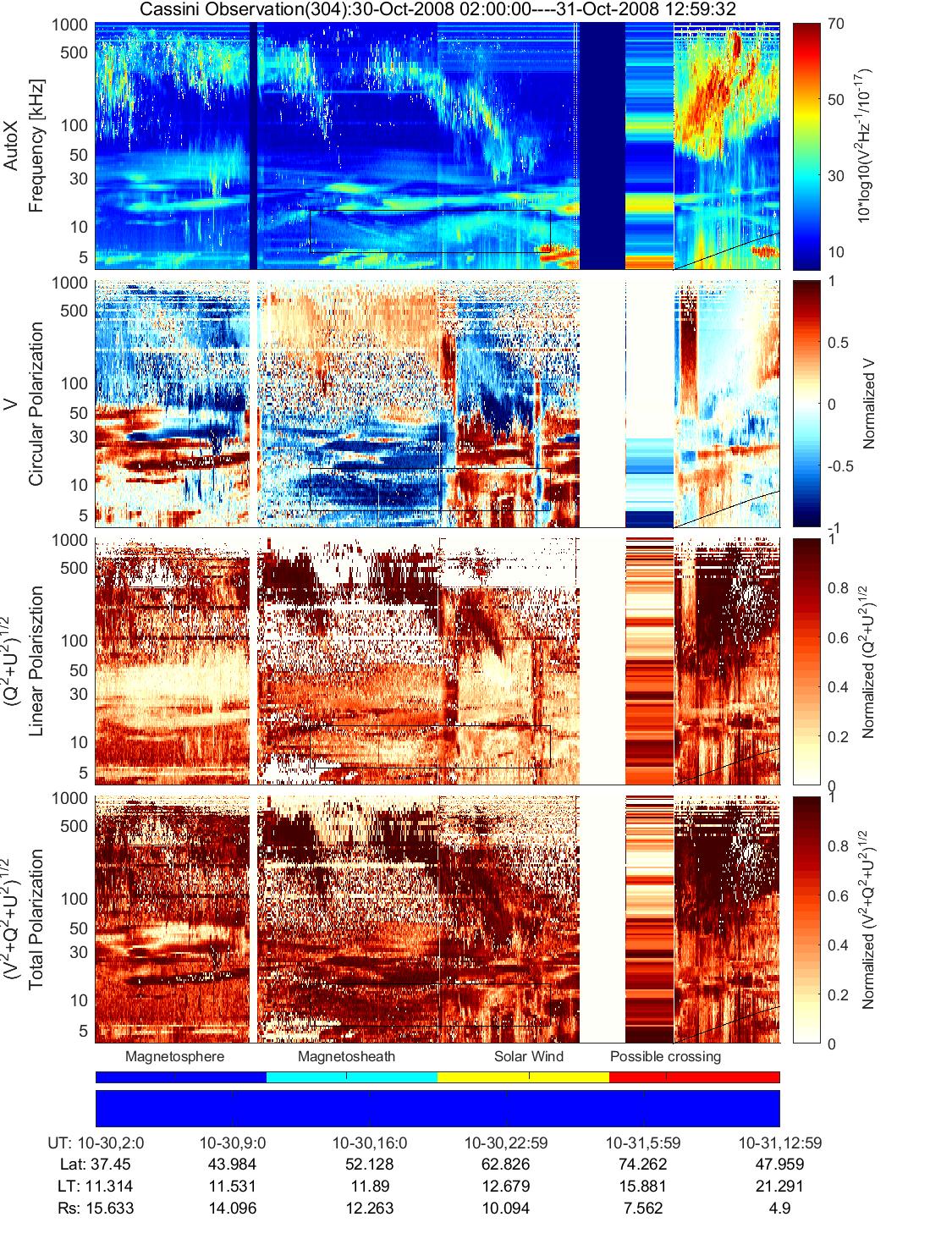
 Figure S57, Case 57 of the SAM emission in Table S1. Same format as Figure S1.


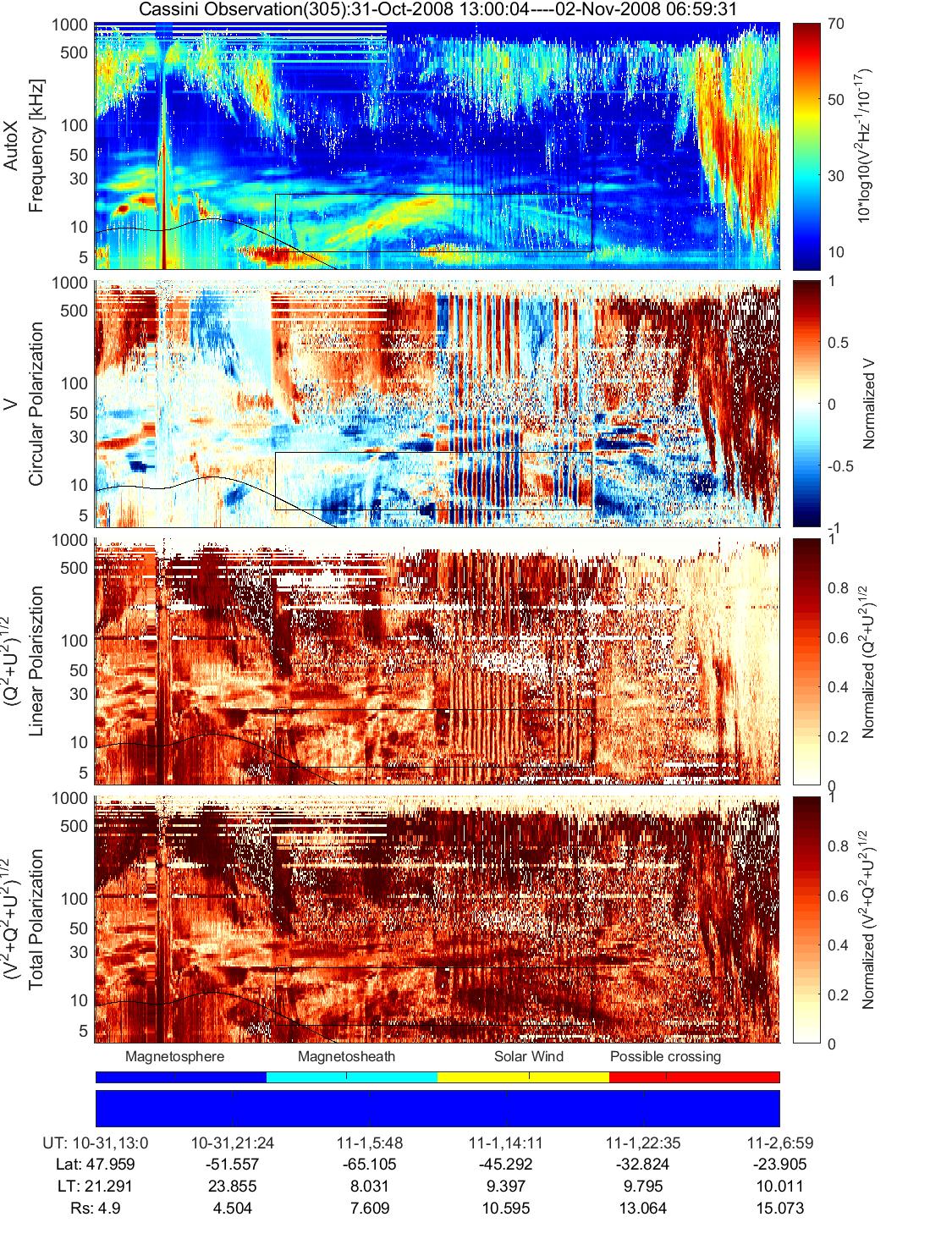
 Figure S58, Case 58 of the SAM emission in Table S1. Same format as Figure S1.


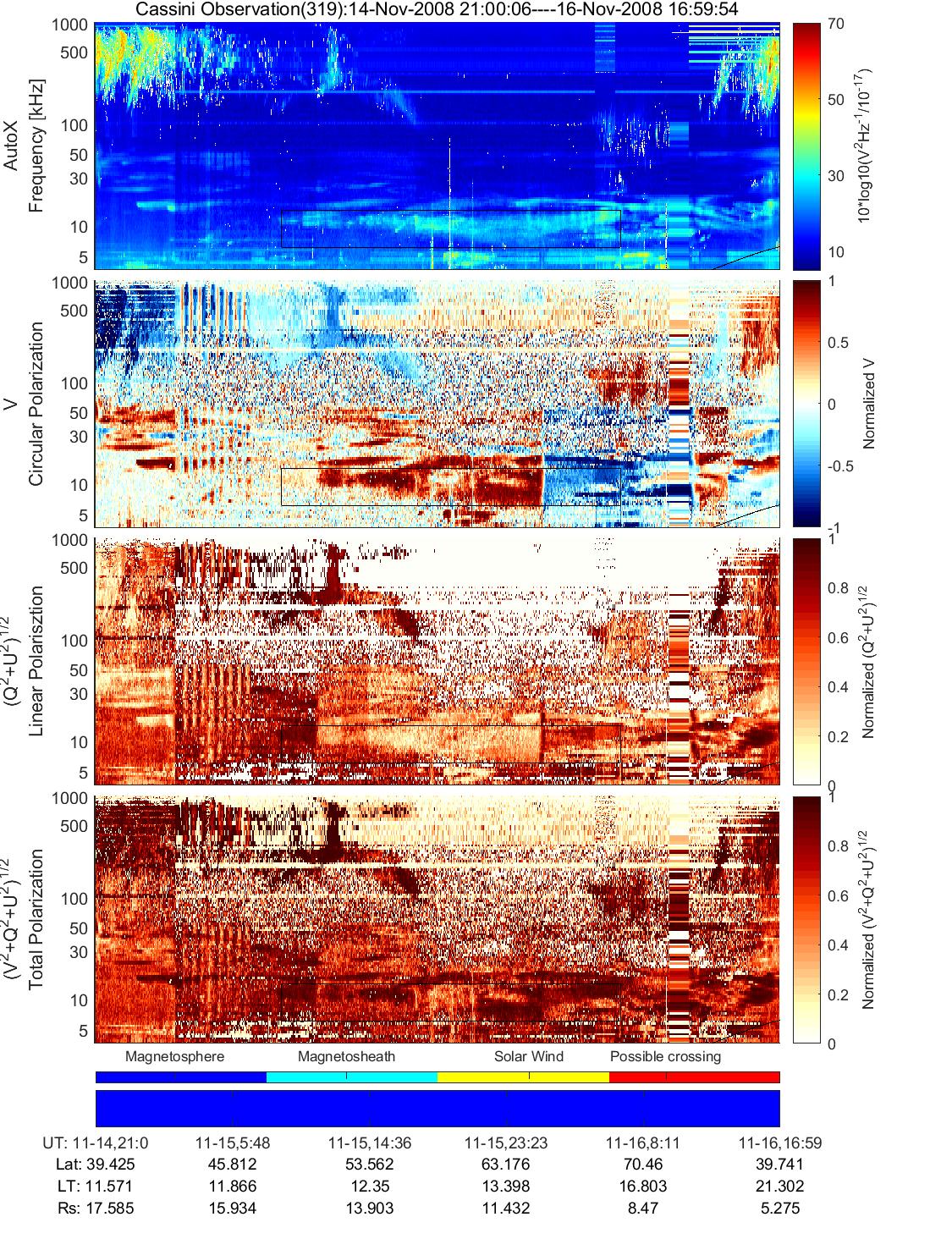
 Figure S59, Case 59 of the SAM emission in Table S1. Same format as Figure S1.


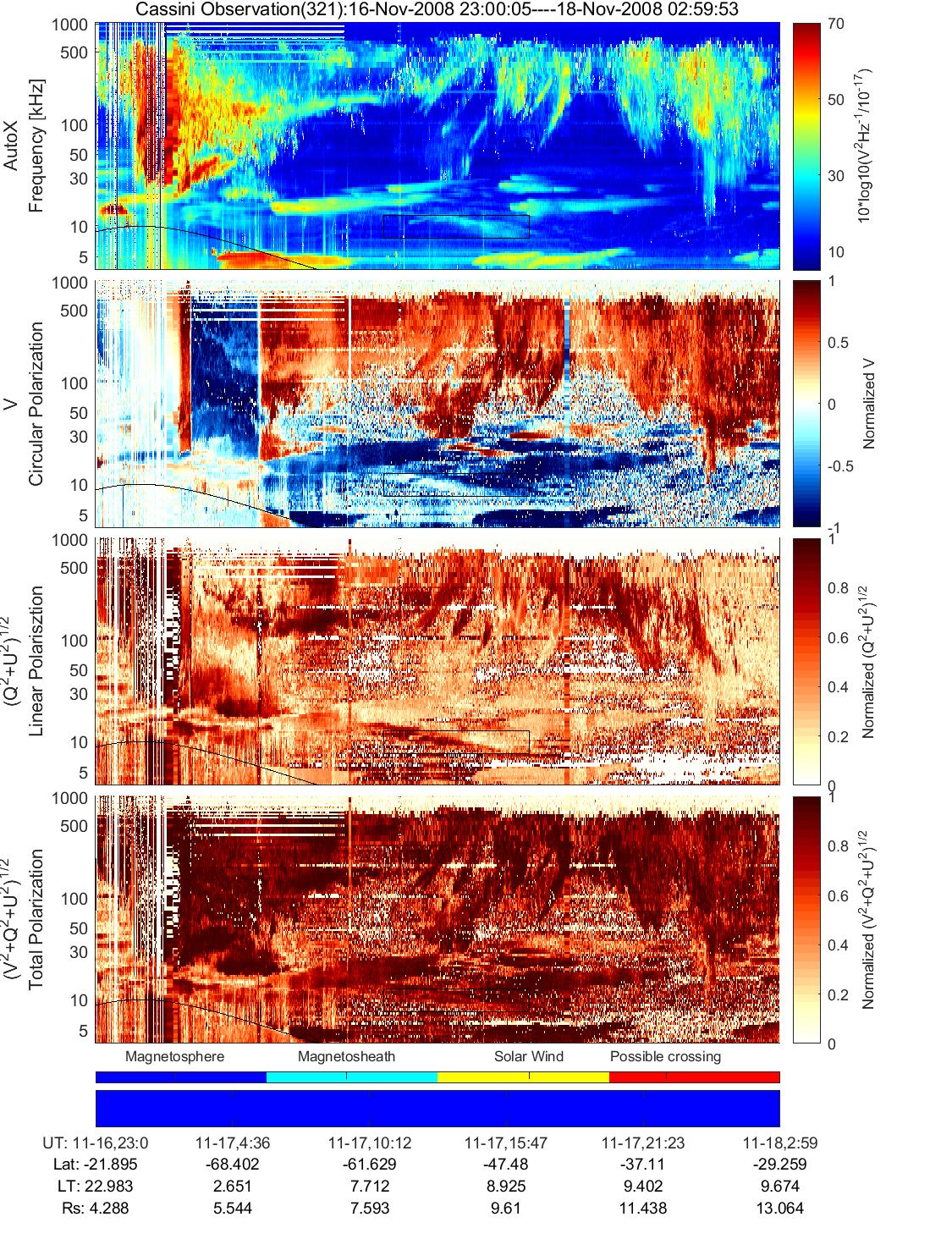
 Figure S60, Case 60 of the SAM emission in Table S1. Same format as Figure S1.


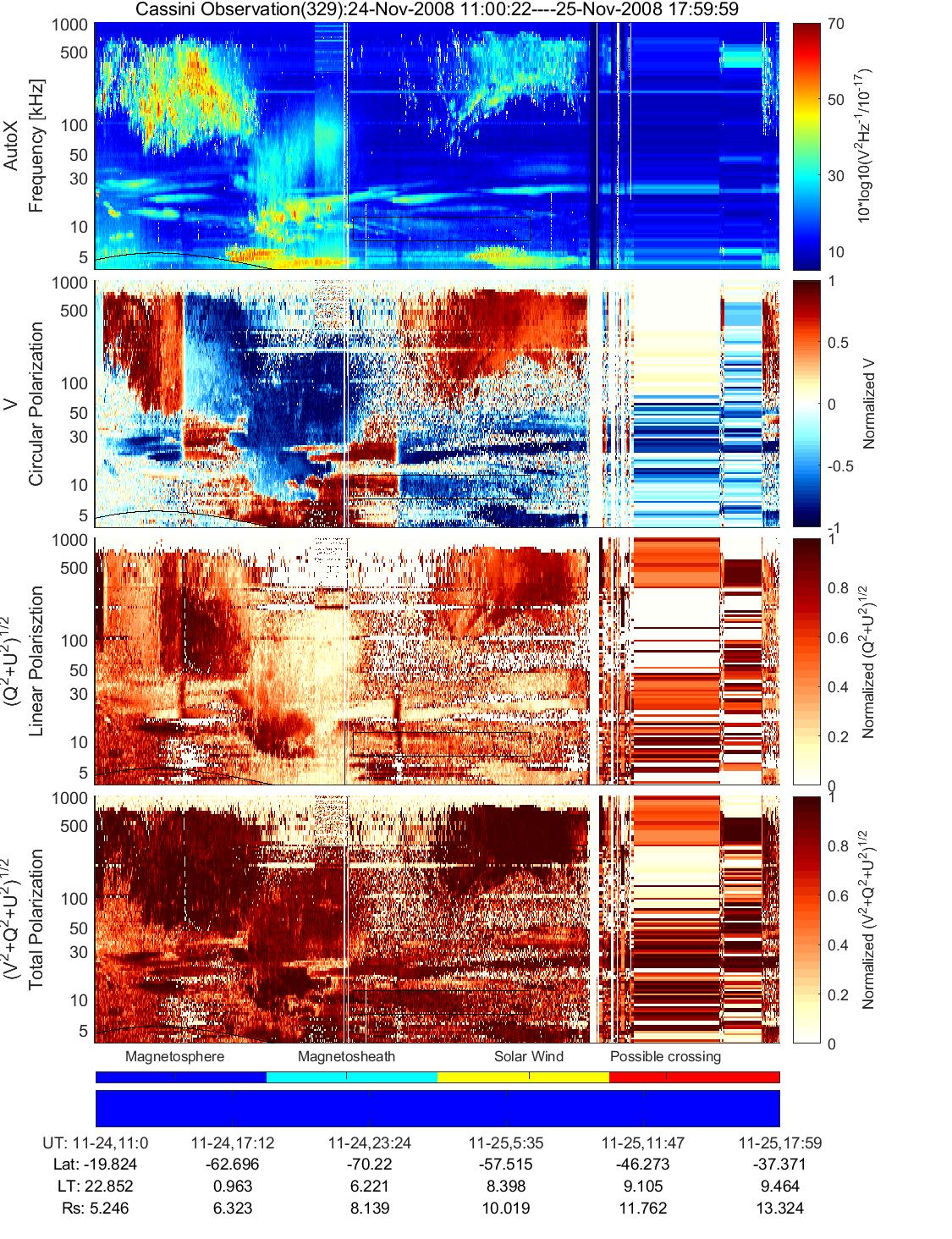
 Figure S61, Case 61 of the SAM emission in Table S1. Same format as Figure S1.


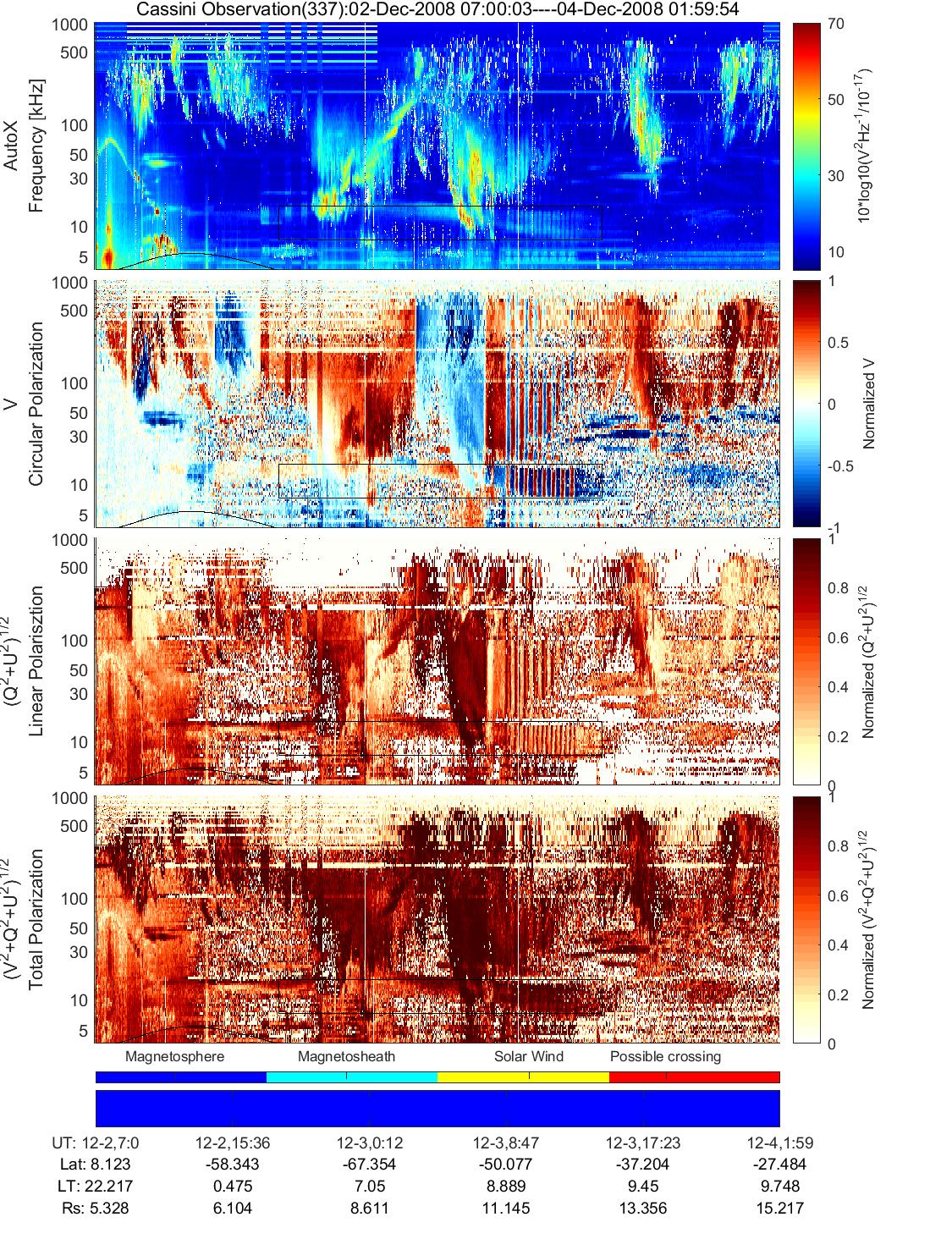
 Figure S62, Case 62 of the SAM emission in Table S1. Same format as Figure S1.


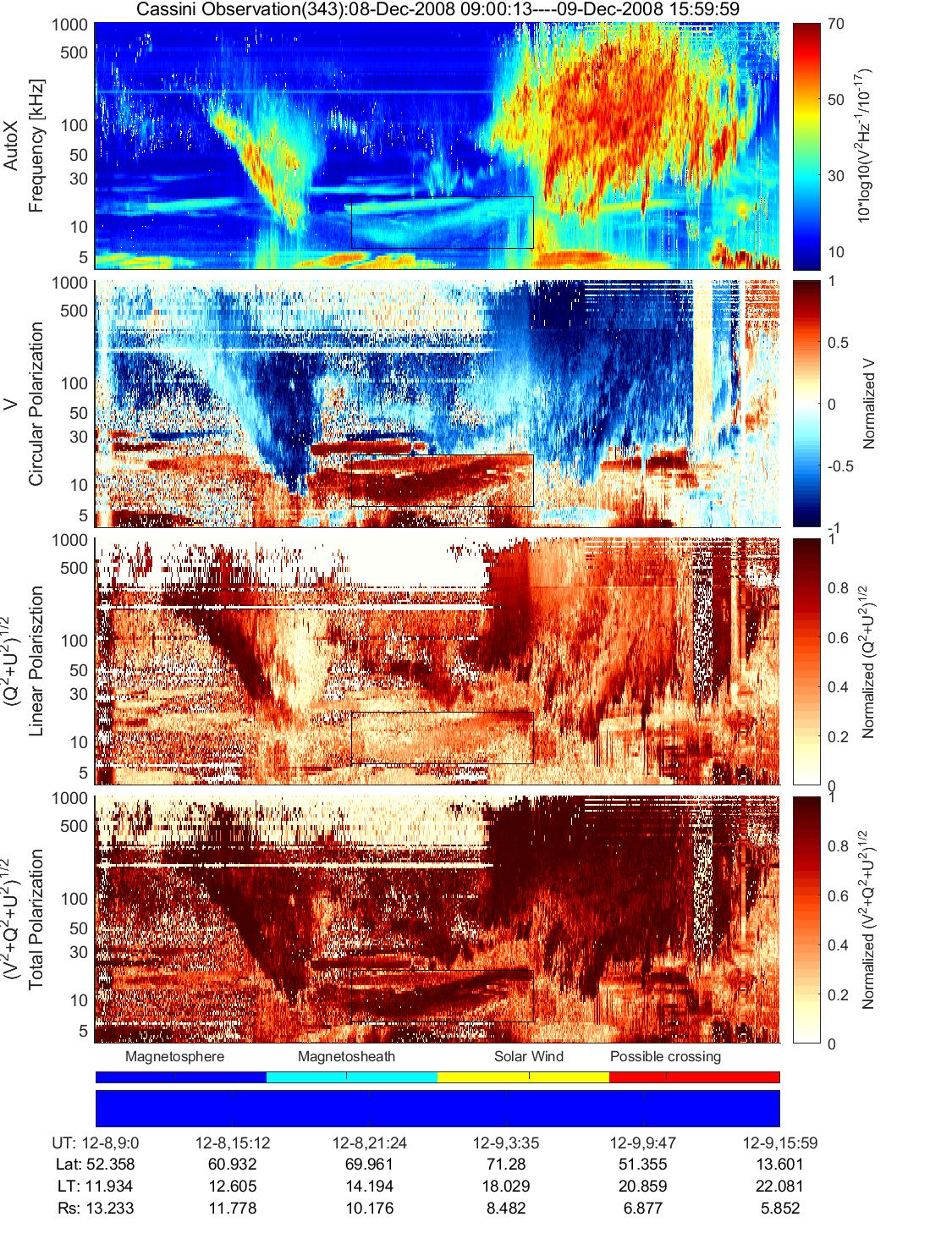
 Figure S63, Case 63 of the SAM emission in Table S1. Same format as Figure S1.


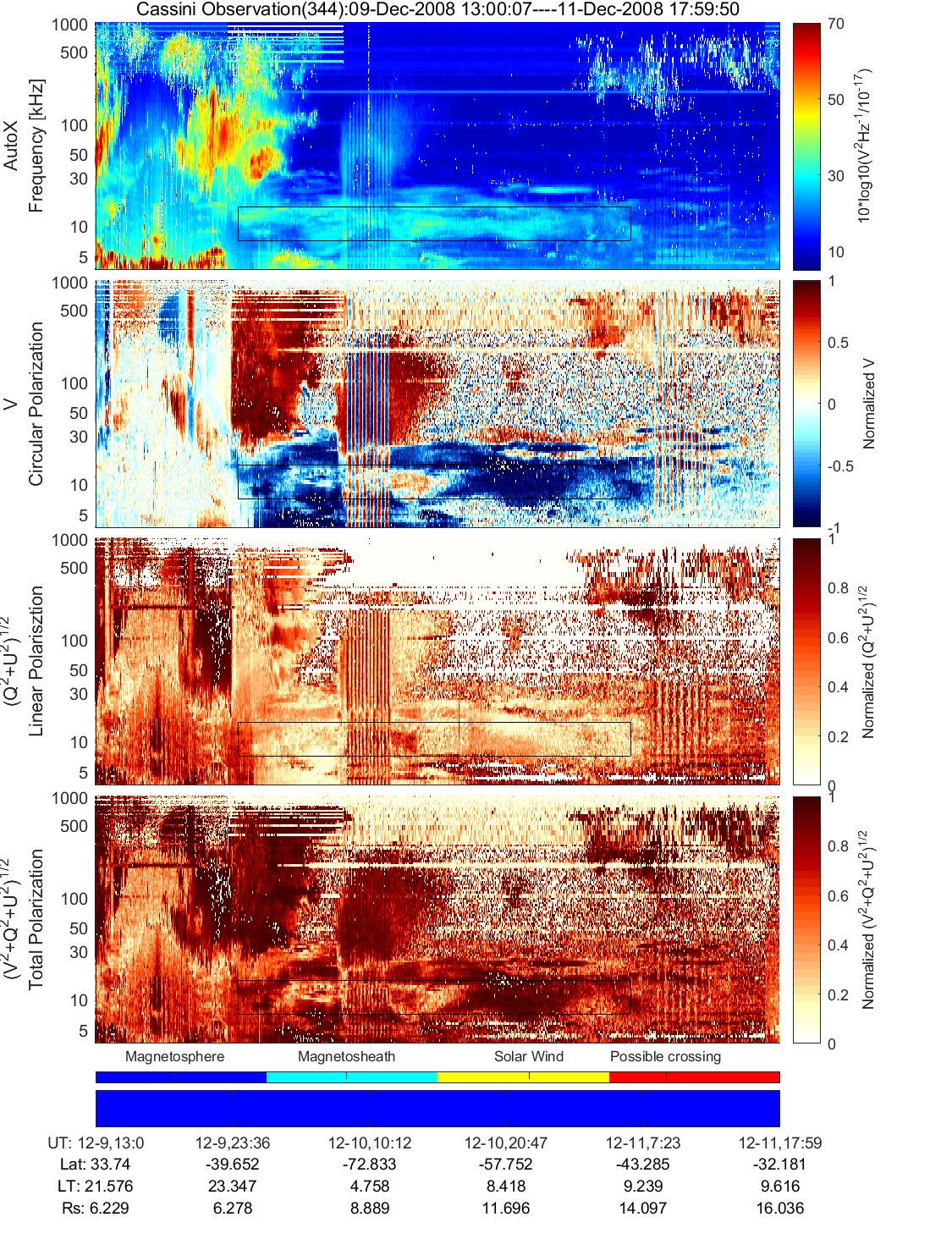
 Figure S64, Case 64 of the SAM emission in Table S1. Same format as Figure S1.


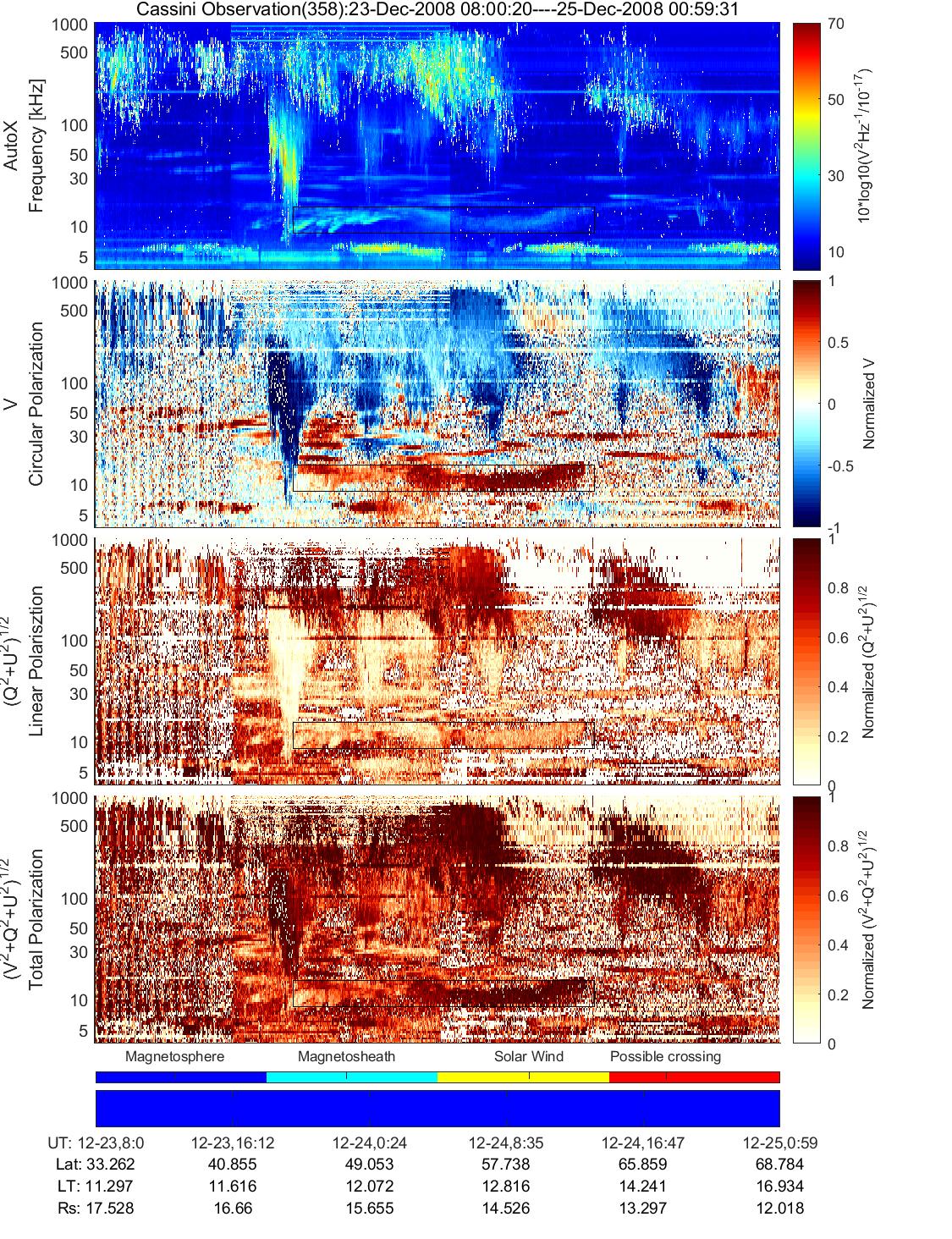
 Figure S65, Case 65 of the SAM emission in Table S1. Same format as Figure S1.


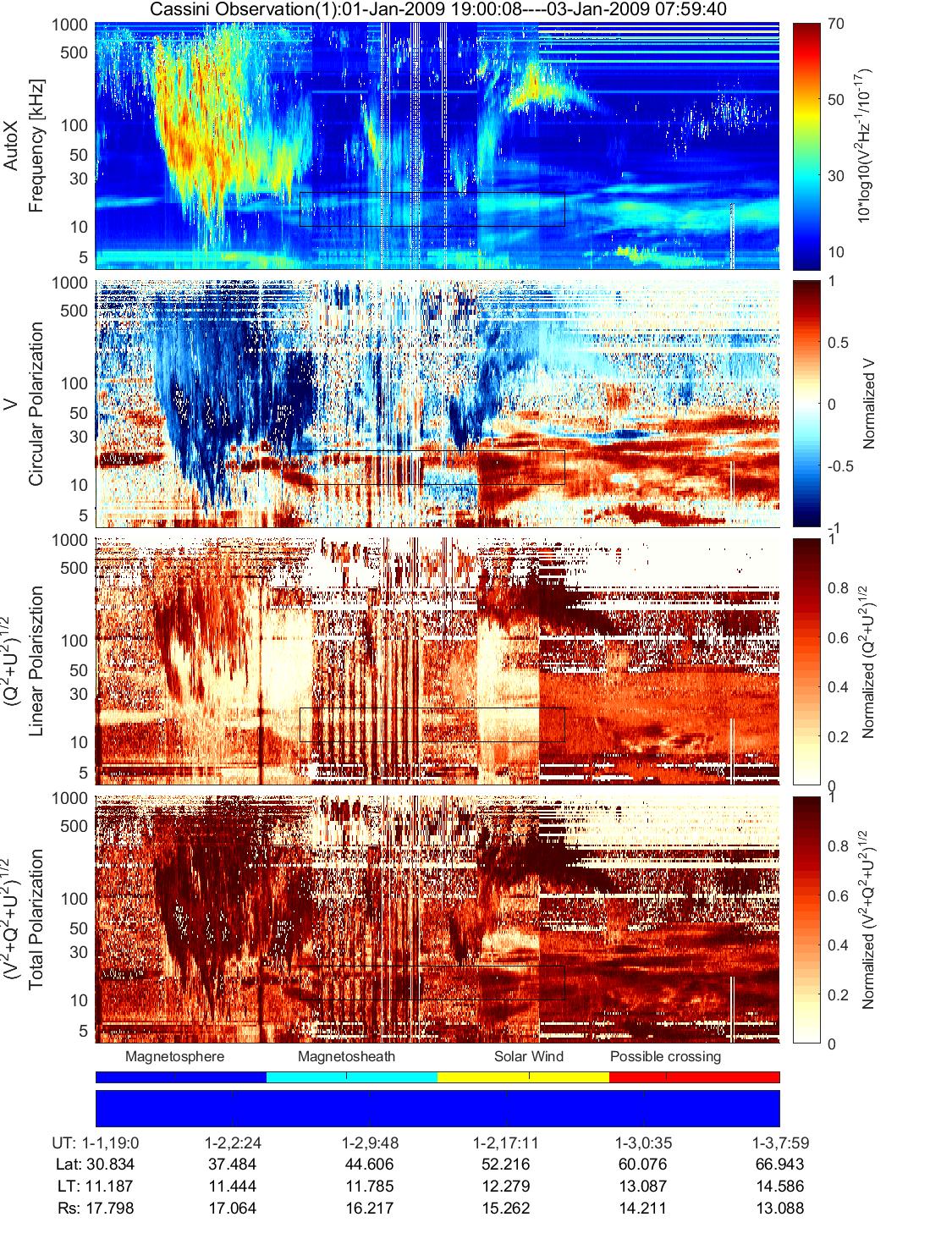
 Figure S66, Case 66 of the SAM emission in Table S1. Same format as Figure S1.


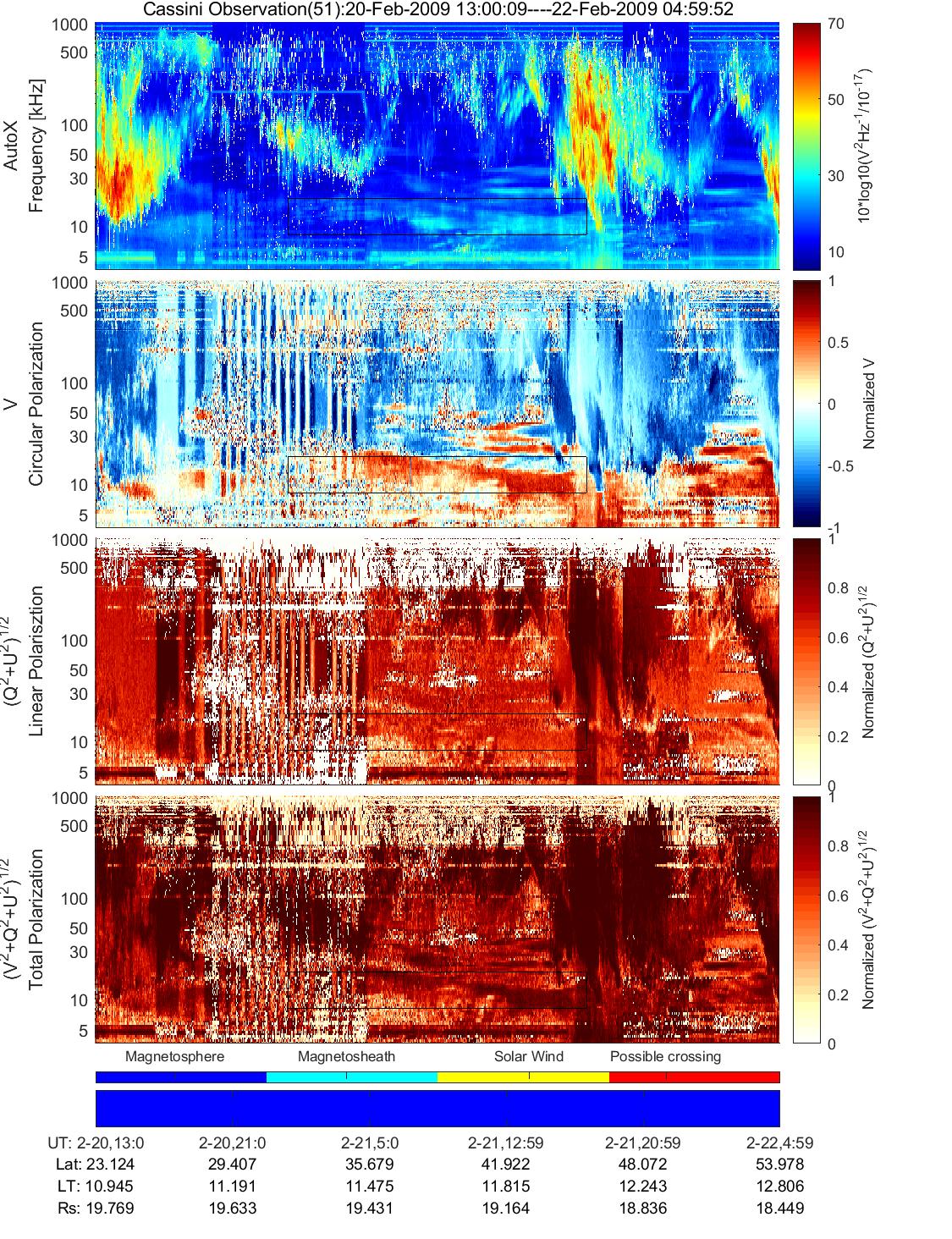
 Figure S67, Case 67 of the SAM emission in Table S1. Same format as Figure S1.


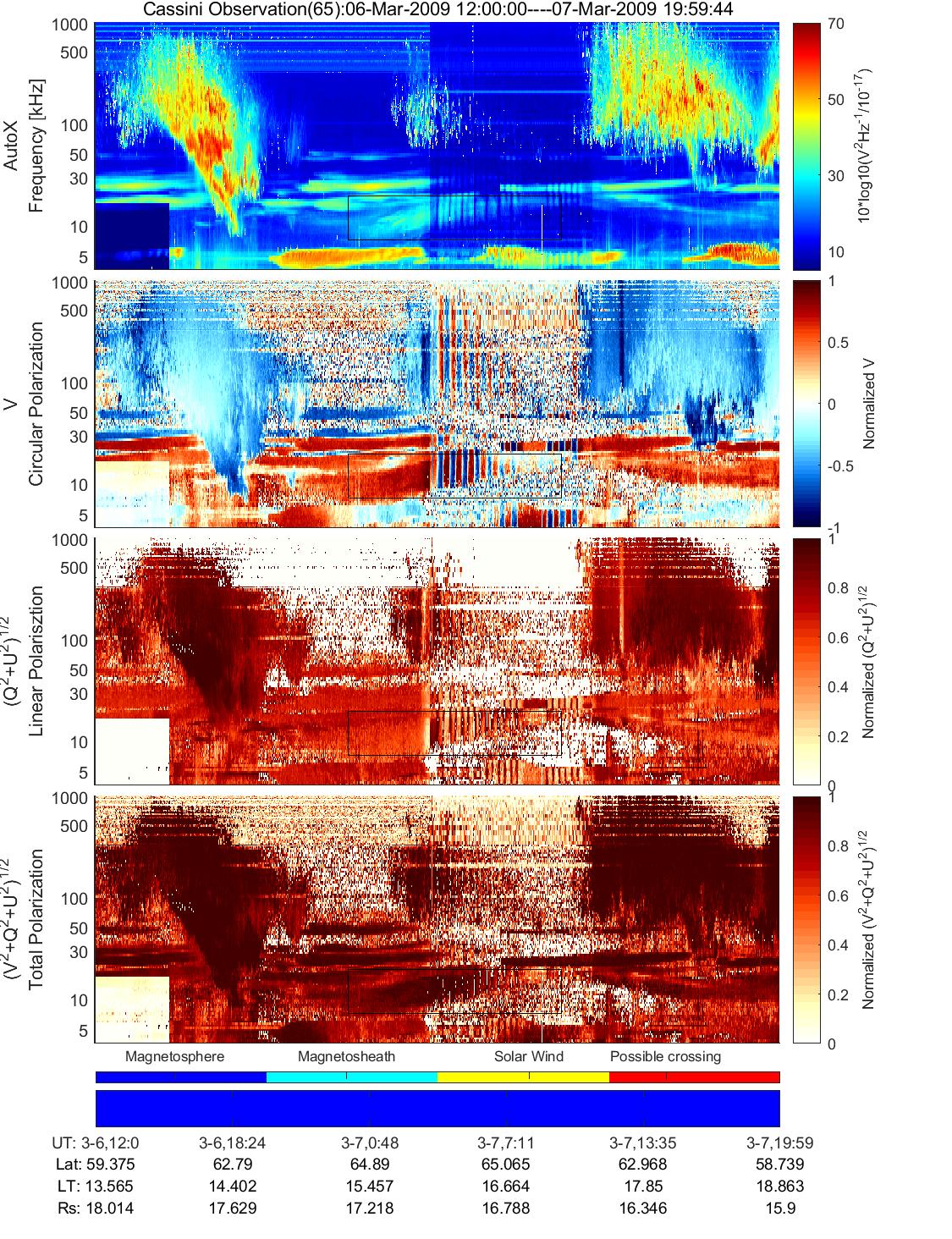
 Figure S68, Case 68 of the SAM emission in Table S1. Same format as Figure S1.


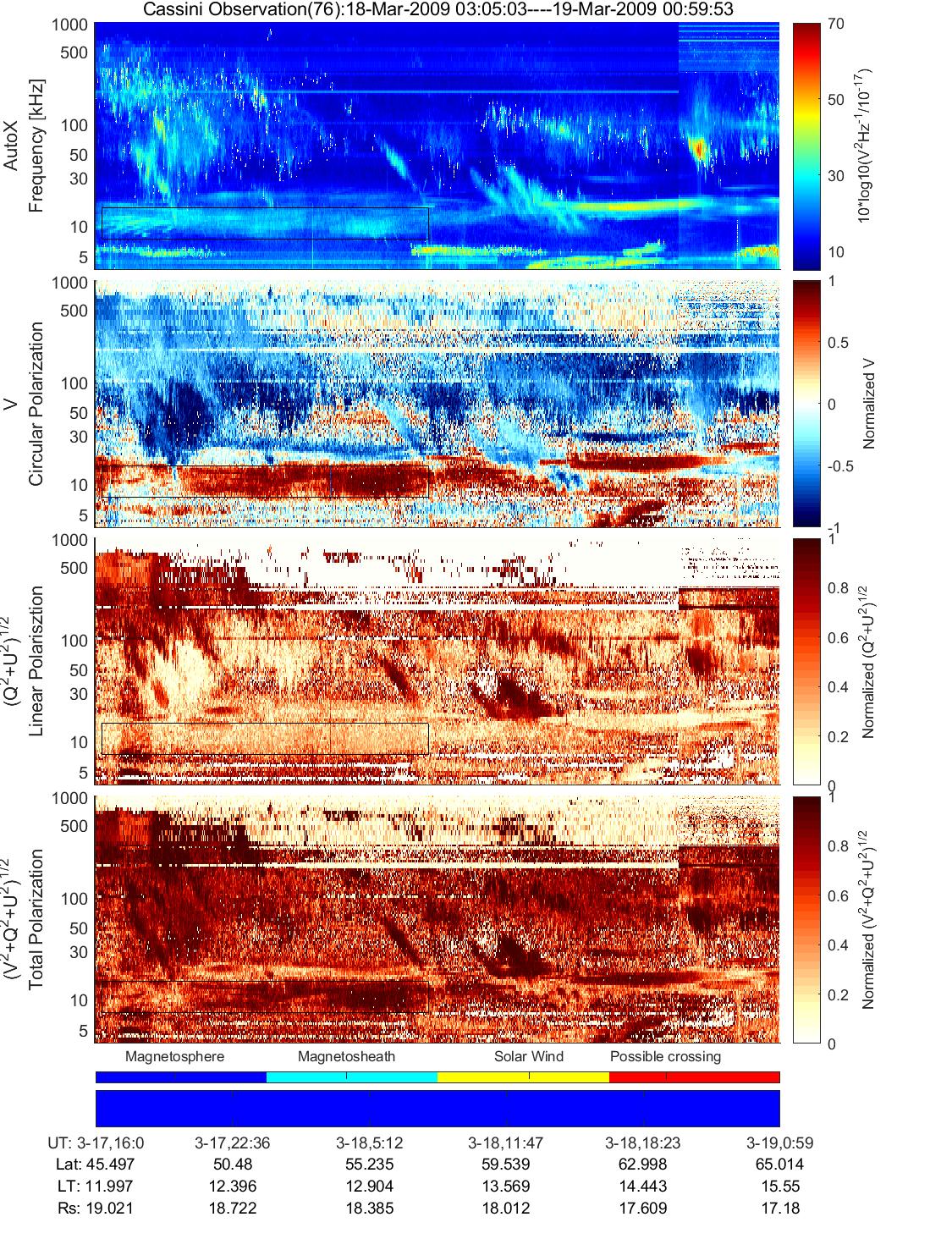
 Figure S69, Case 69 of the SAM emission in Table S1. Same format as Figure S1.


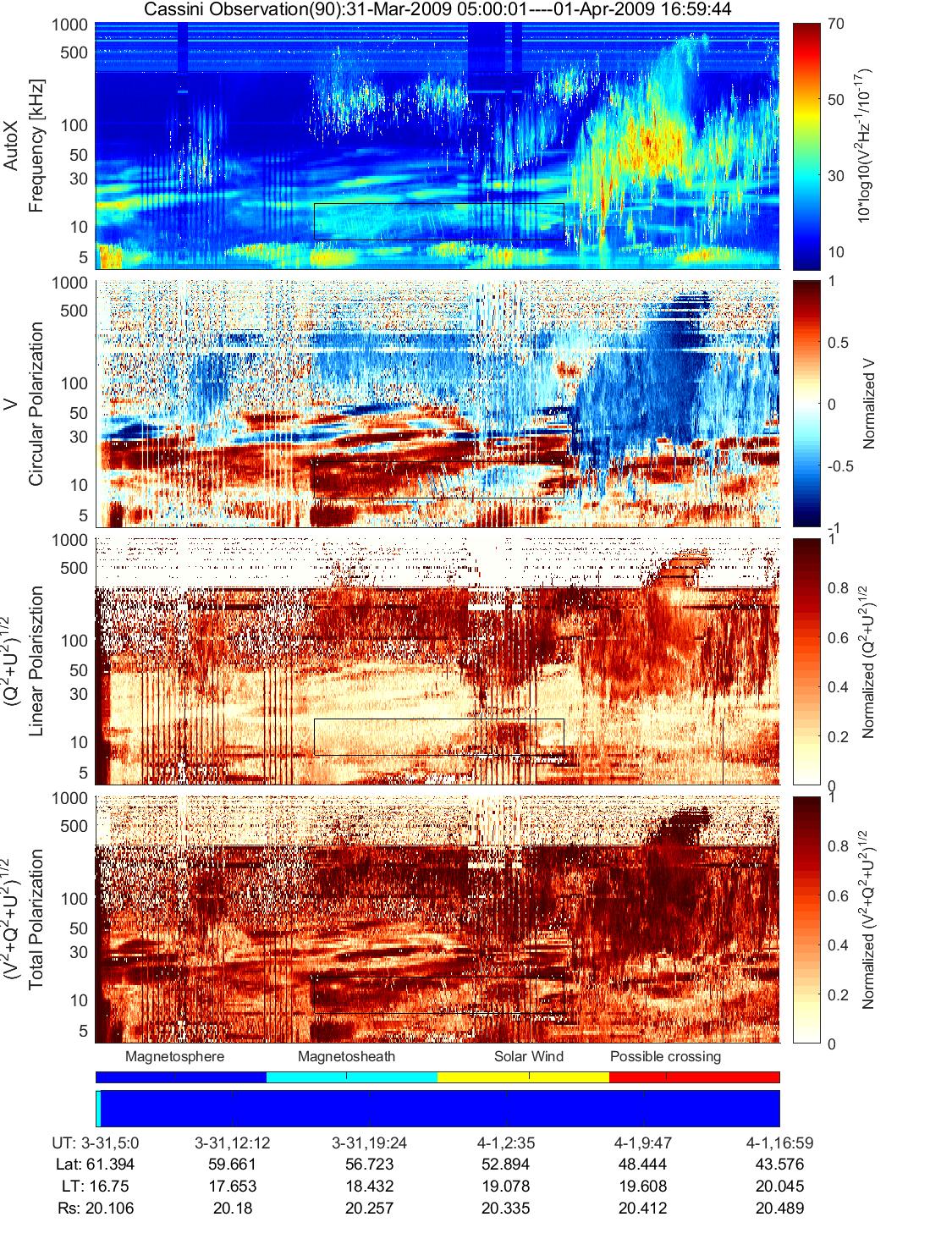
 Figure S70, Case 70 of the SAM emission in Table S1. Same format as Figure S1.


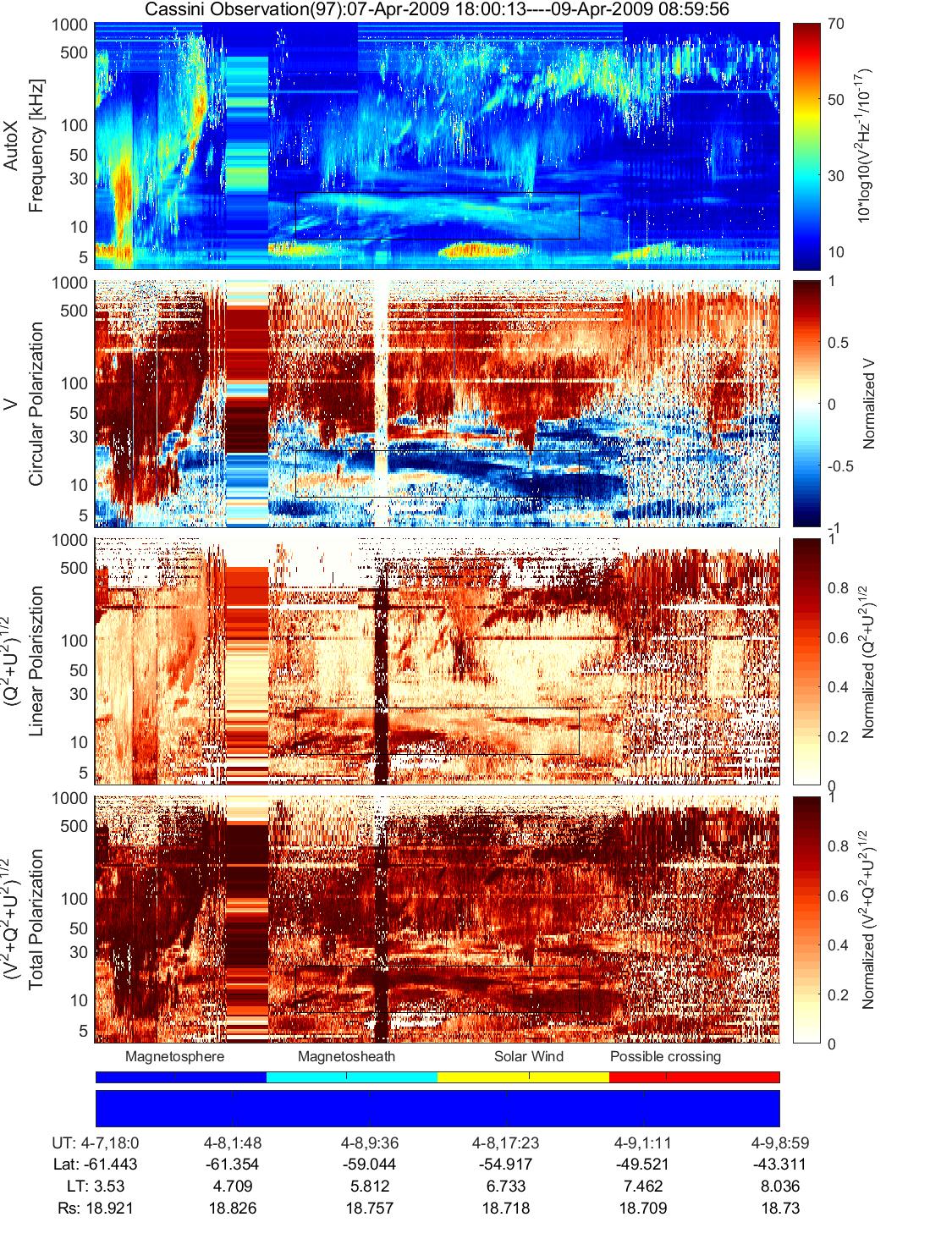
 Figure S71, Case 71 of the SAM emission in Table S1. Same format as Figure S1.
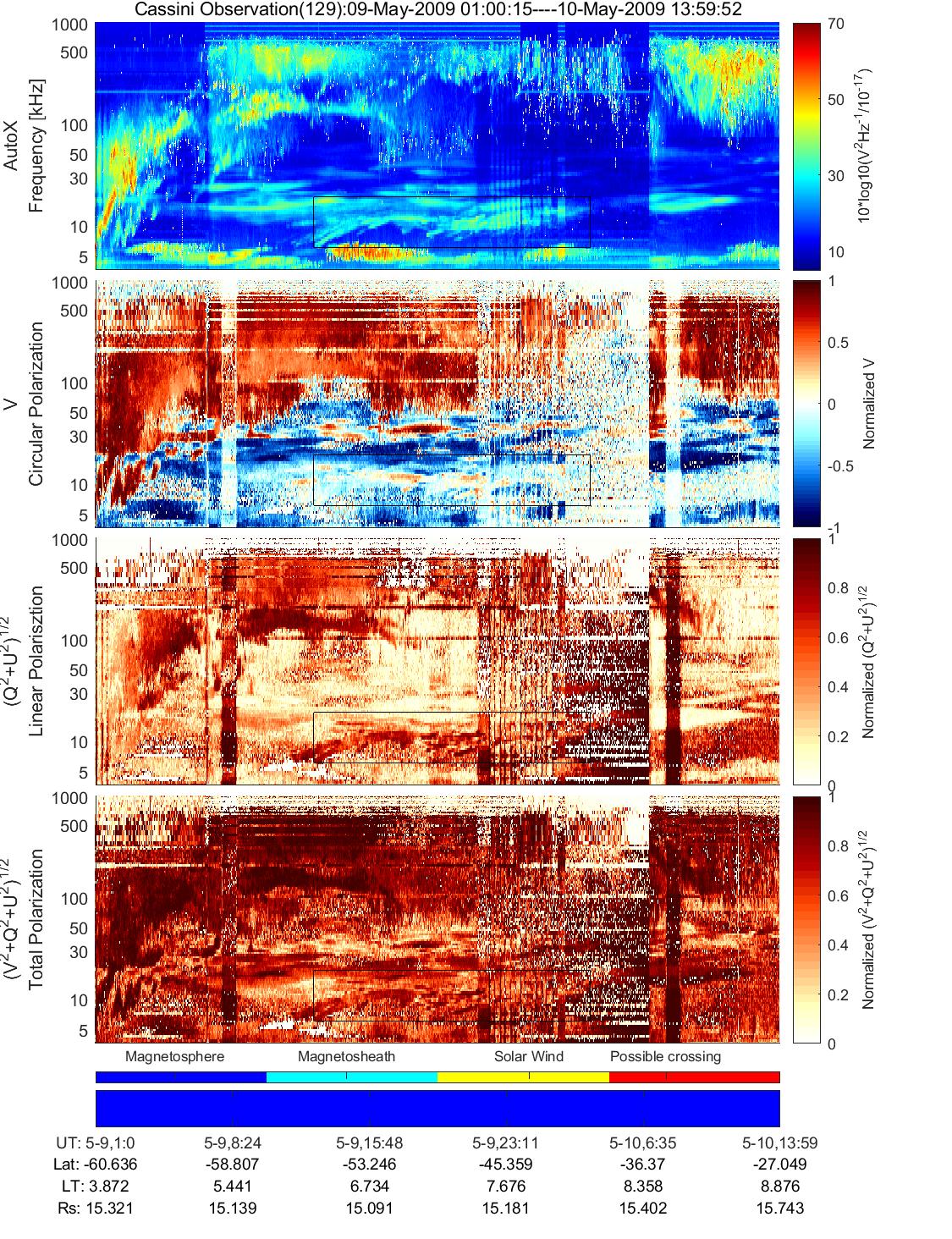
 Figure S72, Case 72 of the SAM emission in Table S1. Same format as Figure S1.


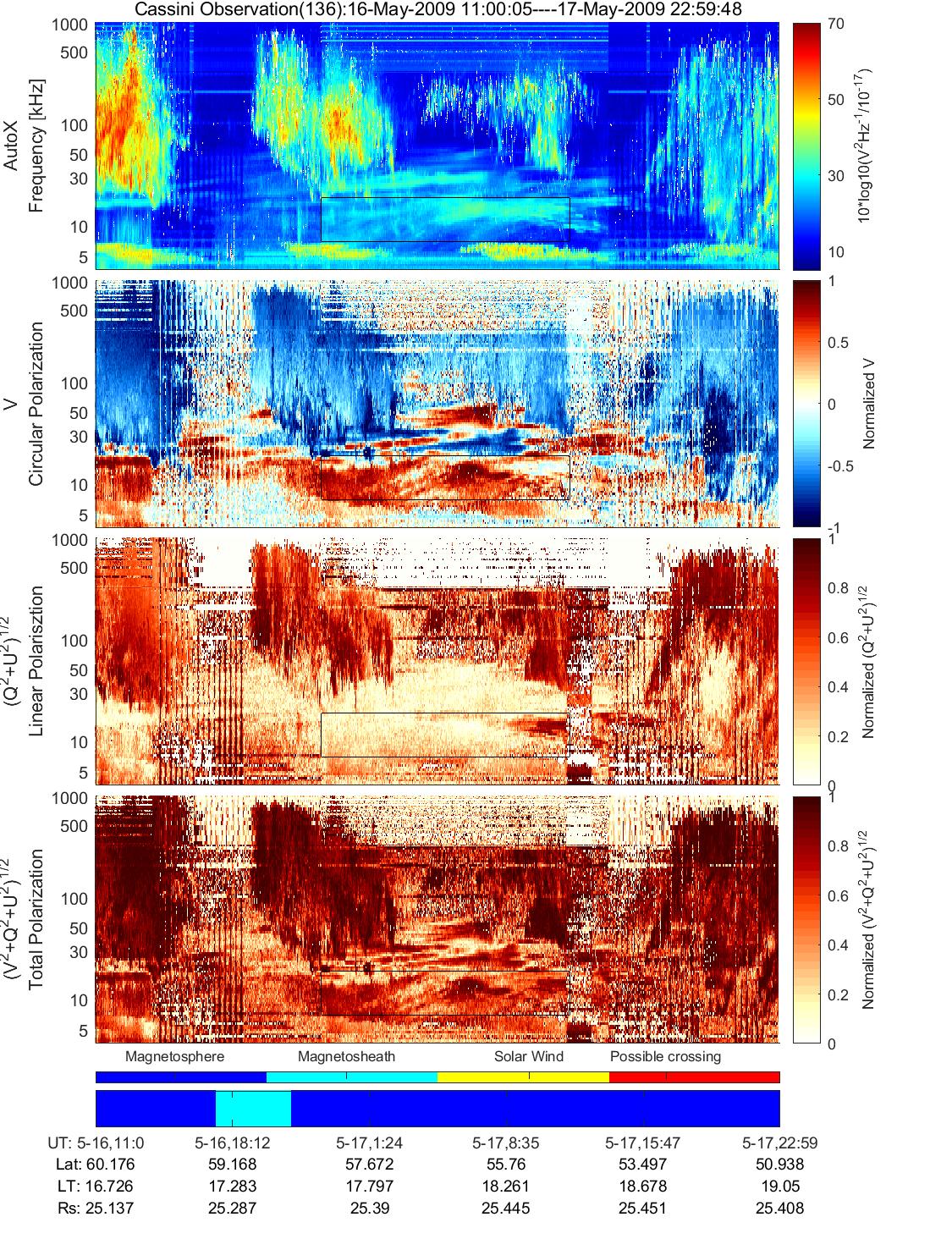
 Figure S73, Case 73 of the SAM emission in Table S1. Same format as Figure S1.


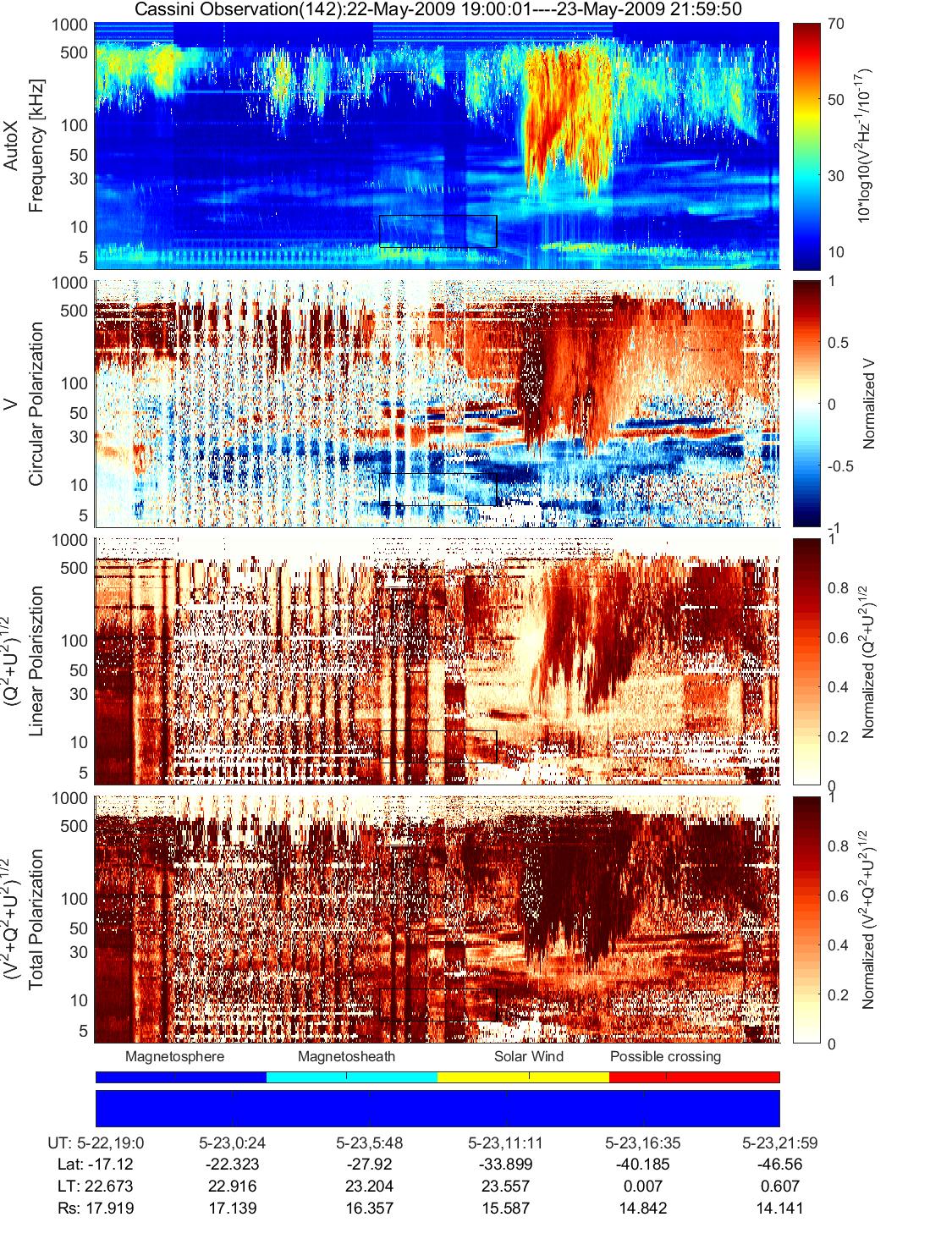
 Figure S74, Case 74 of the SAM emission in Table S1. Same format as Figure S1.


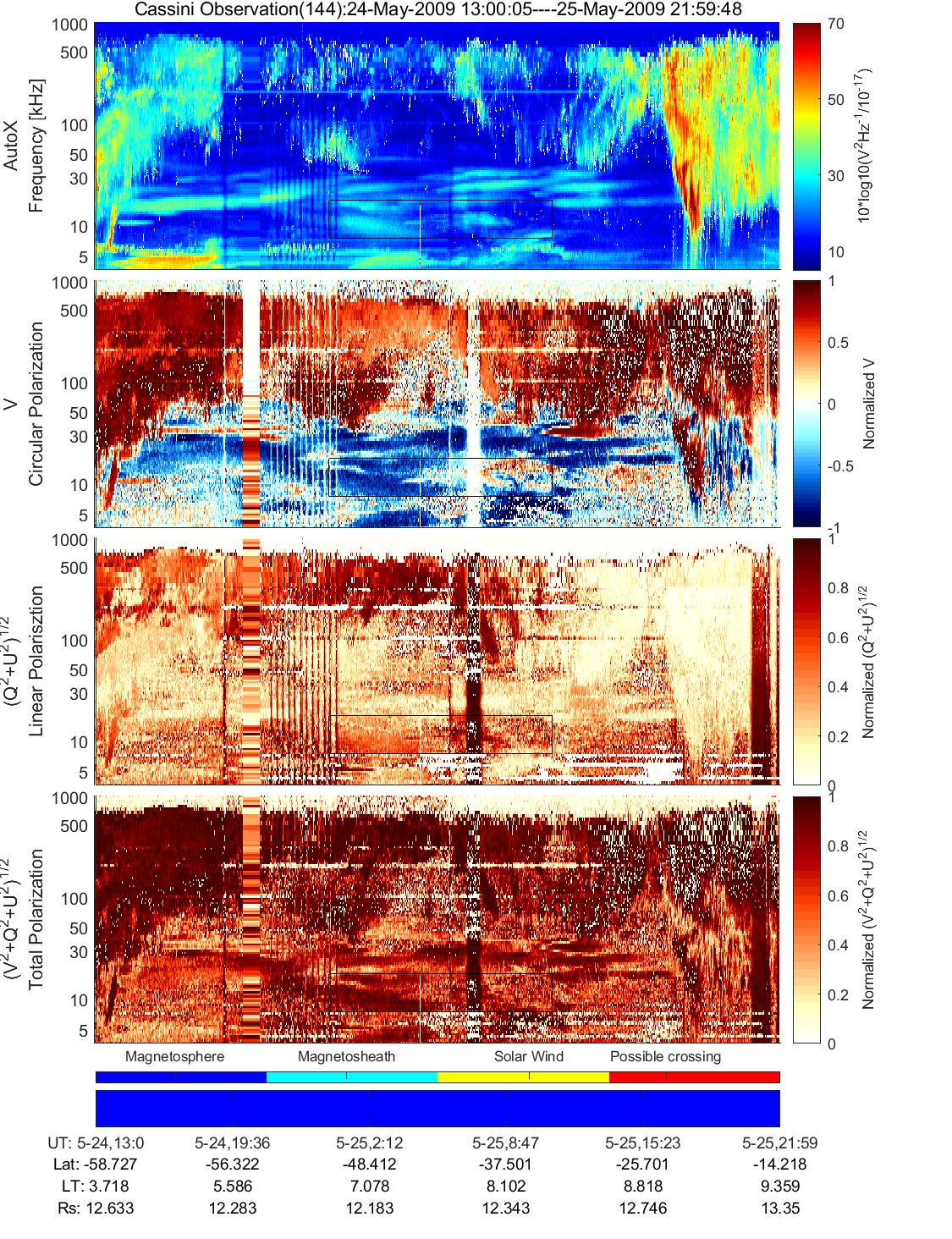
 Figure S75, Case 75 of the SAM emission in Table S1. Same format as Figure S1.


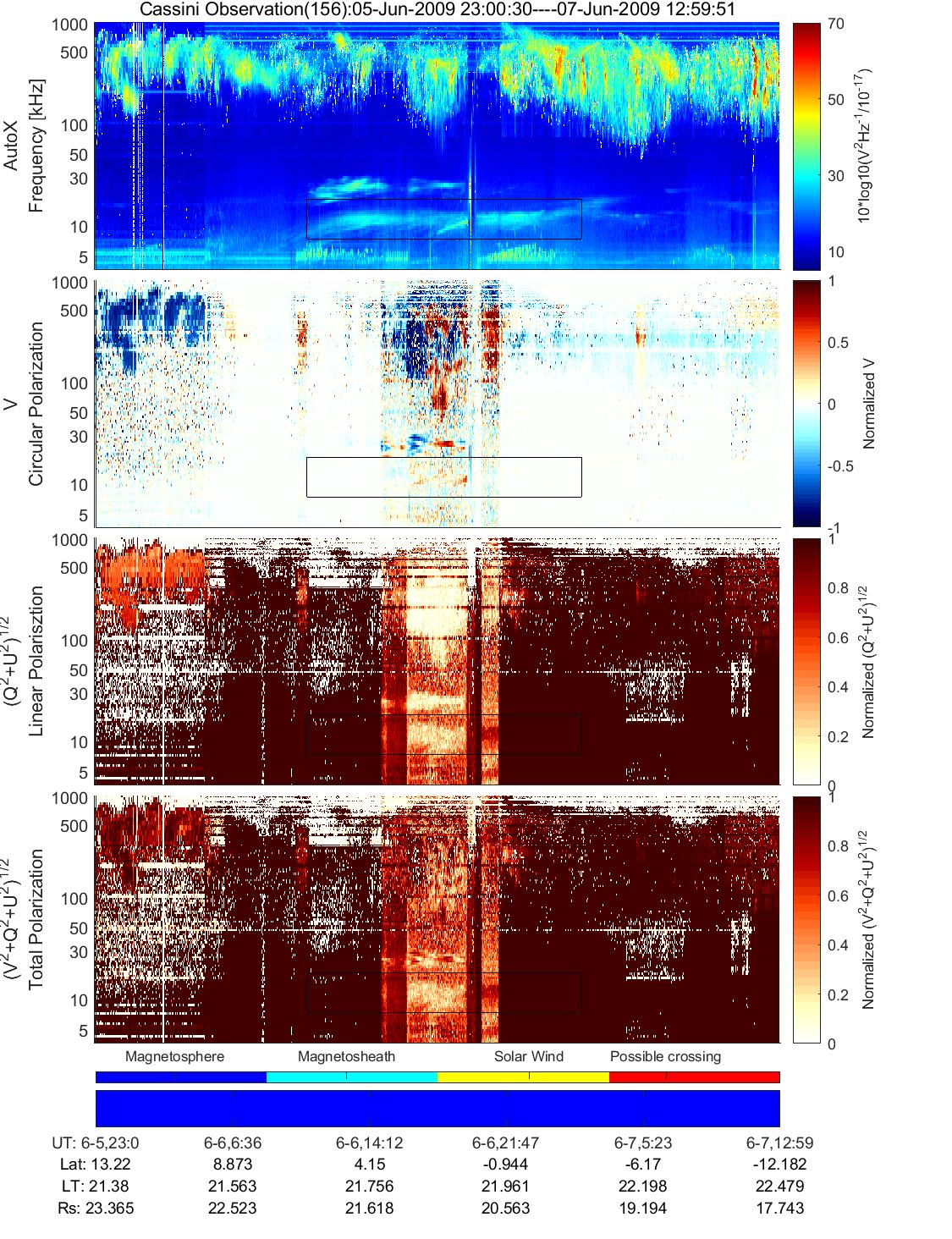
 Figure S76, Case 76 of the SAM emission in Table S1. Same format as Figure S1.


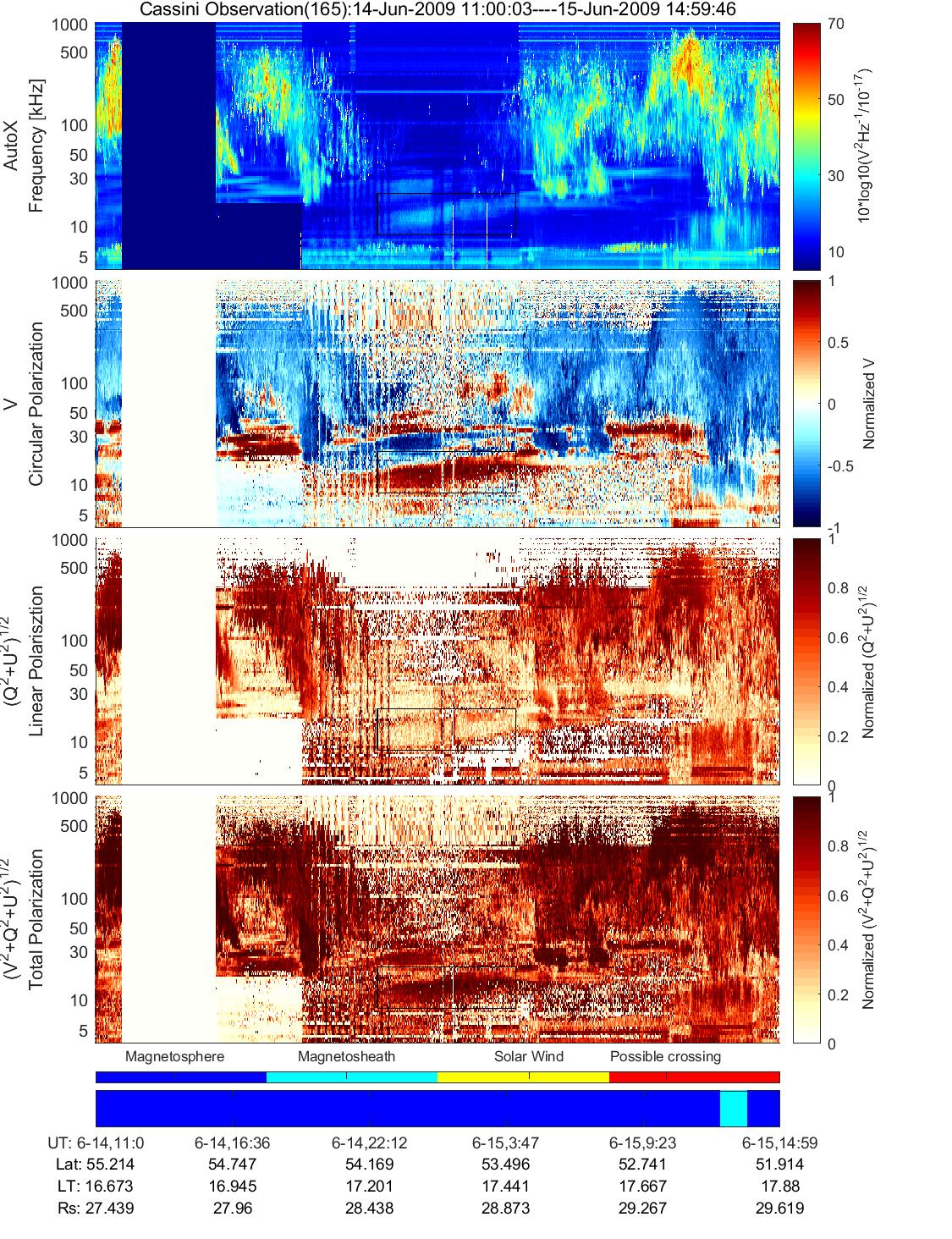
 Figure S77, Case 77 of the SAM emission in Table S1. Same format as Figure S1.


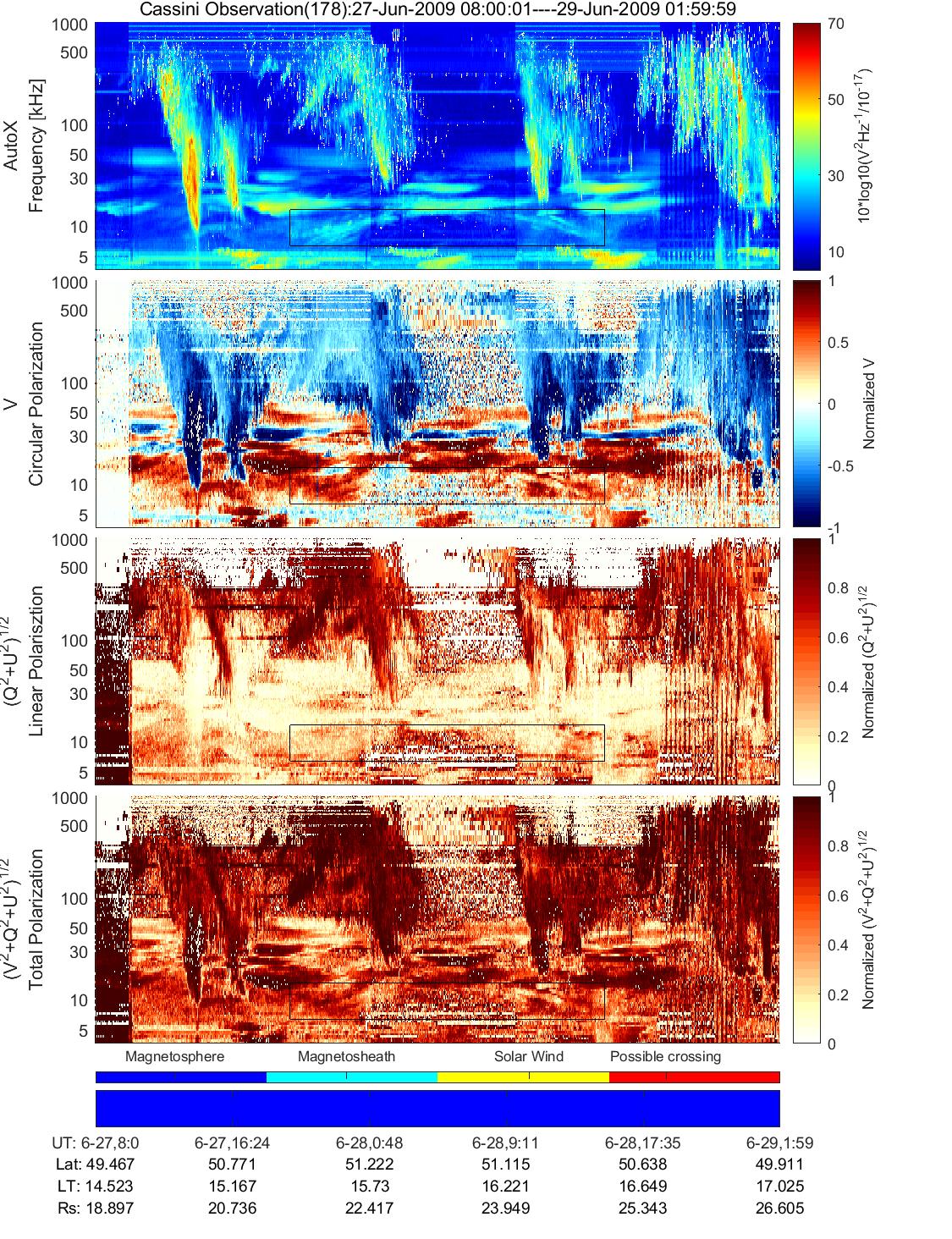
 Figure S78, Case 78 of the SAM emission in Table S1. Same format as Figure S1.


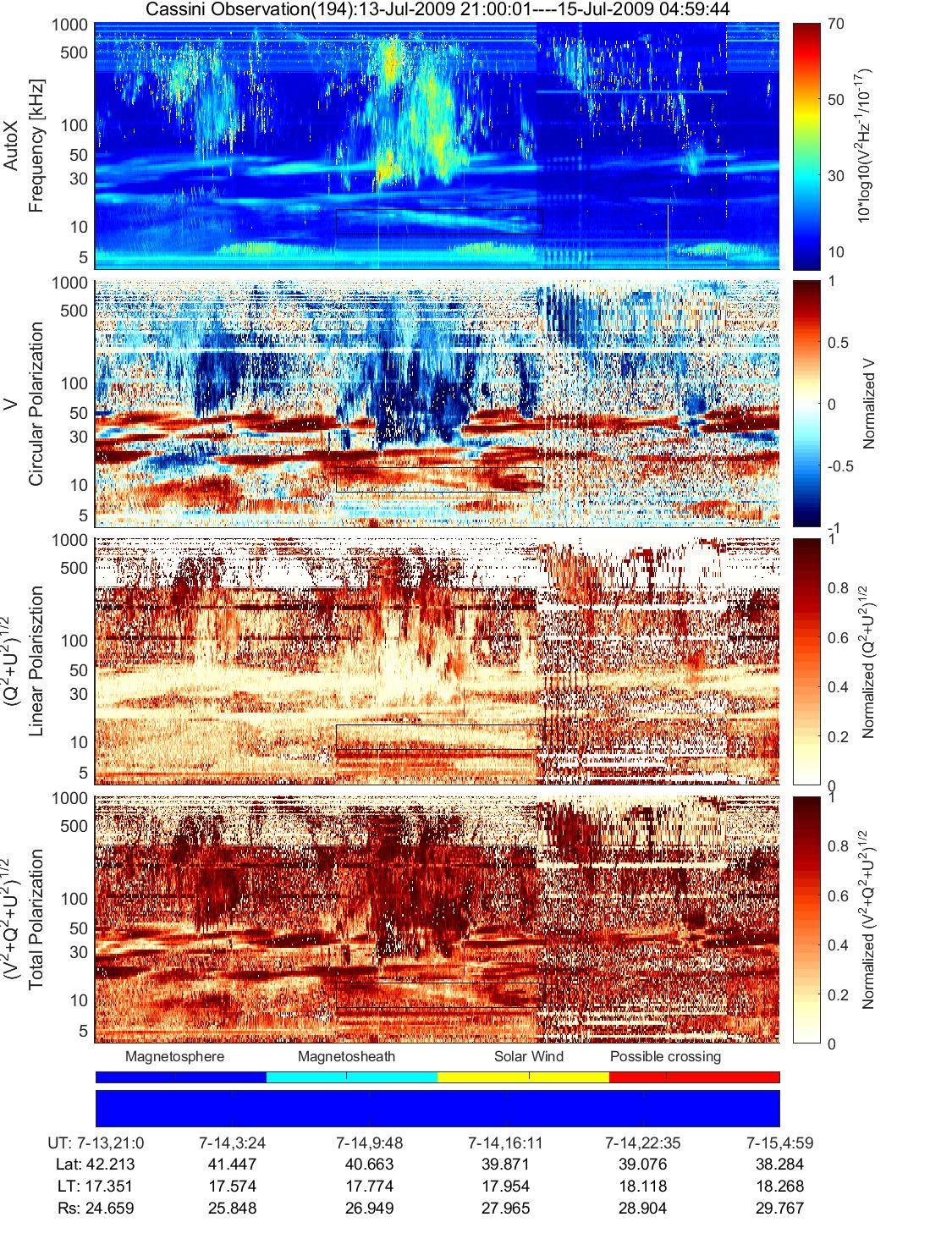
 Figure S79, Case 79 of the SAM emission in Table S1. Same format as Figure S1.


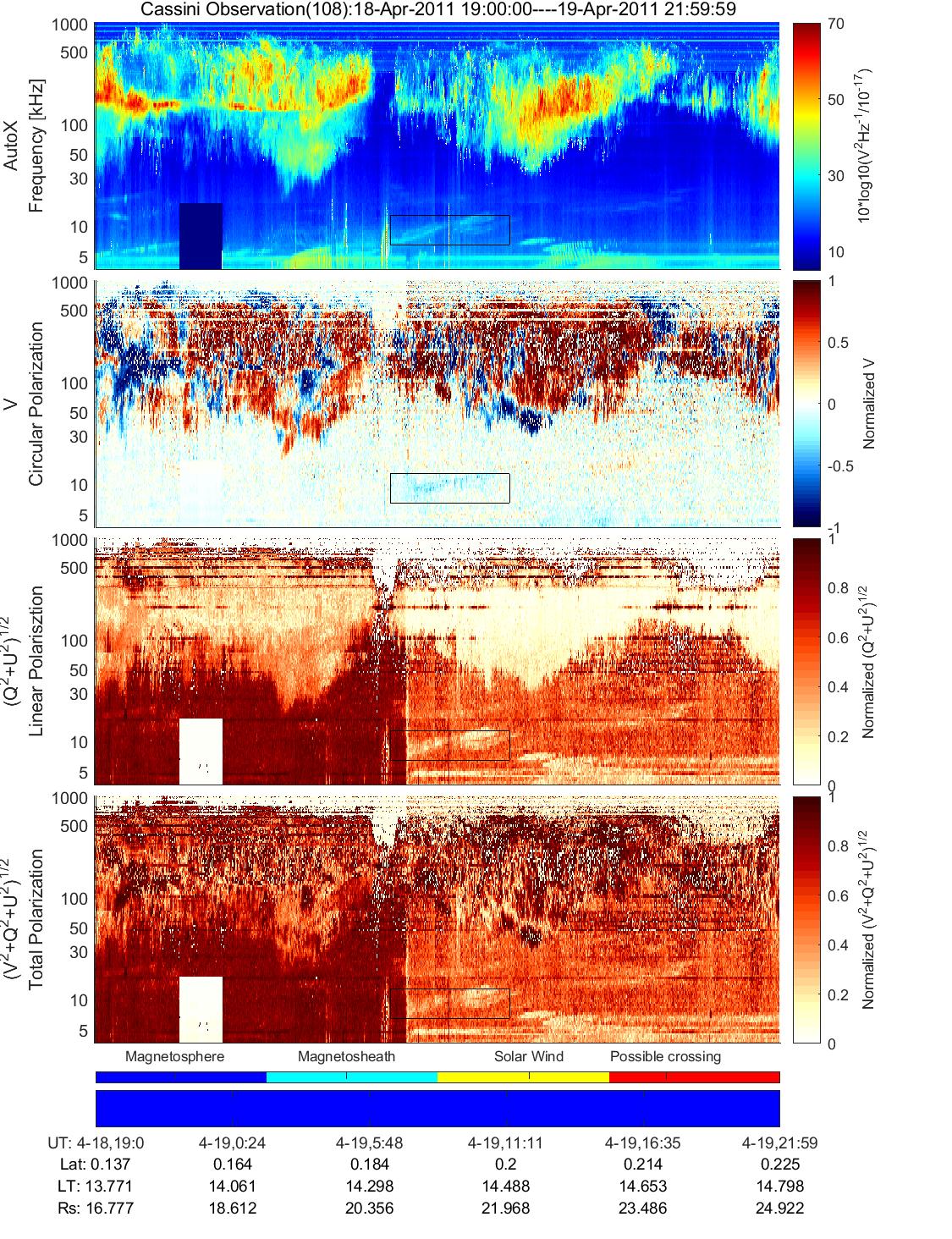
 Figure S80, Case 80 of the SAM emission in Table S1. Same format as Figure S1.


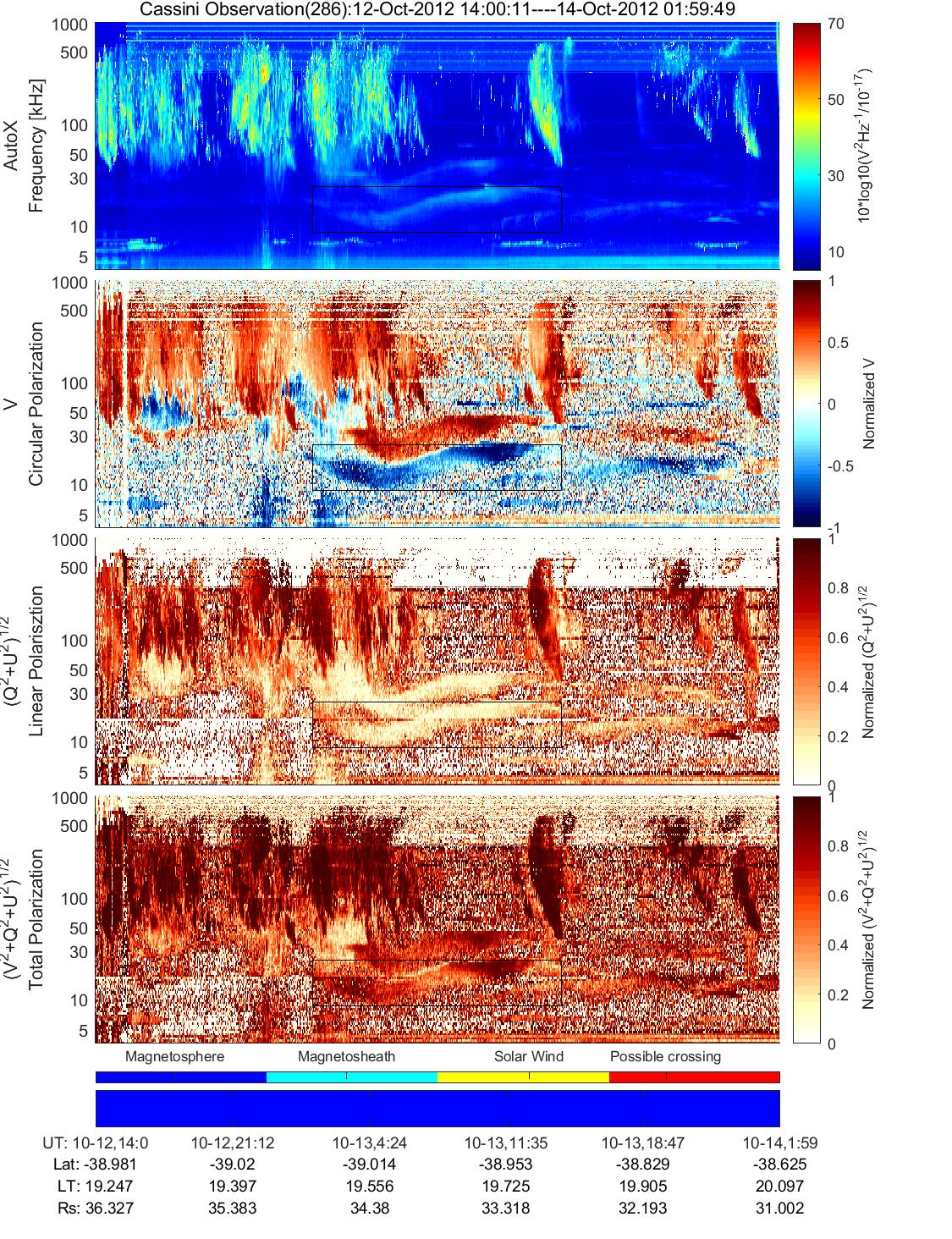
 Figure S81, Case 81 of the SAM emission in Table S1. Same format as Figure S1.


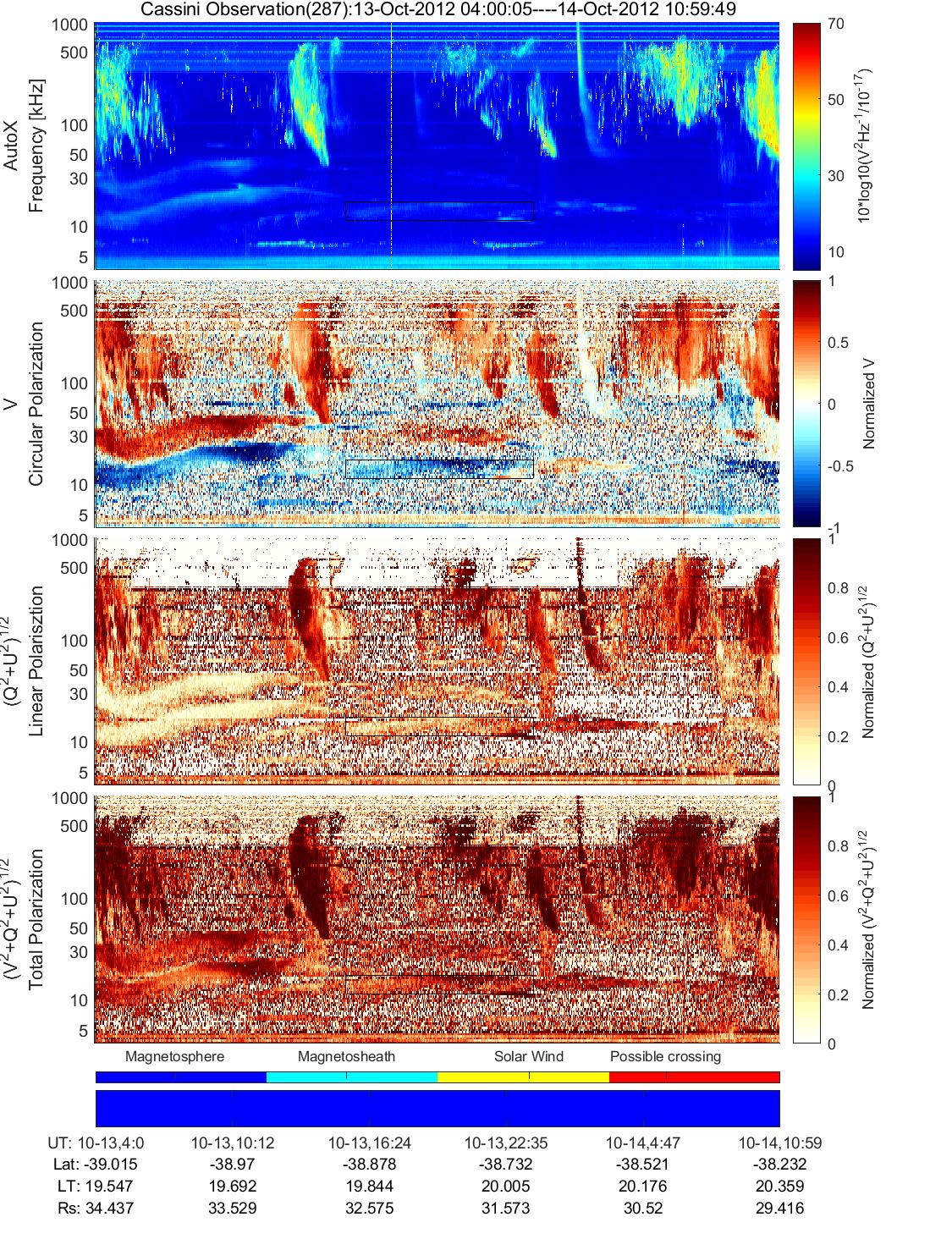
 Figure S82, Case 82 of the SAM emission in Table S1. Same format as Figure S1.


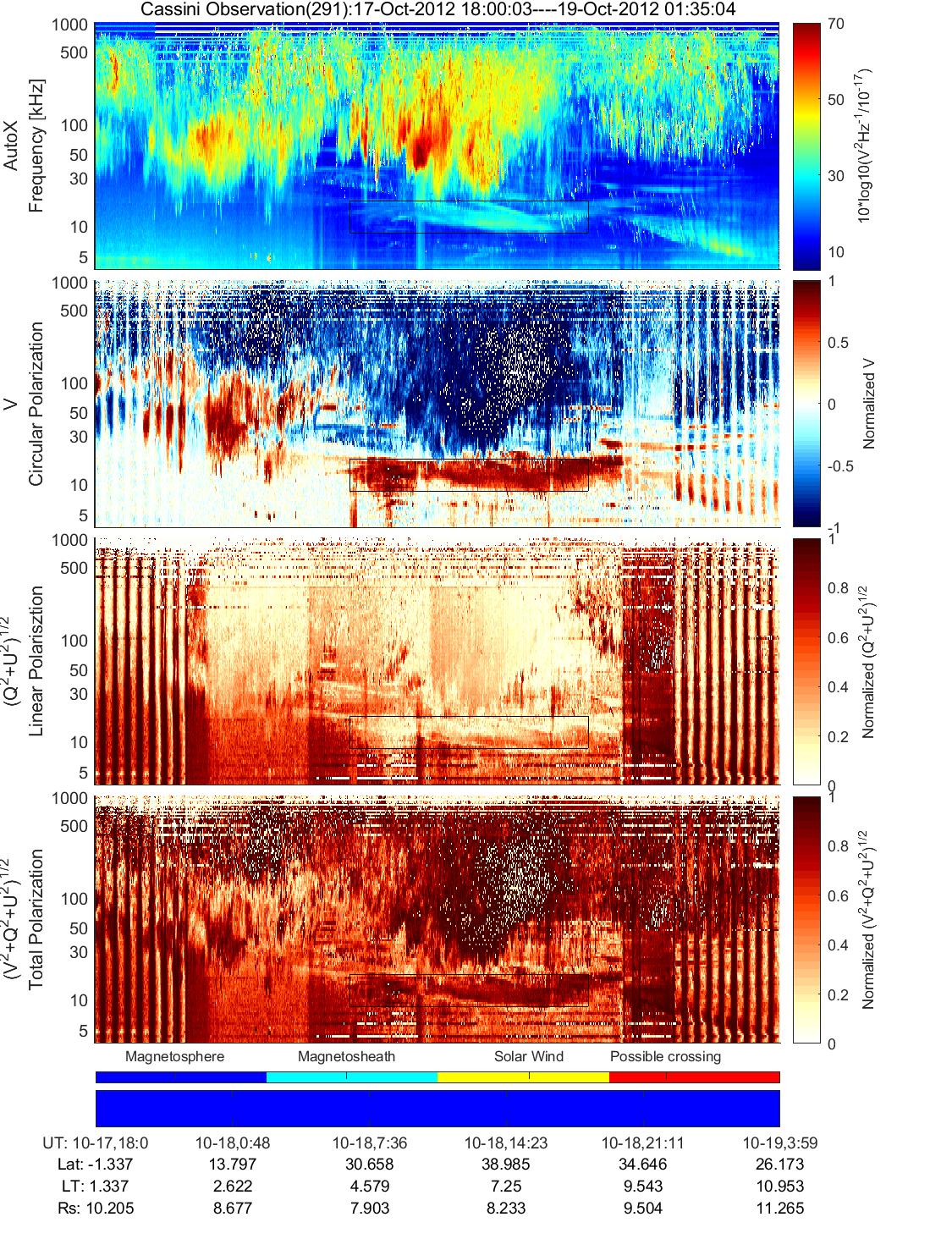
 Figure S83, Case 83 of the SAM emission in Table S1. Same format as Figure S1.


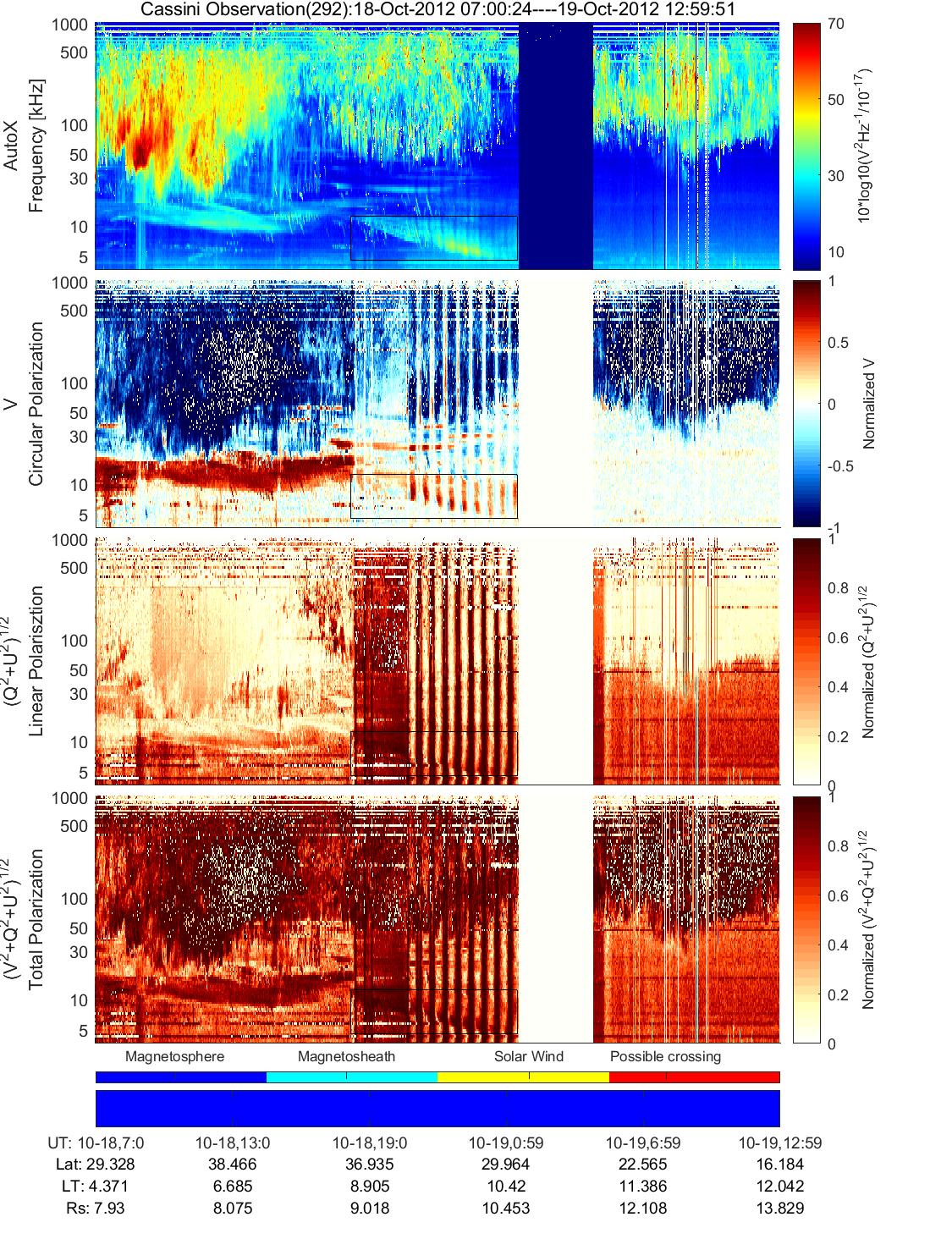
 Figure S84, Case 84 of the SAM emission in Table S1. Same format as Figure S1.


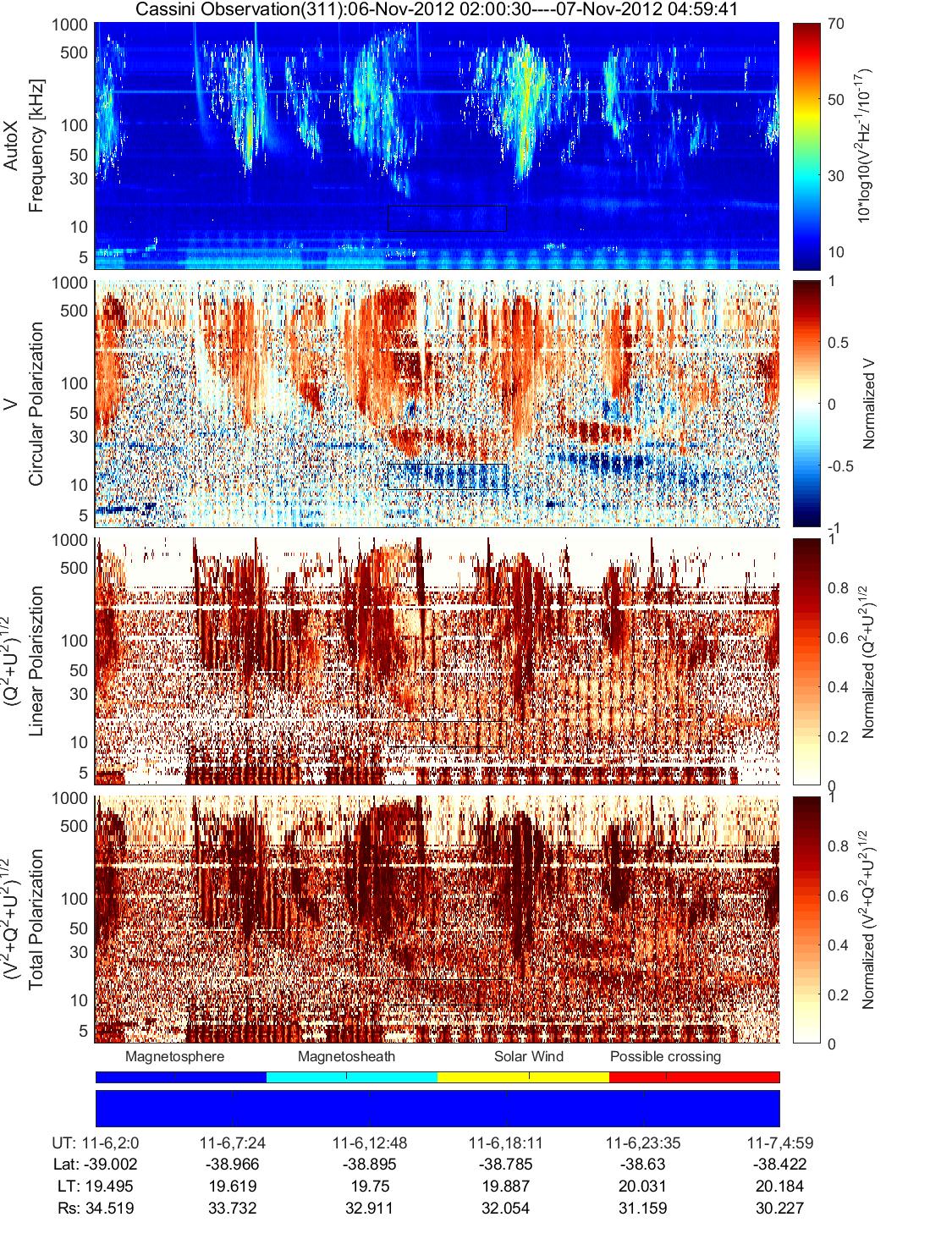
 Figure S85, Case 85 of the SAM emission in Table S1. Same format as Figure S1.


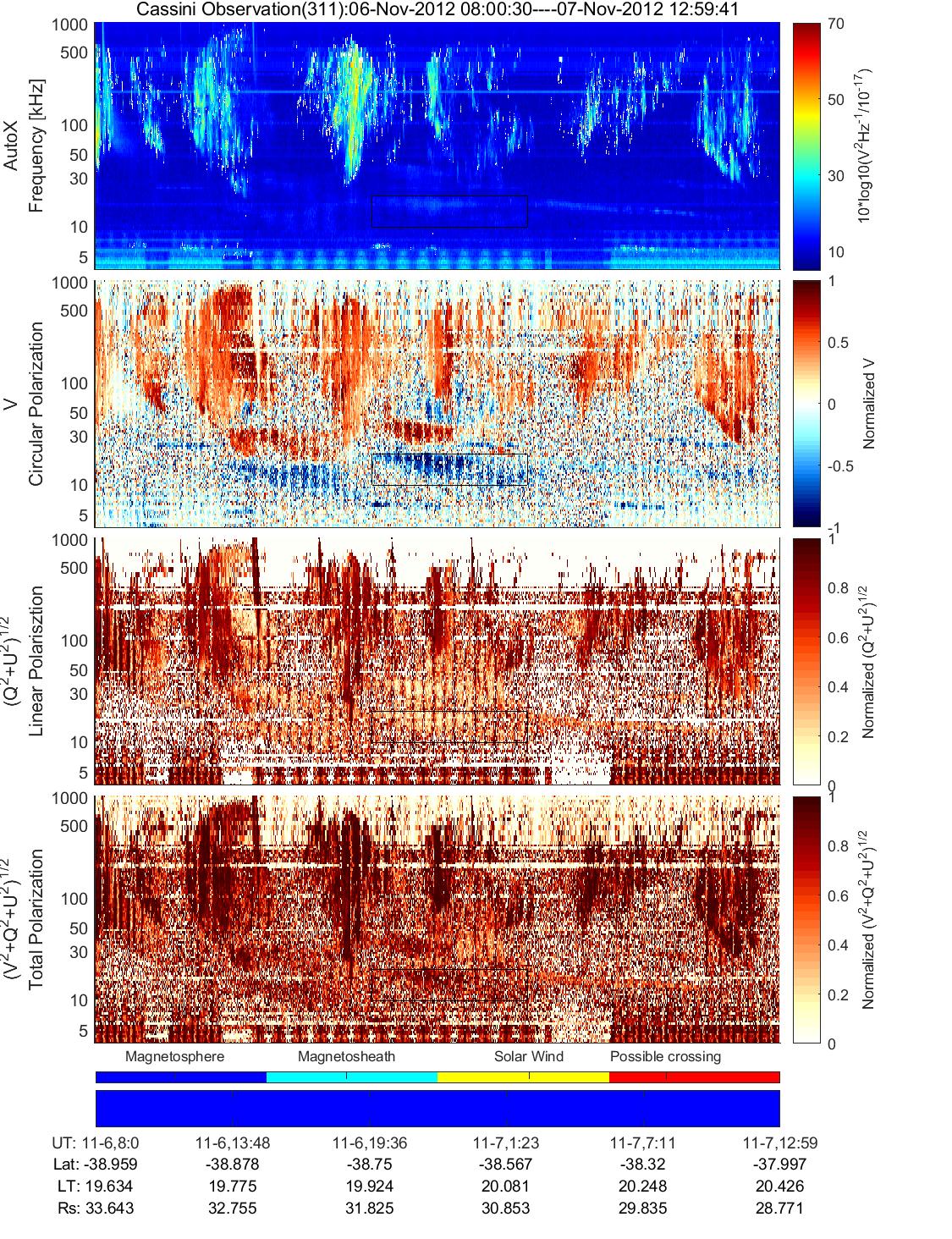
 Figure S86, Case 86 of the SAM emission in Table S1. Same format as Figure S1.


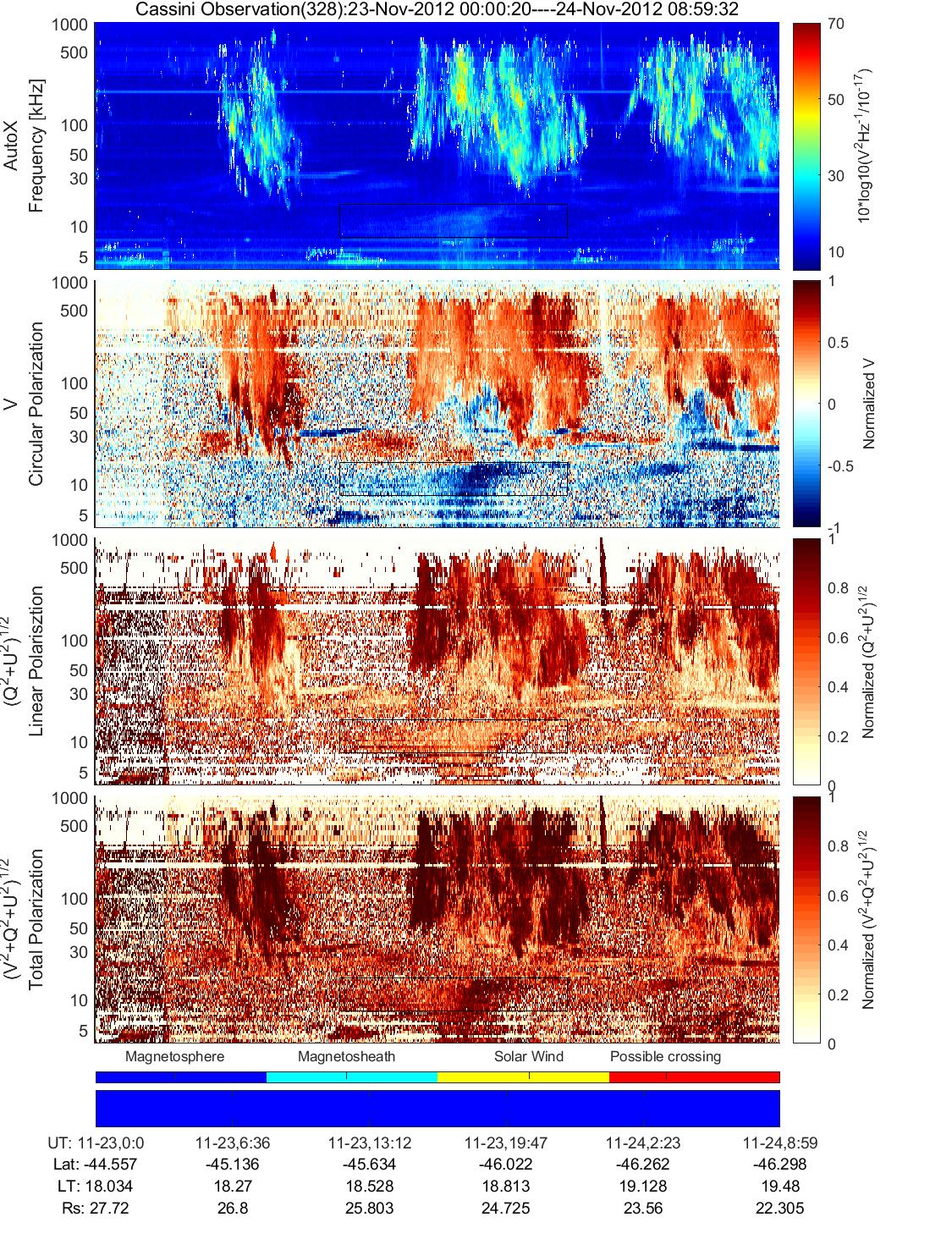
 Figure S87, Case 87 of the SAM emission in Table S1. Same format as Figure S1.


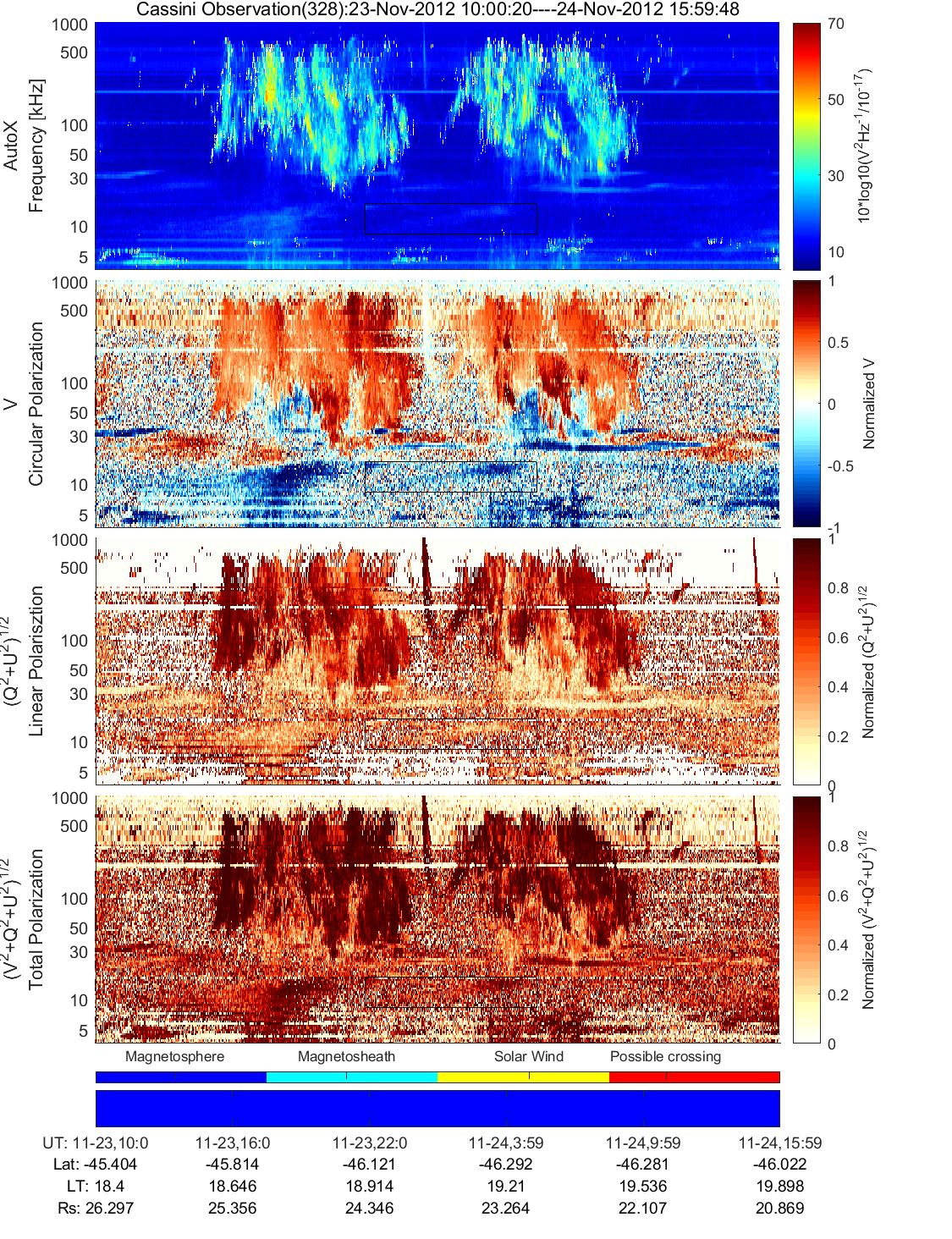
 Figure S88, Case 88 of the SAM emission in Table S1. Same format as Figure S1.


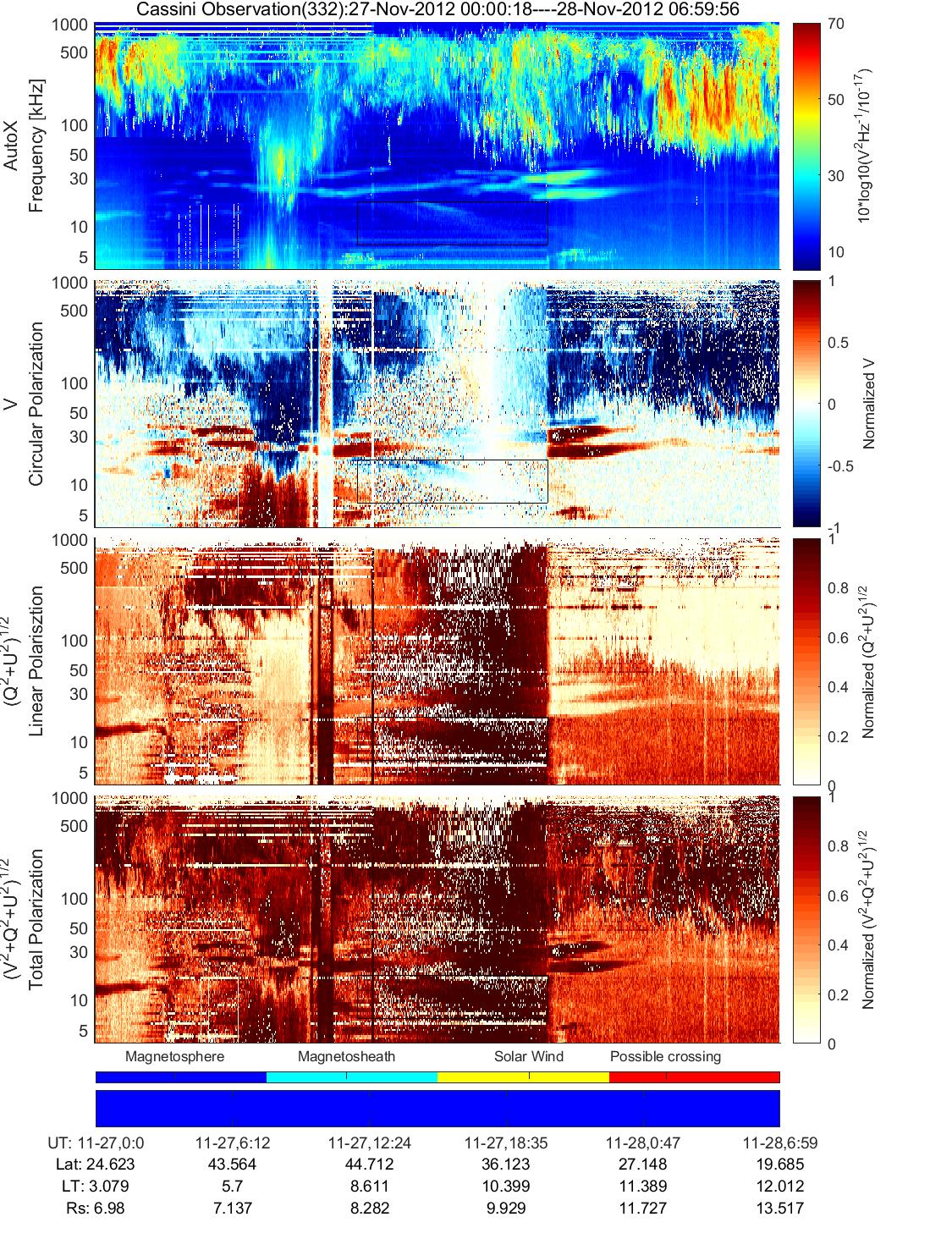
 Figure S89, Case 89 of the SAM emission in Table S1. Same format as Figure S1.


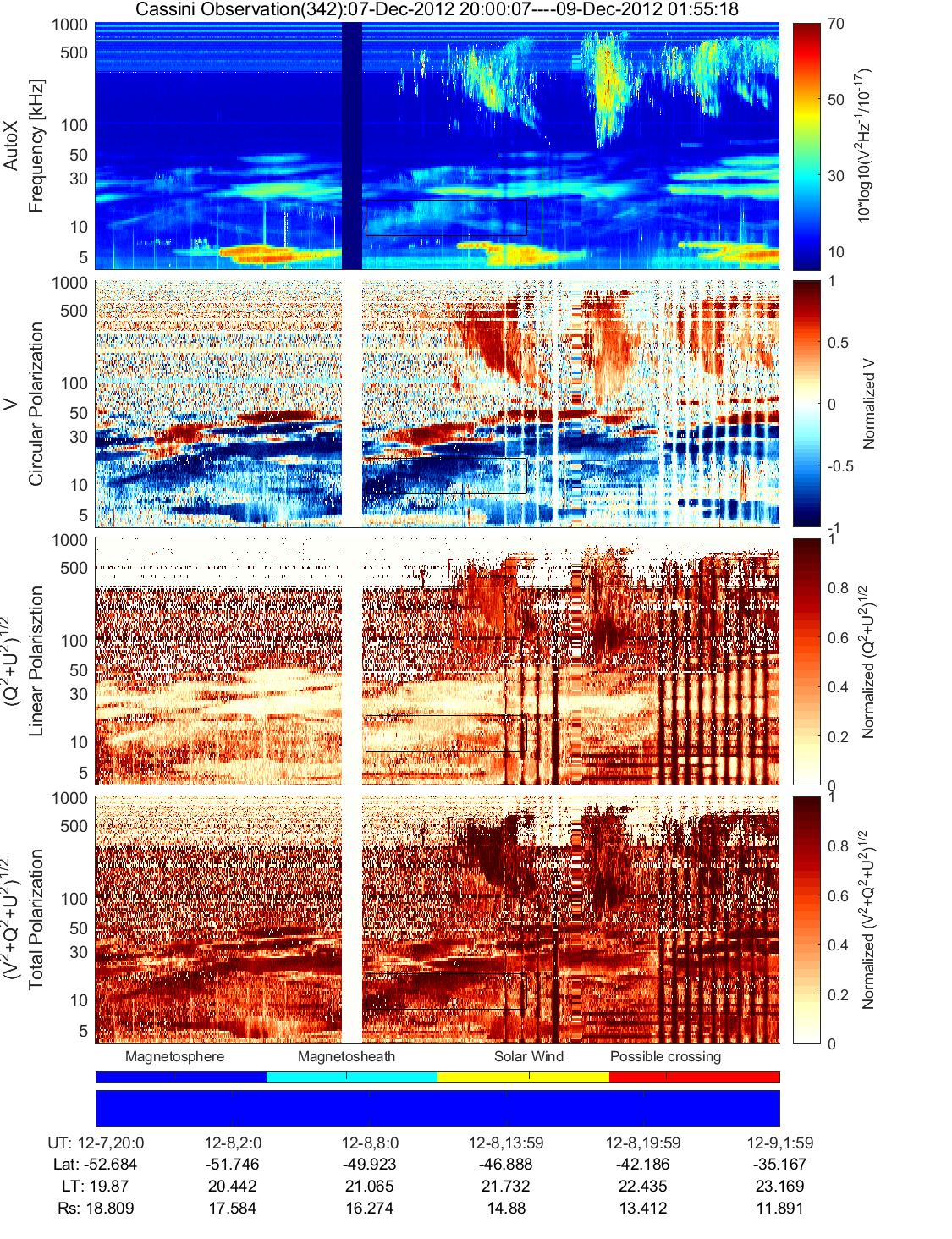
 Figure S90, Case 90 of the SAM emission in Table S1. Same format as Figure S1.


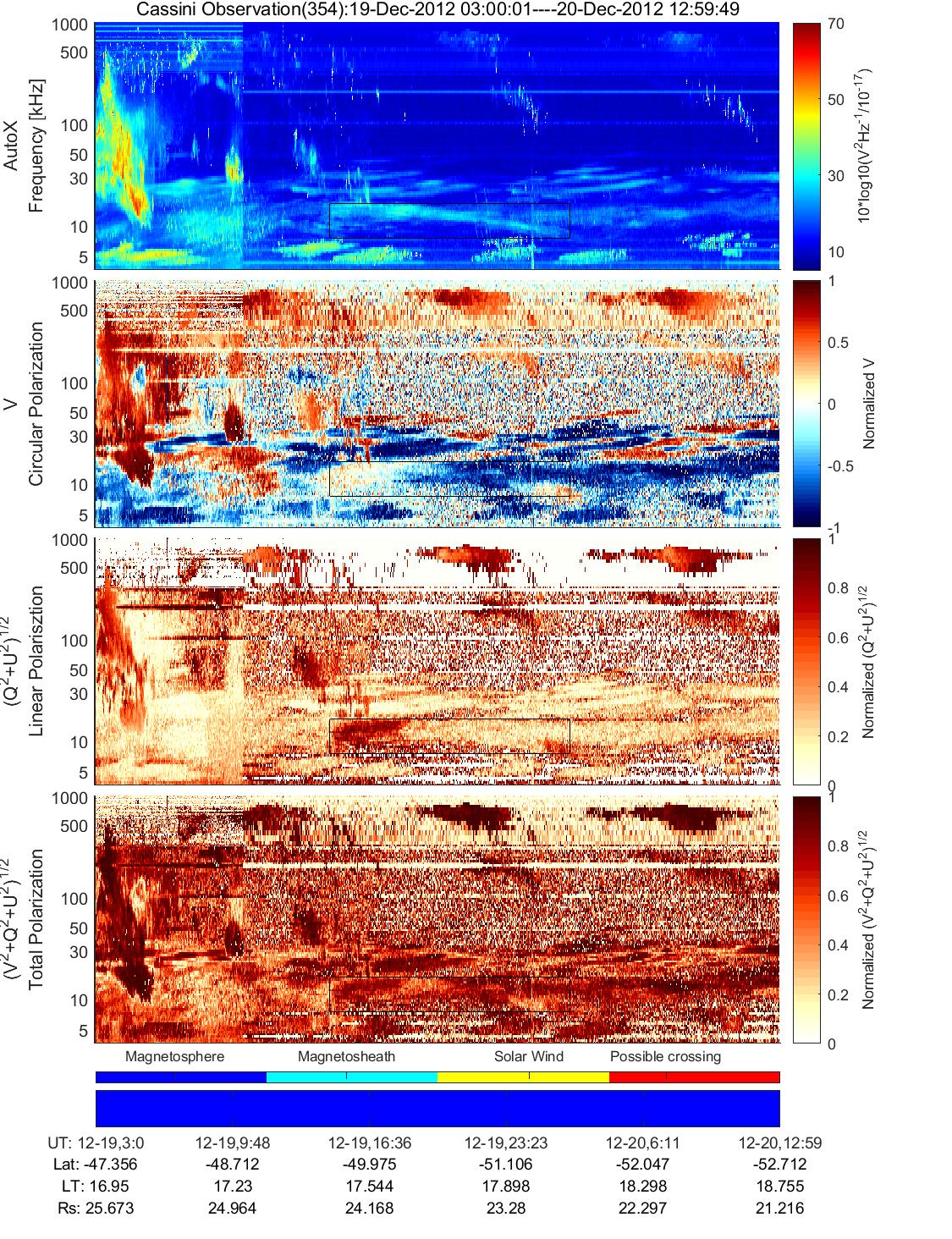
 Figure S91, Case 91 of the SAM emission in Table S1. Same format as Figure S1.


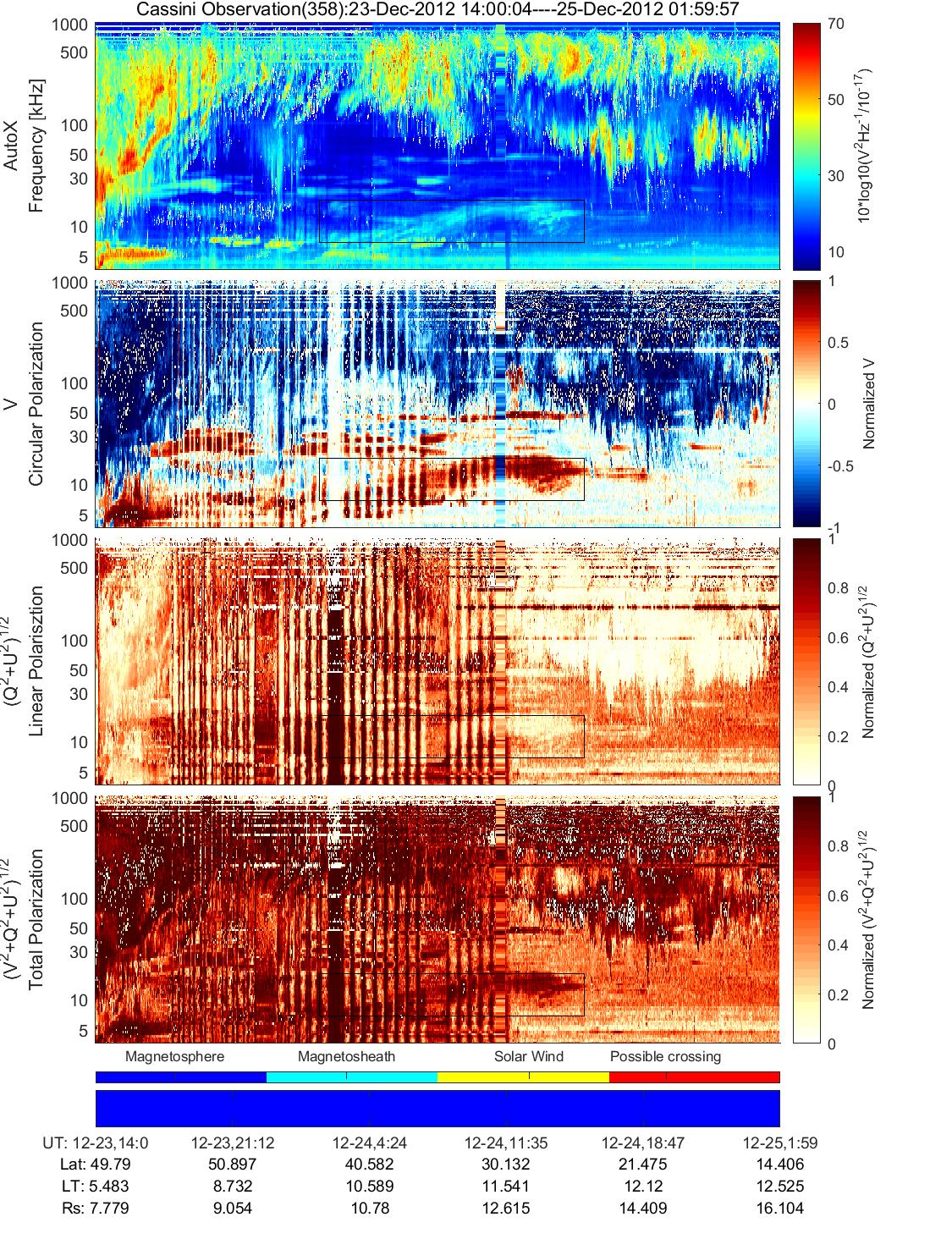
 Figure S92, Case 92 of the SAM emission in Table S1. Same format as Figure S1.


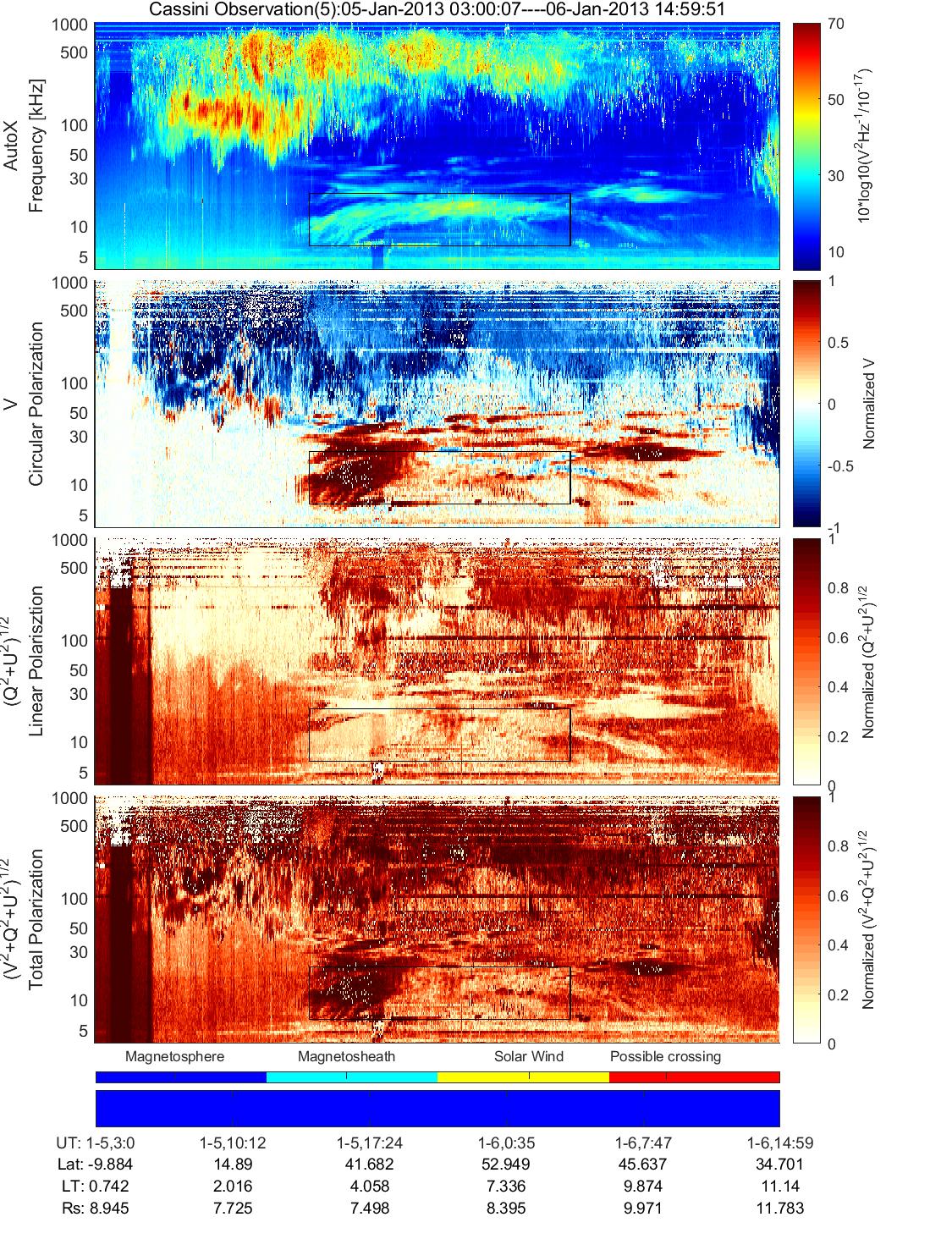
 Figure S93, Case 93 of the SAM emission in Table S1. Same format as Figure S1.


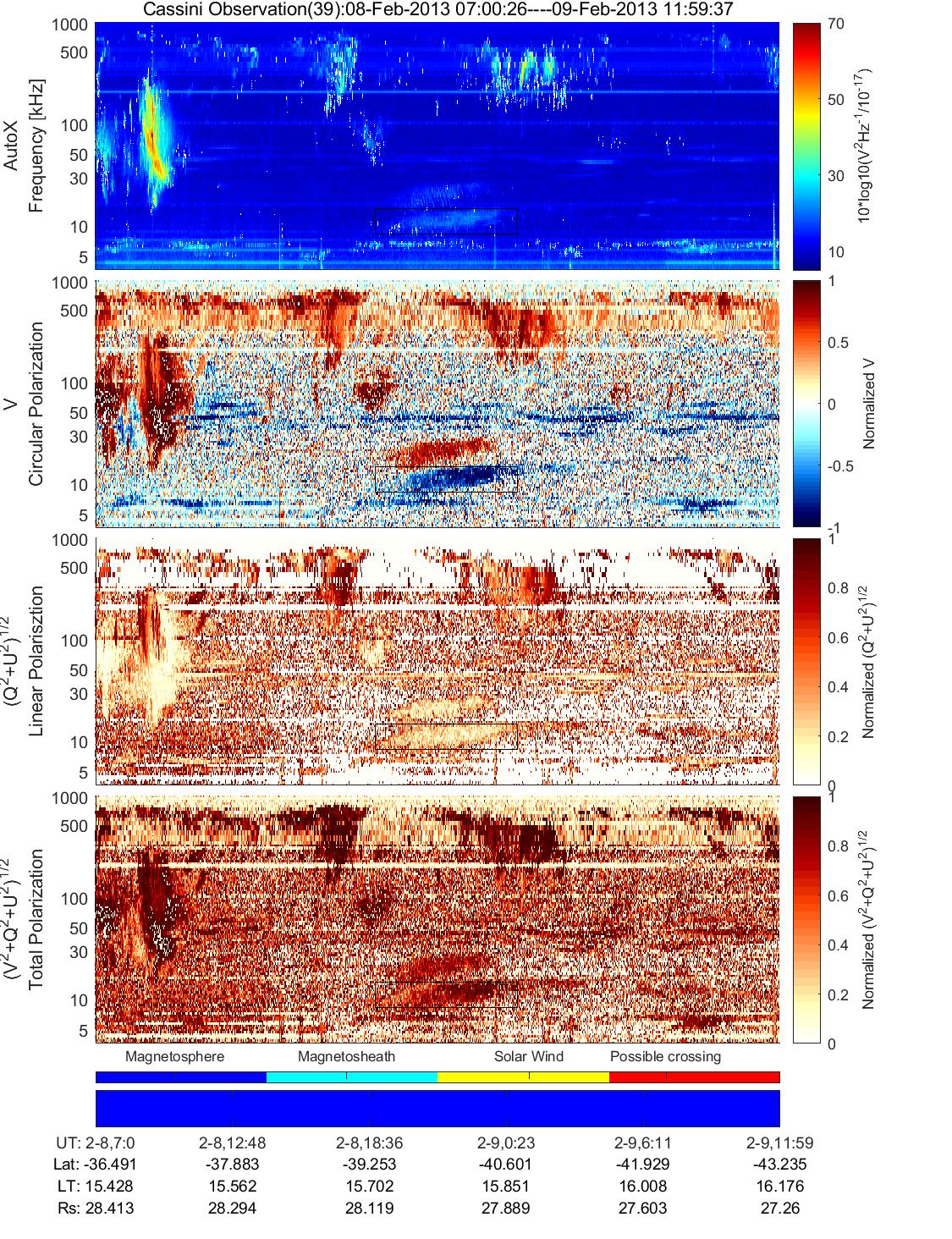
 Figure S94, Case 94 of the SAM emission in Table S1. Same format as Figure S1.


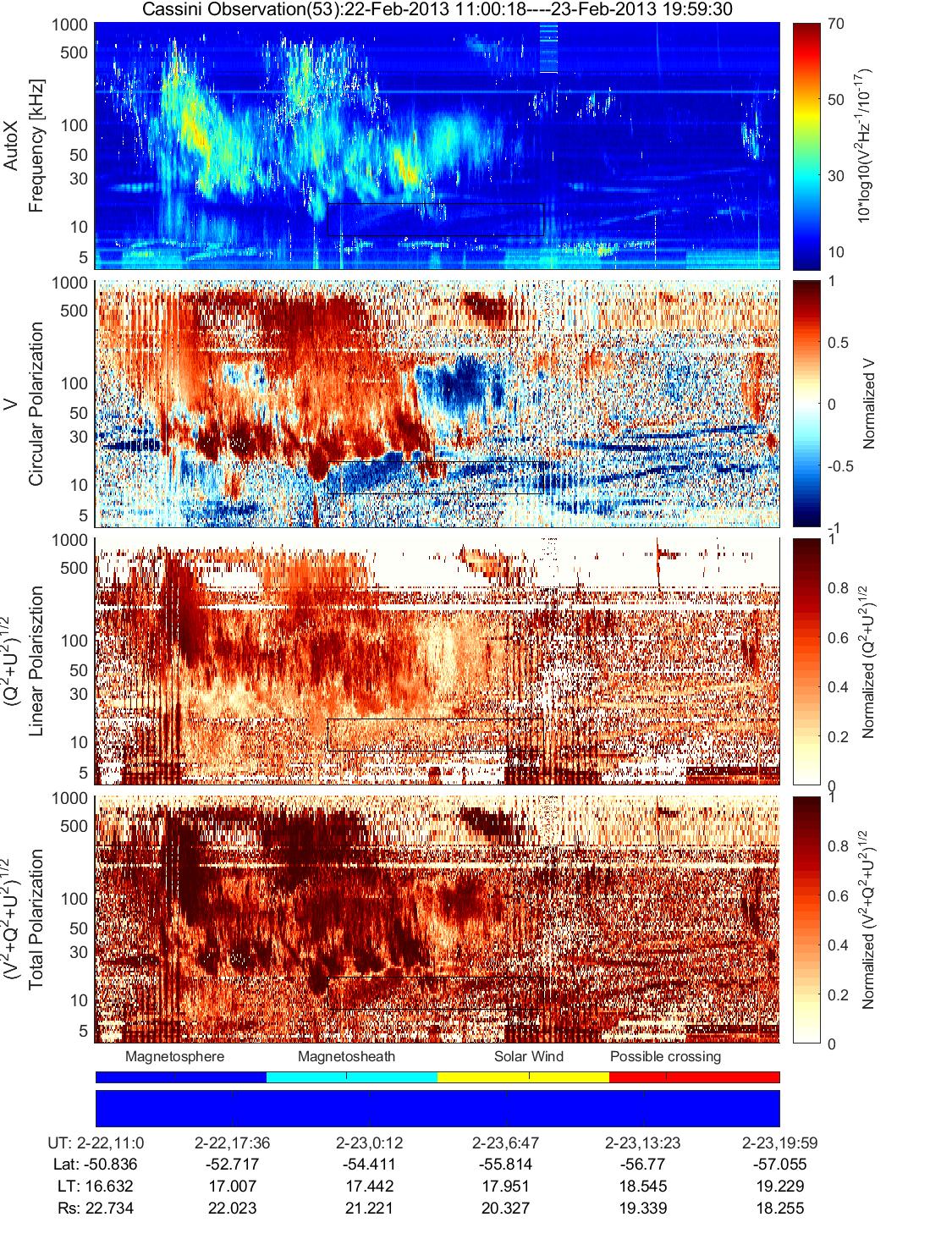
 Figure S95, Case 95 of the SAM emission in Table S1. Same format as Figure S1.


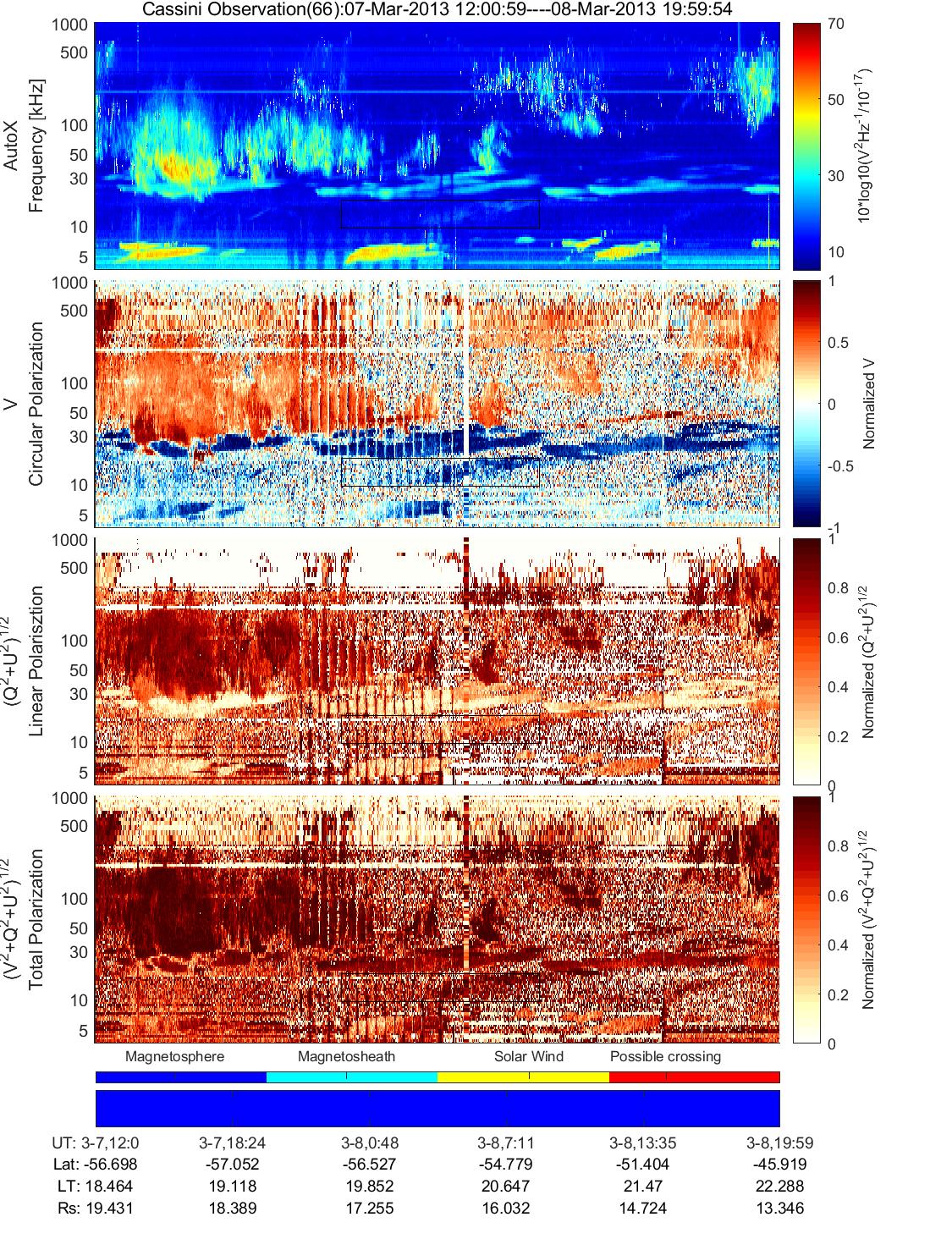
 Figure S96, Case 96 of the SAM emission in Table S1. Same format as Figure S1.


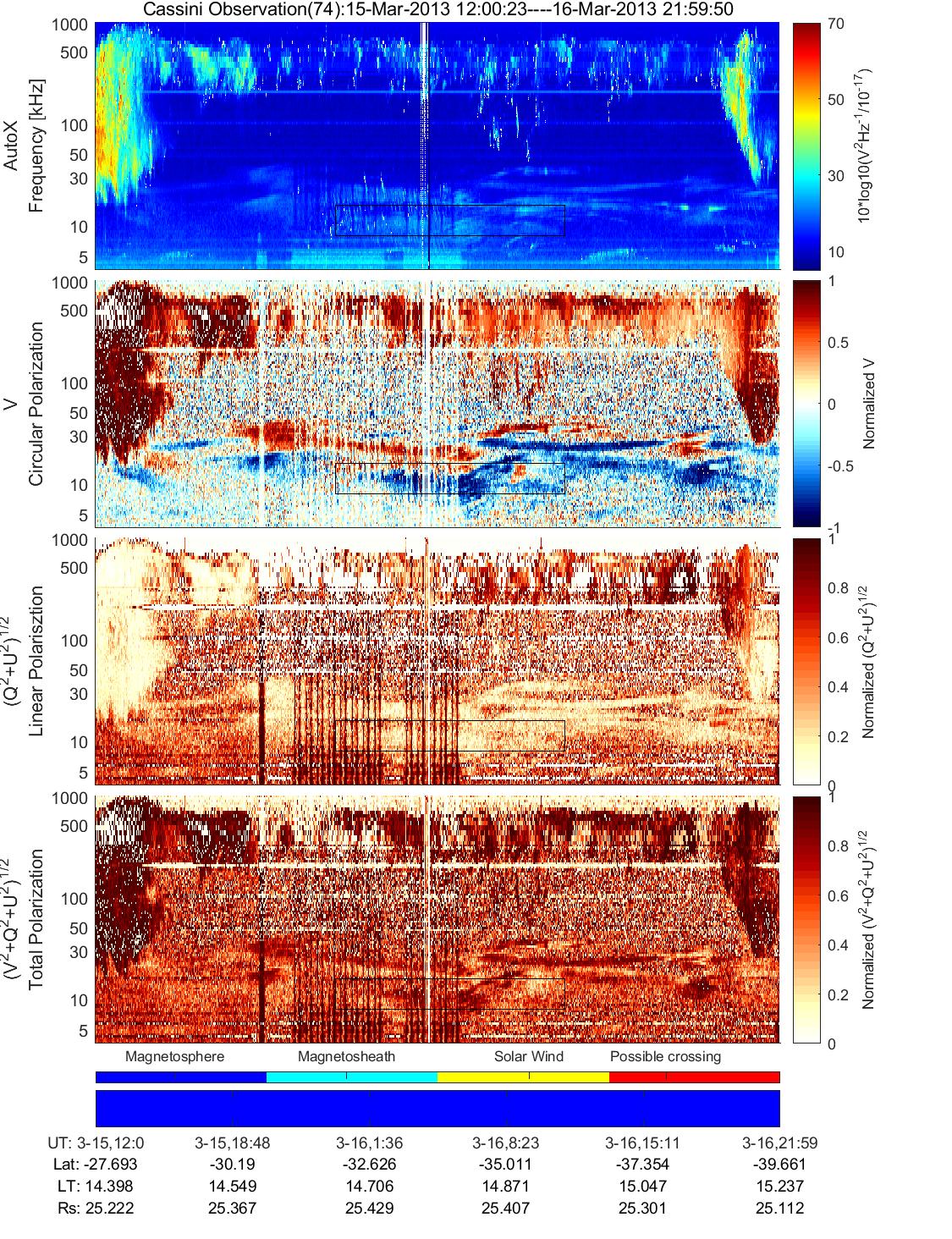
 Figure S97, Case 97 of the SAM emission in Table S1. Same format as Figure S1.


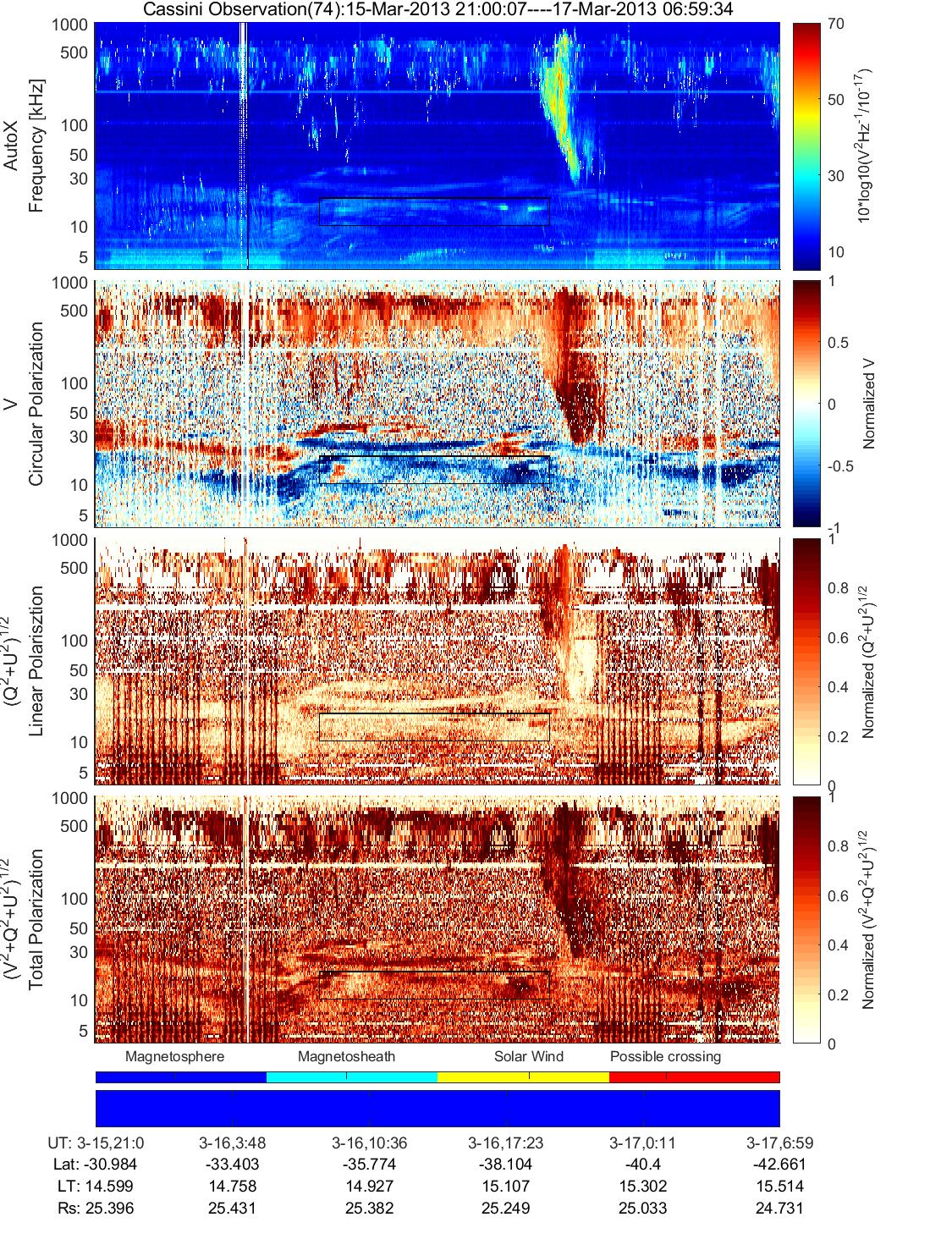
 Figure S98, Case 98 of the SAM emission in Table S1. Same format as Figure S1.


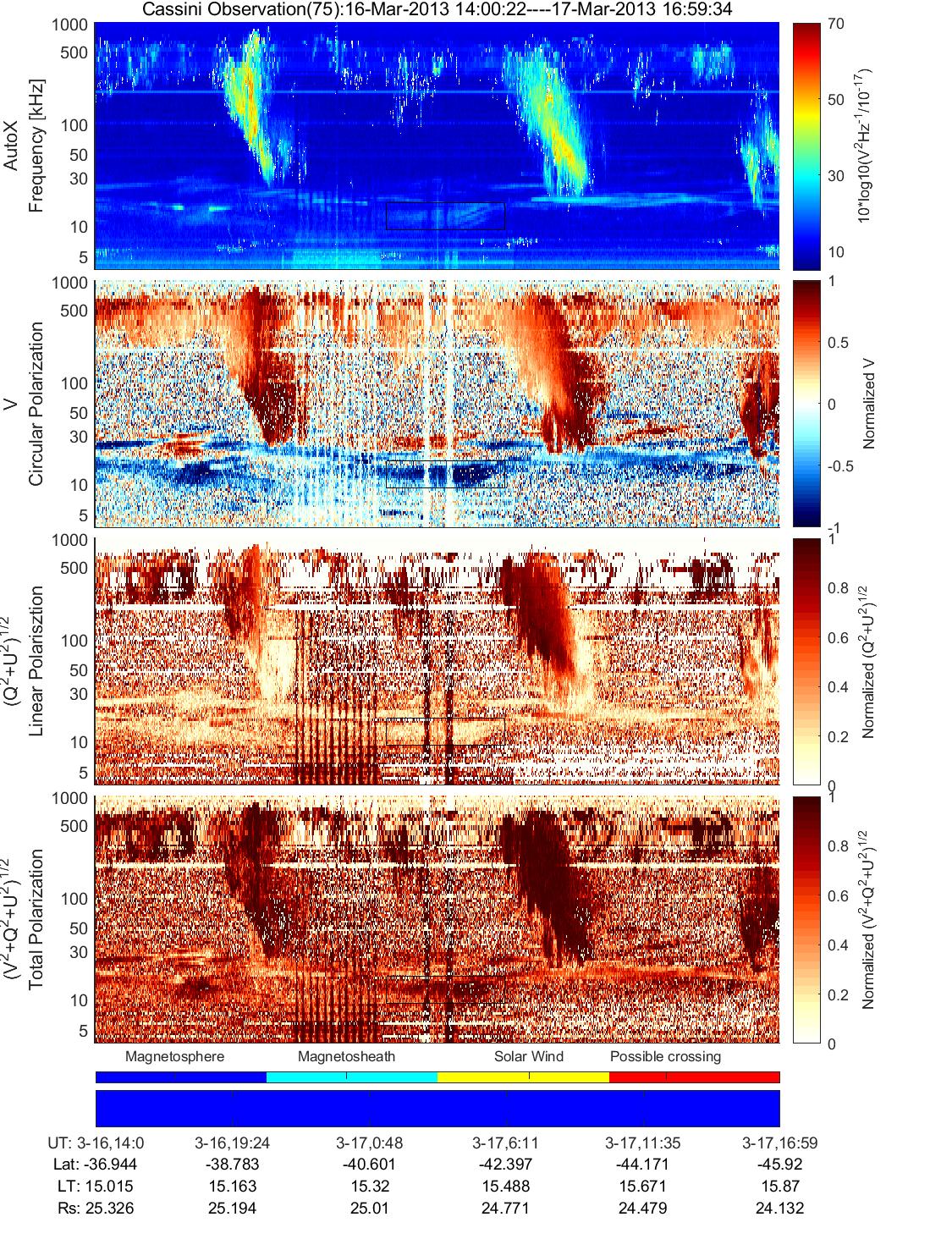
 Figure S99, Case 99 of the SAM emission in Table S1. Same format as Figure S1.

Figure S100, Case 100 of the SAM emission in Table S1. Same format as Figure S1.

Figure S101, Case 101 of the SAM emission in Table S1. Same format as Figure S1.

Figure S102, Case 102 of the SAM emission in Table S1. Same format as Figure S1. Figure S103, Case 103 of the SAM emission in Table S1. Same format as Figure S1. Figure S104, Case 104 of the SAM emission in Table S1. Same format as Figure S1. Figure S105, Case 105 of the SAM emission in Table S1. Same format as Figure S1. Figure S106, Case 106 of the SAM emission in Table S1. Same format as Figure S1. Figure S107, Case 107 of the SAM emission in Table S1. Same format as Figure S1. Figure S108, Case 108 of the SAM emission in Table S1. Same format as Figure S1. Figure S109, Case 109 of the SAM emission in Table S1. Same format as Figure S1. Figure S110, Case 110 of the SAM emission in Table S1. Same format as Figure S1. Figure S111, Case 111 of the SAM emission in Table S1. Same format as Figure S1. Figure S112, Case 112 of the SAM emission in Table S1. Same format as Figure S1. Figure S113, Case 113 of the SAM emission in Table S1. Same format as Figure S1. Figure S114, Case 114 of the SAM emission in Table S1. Same format as Figure S1. Figure S115, Case 115 of the SAM emission in Table S1. Same format as Figure S1. Figure S116, Case 116 of the SAM emission in Table S1. Same format as Figure S1. Figure S117, Case 117 of the SAM emission in Table S1. Same format as Figure S1. Figure S118, Case 118 of the SAM emission in Table S1. Same format as Figure S1. Figure S119, Case 119 of the SAM emission in Table S1. Same format as Figure S1. Figure S120, Case 120 of the SAM emission in Table S1. Same format as Figure S1. Figure S121, Case 121 of the SAM emission in Table S1. Same format as Figure S1. Figure S122, Case 122 of the SAM emission in Table S1. Same format as Figure S1. Figure S123, Case 123 of the SAM emission in Table S1. Same format as Figure S1. Figure S124, Case 124 of the SAM emission in Table S1. Same format as Figure S1. Figure S125, Case 125 of the SAM emission in Table S1. Same format as Figure S1. Figure S126, Case 126 of the SAM emission in Table S1. Same format as Figure S1. Figure S127, Case 127 of the SAM emission in Table S1. Same format as Figure S1. Figure S128, Case 128 of the SAM emission in Table S1. Same format as Figure S1. Figure S129, Case 129 of the SAM emission in Table S1. Same format as Figure S1. Figure S130, Case 130 of the SAM emission in Table S1. Same format as Figure S1. Figure S131, Case 131 of the SAM emission in Table S1. Same format as Figure S1. Figure S132, Case 132 of the SAM emission in Table S1. Same format as Figure S1. Figure S133, Case 133 of the SAM emission in Table S1. Same format as Figure S1. Figure S134, Case 134 of the SAM emission in Table S1. Same format as Figure S1. Figure S135, Case 135 of the SAM emission in Table S1. Same format as Figure S1. Figure S136, Case 136 of the SAM emission in Table S1. Same format as Figure S1. Figure S137, Case 137 of the SAM emission in Table S1. Same format as Figure S1. Figure S138, Case 138 of the SAM emission in Table S1. Same format as Figure S1. Figure S139, Case 139 of the SAM emission in Table S1. Same format as Figure S1. Figure S140, Case 140 of the SAM emission in Table S1. Same format as Figure S1. Figure S141, Case 141 of the SAM emission in Table S1. Same format as Figure S1.

Figure S142, Case 142 of the SAM emission in Table S1. Same format as Figure S1. Figure S143, Case 143 of the SAM emission in Table S1. Same format as Figure S1. Figure S144, Case 144 of the SAM emission in Table S1. Same format as Figure S1. Figure S145, Case 145 of the SAM emission in Table S1. Same format as Figure S1. Figure S146, Case 146 of the SAM emission in Table S1. Same format as Figure S1. Figure S147, Case 147 of the SAM emission in Table S1. Same format as Figure S1. Figure S148, Case 148 of the SAM emission in Table S1. Same format as Figure S1. Figure S149, Case 149 of the SAM emission in Table S1. Same format as Figure S1. Figure S150, Case 150 of the SAM emission in Table S1. Same format as Figure S1. Figure S151, Case 151 of the SAM emission in Table S1. Same format as Figure S1. Figure S152, Case 152 of the SAM emission in Table S1. Same format as Figure S1. Figure S143, Case 153 of the SAM emission in Table S1. Same format as Figure S1. Figure S154, Case 154 of the SAM emission in Table S1. Same format as Figure S1. Figure S155, Case 155 of the SAM emission in Table S1. Same format as Figure S1. Figure S156, Case 156 of the SAM emission in Table S1. Same format as Figure S1. Figure S157, Case 157 of the SAM emission in Table S1. Same format as Figure S1. Figure S158, Case 158 of the SAM emission in Table S1. Same format as Figure S1. Figure S159, Case 159 of the SAM emission in Table S1. Same format as Figure S1. Figure S160, Case 160 of the SAM emission in Table S1. Same format as Figure S1. Figure S161, Case 161 of the SAM emission in Table S1. Same format as Figure S1. Figure S162, Case 162 of the SAM emission in Table S1. Same format as Figure S1. Figure S163, Case 163 of the SAM emission in Table S1. Same format as Figure S1. Figure S164, Case 164 of the SAM emission in Table S1. Same format as Figure S1. Figure S165, Case 165 of the SAM emission in Table S1. Same format as Figure S1. Figure S166, Case 166 of the SAM emission in Table S1. Same format as Figure S1. Figure S167, Case 167 of the SAM emission in Table S1. Same format as Figure S1. Figure S168, Case 168 of the SAM emission in Table S1. Same format as Figure S1. Figure S169, Case 169 of the SAM emission in Table S1. Same format as Figure S1. Figure S170, Case 170 of the SAM emission in Table S1. Same format as Figure S1. Figure S171, Case 171 of the SAM emission in Table S1. Same format as Figure S1. Figure S172, Case 172 of the SAM emission in Table S1. Same format as Figure S1. Figure S173, Case 173 of the SAM emission in Table S1. Same format as Figure S1. Figure S174, Case 174 of the SAM emission in Table S1. Same format as Figure S1. Figure S175, Case 175 of the SAM emission in Table S1. Same format as Figure S1. Figure S176, Case 176 of the SAM emission in Table S1. Same format as Figure S1. Figure S177, Case 177 of the SAM emission in Table S1. Same format as Figure S1. Figure S178, Case 178 of the SAM emission in Table S1. Same format as Figure S1. Figure S179, Case 179 of the SAM emission in Table S1. Same format as Figure S1. Figure S180, Case 180 of the SAM emission in Table S1. Same format as Figure S1. Figure S181, Case 181 of the SAM emission in Table S1. Same format as Figure S1. Figure S182, Case 182 of the SAM emission in Table S1. Same format as Figure S1. Figure S183, Case 183 of the SAM emission in Table S1. Same format as Figure S1. Figure S184, Case 184 of the SAM emission in Table S1. Same format as Figure S1. Figure S185, Case 185 of the SAM emission in Table S1. Same format as Figure S1. Figure S186, Case 186 of the SAM emission in Table S1. Same format as Figure S1. Figure S187, Case 187 of the SAM emission in Table S1. Same format as Figure S1. Figure S188, Case 188 of the SAM emission in Table S1. Same format as Figure S1. Figure S189, Case 189 of the SAM emission in Table S1. Same format as Figure S1. Figure S190, Case 190 of the SAM emission in Table S1. Same format as Figure S1. Figure S191, Case 191 of the SAM emission in Table S1. Same format as Figure S1. Figure S192, Case 192 of the SAM emission in Table S1. Same format as Figure S1. Figure S193, Case 193 of the SAM emission in Table S1. Same format as Figure S1.

Table S1, SAM event list for Manuscript:" Saturn Anomalous Myriametric Radiation, A New Type of Saturn Radio Emission Revealed by Cassini", Columns: No. for the number of the event that corresponding to the event figure in the supplementary material; start_time and end_time give the manually chosen start and end time of each SAM emission; low_freq and up_freq give the lower and upper frequency of each SAM emissions that are marked by the black boxes in the supplementary figures given in units of Hertz; LFE marks whether this SAM emissions is accompanied by a SKR low frequency tension as derived from the criteria in the manuscript.

| **SAM event list** | |  |  |  |  |
| --- | --- | --- | --- | --- | --- |
| **No.** | **start_time** | **end_time** | **low_freq （Hz）** | **up_freq(Hz)** | **LFE** |
| 1 | '2005-11-29/03:18:21.457' | '2005-11-29/09:20:17.538' | 5590 | 14100 | 0 |
| 2 | '2005-11-29/13:26:24.472' | '2005-11-29/22:22:04.271' | 7230 | 14900 | 0 |
| 3 | '2006-05-05/05:29:39.347' | '2006-05-05/23:49:48.593' | 7230 | 17600 | 1 |
| 4 | '2006-05-06/00:44:05.628' | '2006-05-06/15:45:12.412' | 7520 | 15800 | 0 |
| 5 | '2006-08-22/06:14:29.950' | '2006-08-22/16:03:14.774' | 6070 | 11900 | 1 |
| 6 | '2006-10-08/20:33:29.447' | '2006-10-09/11:35:54.874' | 6560 | 17100 | 1 |
| 7 | '2006-10-21/08:44:32.130' | '2006-10-21/18:12:30.920' | 5690 | 9460 | 1 |
| 8 | '2006-10-27/01:34:04.221' | '2006-10-27/14:16:27.381' | 7510 | 14100 | 1 |
| 9 | '2006-11-23/15:30:53.225' | '2006-11-24/00:25:59.022' | 7170 | 11300 | 1 |
| 10 | '2006-12-03/06:10:28.638' | '2006-12-04/04:49:26.834' | 6300 | 20600 | 1 |
| 11 | '2006-12-24/21:30:27.136' | '2006-12-25/03:32:15.678' | 5120 | 12900 | 1 |
| 12 | '2006-12-25/09:00:18.090' | '2006-12-26/04:27:02.724' | 5040 | 14200 | 1 |
| 13 | '2007-01-05/07:08:18.719' | '2007-01-05/20:20:53.923' | 4930 | 15600 | 1 |
| 14 | '2007-01-07/07:37:08.731' | '2007-01-08/05:04:35.763' | 3510 | 9640 | 1 |
| 15 | '2007-02-19/15:28:42.468' | '2007-02-20/06:19:01.607' | 7230 | 15700 | 1 |
| 16 | '2007-02-28/06:09:42.757' | '2007-02-28/15:22:29.208' | 4770 | 10000 | 1 |
| 17 | '2007-02-28/15:52:23.930' | '2007-03-01/04:19:20.047' | 8540 | 15600 | 0 |
| 18 | '2007-03-07/00:12:03.618' | '2007-03-07/10:34:22.312' | 9060 | 17100 | 1 |
| 19 | '2007-03-07/18:17:29.246' | '2007-03-08/12:56:41.005' | 8300 | 17800 | 1 |
| 20 | '2007-03-22/23:56:22.915' | '2007-03-23/10:40:24.121' | 8540 | 15200 | 1 |
| 21 | '2007-03-31/01:14:56.019' | '2007-03-31/17:58:04.331' | 6660 | 12600 | 0 |
| 22 | '2007-04-15/13:33:08.794' | '2007-04-15/17:10:18.442' | 9240 | 15500 | 1 |
| 23 | '2007-04-20/18:49:14.171' | '2007-04-21/00:05:19.548' | 8060 | 13100 | 1 |
| 24 | '2007-04-23/00:56:09.499' | '2007-04-23/09:56:29.146' | 5390 | 15200 | 1 |
| 25 | '2007-04-23/10:54:23.719' | '2007-04-23/19:52:28.291' | 7310 | 15700 | 1 |
| 26 | '2007-05-08/09:07:43.668' | '2007-05-08/22:03:29.546' | 5950 | 18500 | 1 |
| 27 | '2007-05-09/11:06:15.153' | '2007-05-09/13:51:13.067' | 7080 | 16600 | 1 |
| 28 | '2007-05-25/16:34:06.834' | '2007-05-26/04:52:27.638' | 7750 | 14200 | 1 |
| 29 | '2007-06-11/06:09:18.090' | '2007-06-11/12:59:38.191' | 8800 | 20000 | 0 |
| 30 | '2007-06-12/16:41:21.156' | '2007-06-13/05:21:24.925' | 6310 | 18000 | 1 |
| 31 | '2008-01-04/09:52:34.322' | '2008-01-04/14:24:07.035' | 8210 | 14800 | 1 |
| 32 | '2008-01-26/01:37:00.955' | '2008-01-26/04:33:09.447' | 7670 | 16300 | 1 |
| 33 | '2008-01-27/00:27:10.552' | '2008-01-27/10:25:26.432' | 7450 | 28200 | 1 |
| 34 | '2008-02-27/15:31:39.026' | '2008-02-28/07:04:04.874' | 3830 | 17300 | 1 |
| 35 | '2008-02-28/09:28:50.302' | '2008-02-28/19:15:06.281' | 7100 | 12300 | 0 |
| 36 | '2008-03-31/14:57:35.879' | '2008-04-01/03:08:42.362' | 6010 | 15500 | 0 |
| 37 | '2008-04-09/16:42:28.079' | '2008-04-09/23:41:33.597' | 8800 | 15800 | 0 |
| 38 | '2008-04-10/03:46:48.744' | '2008-04-10/12:42:28.543' | 6310 | 14900 | 0 |
| 39 | '2008-04-10/12:45:54.601' | '2008-04-11/00:21:07.239' | 6800 | 12600 | 0 |
| 40 | '2008-04-21/12:53:53.032' | '2008-04-21/19:58:52.434' | 8310 | 16900 | 1 |
| 41 | '2008-05-08/18:52:53.032' | '2008-05-09/00:04:52.434' | 7230 | 22400 | 1 |
| 42 | '2008-05-10/13:19:13.267' | '2008-05-10/21:57:26.533' | 7940 | 21500 | 0 |
| 43 | '2008-05-16/02:59:41.656' | '2008-05-16/12:16:27.117' | 7380 | 13200 | 1 |
| 44 | '2008-06-02/04:34:14.006' | '2008-06-02/14:40:18.018' | 5900 | 15700 | 1 |
| 45 | '2008-06-15/04:02:48.090' | '2008-06-15/15:15:55.327' | 7520 | 13300 | 1 |
| 46 | '2008-06-29/06:31:55.706' | '2008-06-30/00:18:10.491' | 5660 | 13600 | 1 |
| 47 | '2008-07-10/03:34:49.347' | '2008-07-10/09:09:07.487' | 6130 | 12300 | 1 |
| 48 | '2008-09-01/12:32:56.305' | '2008-09-01/19:25:56.129' | 3850 | 8190 | 1 |
| 49 | '2008-09-01/21:19:10.733' | '2008-09-02/14:30:05.279' | 7700 | 20800 | 0 |
| 50 | '2008-09-10/18:37:07.144' | '2008-09-11/14:20:52.504' | 6600 | 19600 | 1 |
| 51 | '2008-09-15/07:12:53.006' | '2008-09-15/13:11:09.347' | 6380 | 11900 | 1 |
| 52 | '2008-10-02/16:08:18.299' | '2008-10-03/10:57:19.953' | 4680 | 19300 | 1 |
| 53 | '2008-10-03/17:52:51.801' | '2008-10-04/07:15:32.833' | 5690 | 19500 | 1 |
| 54 | '2008-10-10/02:57:26.710' | '2008-10-10/20:36:32.516' | 5790 | 19800 | 0 |
| 55 | '2008-10-15/00:00:00.000' | '2008-10-15/09:02:42.814' | 6500 | 19100 | 1 |
| 56 | '2008-10-23/08:21:14.040' | '2008-10-23/17:07:43.804' | 7690 | 14500 | 1 |
| 57 | '2008-10-30/13:00:22.086' | '2008-10-31/01:17:36.985' | 5480 | 14200 | 0 |
| 58 | '2008-11-01/00:03:27.765' | '2008-11-01/19:31:00.176' | 5530 | 20200 | 0 |
| 59 | '2008-11-15/08:59:46.065' | '2008-11-16/06:47:50.710' | 6130 | 14300 | 0 |
| 60 | '2008-11-17/10:49:19.509' | '2008-11-17/16:47:06.994' | 7610 | 12700 | 1 |
| 61 | '2008-11-24/22:43:26.135' | '2008-11-25/06:43:26.135' | 7160 | 12100 | 0 |
| 62 | '2008-12-02/18:33:07.730' | '2008-12-03/14:52:16.196' | 7300 | 15600 | 1 |
| 63 | '2008-12-08/20:38:24.000' | '2008-12-09/04:52:38.710' | 5960 | 19300 | 1 |
| 64 | '2008-12-10/00:04:38.710' | '2008-12-11/06:29:15.871' | 7170 | 15300 | 1 |
| 65 | '2008-12-23/19:51:36.633' | '2008-12-24/13:57:03.484' | 8390 | 15300 | 1 |
| 66 | '2009-01-02/06:05:09.202' | '2009-01-02/20:25:01.840' | 9800 | 21000 | 1 |
| 67 | '2009-02-21/00:17:40.123' | '2009-02-21/17:44:32.393' | 8170 | 18500 | 1 |
| 68 | '2009-03-06/23:51:37.090' | '2009-03-07/09:49:25.724' | 7200 | 19700 | 1 |
| 69 | '2009-03-18/03:18:23.437' | '2009-03-18/13:45:24.176' | 7370 | 14900 | 0 |
| 70 | '2009-03-31/16:32:37.724' | '2009-04-01/05:41:07.425' | 7300 | 16600 | 0 |
| 71 | '2009-04-08/05:27:46.768' | '2009-04-08/21:36:40.540' | 7440 | 21300 | 1 |
| 72 | '2009-05-09/12:49:39.660' | '2009-05-10/03:47:36.774' | 6130 | 19100 | 0 |
| 73 | '2009-05-16/22:55:12.883' | '2009-05-17/11:57:48.246' | 7040 | 18800 | 1 |
| 74 | '2009-05-23/06:13:52.161' | '2009-05-23/10:51:15.377' | 6120 | 12650 | 0 |
| 75 | '2009-05-25/00:16:48.422' | '2009-05-25/11:03:24.809' | 7580 | 17700 | 1 |
| 76 | '2009-06-06/10:47:32.129' | '2009-06-07/02:00:41.384' | 7440 | 18200 | 0 |
| 77 | '2009-06-14/22:33:25.865' | '2009-06-15/04:13:57.818' | 8160 | 20800 | 1 |
| 78 | '2009-06-27/19:58:47.638' | '2009-06-28/15:16:34.975' | 6380 | 14400 | 1 |
| 79 | '2009-07-14/08:16:53.065' | '2009-07-14/17:55:46.734' | 8360 | 14400 | 1 |
| 80 | '2011-04-19/06:39:11.759' | '2011-04-19/11:21:24.422' | 6500 | 12600 | 1 |
| 81 | '2012-10-13/01:26:19.977' | '2012-10-13/14:31:57.588' | 8700 | 24100 | 1 |
| 82 | '2012-10-13/15:22:36.784' | '2012-10-13/23:51:37.090' | 11300 | 17200 | 1 |
| 83 | '2012-10-18/05:45:19.907' | '2012-10-18/16:45:49.749' | 8530 | 17600 | 1 |
| 84 | '2012-10-18/18:12:39.799' | '2012-10-19/01:31:39.497' | 4580 | 12300 | 1 |
| 85 | '2012-11-06/13:34:26.123' | '2012-11-06/18:13:49.802' | 8850 | 15700 | 1 |
| 86 | '2012-11-06/19:45:11.525' | '2012-11-07/02:19:08.312' | 9690 | 19600 | 1 |
| 87 | '2012-11-23/11:48:25.760' | '2012-11-23/22:47:54.971' | 7750 | 16200 | 1 |
| 88 | '2012-11-23/21:50:55.180' | '2012-11-24/05:21:51.758' | 8290 | 16400 | 1 |
| 89 | '2012-11-27/11:53:58.191' | '2012-11-27/20:30:09.045' | 6500 | 17200 | 1 |
| 90 | '2012-12-08/07:50:21.106' | '2012-12-08/14:52:27.739' | 8030 | 17800 | 0 |
| 91 | '2012-12-19/14:40:05.588' | '2012-12-20/02:35:54.133' | 7580 | 16500 | 1 |
| 92 | '2012-12-24/01:48:06.334' | '2012-12-24/15:45:40.223' | 6910 | 17900 | 1 |
| 93 | '2013-01-05/14:17:24.739' | '2013-01-06/04:00:37.161' | 6300 | 20800 | 1 |
| 94 | '2013-02-08/18:52:23.190' | '2013-02-09/00:54:28.917' | 8230 | 14600 | 1 |
| 95 | '2013-02-22/22:15:04.523' | '2013-02-23/08:37:23.216' | 7950 | 16600 | 1 |
| 96 | '2013-03-07/23:33:28.040' | '2013-03-08/08:48:14.472' | 9430 | 17900 | 1 |
| 97 | '2013-03-15/23:56:37.302' | '2013-03-16/11:20:43.636' | 7940 | 16000 | 1 |
| 98 | '2013-03-16/08:08:26.533' | '2013-03-16/19:35:52.764' | 10000 | 18700 | 0 |
| 99 | '2013-03-17/01:30:31.432' | '2013-03-17/06:08:48.056' | 9130 | 16700 | 1 |
| 100 | '2013-03-19/21:37:51.484' | '2013-03-20/07:10:08.516' | 9290 | 18400 | 1 |
| 101 | '2013-03-22/01:19:35.879' | '2013-03-23/06:02:12.515' | 6590 | 15200 | 0 |
| 102 | '2013-03-28/01:05:07.538' | '2013-03-28/07:45:31.658' | 8620 | 13800 | 1 |
| 103 | '2013-03-28/10:15:04.521' | '2013-03-28/22:04:13.266' | 10500 | 15900 | 0 |
| 104 | '2013-03-29/02:24:43.417' | '2013-03-29/08:02:56.687' | 10600 | 20700 | 0 |
| 105 | '2013-04-01/03:02:43.446' | '2013-04-01/10:30:18.859' | 8620 | 18400 | 1 |
| 106 | '2013-04-03/01:14:35.902' | '2013-04-03/15:23:49.146' | 8800 | 17600 | 0 |
| 107 | '2013-04-08/07:03:18.995' | '2013-04-08/11:00:29.336' | 8970 | 16000 | 0 |
| 108 | '2013-04-29/02:25:50.640' | '2013-04-29/22:51:15.377' | 8450 | 18300 | 1 |
| 109 | '2013-04-29/23:34:40.402' | '2013-04-30/07:03:18.995' | 10200 | 19800 | 1 |
| 110 | '2013-04-30/12:18:05.427' | '2013-04-30/17:40:06.030' | 9060 | 22400 | 0 |
| 111 | '2013-05-02/08:54:28.712' | '2013-05-02/13:49:48.126' | 8360 | 15800 | 1 |
| 112 | '2013-05-08/04:22:54.874' | '2013-05-08/11:44:19.296' | 9250 | 16600 | 0 |
| 113 | '2013-05-21/02:24:50.674' | '2013-05-21/23:53:14.604' | 7170 | 18400 | 1 |
| 114 | '2013-06-13/22:04:53.067' | '2013-06-14/16:35:24.950' | 9200 | 20600 | 1 |
| 115 | '2013-06-26/02:29:11.804' | '2013-06-26/15:36:15.087' | 7170 | 18100 | 1 |
| 116 | '2013-07-02/10:54:37.299' | '2013-07-02/17:57:47.485' | 7940 | 17100 | 1 |
| 117 | '2013-07-02/21:27:27.031' | '2013-07-03/02:24:10.058' | 9120 | 16600 | 0 |
| 118 | '2013-07-03/08:21:14.040' | '2013-07-03/13:09:12.147' | 9370 | 16300 | 0 |
| 119 | '2013-07-22/09:24:25.327' | '2013-07-23/01:55:46.734' | 9150 | 15600 | 1 |
| 120 | '2013-07-23/06:50:03.015' | '2013-07-23/20:42:12.663' | 7260 | 16100 | 0 |
| 121 | '2013-08-04/05:28:34.086' | '2013-08-04/23:31:03.317' | 8950 | 18400 | 1 |
| 122 | '2013-08-05/00:03:37.085' | '2013-08-05/23:05:43.719' | 9900 | 21100 | 1 |
| 123 | '2013-08-15/10:39:11.759' | '2013-08-15/23:02:06.633' | 11500 | 17800 | 0 |
| 124 | '2013-08-27/04:08:26.533' | '2013-08-27/12:22:54.874' | 6980 | 11700 | 0 |
| 125 | '2013-09-07/08:18:17.947' | '2013-09-07/23:12:11.824' | 6660 | 13300 | 1 |
| 126 | '2013-09-09/15:23:49.146' | '2013-09-09/22:59:41.910' | 9630 | 14100 | 0 |
| 127 | '2013-10-21/23:06:21.374' | '2013-10-22/10:00:08.382' | 8390 | 15400 | 1 |
| 128 | '2013-11-01/20:18:05.427' | '2013-11-02/00:38:35.578' | 5770 | 13500 | 0 |
| 129 | '2013-11-14/16:34:58.492' | '2013-11-14/21:38:53.668' | 7790 | 13400 | 1 |
| 130 | '2013-11-26/20:59:15.660' | '2013-11-27/11:20:38.569' | 12400 | 22100 | 1 |
| 131 | '2013-12-04/21:17:23.539' | '2013-12-05/07:38:29.197' | 8410 | 22600 | 1 |
| 132 | '2013-12-05/10:16:54.203' | '2013-12-05/17:49:31.362' | 9400 | 20000 | 0 |
| 133 | '2013-12-05/19:52:44.144' | '2013-12-06/04:40:47.497' | 10400 | 19100 | 0 |
| 134 | '2013-12-25/17:15:59.721' | '2013-12-26/07:14:10.757' | 11320 | 19500 | 1 |
| 135 | '2014-01-04/11:23:49.146' | '2014-01-04/18:41:29.406' | 9820 | 18100 | 1 |
| 136 | '2014-01-04/18:31:25.914' | '2014-01-05/05:58:44.563' | 9330 | 18100 | 1 |
| 137 | '2014-02-22/03:25:01.508' | '2014-02-22/17:46:07.839' | 7720 | 13300 | 0 |
| 138 | '2014-03-08/05:11:48.265' | '2014-03-08/12:54:28.917' | 5850 | 12190 | 1 |
| 139 | '2014-03-08/17:00:29.982' | '2014-03-08/23:07:38.182' | 7300 | 12300 | 0 |
| 140 | '2014-03-26/13:47:20.201' | '2014-03-26/21:13:34.070' | 13500 | 18700 | 1 |
| 141 | '2014-04-09/12:24:18.440' | '2014-04-10/20:20:23.749' | 6470 | 14700 | 1 |
| 142 | '2014-05-22/01:26:50.050' | '2014-05-22/11:50:22.311' | 11700 | 15600 | 1 |
| 143 | '2014-05-29/19:55:15.017' | '2014-05-30/12:32:33.769' | 7040 | 16600 | 1 |
| 144 | '2014-06-16/06:50:03.015' | '2014-06-16/20:06:01.809' | 9720 | 18300 | 0 |
| 145 | '2014-07-16/13:23:12.965' | '2014-07-16/23:40:42.211' | 15300 | 25000 | 1 |
| 146 | '2014-07-26/09:55:10.311' | '2014-07-27/00:44:30.545' | 7940 | 15300 | 1 |
| 147 | '2014-11-21/09:43:43.116' | '2014-11-21/17:02:42.814' | 7790 | 14200 | 1 |
| 148 | '2014-12-04/16:50:39.196' | '2014-12-04/22:21:06.332' | 10200 | 16400 | 0 |
| 149 | '2016-02-23/13:30:27.136' | '2016-02-23/18:36:47.035' | 7790 | 14500 | 0 |
| 150 | '2016-05-24/21:01:30.452' | '2016-05-25/09:53:22.010' | 13400 | 25000 | 1 |
| 151 | '2016-06-05/12:51:56.481' | '2016-06-05/20:00:38.850' | 6660 | 15700 | 1 |
| 152 | '2016-07-22/12:02:30.873' | '2016-07-22/22:11:02.577' | 7720 | 17900 | 1 |
| 153 | '2016-07-23/02:04:53.364' | '2016-07-23/08:43:01.607' | 9730 | 16900 | 1 |
| 154 | '2016-07-23/11:55:48.545' | '2016-07-23/22:49:19.509' | 9820 | 20800 | 0 |
| 155 | '2016-08-02/12:57:36.000' | '2016-08-02/23:36:05.912' | 13200 | 19100 | 1 |
| 156 | '2016-08-07/20:33:51.902' | '2016-08-08/08:24:17.736' | 9910 | 18800 | 1 |
| 157 | '2016-08-16/09:56:21.255' | '2016-08-17/04:27:48.880' | 8870 | 16300 | 1 |
| 158 | '2016-08-17/09:13:37.126' | '2016-08-17/22:19:34.804' | 8780 | 14900 | 1 |
| 159 | '2016-08-25/19:25:01.508' | '2016-08-27/05:31:04.399' | 5690 | 17500 | 0 |
| 160 | '2016-09-08/10:22:42.299' | '2016-09-08/21:52:09.648' | 9130 | 15600 | 1 |
| 161 | '2016-09-10/14:01:48.543' | '2016-09-10/17:58:11.457' | 8530 | 13500 | 0 |
| 162 | '2016-09-24/08:50:39.196' | '2016-09-24/22:16:16.884' | 9910 | 17400 | 1 |
| 163 | '2016-10-02/11:08:54.194' | '2016-10-03/12:56:09.853' | 8160 | 17400 | 1 |
| 164 | '2016-10-04/08:38:34.135' | '2016-10-04/22:26:15.132' | 7700 | 15600 | 1 |
| 165 | '2016-10-11/06:51:38.745' | '2016-10-11/22:06:37.990' | 11300 | 17600 | 0 |
| 166 | '2016-10-12/01:19:53.806' | '2016-10-12/09:36:28.945' | 9200 | 16800 | 0 |
| 167 | '2016-10-23/20:02:40.469' | '2016-10-24/03:21:51.202' | 11300 | 23000 | 1 |
| 168 | '2016-11-01/18:39:39.167' | '2016-11-02/19:46:44.020' | 9640 | 15400 | 1 |
| 169 | '2016-11-09/08:53:46.276' | '2016-11-09/23:15:09.185' | 8310 | 15600 | 1 |
| 170 | '2016-11-24/13:47:20.201' | '2016-11-24/23:55:10.553' | 8530 | 16900 | 1 |
| 171 | '2016-11-25/07:06:56.080' | '2016-11-25/22:33:09.950' | 8790 | 15800 | 1 |
| 172 | '2016-11-26/04:44:37.387' | '2016-11-26/12:32:33.769' | 7640 | 15100 | 1 |
| 173 | '2016-11-27/12:19:00.176' | '2016-11-28/15:18:46.380' | 5790 | 13600 | 1 |
| 174 | '2016-12-26/13:53:30.651' | '2016-12-26/16:17:10.381' | 11100 | 17600 | 0 |
| 175 | '2017-01-09/18:41:57.791' | '2017-01-10/08:02:56.687' | 9240 | 19300 | 1 |
| 176 | '2017-01-16/19:46:37.654' | '2017-01-17/10:03:40.859' | 9530 | 19500 | 1 |
| 177 | '2017-01-22/15:02:06.633' | '2017-01-22/22:35:34.673' | 5110 | 9430 | 1 |
| 178 | '2017-02-06/04:07:21.718' | '2017-02-06/19:49:41.595' | 12200 | 17200 | 0 |
| 179 | '2017-02-12/17:07:32.261' | '2017-02-12/22:25:55.779' | 9530 | 14200 | 1 |
| 180 | '2017-02-21/19:05:31.288' | '2017-02-22/05:20:58.896' | 9630 | 16700 | 0 |
| 181 | '2017-02-28/19:58:31.656' | '2017-03-01/03:08:27.975' | 6940 | 11000 | 0 |
| 182 | '2017-03-06/20:07:21.718' | '2017-03-07/05:32:45.644' | 11300 | 18300 | 1 |
| 183 | '2017-03-08/01:38:54.342' | '2017-03-08/15:25:39.238' | 11320 | 17500 | 1 |
| 184 | '2017-03-27/11:14:06.545' | '2017-03-27/17:54:34.372' | 10200 | 15100 | 1 |
| 185 | '2017-03-27/22:58:29.548' | '2017-03-28/07:03:18.995' | 12400 | 19500 | 1 |
| 186 | '2017-03-28/08:54:28.712' | '2017-03-29/00:30:55.215' | 7080 | 13800 | 1 |
| 187 | '2017-04-18/06:27:34.522' | '2017-04-19/02:22:08.516' | 8390 | 16300 | 1 |
| 188 | '2017-05-27/21:28:02.412' | '2017-05-28/08:37:23.216' | 7100 | 15100 | 1 |
| 189 | '2017-05-28/17:29:14.774' | '2017-05-29/03:58:47.638' | 16300 | 34700 | 0 |
| 190 | '2017-06-16/14:34:36.074' | '2017-06-16/20:25:01.840' | 12400 | 23900 | 1 |
| 191 | '2017-07-02/17:57:47.485' | '2017-07-03/07:09:56.319' | 16900 | 33900 | 0 |
| 192 | '2017-07-22/07:57:03.313' | '2017-07-22/20:45:38.650' | 11000 | 14600 | 1 |
| 193 | '2017-09-14/19:14:21.350' | '2017-09-15/07:09:56.319' | 16100 | 21200 | 1 |
